# Nucleotide-Derived Competitive Inhibitors of Ectonucleotidase CD39—A Promising Extracellular Target for Immunotherapy of Cancer

**DOI:** 10.1021/acs.jmedchem.6c00054

**Published:** 2026-05-01

**Authors:** Chunyang Bi, Florian Schwermer, Laura Schäkel, Salahuddin Mirza, Helay Baburi, Patrick Riziki, Constanze C. Schmies, Riekje Winzer, Riham Idris, Georg Rolshoven, Julia Schilling, Leon Luckenbach, Julie Pelletier, Luca Svolacchia Brusoni, Haneen Al Hroub, Ghazl Al Hamwi, Vittoria Lopez, Areso Ahmadsay, Luca Raulien, Katharina Sylvester, Jean Sévigny, Eva Tolosa, Andreas H. Guse, Christa E. Müller

**Affiliations:** † PharmaCenter Bonn, Pharmaceutical Institute, Pharmaceutical Sciences Bonn (PSB), Pharmaceutical & Medicinal Chemistry, 9374 University of Bonn, 53121 Bonn, Germany; ‡ Department of Immunology, Hamburg Center for Translational Immunology, University Medical Center Hamburg-Eppendorf, 20246 Hamburg, Germany; § Axe Maladies Infectieuses et Immunitaires, Centre de Recherche du CHU de Québec − Université Laval, Québec City, Quebec G1V 4G2, Canada; ∥ Départment de Microbiologie-Infectiologie et d’Immunologie, Centres PROTEO-ULaval et ARThrite, Faculté de Médecine, 4440 Université Laval, Quebec City, Quebec G1V 0A6, Canada; ⊥ The Calcium Signalling Group, Department of Biochemistry and Molecular Cell Biology, University Medical Center Hamburg-Eppendorf, 20246 Hamburg, Germany

## Abstract

Ectonucleotidases catalyze the hydrolysis of extracellular nucleotides, maintaining the balance between proinflammatory ATP and immunosuppressive adenosine. In the present study, we developed potent competitive inhibitors of the main ATP-hydrolyzing ectoenzyme nucleoside triphosphate diphosphohydrolase-1 (NTPDase1, CD39) based on 8-butylthio-AMP as a lead structure. Altogether, 88 purine nucleotides and analogs with broad structural modifications were synthesized, 78 of which are new compounds. 8-Substitution of the purine scaffold with bulky residues is essential for high potency and confers metabolic stability. 8-(1-Naphthylthio)-*N* ^6^-(4-phenylbutyl)-AMP (**42b**, PSB-24379) is the most potent CD39 inhibitor of the series (*K*_i_ 77.4 nM), showing ancillary CD73 inhibition (*K*_i_ 240 nM). Docking into a human CD39 homology model rationalized key interactions. PSB-24379 reduced ATP hydrolysis in melanoma and breast cancer cell membranes and partially reverted ATP-mediated effects on T cell activation and proliferation in an ATP-rich environment. These CD39 inhibitors represent high-quality tool compounds with potential as drugs for immunotherapy of cancer.

## Graphical abstract

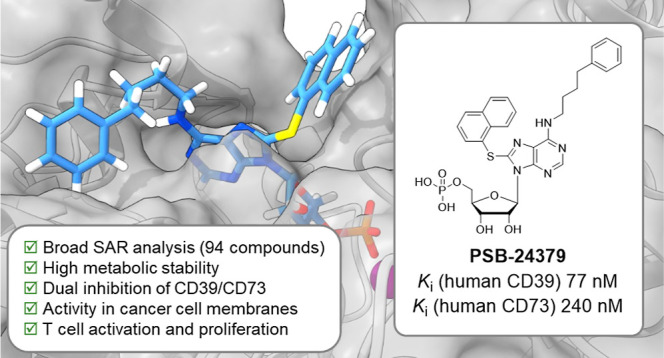

## Introduction

Nucleoside triphosphate diphosphohydrolases (NTPDases) are membrane-bound nucleotidases that are divided into eight subtypes. Four of these, NTPDase1, −2, −3 and −8, are located in the cell membrane acting as ectonucleotidases, whereas the other members are mostly integrated into membranes of intracellular organelles.^1^ NTPDase1 (CD39, EC 3.6.1.5) catalyzes the hydrolysis of nucleoside di- and triphosphates producing the corresponding monophosphates.^2^ The enzyme is often colocalized with ecto-5′-nucleotidase (CD73),^3^ which subsequently hydrolyzes nucleoside monophosphates to the corresponding nucleosides. Together, CD39 and CD73 represent the canonical pathway to convert extracellular proinflammatory ATP and prothrombotic ADP to immunosuppressive, tumor growth-stimulating, angiogenic, and pro-metastatic adenosine (Figure 1).^4,5^

**Figure 1:**
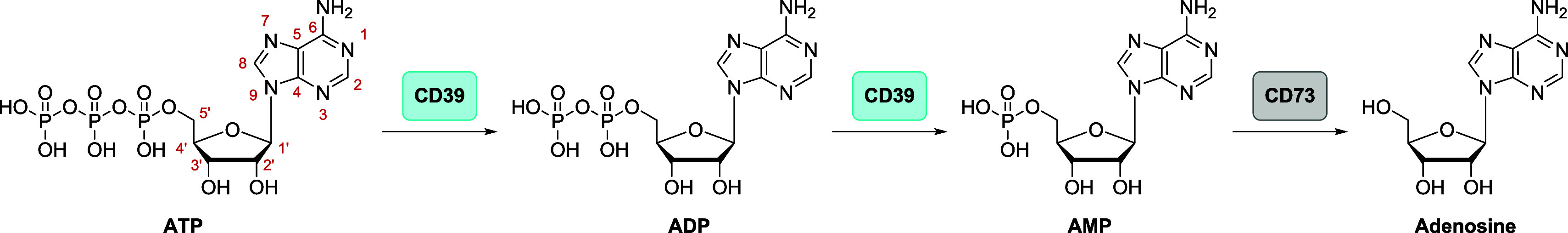
Extracellular hydrolysis of ATP and ADP to AMP by CD39, followed by CD73-catalyzed hydrolysis of AMP to adenosine.

This pathway is upregulated in many tumors, thereby promoting immune evasion, proliferation, angiogenesis, and metastasis.^6–10^ In the tumor microenvironment, extracellular ATP activates nucleotide-activated P2 receptors that can lead to immunostimulatory effects, while adenosine activates P1 receptors, leading to inhibition of immune responses.^4^ Both ectonucleotidases are additionally expressed by immune cells such as lymphocytes and monocytes, acting as regulators of inflammation and immunity.^11,12^ Therefore, CD39 and CD73 have emerged as promising purinergic immune checkpoints in cancer therapy and may also be useful for the treatment of chronic infections.^5,7,13,14,15,16,17^ For target validation studies, potent, selective, and metabolically stable inhibitors of CD39 are required. Dual inhibitors of CD39 and CD73 are expected to display additive or even synergistic effects, since the substrate of CD73, extracellular AMP, may additionally be formed by alternative ectonucleotidases, e.g., nucleotide pyrophosphatase/phosphodiesterase-1 (NPP1, CD203a) and −3 (NPP3, CD203c), and inhibition of CD73 will block the formation of adenosine from AMP produced by different pathways.^18,19^

Despite the availability of crystal structures for human NTPDase2,^20^ rat CD39,^21^ and several bacterial NTPDases, and a homology model for human CD39,^22^ potent and selective small-molecule CD39 inhibitors are currently not available.^2,23^ Over the past decades, several CD39 inhibitors have been described, including nucleotide analogs such as *N* ^6^-diethyl- *d* -β,γ-dibromomethylene-ATP (ARL67156, **1a**), 8-butylthio-AMP (**1b**), and the hybrid of **1a** and **1b**, PSB-172102 (**1c**) (Figure 2), as well as non-nucleotidic inhibitors, e.g., suramin, polyoxometalates (PSB–POM-142), sulfo-anthraquinone derivatives (Reactive Blue 2), and pyridoxalphosphate-6-azophenyl-2′,4′-disulfonic acids (PPADS).^23–33^ Moreover, biologics such as the anti-CD39 monoclonal antibodies Perenostobart (SRF617),^34^ TTX-030,^35^ IPH5201,^36^ ES002023,^37^ PUR001,^38^ and JS019,^39^ the bispecific anti-CD39/TGF-β antibody ES014,^40^ and the recombinant human CD39 enzyme TIN816,^41^ have been developed and advanced to clinical trials. Anti-CD39 antibodies display high inhibitory potency (e.g., TTX-030 shows subnanomolar potency) and long-lasting effects, but are limited by poor tissue penetration and lacking peroral bioavailability.^35,42^ Although small-molecule CD39 inhibitors may be less potent, they offer advantages such as superior tissue penetration, reversible binding, and the potential for oral bioavailability.

**Figure 2:**
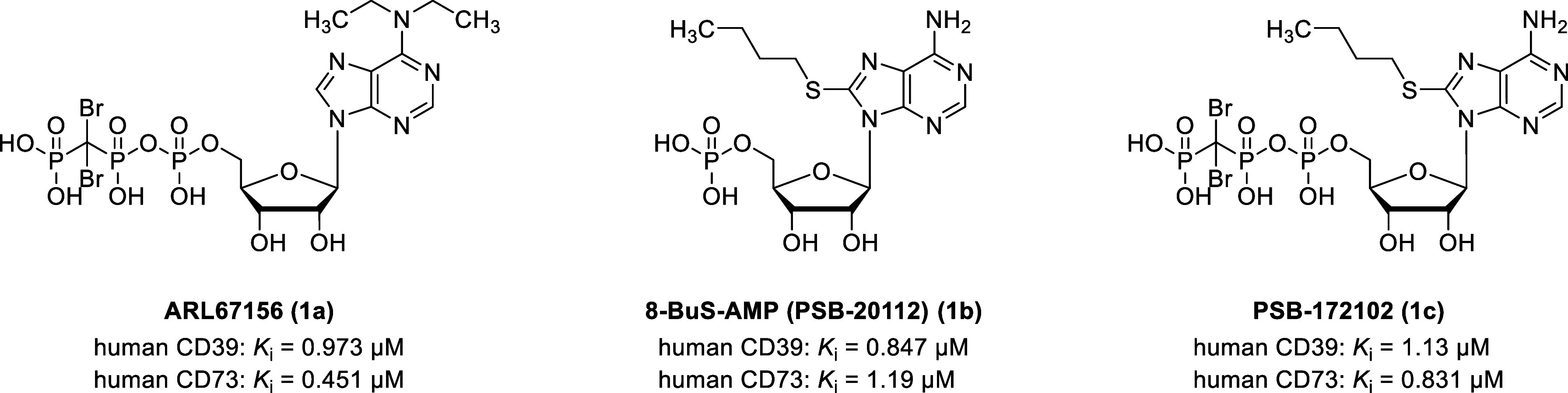
Structures of adenine nucleotide-derived inhibitors of CD39.^43,44^

All representative examples of non-nucleotidic inhibitors suffer from low selectivity, off-target effects, weak or moderate inhibitory potency and/or poor druglikeness.^45^ Nucleotide-based inhibitors exhibit a competitive enzyme inhibition mode and often show ancillary inhibition of CD73. In recent studies, we demonstrated that compounds **1a**–**c** inhibit CD39 with *K*_i_ values of approximately 1 μM (Figure 2).^43,44^ Among these, only **1b** displays high metabolic stability (Figure 3A) and therefore represents a suitable lead structure for the development of tool compounds and drugs that can be applied in vivo. Due to its superior properties, we selected 8-butylthio-AMP (**1b**) to study structure–activity relationships (SARs) regarding substituents at the 1-, 2-, 6-, and 8-positions of its purine core (Figure 3B). Selected potent CD39 inhibitors were further evaluated for selectivity, metabolic stability, ATP-inhibitory activity in cancer cells and at truncated soluble CD39, and effects on T cells. A docking study utilizing the published homology model of human CD39 (Figure 3C)^22^ was performed to rationalize SARs and to provide guidance for future optimization studies.

**Figure 3:**
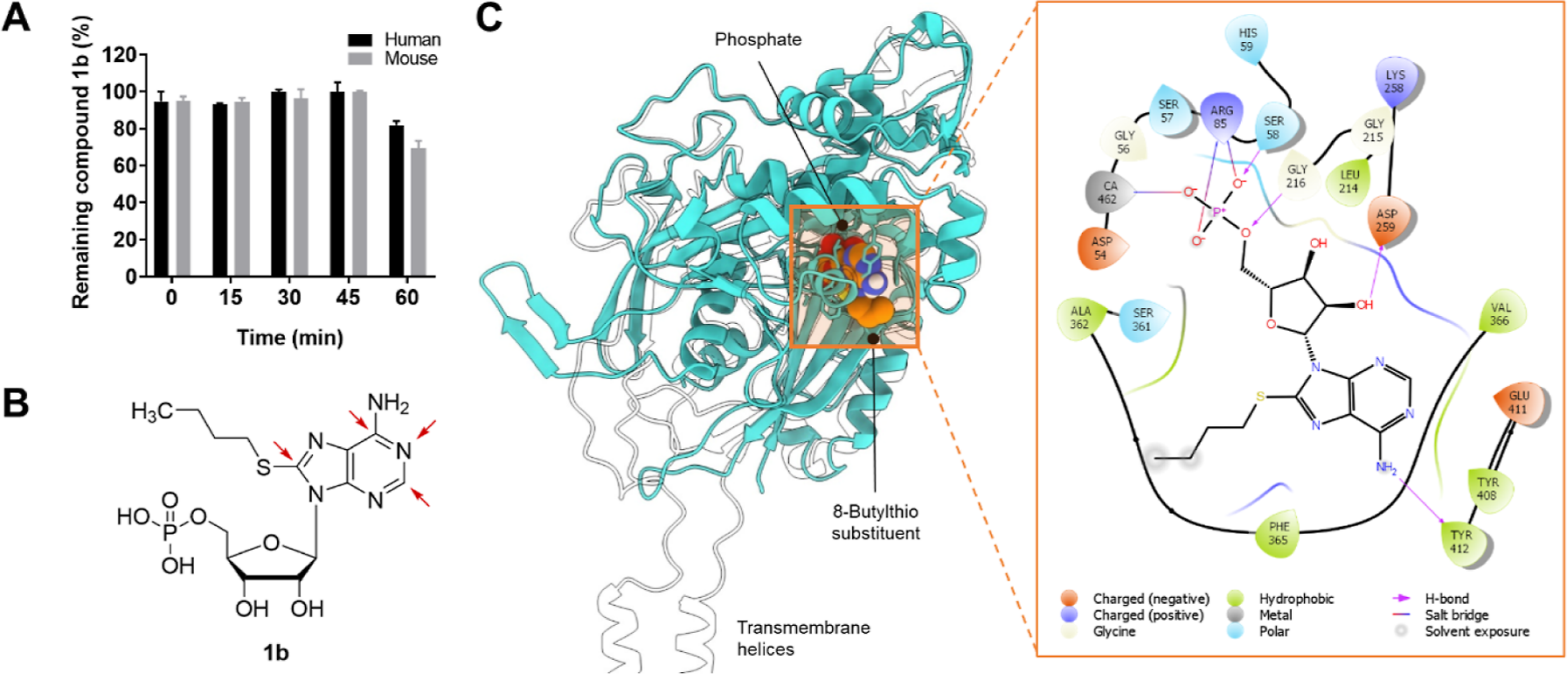
(A) Metabolic stability of 8-butylthio-AMP (**1b**) in human and mouse liver microsomes, adapted from Bi et al.^44^ Copyright 2025 American Chemical Society. (B) Structure of 8-butylthio-AMP (**1b**) with red arrows indicating the investigated positions in this study. (C) (Left) Modeled binding pose of **1b** (spheres) in a human CD39 homology model based on PDB 3ZX3 (cyan cartoon),^21,22^ superimposed with its AlphaFold3 model (UniProt P49961, white contour) using the mmaker command in ChimeraX to visualize the membrane-binding domains. (Right) Ligand interaction diagram of **1b** in the binding pocket of the homology model,^22,44^ as visualized in Schrödinger Maestro.

## Results and Discussion

### Chemistry

A series of AMP derivatives and analogs bearing diverse substituents was designed to explore the SARs of the lead compound 8-butylthio-AMP (**1b**, 8-BuS-AMP). Modifications at the 8-position focused on systematic variation of the butylthio chain. In parallel, substituents were introduced at the 2-position to assess the spatial and electronic tolerance of the binding site regarding this position. The 6-position was functionalized employing substituents that differ in steric demand and electronic character. Beneficial substitutions were subsequently combined to evaluate potential additive effects. The structures of the synthesized nucleosides and nucleotides were confirmed by ^1^H-, ^13^C-, and ^31^P NMR spectroscopy and HRMS analysis. The purity of all nucleotides was greater than 95% as determined by HPLC-UV-ESI-MS.

### Synthesis of 2-Substituted AMP Derivatives

The effect of substituents in the 2-position was investigated by introducing chloro, amino or hydrazino groups (Scheme 1). The commercial 2-aminoadenosine (**2a**) and 2-chloroadenosine (**2b**) were phosphorylated using a modified Yoshikawa procedure.^46,47^ Briefly, the nucleosides were treated with phosphoryl trichloride (POCl_3_) and 1,8-bis­(dimethylamino)­naphthalene (proton-sponge) in trimethyl phosphate (PO­(OCH_3_)_3_) to generate the dichlorophosphate intermediates, which were subsequently hydrolyzed to obtain the nucleoside monophosphates **3a** and **3c**. Compound **2b** was further reacted with hydrazine hydrate to generate the adenosine derivative **2c**,^48^ which was subsequently phosphorylated to yield **3b** (Scheme 1A, B).

**Scheme 1:**
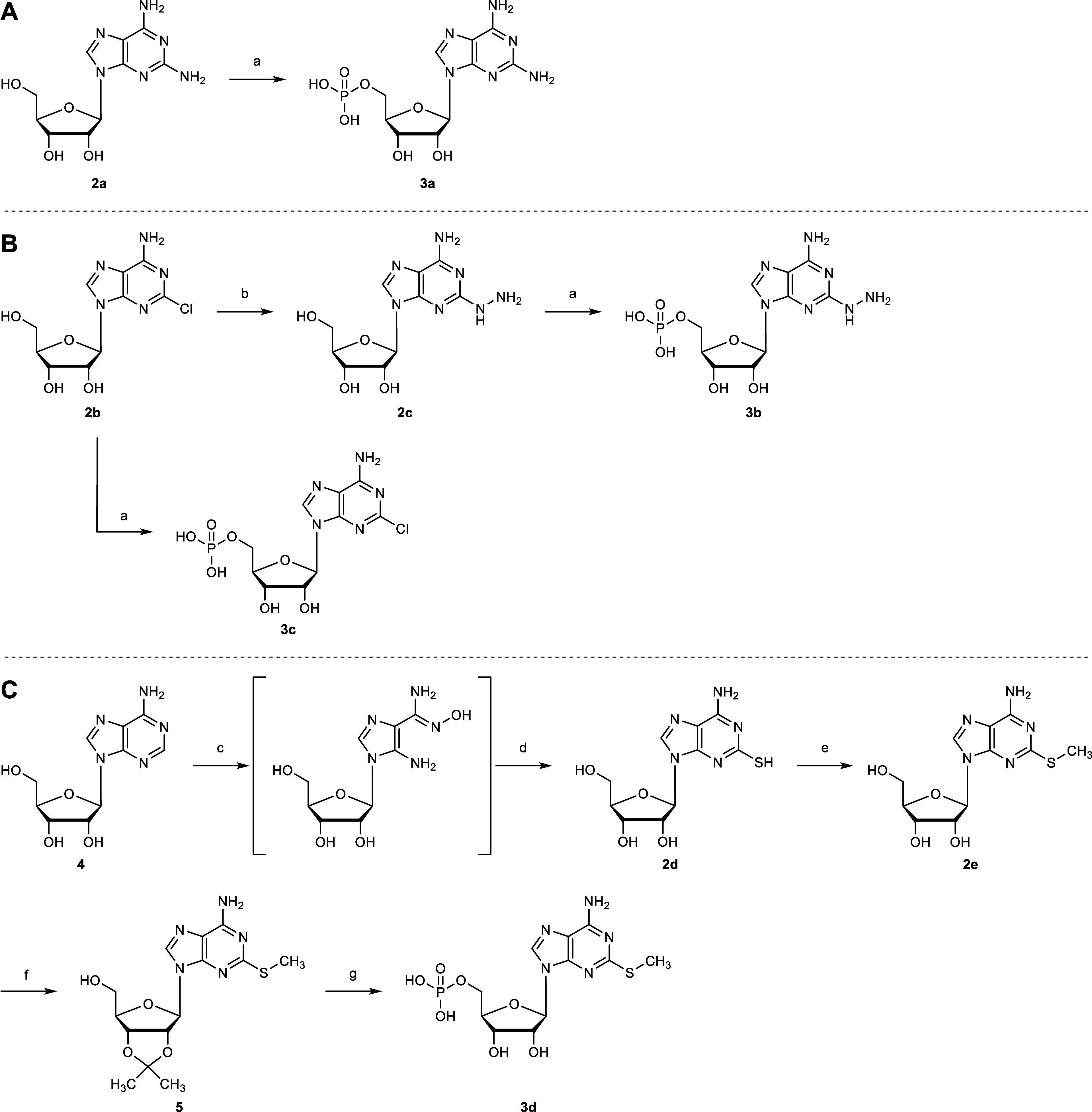
Synthesis of 2-Substituted AMP Derivatives **3a–d** ^a^

a Reagents and conditions: (a) two steps: (i) POCl_3_, proton sponge, PO­(OCH_3_)_3_, 0 °C, 4 h; (ii) 0.5 M TEAC buffer (pH 7.4–7.6), rt, 1 h, yield 45–84%; (b) hydrazine hydrate, 8 h, rt, yield 100%; (c) two steps: (i) CH_3_COOH, 35% H_2_O_2_ (aq.), 50 °C, overnight; (ii) 5 M NaOH (aq.), reflux, 15 min; (d) CS_2_/MeOH/H_2_O (2:7:1), 120 °C, 5 h, yield 26%; (e) CH_3_I, H_2_O/EtOH (1:1), NaOH, rt, 20 min, yield 100%; (f) 2,2-dimethoxypropane, H_2_SO_4_, acetone, rt, 15 min, quantitative yield; (g) three steps: (i) POCl_3_, PO­(OCH_3_)_3_, proton-sponge, 0 °C, 4 h; (ii) 0.5 M TEAC buffer (pH 7.4–7.6), rt, 1 h; (iii) 7% TFA in DCM/H_2_O (9:1), rt, 2 h, yield 8%.

2-Methylthioadenosine (**2e**) was obtained by a three-step procedure from adenosine (**4**) (Scheme 1C).^49^ After oxidative ring-opening of **4** with H_2_O_2_ in acetic acid, the crude intermediate was treated with CS_2_, MeOH and H_2_O at 120 °C in an autoclave to generate adenosine derivative **2d**. Subsequent alkylation of **2d** with methyl iodide in the presence of NaOH in H_2_O generated 2-methylthioadenosine (**2e**). After acetonide protection of the 2′- and 3′-hydroxy groups, intermediate **5** was phosphorylated and then deprotected to generate nucleoside monophosphate **3d**.

### Synthesis of *N* ^6^-Substituted AMP Derivatives

A series of *N* ^6^-mono- or disubstituted AMP derivatives (**8a**–**w**) was synthesized according to Scheme 2. To this end, commercially available 6-chloro-9-(β- *d* -ribofuranosyl)­purine (**6**) was reacted with the appropriate (substituted) alkyl or aryl amine in the presence of triethylamine (Et_3_N) in anhydrous EtOH to obtain the *N* ^6^-substituted adenosine derivatives **7a**–**r**. These compounds were subsequently phosphorylated to generate nucleoside monophosphates **8a**–**r** (Scheme 2A). Substituted alkylarylamines that were not commercially available were prepared by various methods. For the synthesis of nucleosides **7t**–**w**, the disubstituted amines **10a**–**e** were obtained by reaction of amines **9a**–**d** with 4-phenylbutyl bromide. Finally, intermediates **7s**–**w** were phosphorylated to generate nucleoside monophosphates **8s**–**w** (Scheme 2B).

**Scheme 2:**
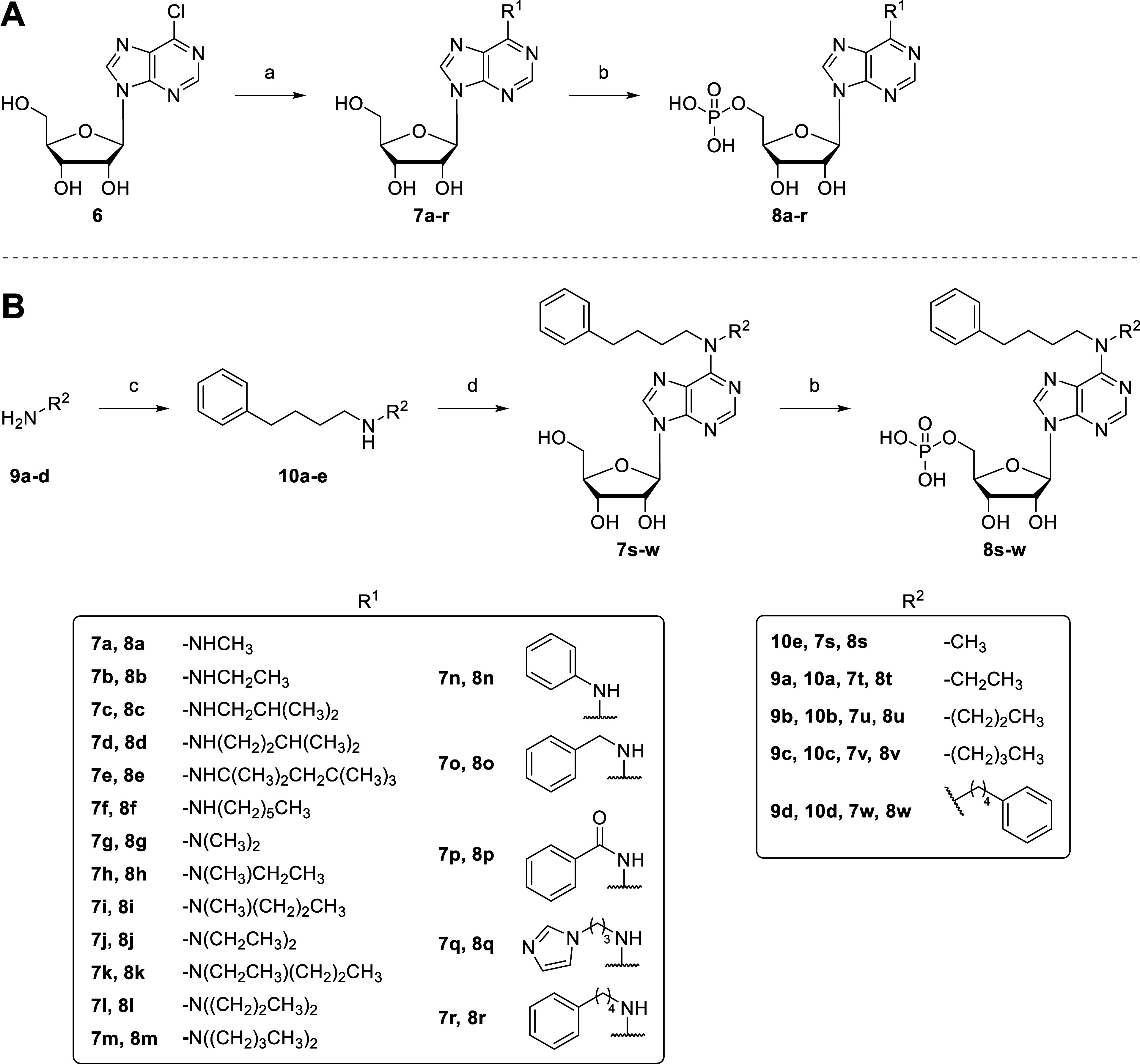
Synthesis of *N* ^6^-Substituted AMP Derivatives **8a–r** (A) and **8s–w** (B)^a^

a Reagents and conditions: (a) amine, Et_3_N, EtOH, reflux, 3–36 h, yield 34–100%; (b) two steps: (i) POCl_3_, PO­(OCH_3_)_3_, proton-sponge, 0 °C, 3–4 h; (ii) 0.5 M TEAC buffer (pH 7.4–7.6) or H_2_O, rt, 1 h, yield 3–74%; (c) 4-phenylbutyl bromide, MeOH, reflux, overnight, 76–92%; (d) 6-chloro-9-(β- *d* -ribofuranosyl)­purine, Et_3_N, EtOH, reflux, 3 h, yield 58–100%.

1-Amino-3-(3-methoxyphenyl)­propane (**13a**) and 1-amino-3-(4-methoxyphenyl)­propane (**13b**) were synthesized according to published procedures.^50,51^ The carboxylic acids **11a**–**b** were converted to primary amides via mixed anhydrides by reaction with isobutyl chloroformate and 4-methylmorpholine followed by quenching with NH_3_ in MeOH to generate amides **12a**–**b**, followed by reduction with LiAlH_4_ to yield the amines **13a**–**b** (A). *N*-(6-Aminohexyl)­benzamide (**19**) was obtained in a two-step procedure. Benzoic acid was activated with 1-hydroxybenzotriazole (HOBt) and DCC in THF and reacted with *N*-*tert*-butyloxycarbonyl (Boc)-protected 1,6-diaminohexane (**17**).^52^ Deprotection of the Boc group with TFA in DCM generated amine **19** (Scheme 3B). The prepared amines **13a**–**b**, **19**, and 3-phenylpropan-1-amine (**13c**) were used for the synthesis of adenosine derivatives **14a**–**d**. After acetonide protection of the 2′- and 3′-hydroxy groups, **15a**–**d** were phosphorylated and then deprotected to generate nucleoside monophosphates **16a**–**d**.

**Scheme 3:**
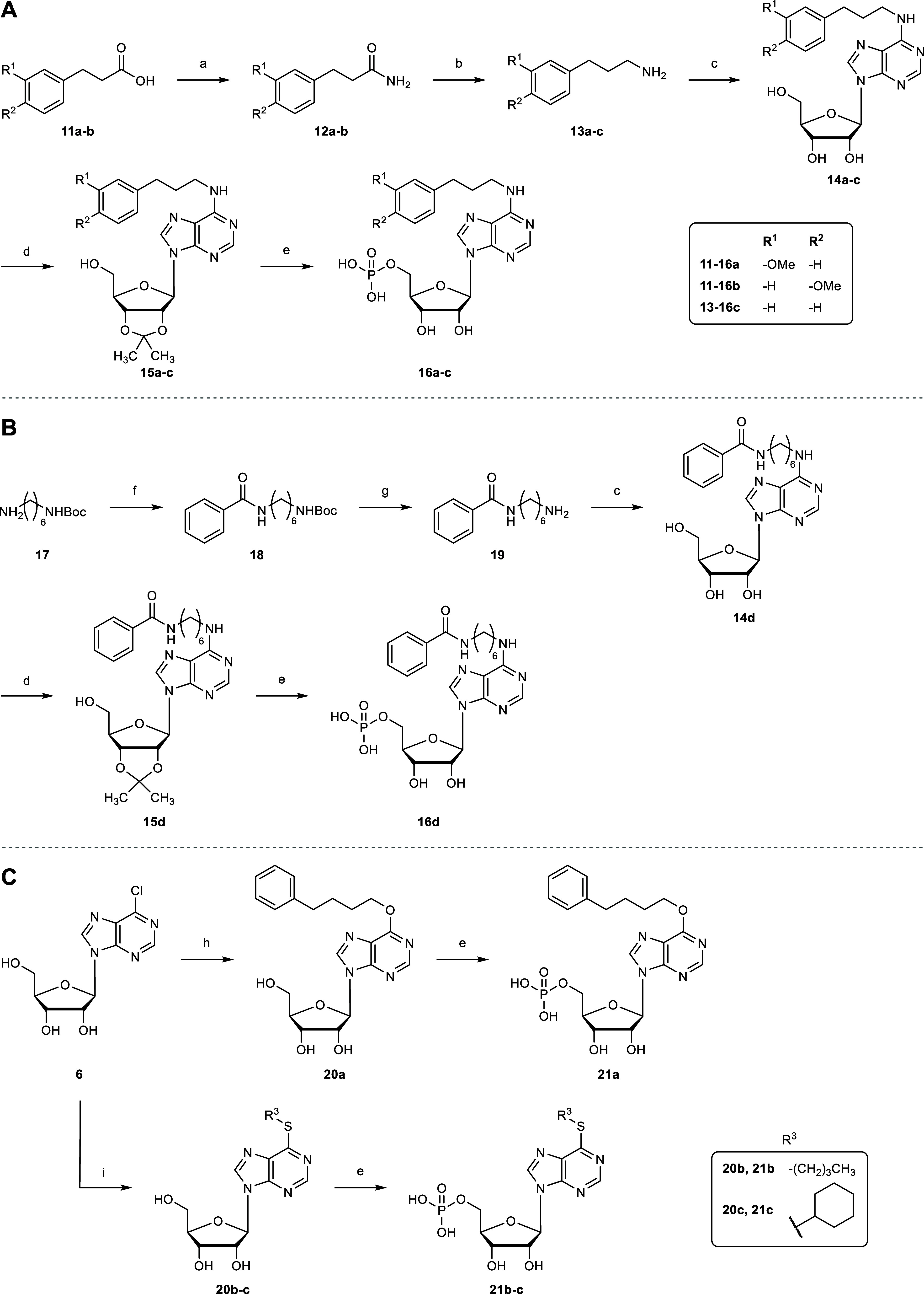
Synthesis of *N* ^6^-Substituted AMP Derivatives **16a–c** (A), **16d** (B), and **21a–c** (C)^a^

a Reagents and conditions: (a) three steps: (i) 4-methylmorpholine, isobutyl chloroformate, THF, 0 °C, 30 min; (ii) 7 M NH_3_ in MeOH, rt, 2 h; (iii) 10% K_2_CO_3_ (aq.), rt, 1 min; (b) two steps: (i) LiAlH_4_, dry THF, 0 °C to reflux, 30 min; (ii) H_2_O, 15% NaOH (aq.), rt, 0 min, yield 38–46%; (c) 6-chloro-9-(β- *d* -ribofuranosyl)­purine, Et_3_N, EtOH, reflux, 3 h, yield 17–47%; (d) 2,2-dimethoxypropane, H_2_SO_4_, acetone, rt, 15 min, 30–86%; (e) three steps: (i) POCl_3_, PO­(OCH_3_)_3_, proton-sponge, 0 °C, 4 h; (ii) 0.5 M TEAC buffer (pH 7.4–7.6), rt, 1 h; (iii) 7% TFA in DCM/H_2_O (9:1), rt, 2 h, yield 10–34%; (f) benzoic acid, HOBt, DCC, THF, rt, overnight, yield 91%; (g) 6% TFA in DCM, rt, 2 days, yield 100%; (h) sodium 4-phenylbutoxide, 4-phenylbutanol, reflux, 100 °C, 20 h, yield 31%; (i) butylthiol or cyclohexylthiol, NaOMe, reflux, overnight, yield 47–63%.

To investigate the effect of the amino group at the 6-position of adenosine, the bioisosteric analogs **21a**–**c** were synthesized, in which the nitrogen atom was replaced by oxygen or sulfur. The reaction of 6-chloro-9-(β- *d* -ribofuranosyl)­purine (**6**) with in situ generated sodium 4-phenylbutoxide by reaction of 4-phenylbutanol with elemental sodium yielded adenosine analog **20a**.^53^ Thio-derivatives were prepared by reaction of the corresponding thiols with **6** in the presence of sodium methoxide (NaOMe) in EtOH to generate adenosine analogs **20b**–**c**, which were subsequently phosphorylated to yield the corresponding nucleoside monophosphates **21a**–**c** (Scheme 3C).

### Synthesis of 8-Substituted AMP Derivatives

8-Chloroadenosine (**22**) was synthesized from adenosine (**4**) using benzoyl chloride and *meta*-chloroperoxybenzoic acid (mCPBA) (Scheme 4).^54^ For the preparation of further 8-substituted AMP derivatives, **4** was brominated using bromine buffered at pH 4.0 according to a published procedure to obtain 8-bromoadenosine (**23**).^55^ Substitution of **23** was performed by different strategies to obtain 8-substituted adenosine derivatives. For the synthesis of 8-amino-substituted adenosine derivatives, **23** was reacted with the appropriate amine in the presence of triethylamine in ethanol (EtOH) yielding **24a**–**d**.^55–57^ For the preparation of 8-methoxy-substituted adenosine (**26a**), compound **23** was reacted with sodium methoxide in methanol (MeOH).^58^ The synthesis of two further 8-alkoxy-substituted adenosine derivatives was achieved by suspending **23** in butanol, or cyclopentanol, respectively, in the presence of sodium hydroxide (NaOH) yielding **26b**–**c**. For the synthesis of 8-substituted adenosine derivatives, in which the residue was attached via a thioether linkage, four different methods were employed. In method I, sodium hydrosulfide was added to **23** to generate 8-thioadenosine (**29**), which was subsequently alkylated using the appropriate alkyl halide in EtOH/H_2_O (3:1) in the presence of NaOH to generate the adenosine derivatives **30a** and **30h**.^59–61^ Method II utilized the reaction of sodium ethanethiolate with **23** to generate adenosine derivative **30b**.^62^ According to method III,^63^ the appropriate thiol and sodium methoxide were added to **23** in EtOH generating adenosine derivatives **30c**–**g**, **30j**, and **30l**–**r**. Method IV employed **23** in a reaction with thiourea in EtOH followed by alkylation using the appropriate alkyl halide in the presence of NaOH to generate adenosine derivatives **30i** and **30k**.^55,59,60,64^ Finally, all 8-substituted adenosine derivatives, **22**, **24a**–**d**, **26a**–**c** and **30a**–**r**, were phosphorylated to obtain the nucleoside monophosphates **28**, **25a**–**d**, **27a**–**c** and **31a**–**r**. 8-Methoxy-AMP could not be obtained since the 8-methoxy group was hydrolyzed under the employed conditions generating the 8-hydroxy derivative **27a**.

**Scheme 4:**
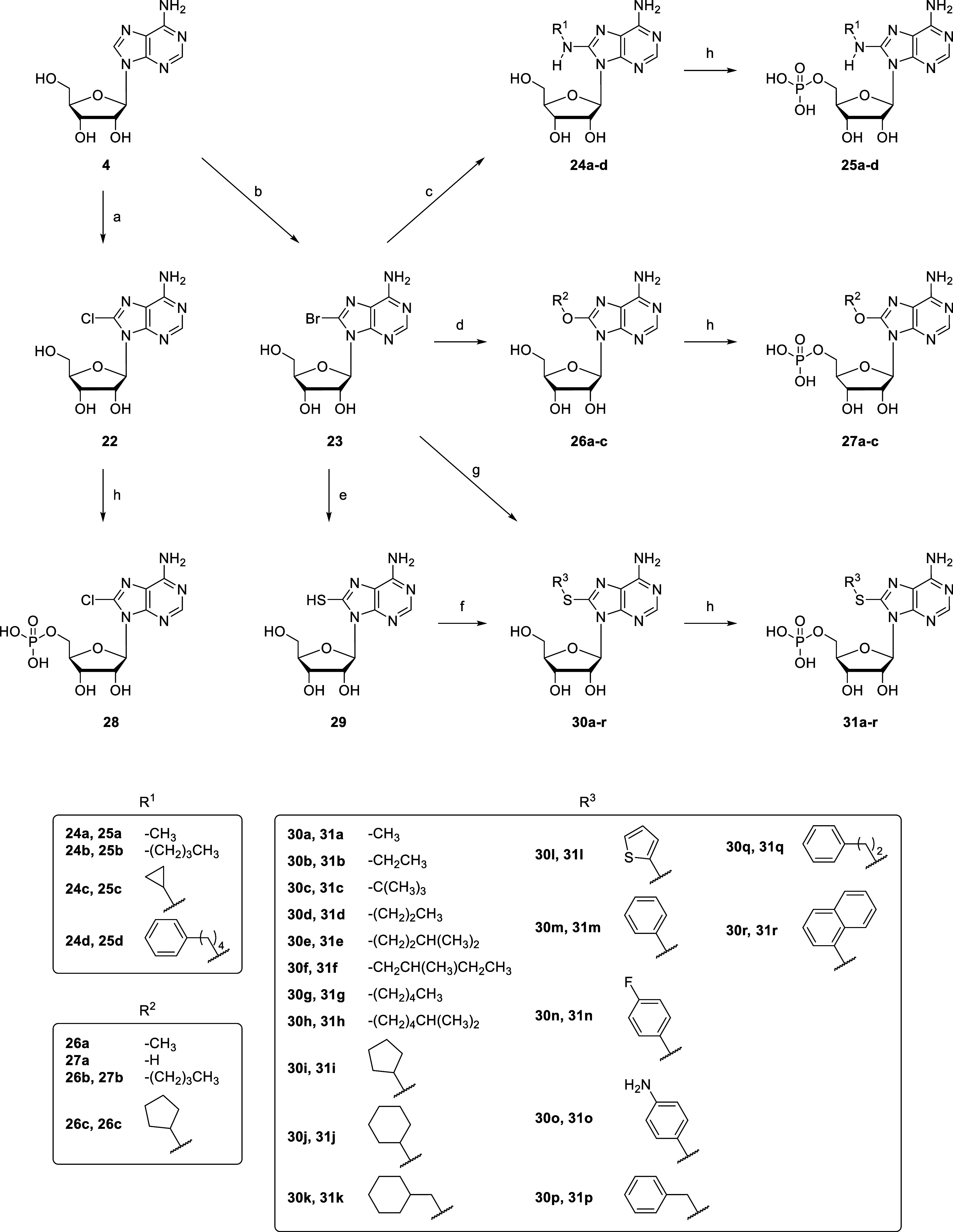
Synthesis of 8-Substituted AMP Derivatives **28**, **25a–d**, **27a–c** and **31a–r** ^a^

a Reagents and conditions: (a) BzCl, 3-chlorobenzene-1-carboperoxoic acid, DMF, rt, 30 min, yield 40%; (b) Br_2_, NaOAc buffer (pH 4.0), H_2_O, rt, overnight, yield 59%; (c) amines, Et_3_N, reflux, 18–48 h, yield 27–100%; (d) for **26a**: NaOMe, MeOH, reflux, overnight, yield 46%; for **26b**–**c**: butanol or cyclopentanol, NaOH, 50 °C, 2 h, yield 28–57%; (e) NaHS, DMF, 100 °C, 5 h, yield 59%; (f) for **30a** and **30h**, alkyl halide,H_2_O/EtOH (1:3), NaOH, reflux, 1–30 h, yield 30–80%; (g) for **30b**: sodium ethanethiolate, dry DMF, rt, overnight, yield 70%; for **30c**–**g**, **30j**, **30l**–**r**: thiol, NaOMe, EtOH, reflux, 3 h or overnight, yield 19–91%; for **30i** and **30k**: two steps: (i) thiourea, EtOH, reflux, overnight; (ii) alkyl halide, NaOH, H_2_O, reflux, 2 h, yield 17–57%; (h) two steps: (i) POCl_3_, PO­(OCH_3_)_3_, 1,8-bis­(dimethylamino)­naphthalene (proton-sponge), 0 °C, 3–4 h; (ii) 0.5 M TEAC buffer (pH 7.4–7.6) or H_2_O, rt, 1 h, yield 3–82%.

Saturated or unsaturated alkyl residues were introduced into the 8-position by C–C bond formation (Scheme 5). For the one-pot synthesis of 8-methyladenosine (**32a**),^65^ the 2′,3′,5′-hydroxyl groups of 8-bromoadenosine (**23**) were protected by reaction with hexamethyldisilazane (HMDS). Methylation was achieved using trimethylaluminum in the presence of triphenyl phosphine and PdCl_2_, which was followed by removal of the trimethylsilyl (TMS) protecting groups to generate **32a**. 8-Allyladenosine (**32b**) was prepared from **23**, and used for the synthesis of 8-propyladenosine (**32c**).^66^ The hydroxyl groups of **23** were again protected by HMDS, and alkylation was performed using allyl­(tributyl)­stannane, PPh_3_ and PdCl_2_ in *N*-methyl-2-pyrrolidone (NMP). The trimethylsilyl groups were then hydrolyzed to generate **32b**. Compound **32b** was subsequently hydrogenated with H_2_ at 45 psi on 10% Pd/C in THF/MeOH (1:1) to generate **32c**. Two 8-alkynyladenosine derivatives were synthesized from **23** according to a reported Sonogashira coupling reaction.^67^ To **23** in anhydrous DMF, Pd­(PPh_3_)_2_Cl_2_, CuI, triethylamine and 1-pentyne, or 1-hexyne, respectively, were added to generate **32d** and **32e**. Compound **32d** was further hydrogenated to generate 8-pentyladenosine (**32f**). 8-Phenyladenosine (**32g**) was synthesized from **23** according to a reported Suzuki reaction.^68^ To a solution of **23** in dioxane/H_2_O (2:1), phenylboronic acid, Pd­(PPh_3_)_2_Cl_2_ and K_2_CO_3_ were added to obtain **32g**. Finally, **32a** and **32c**–**g** were phosphorylated to yield the nucleoside monophosphates **33a**–**f** (Scheme 5A).

**Scheme 5:**
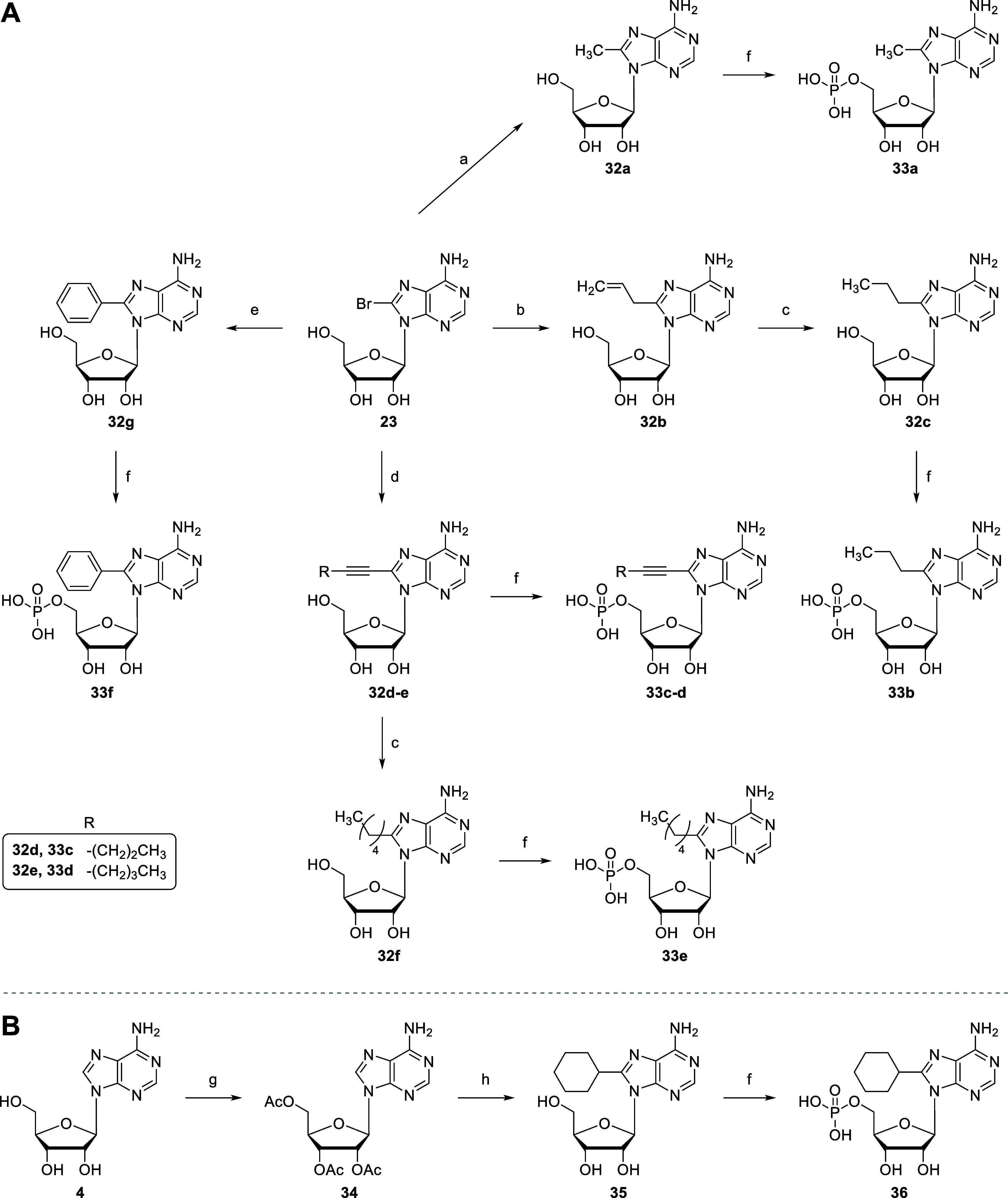
Synthesis of 8-Alkyl-, Alkenyl-, Alkynyl- and Aryl-Substituted AMP Derivatives **33a–f** (A) and **36** (B)^a^

a Reagents and conditions: (a) three steps: (i) HMDS, (NH_4_)_2_SO_4_, dry dioxane, reflux, 3 h; (ii) PPh_3_, PdCl_2_, Al­(CH_3_)_3_, THF, argon, reflux, 3 h; (iii) NH_4_Cl, MeOH, reflux, overnight, yield 31%; (b) three steps: (i) HMDS, (NH_4_)_2_SO_4_, dry dioxane, reflux, 3 h; (ii) PPh_3_, PdCl_2_, allyltributyltin, NMP, argon, reflux, 3 h; (iii) NH_4_Cl, MeOH, reflux, overnight, yield 63%; (c) THF/MeOH (1:1), 10% Pd/C, H_2_, 45 psi, rt, 2 h, yield 83–95%; (d) 1-pentyne or 1-hexyne, Pd­(PPh_3_)_2_Cl_2_, CuI, Et_3_N, dry DMF, argon, 95 °C, 6 h, yield 16–17%; (e) phenylboronic acid, dioxane/H_2_O (2:1), Pd­(PPh_3_)_2_Cl_2_, K_2_CO_3_, argon, 90 °C, 2 h, yield 95%; (f) two steps: (i) POCl_3_, PO­(OCH_3_)_3_, 1,8-bis­(dimethylamino)­naphthalene (proton-sponge), 0 °C, 3 h; (ii) satd. aq. NH_4_HCO_3_ or H_2_O, rt, 1 h, yield 3–56%; (g) DMAP, Et_3_N, MeCN, acetic anhydride, rt, 30 min, yield 75%; (h) two steps: (i) cyclohexane, di-*tert*-butyl peroxide, 140 °C, 24 h; (ii) 7 N NH_3_ in MeOH, rt, overnight, yield 49%.

8-Cyclohexyladenosine (**35**) was synthesized from adenosine (**4**) by a reported metal-free C–C bond formation procedure with minor modifications (Scheme 5B).^69^ The hydroxyl groups of **4** were acetyl-protected using acetic anhydride and DMAP in acetonitrile to yield **34**. To a solution of **34** in cyclohexane, di-*tert*-butyl peroxide was added, and the mixture was stirred at 140 °C to introduce the cyclohexyl moiety at the 8-position presumably via a cyclohexyl free-radical intermediate. The 2′,3′,5′-*O*-acetyl groups were removed by treatment with NH_3_ in MeOH to yield **35**, followed by phosphorylation to generate nucleoside monophosphate **36**.

### Synthesis of *N* ^6^,8-Disubstituted AMP Derivatives

The standard CD39 inhibitors ARL67156 (**1a**) and 8-butylthio-AMP (**1b**) have substitutions at the 8- or *N* ^6^-position.^26,28^ Thus, we decided to combine both and additionally prepare disubstituted AMP derivatives. Since *N* ^6^-(4-phenylbutyl)-AMP (**8r**) was found to show relatively high CD39 inhibitory potency, its *N* ^6^-substituent was combined with the promising 8-substituents of 8-butylthio-AMP (**1b**) and 8-methylamino-AMP (**25a**). Bromination of the 8-position of *N* ^6^-(4-phenylbutyl) was not successful in contrast to the preparation of 8-bromoadenosine (**23**) as described above. We tried several reaction conditions all of which did not yield the desired product. Therefore, we decided to introduce the 8-substituent before *N* ^6^-substitution (Scheme 6).

**Scheme 6:**
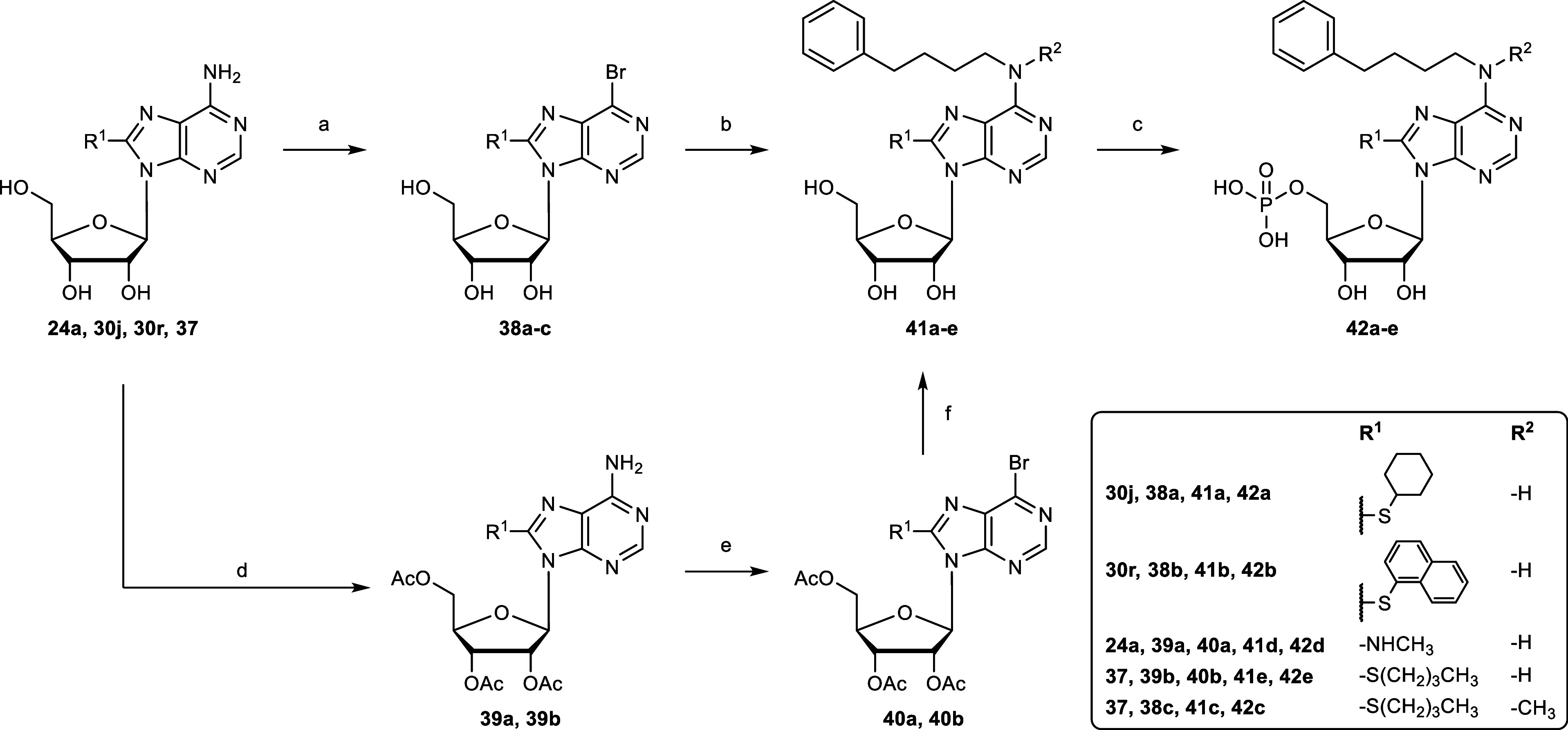
Synthesis of *N* ^6^,8-Disubstituted AMP Derivatives **42a–e** ^a^

a Reagents and conditions: (a) TMSBr, *tert*-butyl nitrite, CH_2_Br_2_, argon, 0 °C to rt, overnight, yield 30–91%; (b) 4-phenylbutylamine or methyl­(4-phenylbutyl)­amine, Et_3_N, EtOH, reflux, 3 h, yield 41–90%; (c) two steps: (i) POCl_3_, PO­(OCH_3_)_3_, proton-sponge, 0 °C, 3–4 h; (ii) 0.5 M TEAC buffer (pH 7.4–7.6) or satd. aq. NH_4_HCO_3_, rt, 1 h, yield 5–28%; (d) DMAP, *N*,*N*-dimethylethylamine, acetic anhydride, MeCN, rt, 15 min, yield 71–76%; (e) SbBr_3_, benzyltriethylammonium bromide, NaNO_2_, dichloroacetic acid, AcOH, CH_2_Br_2_, rt, argon, overnight, yield 47–51%; (f) two steps: (i) 4-phenylbutylamine, Et_3_N, EtOH, reflux, 3 h; (ii) 20% NaOMe, MeOH, rt, 48 h, yield 57–88%.

The hydroxyl groups of the adenosine derivatives 8-methylamino adenosine (**24a**) and 8-butylthio adenosine (**37**) were protected by acetylation, and the 6-amino group was subsequently brominated through a bromo-dediazotization process using NaNO_2_, SbBr_3_, benzyltriethylammonium bromide, dichloroacetic acid and acetic acid in dibromomethane.^70^ The resulting bromopurine derivatives **40a** and **40b** were substituted with 4-phenylbutylamine, followed by basic deprotection of the acetyl groups with NaOMe to generate the disubstituted adenosine derivatives **41d** and **41e**. Final phosphorylation of **41d** and **41e** yielded nucleoside monophosphates **42d** and **42e**. The adenosine derivatives **30j**, **30r**, and **37** underwent bromo-dediazotization using trimethylsilyl bromide (TMSBr) and *tert*-butyl nitrite in dibromomethane to obtain the bromopurine derivatives **38a**–**c**,^71^ followed by nucleophilic aromatic substitution and phosphorylation, omitting the use of riboside-protecting groups to generate nucleoside monophosphates **42a**–**c**.

Further *N* ^6^,8-disubstituted AMP derivatives **48a**–**f**, **49a**–**c**, and **50** were prepared starting from *N* ^6^-substituted adenosine derivatives (Scheme 7). The starting materials were prepared by introducing methyl- (**7a**), ethyl- (**7b**), dimethyl- (**7g**), diethyl- (**7j**) and 4-phenylbutylamine (**7r**) by nucleophilic substitution of 6-chloropurine riboside with the appropriate alkyl amines at the *N* ^6^ of adenosine (Scheme 2). The 8-position was then brominated using bromine buffered at pH 4.0, as described above, to generate **43a**–**d**. Nucleosides **43a**–**d** were subsequently substituted with various amines in the presence of triethylamine to generate the 8-alkylamino-substituted adenosine derivatives **44a**–**f** bearing an additional *N* ^6^-substituent, while **43a** and **43d** were reacted with 1-butanethiol, or 1-thionaphthol, in the presence of NaOMe in EtOH, to generate the *N* ^6^-substituted 8-alkyl and 8-arylthioadenosine derivatives **45a**–**c**. A phenyl residue was introduced at the 8-position of *N* ^6^-(4-phenylbutyl)-AMP (**8r**) by a reported procedure with modifications.^72^ To compound **7r** in anhydrous DMF was added iodobenzene, Pd­(OAc)_2_, CuI and Cs_2_CO_3_, and the mixture was stirred in an autoclave under argon at 120 °C to generate **46**. Finally, the adenosine derivatives **44a**–**f**, **45a**–**c** and **46** were phosphorylated to generate the nucleoside monophosphates **47a**–**f**, **48a**–**c** and **49**.

**Scheme 7:**
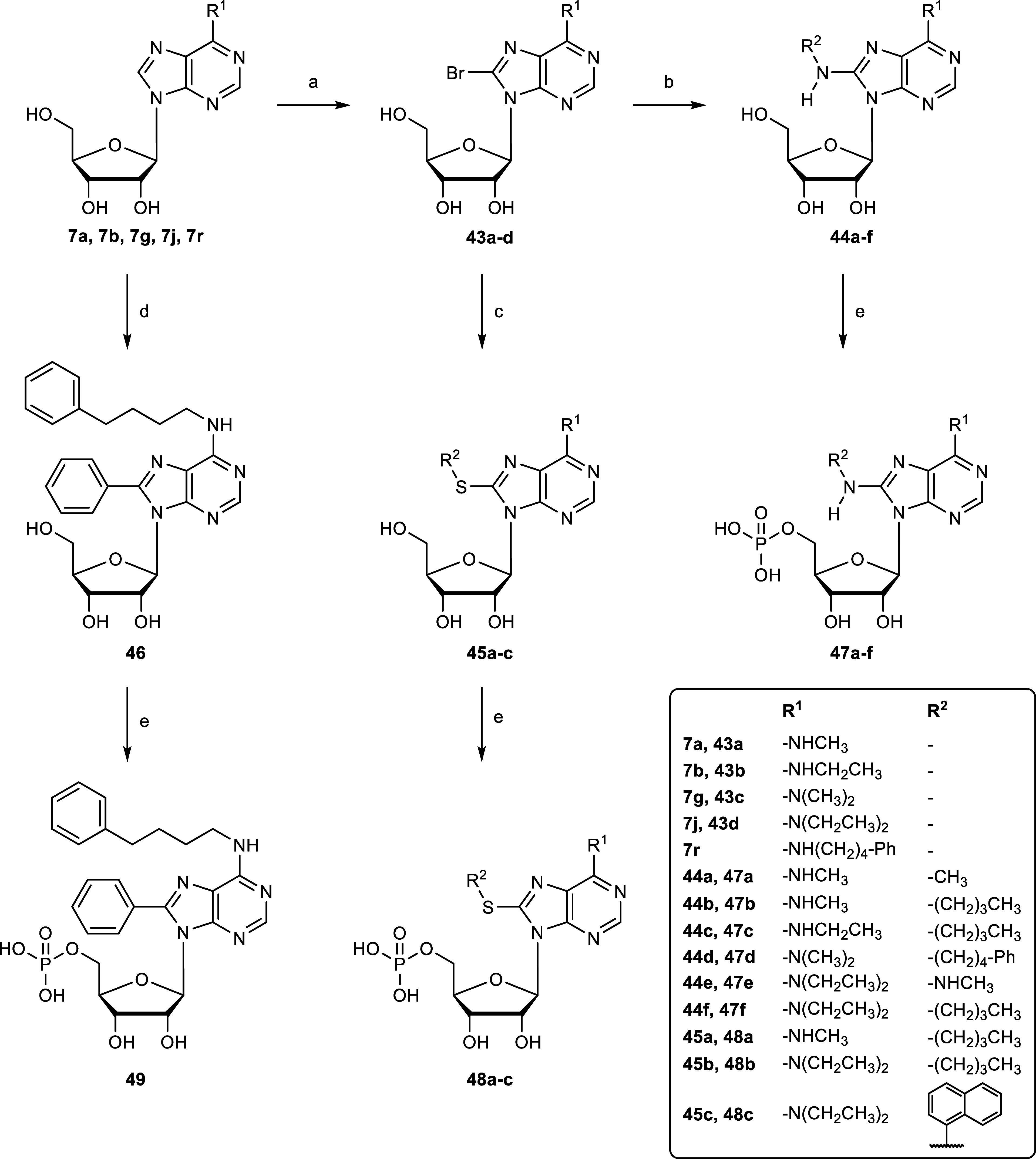
Synthesis of *N* ^6^,8-Disubstituted AMP Derivatives **47a–f**, **48a–c** and **49** ^a^

a Reagents and conditions: (a) Br_2_, NaOAc buffer (pH 4.0), rt, overnight, yield 14–25%; (b) aryl- or alkylamine, Et_3_N, reflux, 18–36 h, yield 36–100%; (c) 1-butanethiol or 1-thionaphthol, NaOMe, EtOH, reflux, 4–18 h, yield 12–88%; (d) iodobenzene, Pd­(OAc)_2_, CuI, Cs_2_CO_3_, dry DMF, argon, 120 °C, overnight, yield 28%; (e) two steps: (i) POCl_3_, PO­(OCH_3_)_3_, proton-sponge, 0 °C, 3–4 h; (ii) 0.5 M TEAC buffer (pH 7.4–7.6) or satd. aq. NH_4_HCO_3_, rt, 1 h, yield 5–56%.

### Synthesis of 2,*N* ^6^-Disubstituted AMP Derivatives **53**, **56**, **58**, and **59**

In addition to 8-mono- and *N* ^6^,8-disubstituted AMP derivatives, the *N* ^6^-(4-phenylbutyl)-substituent was also combined with chloro-, bromo-, and amino-substitution at the 2-position of the purine core (Scheme 8). 2,6-Dichloropurine was added to molten tetraacetylribose (**50**) at 110 °C in the presence of triflic acid.^73^ The mixture was stirred at 110 °C and 0.09 MPa to continuously remove the formed acetic acid generating 2′,3′,5′-tri-*O*-acetyl-2,6-dichloropurine riboside (**51**). Subsequently, **51** was reacted with 4-phenylbutylamine in the presence of triethylamine in EtOH to yield adenosine derivative **52**, which was phosphorylated to generate the disubstituted AMP derivative **53** (Scheme 8A). Starting from commercial 2-amino-6-chloro-9-(β- *d* -ribofuranosyl)­purine (**54**), the corresponding AMP derivative **56** was prepared as described above (Scheme 8B). Additionally, **54** was brominated through the previously described bromo-dediazotization reaction using TMSBr and *tert*-butyl nitrite in dibromomethane,^71^ followed by phosphorylation to generate the nucleoside monophosphate **58**. Compound **58** was reacted with 4-phenylbutylamine in the presence of triethylamine in EtOH to obtain the disubstituted AMP derivative **59** (Scheme 8B).

**Scheme 8:**
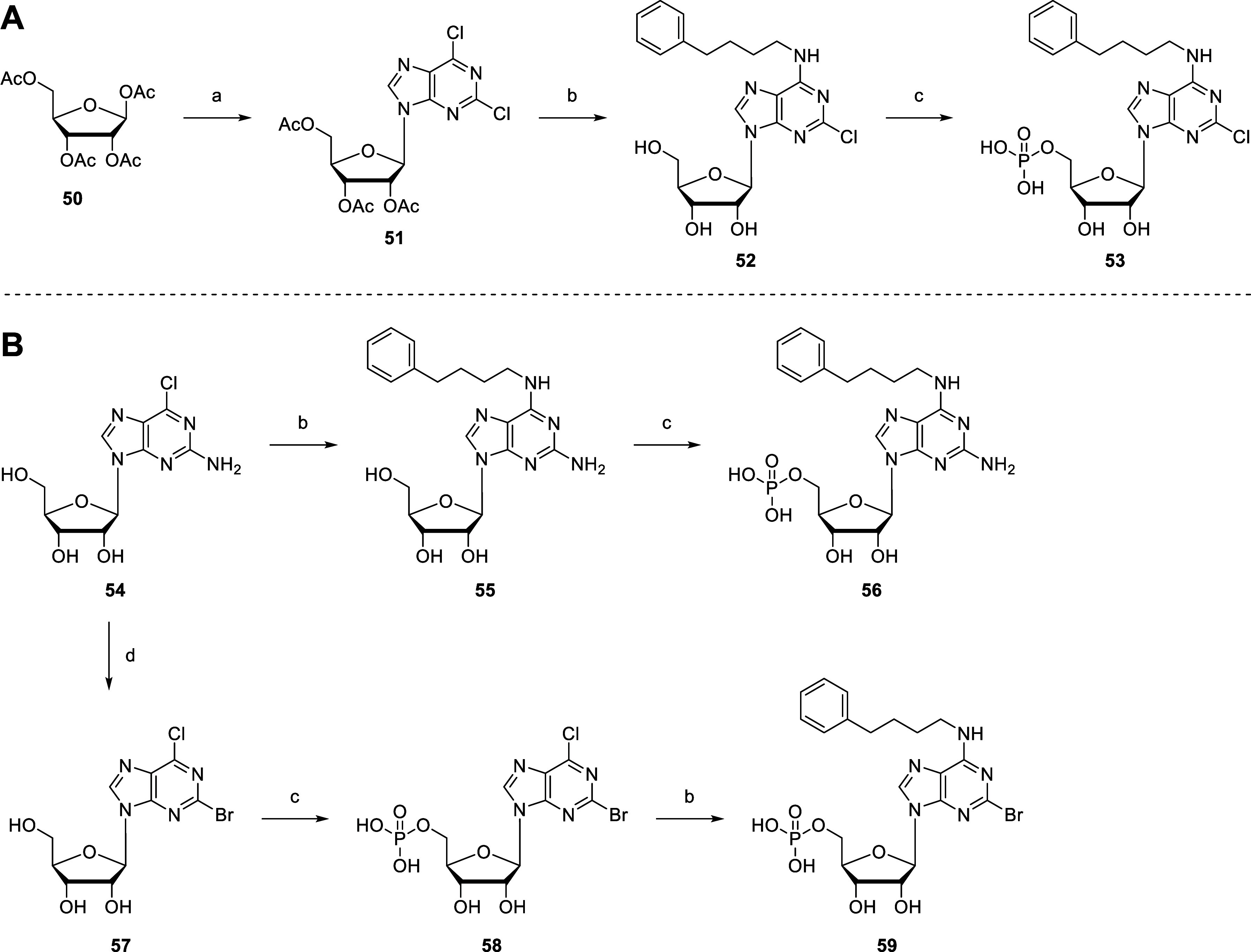
Synthesis of 2,*N* ^6^-Disubstituted AMP Derivatives **53** (A) and **56**, **58**, and **59** (B)^a^

a Reagents and conditions: (a) 2,6-dichloropurine, triflic acid, 110 °C, 0.09 MPa, 1 h, yield 52%; (b) 4-phenylbutylamine, Et_3_N, EtOH, reflux, 3 h, yield 38–74%; (c) two steps: (i) POCl_3_, PO­(OCH_3_)_3_, proton-sponge, 0 °C, 3 h; (ii) 0.5 M TEAC buffer (pH 7.4–7.6), rt, 1 h, yield 21–33%; (d) TMSBr, *tert*-butyl nitrite, CH_2_Br_2_, rt, overnight, yield 56%.

### Synthesis of Etheno-AMP Derivatives

In analogy to ethenoadenosine, a fluorescent tool compound in which *N*1 and *N* ^6^ of the adenine moiety are cyclized via an etheno bridge, etheno-AMP derivatives without 8-substituent (**61a**) and with 8-butylthio substitution (**61b**) were prepared (Scheme 9). The nucleosidic intermediates **60a** and **60b** were synthesized by reacting adenosine (**4**) and 8-butylthioadenosine (**37**) with chloroacetaldehyde in the presence of sodium acetate.^74^ The resulting tricyclic compounds **60a** and **60b** were subsequently phosphorylated to yield nucleoside monophosphates **61a** and **61b**.

**Scheme 9:**
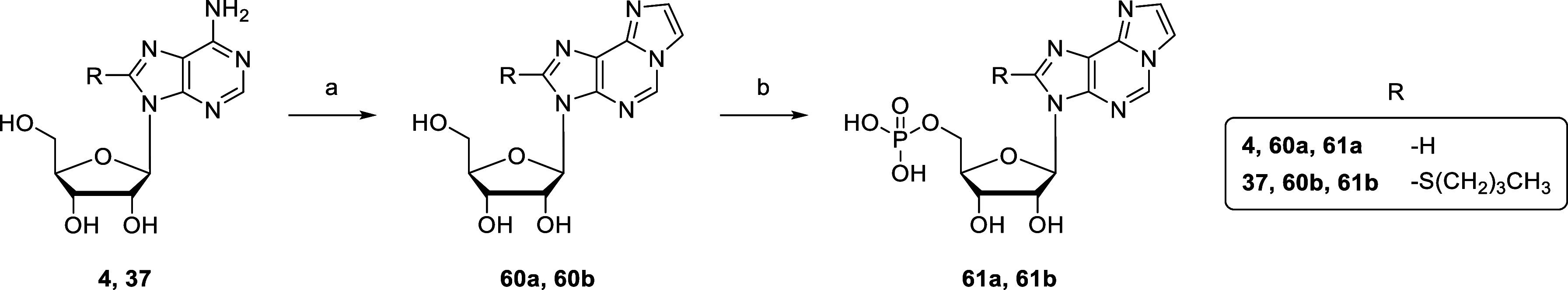
Synthesis of 1,*N* ^6^-Etheno-AMP (61a) and 8-Butylthio-1,*N* ^6^-etheno-AMP (61b)^a^

a Reagents and conditions: (a) 2 M aq. chloroacetaldehyde, CH_3_COONa, 50 °C, 5 h, yield 92–100%; (b) two steps: (i) POCl_3_, PO­(OCH_3_)_3_, proton sponge, 0 °C, 3 h; (ii) satd. aq. NH_4_HCO_3_, rt, 1 h, yield 15–30%.

### Monophosphorylation

For most nucleosides, monophosphorylation was performed according to the Kovács and Ötvos modification of the Yoshikawa procedure with minor adjustments.^46,47,59^ Briefly, nucleosides were dissolved in trimethyl phosphate and reacted with phosphoryl trichloride in the presence of 1,8-bis­(dimethylamino)­naphthalene at 0 °C under argon to yield the reactive 5′-dichlorophosphate intermediates. Hydrolysis with triethylammonium hydrogen carbonate (TEAC) buffer, water, or preferably with saturated aq. NH_4_HCO_3_ solution yielded the desired nucleoside monophosphates. The crude mixtures were extracted with methyl *tert*-butyl ether (MTBE) to remove trimethyl phosphate and proton-sponge. The products were further purified by preparative HPLC on a reversed-phase C18 column yielding the desired pure nucleotides.

For adenosine derivatives **14a**–**d** containing larger *N* ^6^-substituents, mixtures of different monophosphates were obtained by this method. Interestingly, in the LC/ESI-MS analysis, only one peak with the desired mass was detected, but ^31^P NMR clearly showed multiple signals for phosphorus atoms. This phenomenon was also observed for the phosphorylation of 2-methylthioadenosine (**2e**), but not when smaller *N* ^6^-substituents like ethyl or methyl were present. Unfortunately, the different monophosphates eluted simultaneously from the HPLC column and were therefore not separable. In order to obtain the desired pure 5′-*O*-monophosphates, the hydroxy groups at the 2′,3′-positions of adenosine derivatives **14a**–**d** and **2e** were protected using 2,2-dimethoxypropane in acetone in the presence of H_2_SO_4_.^75^ The 2′,3′-acetonide-protected adenosine derivatives **15a**–**e** were phosphorylated according to the procedure described above but without the addition of proton-sponge, which prolonged the reaction time.^47^ The acidic conditions in the absence of the strong base led to the partial deprotection of the 2′,3′-acetonide groups, as observed by LC/ESI-MS analysis. After purification by HPLC, the protected AMP derivatives were treated with 7% trifluoroacetic acid (TFA) in DCM/H_2_O (9:1) to complete the deprotection. The nucleotides were precipitated by adding cold diethyl ether followed by a second purification step by HPLC yielding the desired monophosphates **16a**–**d** and **3d**.^55^

### CD39 Inhibitory Potency

The synthesized AMP derivatives substituted in the 1-, 2-, *N* ^6^-, or 8-position, or combinations thereof, were investigated for inhibition of CD39 to study SARs. Preparations of human umbilical cord, which natively expresses high levels of CD39,^76^ or recombinant human CD39, expressed in COS-7 cells, were used as sources of CD39. Further characterization of potent compounds to determine concentration-dependent inhibition, and the type of inhibition, was performed using recombinant human CD39 enzyme expressed in COS-7 cells.^26,77^ Results were very similar in both enzyme preparations, as previously reported.^9^ Inhibitory potency was determined using the malachite green assay, which measures phosphate, and the natural substrate ATP, or a previously established capillary electrophoresis-based method, employing a fluorophore-labeled ATP derivative as a substrate.^22^ The *K*_i_ values of a number of inhibitors were validated in both assay systems, and results were found to be similar in both assays, in agreement with previous reports.^22,44,78^ The inhibitory potencies are collected in Tables 1, 2, 3 and 4.

**Table 1:**
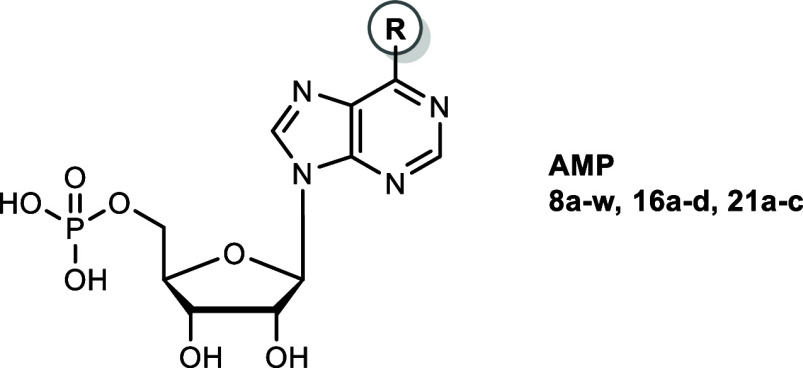

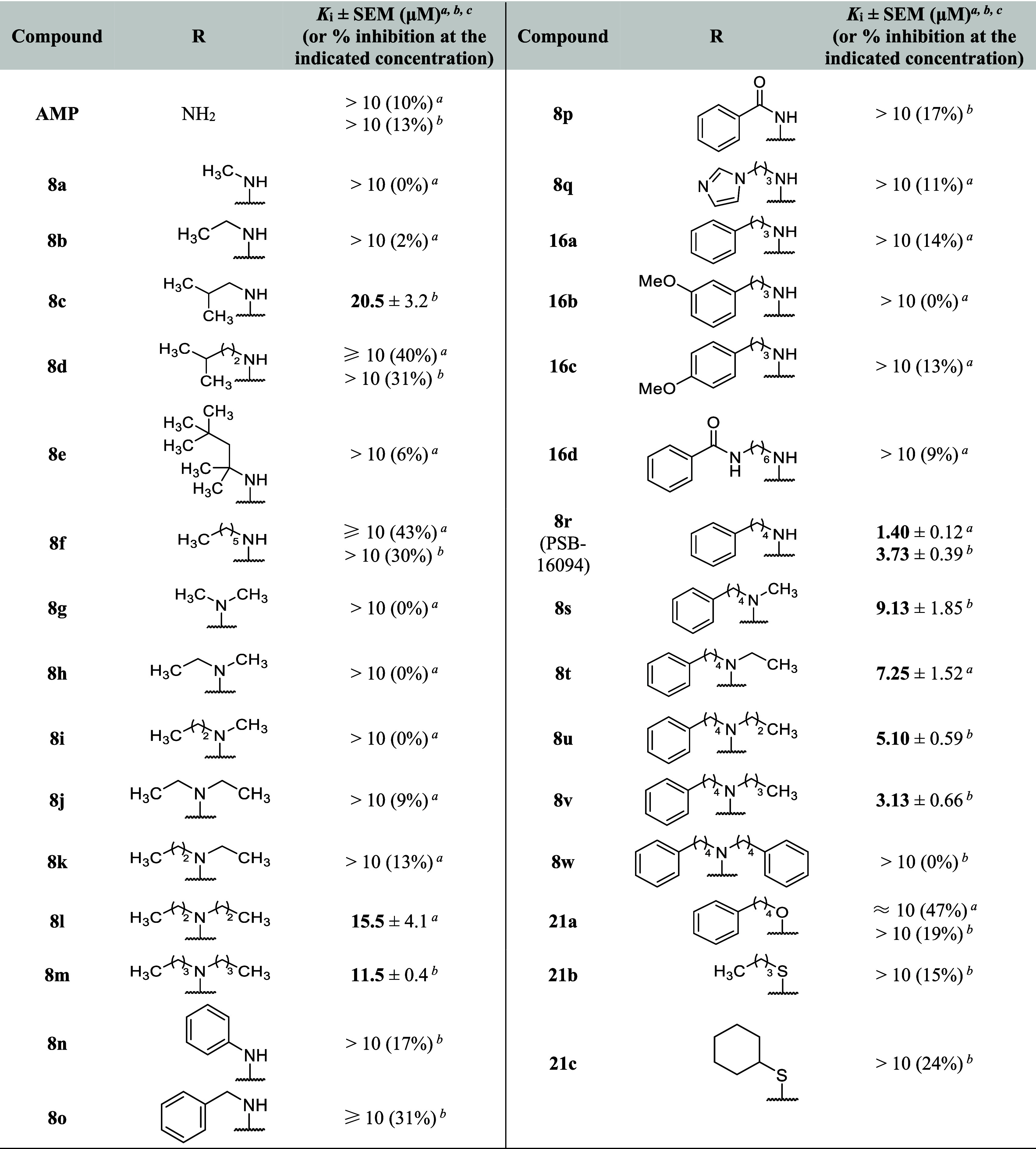
Potency of *N* ^6^-Substituted AMP Derivatives and Analogs as Inhibitors of Human CD39

a Fluorescence-based capillary electrophoresis assay: screening at 10 μM was performed and concentration–inhibition curves were determined for potent inhibitors using the fluorescent substrate PSB-170621A (0.5 μM) and human membrane-bound CD39 (*n* = 3).

b Malachite green assay: screening at 10 μM was performed and concentration–inhibition curves were determined for potent inhibitors using the natural substrate ATP (50 μM) and human membrane-bound CD39 (*n* = 3).

c *K*_i_ values of inhibitors for which concentration-dependent inhibition was determined are shown in bold.

**Table 2:**
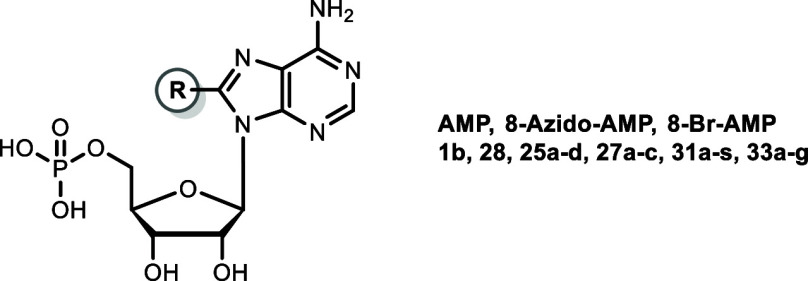

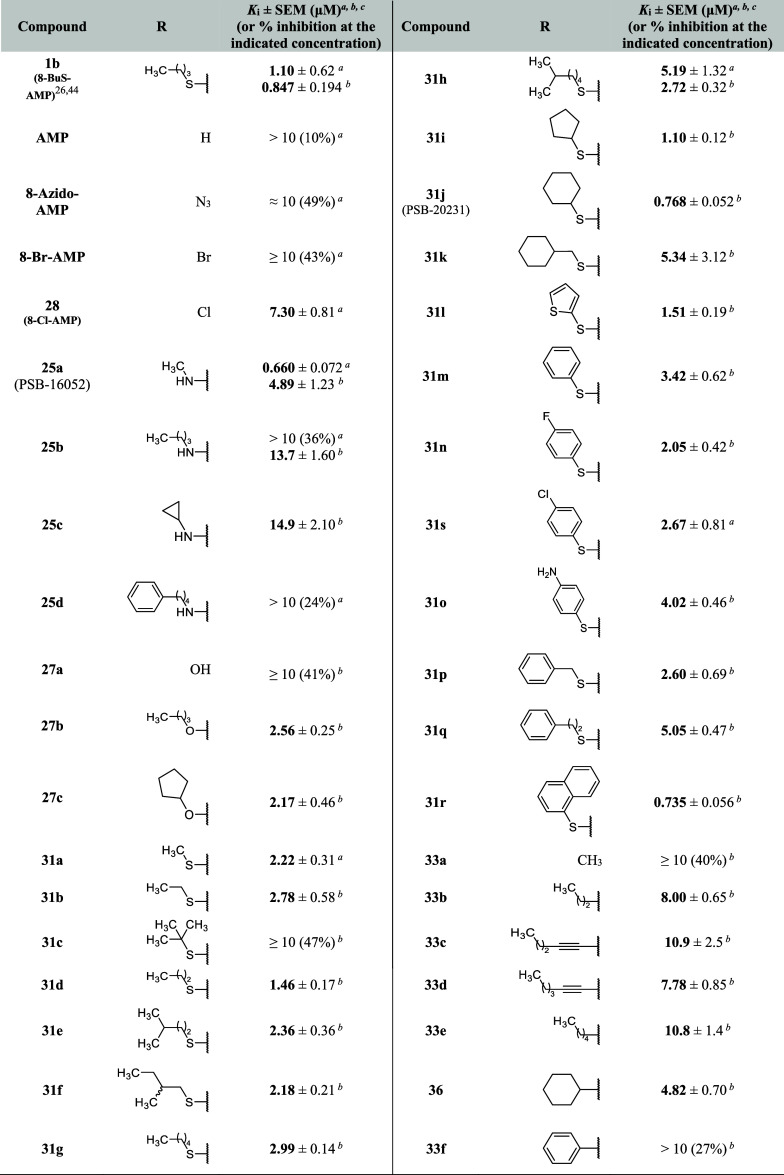
Potency of 8-Substituted AMP Derivatives as Inhibitors of Human CD39

a Fluorescence-based capillary electrophoresis assay: screening at 10 μM was performed and concentration–inhibition curves were determined for potent inhibitors using the fluorescent substrate PSB-170621A (0.5 μM) and human membrane-bound CD39 (*n* = 3).

b Malachite green assay: screening at 10 μM was performed and concentration–inhibition curves were determined for potent inhibitors using the natural substrate ATP (50 μM) and human membrane-bound CD39 (*n* = 3).

c *K*_*i*_ values of inhibitors for which concentration-dependent inhibition was determined are shown in bold.

**Table 3:**
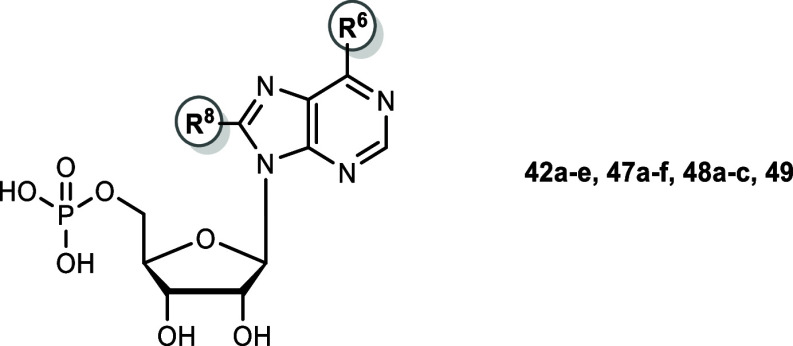

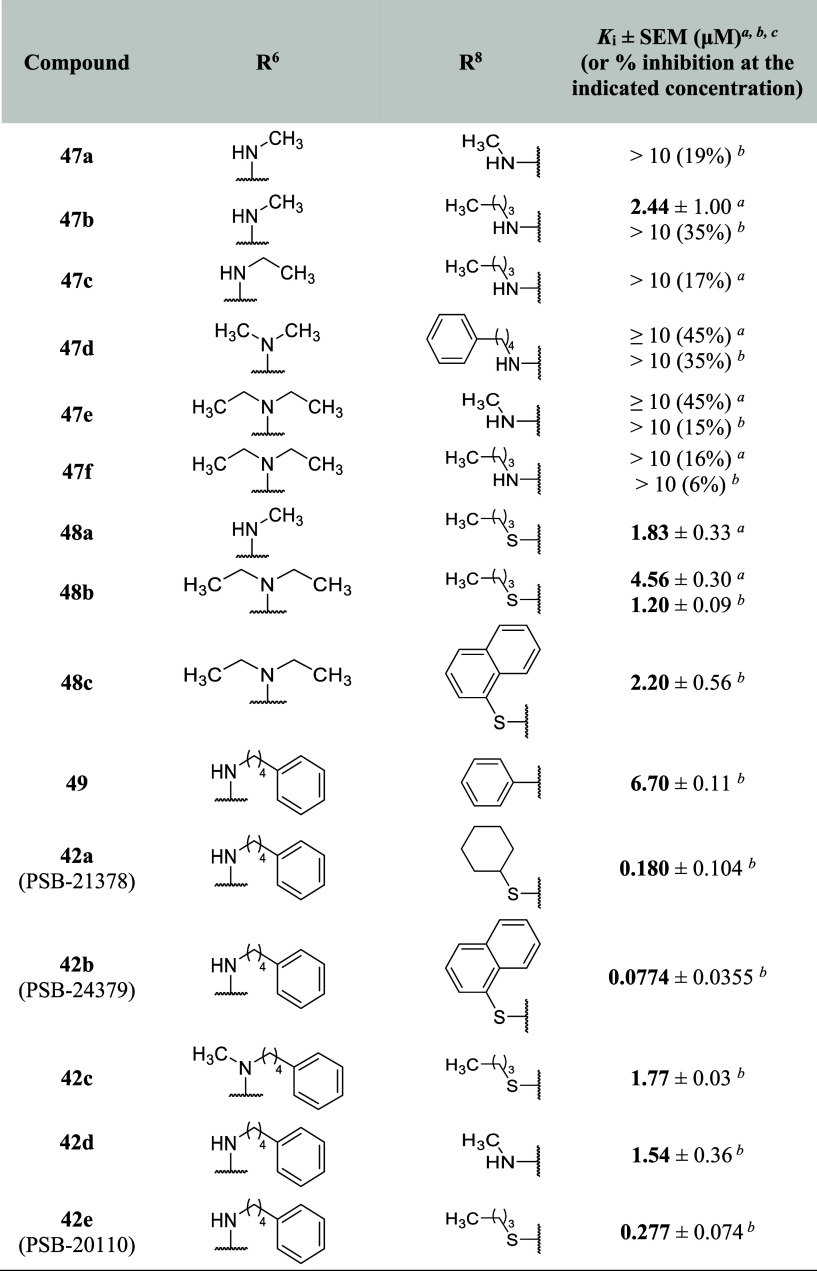
Potency of *N* ^6^,8-Disubstituted AMP Derivatives as Inhibitors of Human CD39

a Fluorescence-based capillary electrophoresis assay: screening at 10 μM was performed and concentration–inhibition curves were determined for potent inhibitors using the fluorescent substrate PSB-170621A (0.5 μM) and human membrane-bound CD39 (*n* = 3).

b Malachite green assay: screening at 10 μM was performed and concentration–inhibition curves were determined for potent inhibitors using the natural substrate ATP (50 μM) and human membrane-bound CD39 (*n* = 3).

c *K*_i_ values of inhibitors for which concentration-dependent inhibition was determined are shown in bold.

**Table 4:**
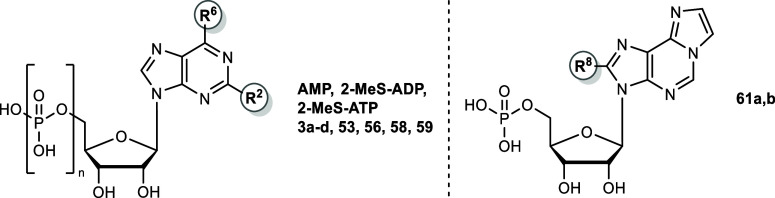

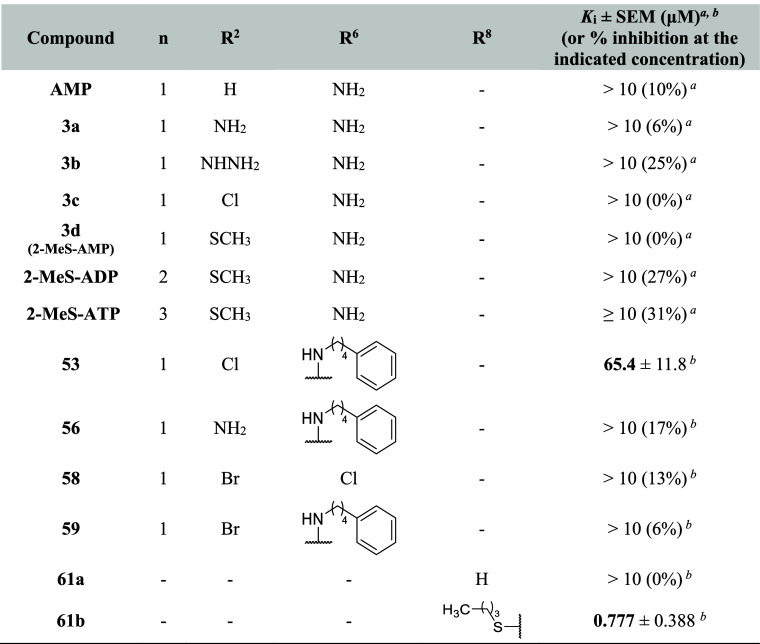
Potency of 2-Substituted, 2,*N* ^6^-Disubstituted and Tricyclic AMP Derivatives as Inhibitors of Human CD39

a Fluorescence-based capillary electrophoresis assay: screening at 10 μM was performed and concentration–inhibition curves were determined for potent inhibitors using the fluorescent substrate PSB-170621A (0.5 μM) and human membrane-bound CD39 (*n* = 3).

b Malachite green assay: screening at 10 μM was performed and concentration–inhibition curves were determined for potent inhibitors using the natural substrate ATP (50 μM) and human membrane-bound CD39 (*n* = 3).

c *K*_i_ values of inhibitors for which concentration-dependent inhibition was determined are shown in bold.

Representative concentration–response curves for selected inhibitors are shown in Figure 4. The data confirm dose-dependent inhibition with well-defined sigmoidal profiles, enabling reliable estimation of IC_50_ and calculation of *K*_i_ values.

**Figure 4:**
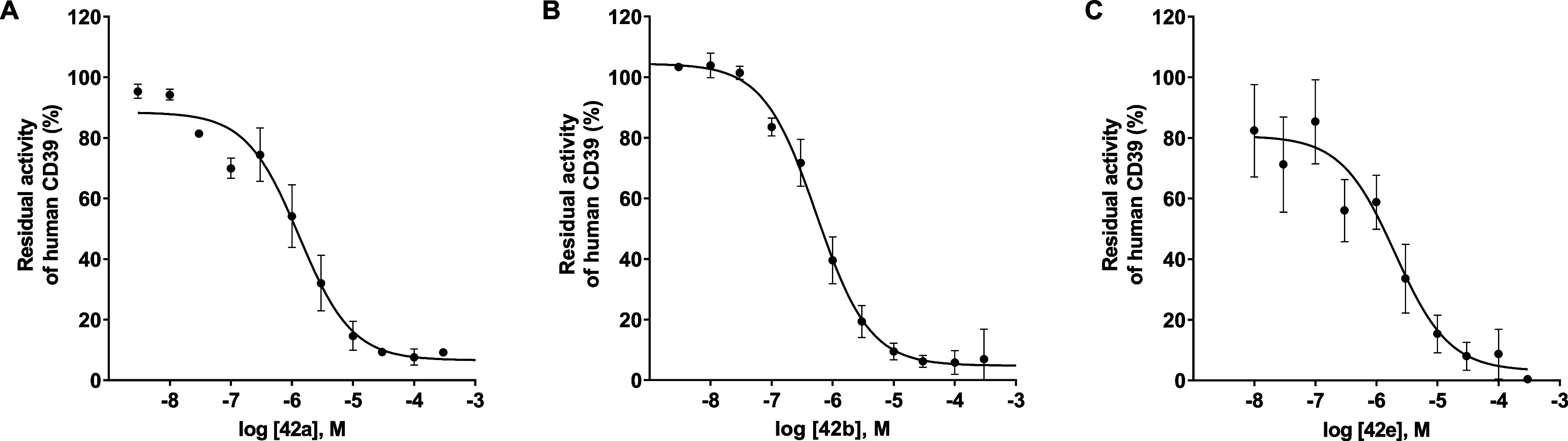
Concentration-dependent inhibition of ATP hydrolysis by compounds **42a** (A), **42b** (B), and **42e** (C) using recombinant human membrane-bound CD39 expressed in COS-7 cells and the natural substrate ATP (50 μM), measured by the malachite green assay; **42a**, IC_50_ 1.31 μM, *K*_i_ 0.180 μM (*n* = 6); **42b**, IC_50_ 0.563 μM, *K*_i_ 0.0774 μM (*n* = 10); **42e**, IC_50_ 2.02 μM, *K*_i_ 0.277 μM (*n* = 6).

### Structure–Activity Relationships

8-Butylthio-AMP-derived CD39 inhibitors were explored through systematic modification of the 1-, 2-, 6-, and 8-positions. The parent AMP (19% inhibition at 10 μM) and *N* ^6^-substituted derivatives bearing short alkyl chains (**8a**–**m**) were inactive or only weakly potent as CD39 inhibitors, with *K*_i_ values of above 10 μM. This includes the *N* ^6^-diethylamino substituent (**8j**, 9% at 10 μM), which had been found to be crucial for the inhibitory potency of the standard CD39 inhibitor ARL-67156 (*K*_i_ 0.973 μM) as compared to the *N* ^6^-unsubstituted β,γ-dibromomethylene-ATP.^43^ Increasing length of the *N* ^6^-substituent resulted in increased potency: a pentyl chain (**8f**) conferred moderate inhibition (43% at 10 μM), whereas branched alkyl residues (e.g., **8e**) were poorly tolerated (6% at 10 μM). An aryl group directly connected to *N* ^6^ (phenylamine, **8n**) or via a linker of up to 3 methylene groups (**16a**) resulted in inactive compounds (**8n**–**q**, **16a**–**c**). However, extension to a four-carbon linker yielded the most potent compound in the series, *N* ^6^-(phenylbutyl)-AMP (**8r**, *K*_i_ 1.40 μM). These findings suggest a distal hydrophobic pocket that is only accessed by *N* ^6^-substituents of sufficient length. Further substitution at the *N* ^6^-nitrogen atom in addition to the 4-phenylbutyl moiety generally reduced inhibitory potency, but increase in alkyl chain length of the second substituent on the amino group improved potency: butyl (**8v**, *K*_i_ 3.13 μM) > propyl (**8u**, *K*_i_ 5.10 μM) > ethyl (**8t**, *K*_i_ 7.25 μM) > methyl (**8v**, *K*_i_ 9.13 μM). Disubstitution with two 4-phenylbutyl groups (**8w**) as well as prolongation of the alkyl chain and insertion of an amide group (**16d**) abolished inhibitory potency. Surprisingly, the oxygen analog **21a** of the most potent compound **8r** was inactive, suggesting that the hydrogen bonding capability of *N* ^6^–H is favorable, albeit not crucial. In agreement with the findings on the oxygen analog, the sulfur analogs **21b** and **21c** were found inactive.

Exploration of the 8-position demonstrated that potency is highly dependent on both substituent size and heteroatom attached to C8. Polar substituents and several (pseudo)­halogens at C8 (8-OH, 8-Br, 8-azido) led to inactive compounds, whereas the 8-Cl derivative (**28**) showed moderate inhibition (*K*_i_ 7.30 μM). In general, small thioethers (**31a**–**h**) allowed for compounds with *K*_i_ values in the low micromolar range (2–3 μM), with the notable exception of a *tert*-butylthioether residue (**31c**, 47% inhibition at 10 μM). Truncation (propyl, **31d**, *K*_i_ 1.46 μM) or elongation (pentyl, **31g**, *K*_i_ 2.99 μM) of the 8-butylthio residue reduced inhibitory potency, indicating that the butyl chain of 8-butylthio-AMP (**1b**, *K*_i_ 0.847 μM) is near-optimal. Cyclic and rigid substituents performed comparably or better than linear alkyl chains: 8-cyclopentylthio (**31i**, *K*_i_ 1.10 μM) and its homologue 8-cyclohexylthio (**31j**, *K*_i_ 0.768 μM) matched the potency of **1b**. Docking of 8-cyclohexylthio-*N* ^6^-(4-phenylbutyl)­amino-AMP (**42a**) and **1b** into a homology model of human CD39 proposed a similar binding conformation of the 8-thio substituents (see Supporting Information Figure S1).

The potency of **31j** was decreased by 5-fold by the addition of a methylene group (8-cyclohexylmethylthio-AMP, **31k**, *K*_i_ 5.34 μM). Oxygen (**27b**) and carbon (**33e**) isosteres of the butylthio group led to less potent inhibitors (**27b**, 3-fold loss; **11e**, 13-fold loss), and the nitrogen isostere (**25b**) resulted in an even larger loss (16-fold). When comparing 8-cyclopentylthio-AMP (**31i**, *K*_i_ 1.10 μM) and its oxygen analog **27c** (*K*_i_ 2.17 μM) the difference was smaller. However, the shorter 8-methylamino-AMP (**25a**, *K*_i_ 0.660 μM) was similarly potent as its sulfur analog **31a** (*K*_i_ 2.22 μM).

The thioether derivatives 8-(2-sulfanylphenylthio)-AMP (**31l**, *K*_i_ 1.51 μM) and 8-(phenylthio)-AMP (**31m**, *K*_i_ 3.42 μM) bearing aromatic rings were moderately active, with small electronegative *para*-substituents (F (**31n**), Cl (**31s**)) giving slight improvements and *para*-amino substitution (**31o**) reducing potency. Elongation of a phenylthio substituent with methylene (**31p**) or ethylene linkers (**31q**) reduced activity. Annelation to give 8-(1-naphthylthio)-AMP (**31r**) increased potency by 4-fold (*K*_i_ 0.735 μM), making this residue one of the best C8-substituents besides 8-butylamino (**1b**) and 8-cyclohexylthio substitution (**31j**). The 8-alkyl, -alkenyl, -alkynyl and -aryl derivatives (**33a**–**g**) were found to be only moderately potent CD39 inhibitors (*K*_i_ ≈ 4–11 μM).

The introduction of amino (**3a**), chloro (**3d**), or methylthio (**3c**) substitution at the 2-position of AMP did not lead to inhibition of CD39 activity, while substitution with a 2-hydrazino group (**3b**) led to weak inhibition of CD39. Even in combination with the favorable *N* ^6^-(4-phenylbutyl) substituent, a 9-fold loss in inhibitory potency was observed for the 2-chloro-substituted derivative **53** (*K*_i_ 65.4 μM) relative to the 2-unsubstituted analog **8r**. Similarly, 2-bromo (**59**) and 2-amino substitution (**56**) was also detrimental. Di- and triphosphate analogs of 2-methylthio-AMP (**3d**) appeared to be slightly more potent (27% and 31% vs 0% inhibition at 10 μM). Introduction of an etheno bridge in the tricyclic AMP analog **61a** did not result in inhibition of CD39 (0% at 10 μM), while its combination with the beneficial 8-butylthio substituent induced significant inhibitory potency (**61b**, *K*_i_ 0.777 μM).

Next, combinations of promising 6- and 8-substituents were investigated to evaluate potential additive effects. The inhibitory potency of 6- or 8-monosubstituted derivatives with minimal activity (*K*_i_ > 10 μM) was rescued upon introduction of a second, favorable substituent, as exemplified by 8-butylthio-*N* ^6^-methylamino-AMP (**48a**, *K*_i_ 1.83 μM) and 8-phenyl-*N* ^6^-(4-phenylbutyl)­amino-AMP (**49**, *K*_i_ 6.70 μM). Incorporation of the *N* ^6^-(4-phenylbutyl) moiety consistently enhanced inhibitory potency relative to the corresponding C8-monosubstituted compounds, most prominently in derivatives bearing 8-butylthio (**42e**, *K*_i_ 0.277 μM), 8-cyclohexylthio (**42a**, *K*_i_ 0.180 μM), or 8-(1-naphthylthio) (**42b**, *K*_i_ 0.0774 μM) groups. Introduction of an additional methyl group at the *N* ^6^-position of **42b** reduced the inhibitory potency by 7-fold, again indicating that a hydrogen bond donor at this position is beneficial. Overall, C8-substituents exert a stronger influence on inhibitory potency than *N* ^6^-modifications, as reflected by the larger improvements observed for C8- relative to *N* ^6^-monosubstituted AMP derivatives. Selected structure–activity relationships are compiled in Figure 5. The most potent CD39 inhibitor discovered in this study is 8-(1-naphthylthio)-*N* ^6^-(4-phenylbutyl)-AMP (**42b**, PSB-24379), with a *K*_i_ value of 77.4 nM. At present, this is the most potent small molecule that competitively blocks CD39.

**Figure 5:**
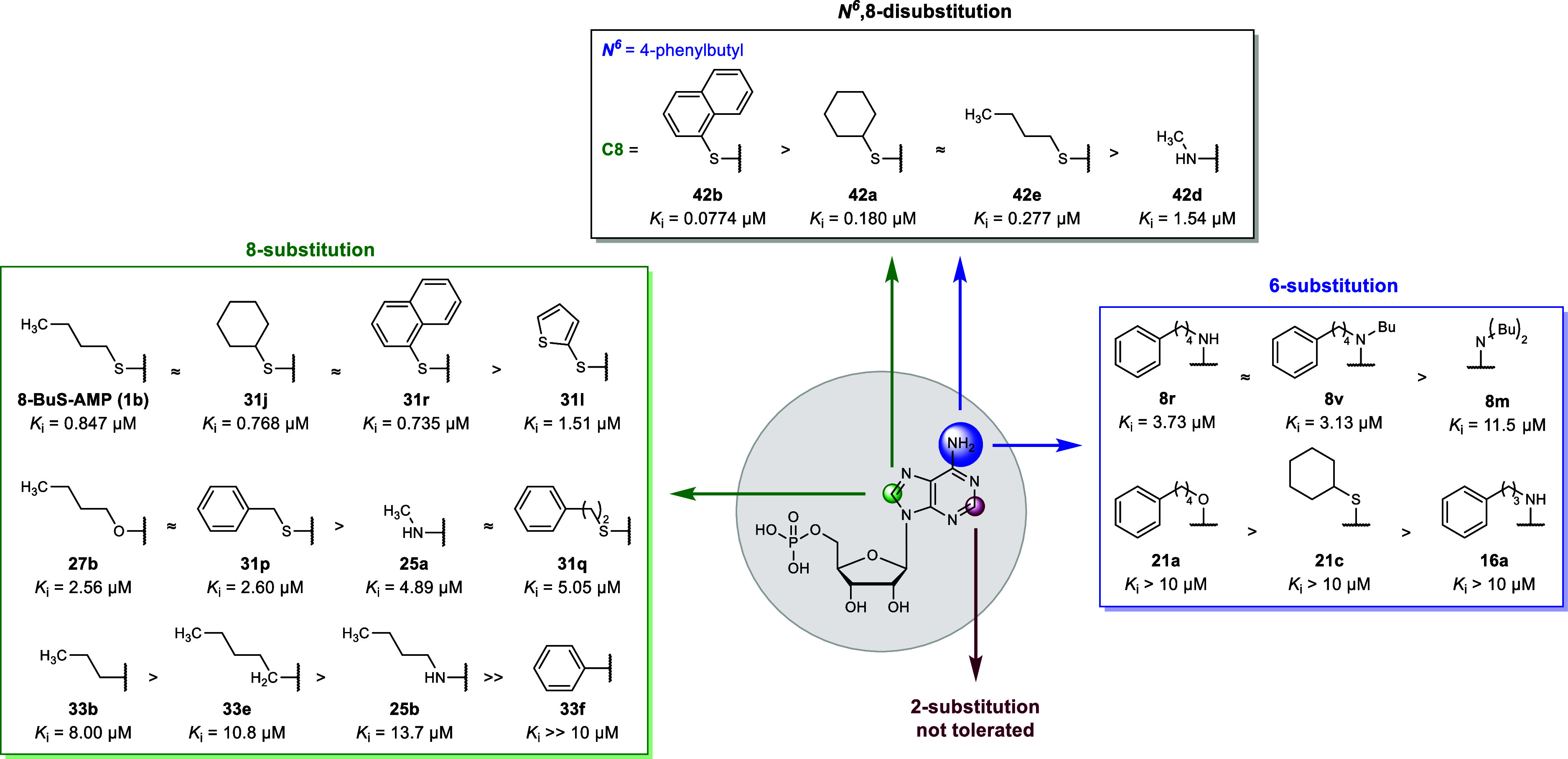
Selected structure–activity relationships of AMP derivatives and analogs as CD39 inhibitors.

### Computational Studies

The most potent CD39 inhibitor, 8-naphthylthio-*N* ^6^-(4-phenylbutyl)-AMP (**42b**), was selected for docking studies to analyze its interactions within the substrate binding site. Molecular docking was performed using Schrödinger Maestro employing a previously reported homology model of human CD39, based on the rat CD39 structure (PDB 3ZX3),^22,44^ which included the bound Ca^2+^ ion and coordinating water molecules (see Experimental section). Docking analysis suggested a tightly anchored 8-naphthylthio-*N* ^6^-(4-phenylbutyl)-AMP (**42b**) within the binding pocket (Figure 6). The purine core is likely held in place by π–π interactions with Phe365 and Tyr408, forming a sandwich-type complex. The 8-naphthylthio residue may contribute an additional stacking interaction with His59. The *N* ^6^–H atom is predicted to engage with Tyr412 forming a hydrogen bond. In the docked pose, the sulfur atom of the 8-naphthylthio substituent is positioned in close proximity to Arg85. The phosphate moiety likely establishes an extensive hydrogen-bonding network with Ser58, His59, Arg85, and the Gly216 backbone while being stabilized by complexation with the Ca^2+^ ion, which in turn is anchored in the catalytic site through coordination with Asp54, Glu174, and water molecules.

**Figure 6:**
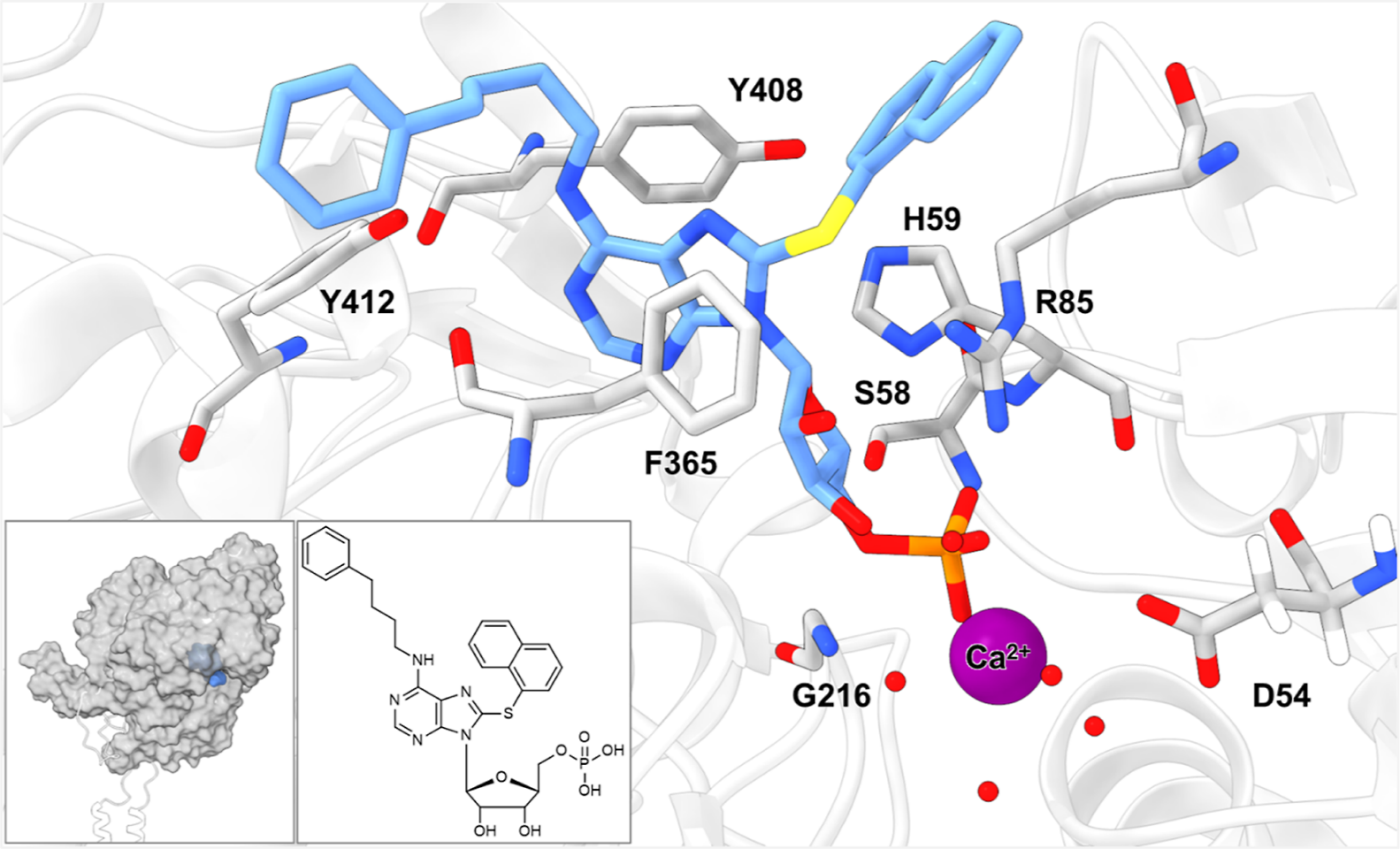
Docked pose of compound **42b** (blue) within the substrate binding pocket of a human CD39 homology model (based on PDB 3ZX3).^21,22,44^ Key interacting residues are shown as gray sticks, water as red spheres, and the coordinated Ca^2+^ ion is depicted as a purple sphere. Bottom left corner: complete protein (gray) with binding pocket (blue) superimposed with its AlphaFold3 model (UniProt P49961, white contour) using the mmaker command in ChimeraX to visualize the membrane-binding domains. According to the model, the ligand is stabilized by π–π contacts with Phe365, Tyr408, and His59, hydrogen bonding of *N* ^6^–H with Tyr412, and an extended phosphate interaction network involving Ser58, His59, Arg85, Gly216, and the Ca^2+^ ion. The figure was created using UCSF ChimeraX.^79^

### Broad Characterization of Selected Potent CD39 Inhibitors

#### Determination of Inhibition Type

The inhibition type of the potent CD39 inhibitor 8-(1-naphthylthio)-*N* ^6^-(4-phenylbutyl)-AMP (**42b**) was determined using a range of substrate and inhibitor (Figure 7). The inhibitor shows a competitive inhibition type as indicated by an increase in the apparent substrate affinity K_*m*_^app^, while the maximum velocity of the enzyme reaction stays virtually constant. This is in agreement with the previously determined inhibition type of the lead structure 8-butylthio-AMP (**1b**), which was also found to be exclusively competitive.^44^ Our results show that the additional *N* ^6^-substitution does not prevent the compound from binding to the substrate-binding site of CD39.

**Figure 7:**
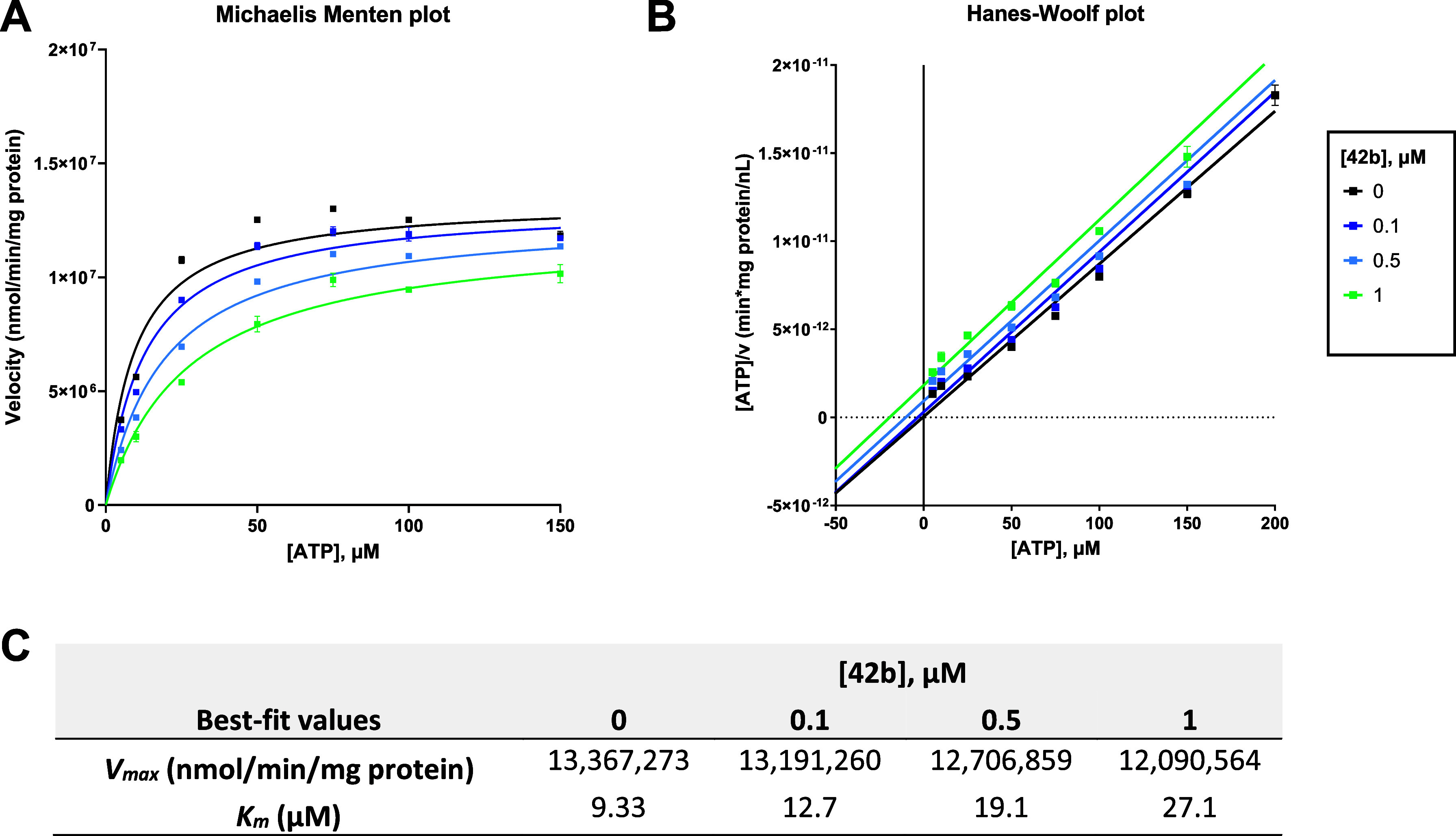
Inhibition type determination of **42b**. (A) Michaelis Menten plot of human CD39 inhibition by 0.1, 0.5, 1.0, and 2.0 μM **42b** in the presence of 5, 10, 25, 50, 75, 100, 150, and 200 μM ATP as substrate determined using the malachite green assay. (B) Hanes–Woolf plot. The experiments were performed three times, each in duplicate. (C) The observed increase in K_*m*_^app^ with no significant effect on the *V*_max_ value indicates a competitive inhibition type.

#### Truncated Soluble CD39

Inhibitory potencies of selected AMP derivatives were additionally determined at recombinantly prepared, purified human CD39 consisting of the enzymatically active soluble part of the enzyme, lacking the transmembrane domains^44^ (Supporting Information Table S2 and Figure S2). Such a CD39 preparation is frequently used for drug screening.^80,81^ Data were compared to those determined using full-length, membrane-bound CD39. We had previously observed that certain allosteric CD39 inhibitors, e.g. ceritinib,^9^ only inhibit the membrane-bound enzyme, but not the soluble preparation (unpublished results). Some of the competitive CD39 inhibitors investigated in this study showed higher potency (lower *K*_i_ value) at the artificial, soluble CD39 as compared to the membrane-bound enzyme, while for others similar *K*_i_ values were determined at both enzyme preparations. Notably, **42a** displayed significantly higher potency at soluble CD39 (*K*_i_ 11.2 nM vs 237 nM; 16-fold difference). Compound **42b** displayed a *K*_i_ value of 23.1 nM at soluble CD39 compared to 77.4 nM at the membrane-bound enzyme (3.4-fold difference), **42e** showed a *K*_i_ value of 58.9 nM at the soluble enzyme vs 277 nM at the membrane-bound CD39 (4.7-fold difference). On the other hand, 8-butylthio-AMP (**1b**), lacking an *N* ^6^-substituent, exhibited nearly identical *K*_i_ values at both enzyme preparations.^44^ These and other results indicate that the artificial soluble CD39 is not ideal for the characterization of CD39 inhibitors, in particular for allosteric inhibitors. But it should be sufficiently predictive and thus well suitable for the initial screening of competitive CD39 inhibitors.

#### Selectivity

Next, we selected prominent inhibitors of the present study, **8r**, **25a**, **31j**, **31r**, **42a**, **42b**, **42e**, for testing at a broad range of human ectonucleotidases, namely NTPDase2, −3 and −8, NPP1, −3, −4 and −5, CD38 and CD73, as depicted in comparison with the lead structure 8-butylthio-AMP (**1b**) in Figure 8 (for *K*_i_ values see Supporting Information Table S1). Further compounds were additionally tested at human CD73 since many of the compounds showed significant dual CD39/CD73 inhibition (Figure 8 and Supporting Information Table S3).^82^ The experiments were performed by established procedures (see Experimental section).^48,77,83–85^ The 8-monosubstituted 8-cyclohexylthio-AMP (**31j**) was selective for CD39 versus NTPDase2, −3, −8, NPP1, −3, −4, −5 and CD38, but also inhibited CD73 and can therefore be characterized as a dual CD39/CD73 inhibitor, similar to **1b**. The 8-naphthylthio-AMP derivative **31r** displayed additional inhibition of NTPDase2 and, in particular, of NTPDase3. 8-Methylamino-AMP (**25a**) was the only investigated derivative that selectively inhibited CD39, although its potency was moderate. It represents the first selective CD39 inhibitor, which will be useful as a pharmacological tool compound to study CD39 inhibition. The findings for 8-monosubstituted AMP derivatives are in agreement with the previously determined selectivity of 8-butylthio-AMP (**1b**), showing that sterically demanding 8-substituents allow for additional inhibition of CD73.^44^ The 6-monosubstituted *N* ^6^-(4-phenylbutyl)-AMP (**8r**) was selective versus NTPDase3, −8, NPP3, −5 and CD38 but showed relatively high inhibition of NTPDase2 and CD73, in addition to CD39. The *N* ^6^,8-disubstituted compounds **42a**, **42b** and **42e** were selective versus NTPDase8, NPP1, −3, −4, −5 and CD38 but inhibited NTPDase2, −3 and CD73, although with lower potency. This supports the notion that the *N* ^6^-(4-phenylbutyl) substituent favors additional inhibition of NTPDase2 (and −3).

**Figure 8:**
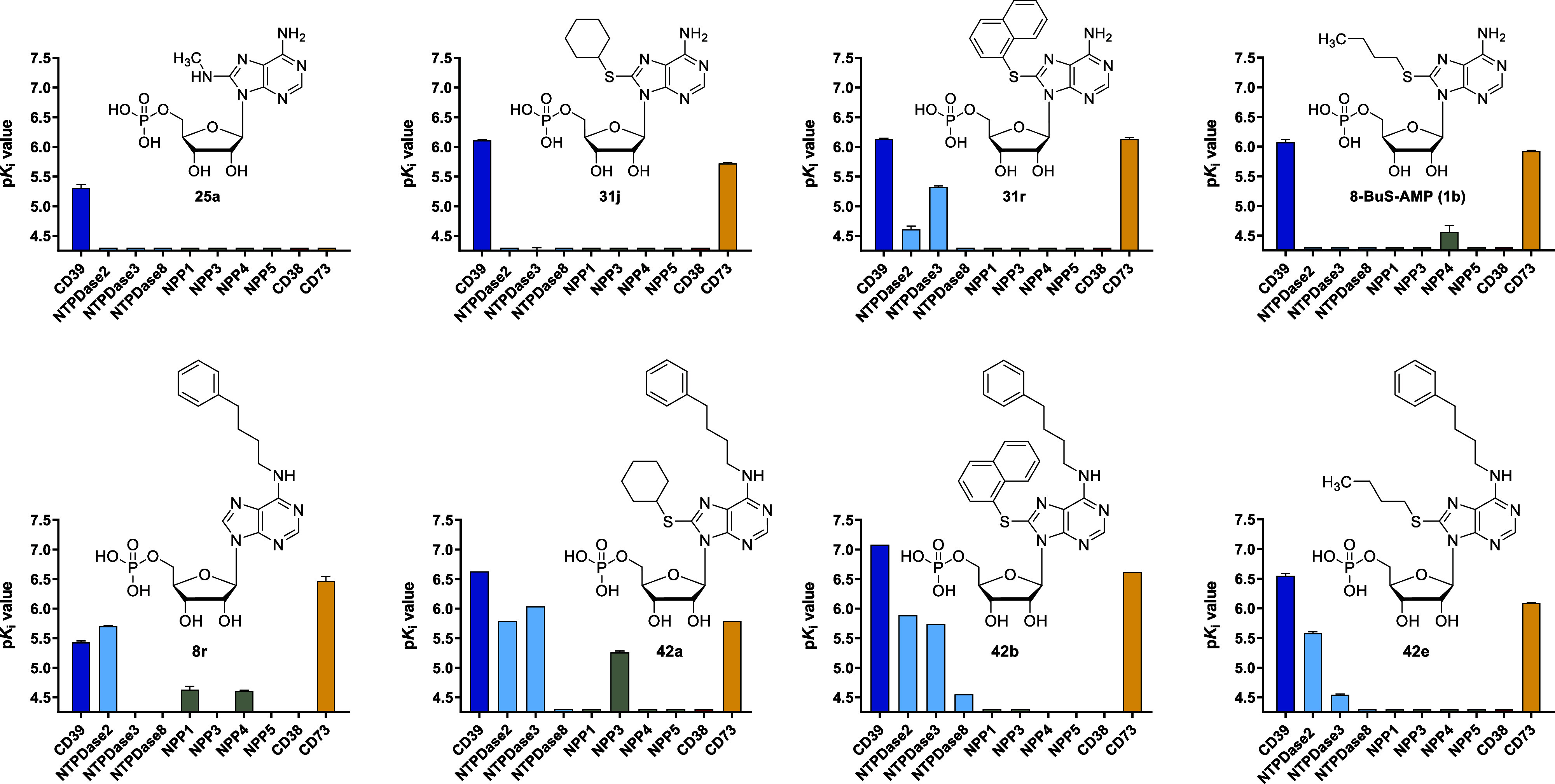
Selectivity profile of CD39 inhibitors **1b**, **8r**, **25a**, **31j**, **31r**, **42a**, **42b**, and **42e** at human ectonucleotidases.

Inhibition of human CD73 was additionally determined for 21 AMP derivatives, with many compounds exhibiting similar *K*_i_ values for CD39 and CD73 (Figure 9 and Supporting Information Table S3). Exceptions include, for example, *N* ^6^-(4-phenylbutyl)-AMP (**8r**), *N* ^6^-(4-phenylbutyl),*N* ^6^-propyl-AMP (**8u**), *N* ^6^-methyl-8-methylamino-AMP (**47a**), 8-butylamino-*N* ^6^-methyl-AMP (**47b**), and 8-butylthio-*N* ^6^-methyl-AMP (**48a**), for which CD73 inhibition was even higher, and *N* ^6^-butyl,*N* ^6^-(4-phenylbutyl)-AMP (**8v**) and 8-methylamino-*N* ^6^-(4-phenylbutyl)-AMP (**42d**), for which CD39 inhibition was significantly higher (Figure 9).

**Figure 9:**
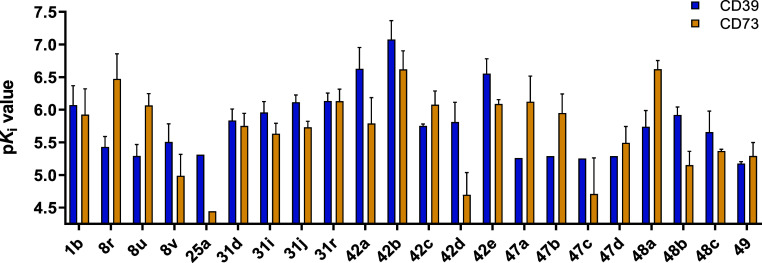
Potency of AMP derivatives as inhibitors of human membrane-bound CD39 (blue) and soluble human CD73 (yellow). p*K*_i_ values for human CD39 inhibition were determined using the malachite green assay, except for compounds **47c** and **48a**, which were evaluated in the fluorescence-based capillary electrophoresis assay. p*K*_i_ values for compounds **47a**–**d** at CD39 and for **25a** at CD73 were estimated based on extrapolation.

#### Metabolic Stability

Metabolic stability profiling of the newly identified inhibitors **8r**, **25a**, **31j**, **42b** and **42e** revealed pronounced differences (Figure 10). *N* ^6^-(4-Phenylbutyl)-AMP (**8r**, Figure 10A) and 8-methylamino-AMP (**25a**, Figure 10B) were rapidly degraded in human and mouse liver microsomes. LC/ESI-MS analysis confirmed that the extremely rapid degradation of **25a** was enzymatic rather than due to chemical instability (data not shown). In contrast, 8-cyclohexylthio-AMP (**31j**, Figure 10D) exhibited metabolic stability with a long half-life (*t*_1/2_ 84 min, human; >2 h, mouse), which suggests that sufficiently bulky substituents such as cyclohexylthio at the 8-position can effectively protect the scaffold from microsomal degradation. This is further demonstrated by the *N* ^6^,8-disubstituted derivatives 8-butylthio-*N* ^6^-(4-phenylbutyl)-AMP (**42e**, Figure 10C) and 8-naphthylthio-*N* ^6^-(4-phenylbutyl)-AMP (**42b**, Figure 10E), which showed high resistance to metabolic degradation. These results suggest that large C8-substituents at the purine core, in particular a naphthylthio residue protects the compounds from metabolic degradation, whereas AMP derivatives without or with smaller C8-substituents, such as methylamino (**25a**), are rapidly degraded in liver microsomes. Accordingly, **31j**, **42b** and **42e** represent promising candidates for in vivo studies, while **25a** and **8r** are primarily suited as in vitro tool compounds.

**Figure 10:**
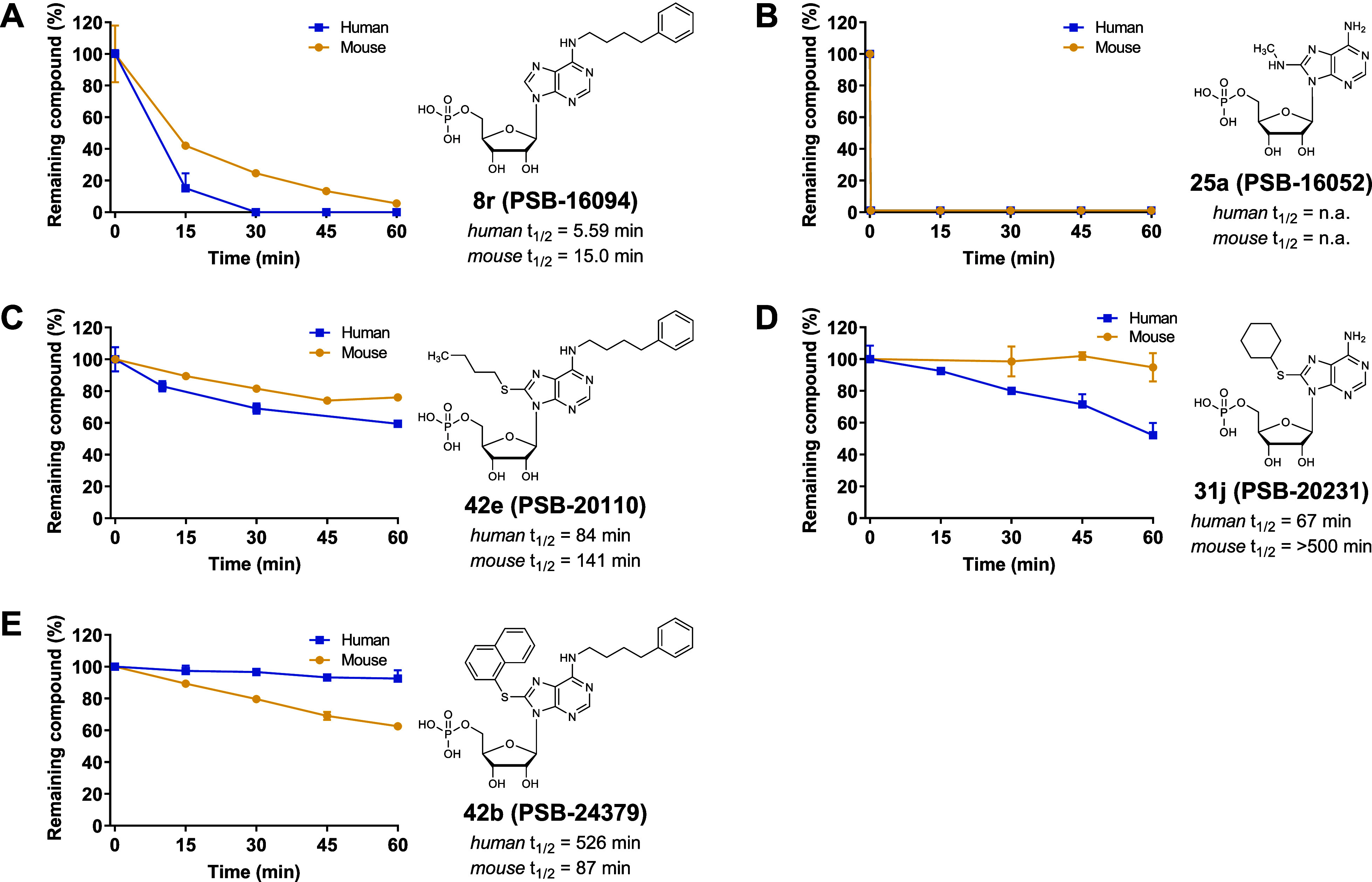
Metabolic stability of CD39 inhibitors **8r** (A), **25a** (B), **42e** (C), **31j** (D), and **42b** (E). Compounds were incubated with pooled mouse or human liver microsomes (mixed gender), and residual concentrations were quantified by LC/ESI-MS. Values are expressed as mean ± SEM and half-lives (*t*_1/2_) were extrapolated.

#### Inhibition of ATPase Activity of Cancer Cell Membrane Preparations

As a next step, the inhibition of ATPase activity by two of the potent CD39 inhibitors, 8-cyclohexylthio-*N* ^6^-(4-phenylbutyl)­amino-AMP (**42a**) and 8-naphthylthio-*N* ^6^-(4-phenylbutyl)­amino-AMP (**42b**), was investigated in a cancer-relevant context using cell membrane preparations of human melanoma cells (Ma-Mel-65). Flow cytometric analyses had indicated that melanoma cell lines, including Ma-Mel-65, exhibit high expression of CD39 and CD73.^86^ Together, both ectonucleotidases catalyze the stepwise conversion of extracellular ATP to AMP (CD39) and ultimately to adenosine (CD73), which promotes the development of an immunosuppressive microenvironment.^87–89^ The enzymatic activity was measured using the malachite green assay to determine the release of phosphate generated by hydrolysis of added ATP.^9^ Compound **42a** induced a concentration-dependent decrease in ATPase activity in the melanoma cell membrane preparations (Figure 11). The more potent inhibitor **42b** showed significant inhibition of ATPase activity at the lowest tested concentration (1 μM) in these preliminary studies.

**Figure 11:**
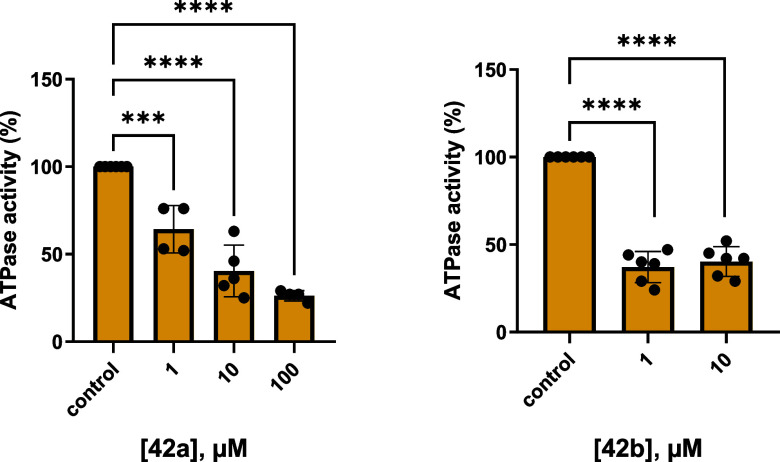
Inhibition of ATPase activity by 8-cyclohexylthio-*N* ^6^-(4-phenylbutyl)­amino-AMP (**42a**) and 8-naphthylthio-*N* ^6^-(4-phenylbutyl)-AMP (**42b**) in human melanoma (Ma-Mel-65) cell membrane preparations. ATP hydrolysis was determined using the malachite green assay with 50 μM of ATP added as a substrate. Cell membrane preparations were incubated for 15 min at 37 °C. Values are normalized to controls (intact enzyme without inhibitor, and denatured enzyme in the presence of ATP, respectively. Bars show means ± SEM from 3 independent experiments. Statistical analysis was performed by one-way ANOVA with Dunnett’s post hoc test (***p < 0.001; ****p < 0.0001) in GraphPad Prism 9.

As a next step, we determined full concentration–inhibition curves for **42b** using the melanoma cells, and a second cancer cell line, the human triple-negative breast cancer (TNBC) cell line MDA-MB-231 (Figure 12). IC_50_ values of 809 ± 0.248 μM (Ma-Mel-65), and 3.94 ± 2.22 μM (MDA-MB-231) were determined. ATP hydrolysis was not completely inhibited by **42b** indicating the presence of additional ATP hydrolysis pathways besides CD39, e.g. nucleotide pyrophosphatases, although CD39 appeared to be predominant in both cell lines.

**Figure 12:**
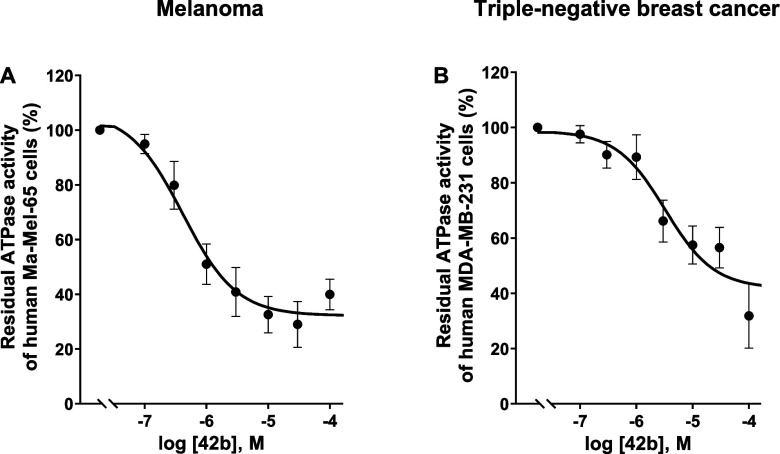
Concentration-dependent inhibition of ATPase activity by 8-(1-naphthylthio)-*N* ^6^-(4-phenylbutyl)-AMP (**42b**) (A). In human triple-negative breast cancer (MDA-MB-231) and (B). In melanoma (Ma-Mel-65) cell membrane preparations. For details see Figure 11. IC_50_ values were 0.809 ± 0.248 μM in melanoma (Ma-Mel-65) cells and 3.94 ± 2.22 μM in MDA-MB-231 cells.

These findings confirm the presence and importance of functional CD39 in Ma-Mel-65 and MDA-MB-231 cell membranes, and underline the potential of inhibiting CD39 to increase ATP concentrations and to prevent adenosine-mediated immunosuppression in melanoma and breast cancer.

#### T Cell Activation and Proliferation Assays and Quantification of ATP Hydrolysis

Finally, inhibitor **42b** was selected to study its impact on T cells obtained from human peripheral blood mononuclear cells. In peripheral blood, CD39 is mainly expressed on antigen-presenting cells such as monocytes, dendritic cells, and B lymphocytes. Within the T cell population, its basal expression is restricted to a subset of regulatory T cells, while activation rapidly induces CD39 on both CD4^+^ and CD8^+^ T cells.^90^ Elevated CD39 levels in T cells are often associated with functional exhaustion, a characteristic feature of chronic infections and cancer.^11,90^ By degrading extracellular ATP, CD39 limits pro-inflammatory signaling and together with CD73-mediated adenosine production, shapes a suppressive immune milieu.^91^

When applied alone, compound **42b** led to a decrease in T cell activation and proliferation (Figure 13). To mimic the microenvironment in inflamed tissue and tumors, we added ATP to the culture. Under these conditions, **42b** resulted in a concentration-dependent increase in activation, as measured by CD25 expression, and proliferation of polyclonally stimulated CD4^+^ and CD8^+^ T cells. These findings suggest that **42b** counteracts adenosine-mediated suppression by preventing the conversion of ATP to adenosine (Figure 13). Cell viability was not affected by **42b** at concentrations of 10 or 50 μM, only at 100 μM (data not shown). The effect of the CD73 inhibitor PSB-14685, included for comparison, was even stronger, probably due to its very high, subnanomolar potency.^92,93^

**Figure 13:**
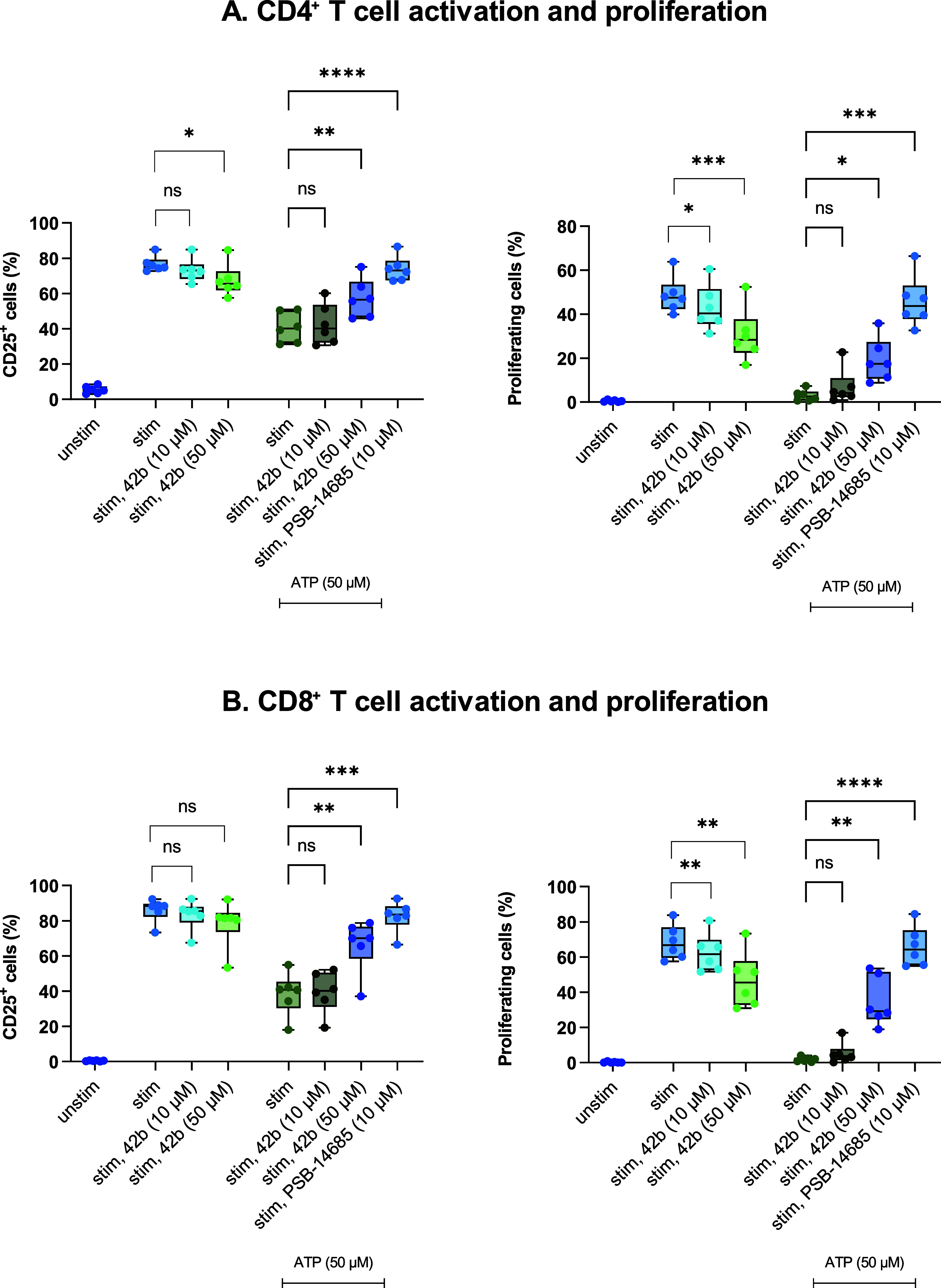
Impact of **42b** and the CD73-specific inhibitor 2-chloro-*N* ^6^-*o*-chlorobenzyl-α,β-methylene-ADP (PSB-14685)^83,84^ on (A) CD4^+^ and (B) CD8^+^ T cell activation and proliferation. T cells were labeled with the proliferation dye eFluor 670 and activated with αCD3/αCD28 in the presence of the adenosine deaminase inhibitor erythro-9-(2-hydroxy-3-nonyl)­adenine (EHNA). Cells were treated with ATP, **42b**, and PSB-14685 as specified in the figure. After 4 days, surface CD25 expression (activation marker) and eFluor 670 dye dilution (marker for proliferation) were analyzed by flow cytometry. Data represent means ± SEM. Each dot corresponds to an individual donor (*n* = 6, as shown). “Unstim” refers to unstimulated cells, whereas “stim” refers to stimulated cells. One-way ANOVA with Dunnett’s multiple comparisons test was performed in GraphPad Prism 9, and significance was set at *p* < 0.05 (*), *p* < 0.01 (**), *p* < 0.001 (***), and *p* < 0.0001 (****).

To further assess CD39 inhibition by **42b**, we determined nucleotide hydrolysis by HPLC using the same compound concentrations as in the proliferation assay, and activated primary human T cells as a source of CD39. Activated T cells exhibited pronounced ATPase activity, as reflected by the conversion of 1 μM etheno-ATP (eATP) into its degradation products eADP and eAMP (Figure 14). Pretreatment with **42b** for 15 min reduced this nucleotide turnover, and a significant inhibition was observed at the lowest tested concentration of 10 μM **42b**, even though this concentration had not affected T-cell proliferation in the proliferation assay (Figure 13). It appeared that inhibition was already maximal at this concentration (Figure 14). These findings show that **42b** efficiently inhibits CD39 enzymatic activity in primary human T cells in a short-term assay. The higher concentrations of **42b** required in the T cell activation and proliferation assays (Figure 13) as compared to the CD39 inhibition assays can be explained by the fact that those are long-term assays lasting for 4 days during which the effective concentration of the inhibitor that reaches and blocks CD39 can be reduced, e.g. due to nonspecific binding or cellular uptake over time.

**Figure 14:**
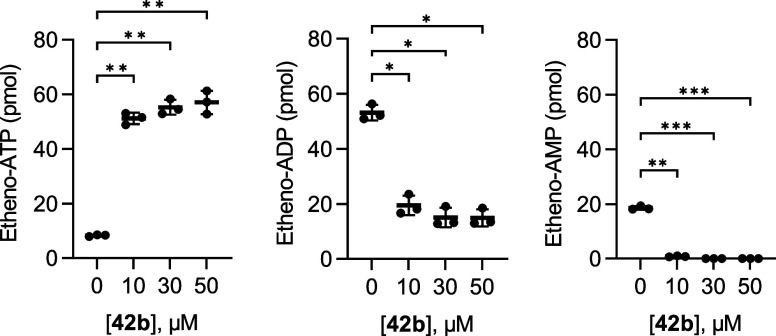
Compound **42b** inhibits the ATPase activity of activated human T cells in a short-term assay. Primary human T cells were activated with αCD3/αCD28 for 4 days. Around 0.2 mio. cells were then pretreated with the indicated concentrations of **42b** for 15 min, followed by a 30 min incubation with 1 μM etheno-ATP. Degradation of etheno-ATP was measured by HPLC. One-way ANOVA with Dunnett’s multiple comparisons test was performed in GraphPad Prism 9 to compare all conditions to the control without inhibitor, with data shown as mean ± SEM from three independent experiments, and significance set at *p* < 0.05 (*), *p* < 0.01 (**), and *p* < 0.001 (***).

## Conclusions

Potent CD39 inhibitors are urgently needed as tool compounds, e.g., for target validation studies, and have potential as novel drugs. In the present study, 88 nucleotide derivatives and analogs related to the previously described CD39 inhibitor 8-butylthio-AMP,^44^ employed as a lead structure, were synthesized, 78 of which are novel compounds, not previously described in literature, and their inhibitory activity at human CD39 was evaluated, in addition to 6 commercially available analogs. SARs were systematically explored, which resulted in the identification of the most potent nucleotide-derived CD39 inhibitors described to date. The combination of an *N* ^6^-(4-phenylbutylamino) substituent with a bulky 8-substituent, preferably cyclohexylthio (**42a**, *K*_i_ 180 nM), naphthylthio (**42b**, *K*_i_ 77.4 nM) or butylthio (**42e**, *K*_i_ 238 nM), was particularly beneficial. These compounds showed even higher potency when tested at the truncated soluble CD39 lacking the transmembrane domains (**42a**, *K*_i_ 11.2 nM; **42b**, *K*_i_ 23.1 nM; **42e**, *K*_i_ 58.9 nM), which is frequently used for drug screening. Mechanistically, inhibition was confirmed to be competitive, and a binding mode was proposed based on docking of **42b** into a homology model of human CD39. Selectivity studies revealed 8-methylamino-AMP (**25a**) as the first small-molecule inhibitor that is selective for CD39. Moreover, dual CD39/CD73 inhibitors, including 8-cyclohexyl-AMP (**31j**), were obtained. We identified *N* ^6^-residues, in particular *N* ^6^-(4-phenylbutyl), which confer additional NTPDase2, and −3 inhibition. Metabolic stability assessed in human and mouse liver microsomes showed that bulky 8-substituents and appropriate *N* ^6^,8-disubstitution patterns (e.g., **31j**, **42b**, **42e**) markedly improve stability, supporting these compounds as drug-like lead compounds suitable for in vivo studies. Importantly, *N* ^6^,8-disubstituted AMP derivatives reduced ATPase activity in human melanoma (Ma-Mel-65) and triple-negative breast cancer (MDA-MB-231) cell membrane preparations and partially limited the suppression of T cell activation by interfering with the generation of adenosine in primary human peripheral blood mononuclear cells in an ATP-containing microenvironment, which supports functional activity in tumor cells, and highlights CD39 and CD73 inhibition as a means to restore antitumor immune signaling. This study provides starting points for further medicinal chemistry optimization and will enable preclinical evaluation of potent small-molecule CD39 inhibitors.

## Experimental Section

### General Chemistry

Reagents were obtained from Acros, Fluorochem, Merck, Carbosynth, Santa Cruz, Sigma-Aldrich, and TCI and used without further purification unless otherwise noted. 8-Azido-AMP, 8-Br-AMP, 2-MeS-ADP, 2-MeS-ATP, and **31s** were obtained from Biolog (Bremen, Germany). Commercial solvents of specific reagent grades were used without additional purification or drying. A 1 M solution of TEAC buffer was prepared by adding dry ice slowly to a 1 M aqueous triethylamine solution for several hours until a pH value of 7.4–7.6 was reached, as measured by a Mettler Toledo (Gieβen, Germany) FiveEasy pH meter. The reactions were monitored by TLC using Merck silica gel 60 F_254_ aluminum 0.255 mm plates, using MeOH/DCM (1:9 or 1:4) as the mobile phase. TLC plates were analyzed by UV light irradiation (254 nm). Column chromatography was carried out with silica gel 0.040–0.060 mm, pore diameter 60 Å. Anion exchange chromatography was performed on an FPLC instrument (ÄKTA FPLC, Amersham Biosciences) with a HiPrep Q Fast Flow (FF) sepharose column, 16 ⊆ 100 mm (GE Healthcare Life Sciences, Chicago, USA). Elution of the nucleoside monophosphates was achieved with a linear gradient (5–100%, 0.5 M aqueous ammonium bicarbonate buffer in H_2_O, 8 column volumes, flow 1 mL/min). Semipreparative HPLC was performed on a Knauer Smartline 1050 HPLC system equipped with a Eurospher-100C18 column, 250 mm ⊆ 20 mm, particle size 10 μm with detection of UV absorption at 254 nm. Appropriate fractions were pooled, diluted with H_2_O, and lyophilized several times, using a CHRIST ALPHA 1–4 LSC freeze-dryer, to remove the NH_4_HCO_3_ buffer, yielding the nucleoside monophosphates as white powders. Mass spectra were recorded on an API 2000 mass spectrometer (Applied Biosystems, Darmstadt, Germany) with a turbo ion spray ion source coupled with an Agilent 1100 HPLC system (Agiland, Böblingen, Germany) using a EC50/2 Nucleodur C18 Gravity 3 μm (Macherey-Nagel, Düren, Germany), or on a micrOTOF-Q mass spectrometer (Bruker, Köln, Germany) with ESI-source coupled with a HPLC Dionex Ultimate 3000 (Thermo Scientific, Braunschweig, Germany) using an EC50/2 Nucleodur C18 Gravity 3 μm column (Macherey-Nagel, Düren, Germany). UV absorption was detected from 220 to 600 nm using the diode array detector (DAD). NMR spectra were recorded on Bruker Avance 500 and 600 MHz spectrometers. DMSO-*d*_6_, CD_3_OD, or D_2_O were used as solvent. ^31^P NMR spectra were recorded at 25 °C; phosphoric acid was used as an external standard. For spectra recorded in D_2_O, 3-(trimethylsilyl)­propionic-2,2,3,3 acid sodium salt-*d*_*4*_ was used as external standard. When DMSO-*d*_6_ was used, spectra were recorded at 30 °C. Shifts are given in ppm relative to the external standard (in ^31^P NMR) or relative to the remaining protons of the deuterated solvents used as internal standard (^1^H-, ^13^C NMR). Coupling constants are given in Hertz (Hz). The designation used to assign the peaks in the spectra is as follows: singlet (s), doublet (d), triplet (t), quartet (q), multiplet (m), broad (br). Melting points were determined on a Buchi 530 melting point apparatus and are uncorrected.

### General Procedure for the Synthesis of 2′,3′-Unprotected Nucleotides

The 2′,3′-unprotected nucleoside (1 equiv) was dissolved in trimethyl phosphate (5 mL), and proton-sponge was added. The mixture was cooled to 0 °C under argon, and POCl_3_ (4 equiv) was added 5 min later. The mixture was stirred at 0 °C for 3–4 h and monitored by TLC (2-propanol: NH_4_OH (25% in H_2_O): H_2_O = 6:3:1). After the reaction was completed, the mixture was quenched by cold 0.5 M aqueous TEAC buffer (pH 7.4–7.6), or H_2_O, or saturated aqueous NH_4_HCO_3_ until pH ≥ 8, and stirred at 0 °C for several minutes. The solution was aged at rt for 1 h. PO­(OCH_3_)_3_ and proton-sponge were extracted with MTBE (500 mL), and the crude product was lyophilized. Purification was achieved by preparative HPLC (0–80% MeCN in 50 mM NH_4_HCO_3_ buffer in 20 min, 20 mL/min; or 5–70% MeCN (+0.05% TFA) in H_2_O (+0.05% TFA) in 16 min, 25 mL/min). Appropriate fractions were collected and lyophilized multiple times to remove TEAC, NH_4_HCO_3_, and TFA, yielding the desired nucleotide.

### General Procedure for the Synthesis of 2′,3′-Protected Nucleotides and Deprotection

The 2′,3′-protected nucleoside (1 equiv) was dissolved in PO­(OCH_3_)_3_ (5 mL). The mixture was cooled to 0 °C under argon, POCl_3_ (4 equiv) was added 5 min later. The mixture was stirred at 0 °C for 6–7 h and monitored by TLC (2-propanol: NH_4_OH (25% in H_2_O): H_2_O = 6:3:1). After the reaction was completed, the mixture was quenched by cold 0.5 M aqueous TEAC buffer (pH 7.4–7.6) and stirred at 0 °C for several minutes. The solution was aged at rt for 1 h. PO­(OCH_3_)_3_ was extracted with MTBE (500 mL), and the crude product was lyophilized. Purification was achieved by preparative HPLC (10–75% MeCN in 50 mM NH_4_HCO_3_ buffer in 20 min, 20 mL/min). Appropriate fractions were collected and lyophilized, yielding the 2′,3′-*O*-isopropylidene nucleotide, which was dissolved in 7–8% TFA in H_2_O/DCM (1:9) (5 mL) and the reaction mixture was stirred at rt for 2 h. The solvent was evaporated, and precipitation was induced by the addition of diethyl ether. The ether was decanted and the crude product again purified by preparative HPLC (0–50% MeCN in 50 mM NH_4_HCO_3_ buffer in 20 min, 20 mL/min). Lyophilization afforded the desired nucleotide.

#### ((2*R*,3*S*,4*R*,5*R*)-5-(2,6-Diamino-9*H*-purin-9-yl)-3,4-dihydroxytetra-hydrofuran-2-yl)­methyl dihydrogen Phosphate (**3a**)

The compound was synthesized using **2a** (100 mg, 0.35 mmol) and afforded a white powder (60 mg, 45%), mp: 228 °C. ^1^H NMR (600 MHz, D_2_O) δ: 8.20 (s, 1H), 5.95 (d, 1H, *J* = 5.89 Hz), 4.49 (br s, 1H), 4.34 (s, 1H), 4.05 (s, 2H), 3.59 (d, 1H, *J* = 10.56 Hz). ^13^C NMR (150 MHz, D_2_O) δ: 162.63, 158.77, 154.03, 140.47, 115.79, 89.18, 87.19, 76.78, 73.46, 66.78. ^31^P NMR (202 MHz, D_2_O) δ: 2.62. LC/ESI-MS (*m*/*z*): positive mode 363.0812 [M + H]^+^ and negative mode 361.0666 [M – H]^−^. Purity by HPLC-UV (254 nm)-ESI-MS: 99.5%.

#### ((2R,3S,4R,5R)-5-(6-Amino-2-hydrazinyl-9*H*-purin-9-yl)-3,4-dihydroxytetrahydrofuran-2-yl)-methyl dihydrogen Phosphate (**3b**)

The compound was synthesized using **2c** (100 mg, 0.30 m mol) and afforded a white solid (70 mg, 58%), mp: 77 °C. ^1^H NMR (600 MHz, D_2_O) δ: 8.23 (s, 1H), 6.03 (d, 1H, *J* = 5.98 Hz), 4.86 (t, 1H, *J* = 5.25 Hz), 4.51 (t, 1H, *J* = 4.23 Hz), 4.34 (d, 1H, *J* = 2.97 Hz), 4.03 (t, 2H, *J* = 4.23 Hz). ^13^C NMR (125 MHz, D_2_O) δ: 158.74, 154.08, 140.90, 132.50, 116.32, 89.47, 87.22, 76.66, 73.51, 66.63. ^31^P NMR (202 MHz, D_2_O) δ: 5.33. LC/ESI-MS (*m*/*z*): positive mode 378.0911 [M + H]^+^ and negative mode 376.0774 [M – H]^−^. Purity by HPLC-UV (254 nm)-ESI-MS: 100.0%.

#### ((2*R*,3*S*,4*R*,5*R*)-5-(6-Amino-2-chloro-9*H*-purin-9-yl)-3,4-dihydroxytetrahydrofuran-2-yl)-methyl dihydrogen Phosphate (**3c**)

The compound was synthesized using **2b** (100 mg, 0.33 mmol) and afforded a white solid (100 mg, 84%), mp: 185 °C. ^1^H NMR (600 MHz, D_2_O) δ: 8.57 (s, 1H), 6.04 (d, 1H, *J* = 5.60 Hz), 4.50 (m, 1H), 4.36 (br s, 1H), 4.01 (br s, 2H), 3.59 (d, 1H, *J* = 10.69 Hz). ^13^C NMR (150 MHz, D_2_O) δ: 163.08, 159.07, 153.07, 143.24, 120.46, 89.81, 87.62, 77.48, 73.48, 66.30. ^31^P NMR (202 MHz, D_2_O) δ: 3.99. LC/ESI-MS (*m*/*z*): positive mode 382.0313 [M + H]^+^ and negative mode 380.0172 [M – H]^−^. Purity by HPLC-UV (254 nm)-ESI-MS: 99.9%.

#### ((2*R*,3*S*,4*R*,5*R*)-5-(6-Amino-2-(methylthio)-9*H*-purin-9-yl)-3,4-dihydroxytetrahydrofuran-2-yl)­methyl dihydrogen Phosphate (**3d**)

The compound was synthesized using **5** (200 mg, 0.67 mmol) and afforded a white powder (20 mg, 8%), mp: 85 °C. ^1^H NMR (500 MHz, D_2_O) δ: 8.44 (s, 1H), 6.12 (d, 1H, *J* = 5.78 Hz), 4.53 (m, 1H), 4.34 (br s, 1H), 3.99 (d, 2H, *J* = 3.77 Hz), 3.59 (d, 1H, *J* = 10.69 Hz), 2.58 (s, 3H). ^13^C NMR (125 MHz, D_2_O) δ: 169.08, 163.28, 153.23, 142.25, 119.07, 87.61, 87.38, 77.03, 73.59, 66.43, 16.53. ^31^P NMR (202 MHz, D_2_O) δ: 4.09. LC/ESI-MS (*m*/*z*): positive mode 394.0582 [M + H]^+^ and negative mode 392.0425 [M – H]^−^. Purity by HPLC-UV (254 nm)-ESI-MS: 100.0%.

#### ((2*R*,3*S*,4*R*,5*R*)-3,4-Dihydroxy-5-(6-(methylamino)-9*H*-purin-9-yl)­tetrahydrofuran-2-yl)­methyl dihydrogen Phosphate (**8a**)

The compound was synthesized using **7a** (100 mg, 0.36 mmol) and afforded a white powder (90 mg, 74%), mp: 105 °C. ^1^H NMR (600 MHz, D_2_O) δ: 8.55 (s, 1H), 8.23 (s, 1H), 6.12 (d, 1H, *J* = 5.92 Hz), 4.51 (m, 1H), 4.36 (d, 1H, *J* = 3.42 Hz), 4.00 (d, 2H, *J* = 4.04 Hz), 3.59 (d, 1H, *J* = 10.69 Hz), 3.08 (br s, 3H). ^13^C NMR (150 MHz, D_2_O) δ: 163.11, 158.00, 155.67, 142.29, 121.82, 89.51, 87.68, 77.29, 73.29, 73.56, 66.52, 30.17. ^31^P NMR (202 MHz, D_2_O) δ: 4.22. LC/ESI-MS (*m*/*z*): positive mode 362.0864 [M + H]^+^ and negative mode 360.0713 [M – H]^−^. Purity determined by HPLC-UV (254 nm)-ESI-MS: 99.9%.

#### ((2*R*,3*S*,4*R*,5*R*)-5-(6-(Ethylamino)-9*H*-purin-9-yl)-3,4-dihydroxytetra-hydrofuran-2-yl)­methyl dihydrogen Phosphate (**8b**)

The compound was synthesized using **7b** (100 mg, 0.35 mmol) and afforded a white powder (80 mg, 65%), mp: 125 °C. ^1^H NMR (600 MHz, D_2_O) δ: 8.50 (s, 1H), 8.21 (s, 1H), 6.12 (d, 1H, *J* = 5.93 Hz), 4.78 (d, 1H, *J* = 5.46 Hz), 4.50 (m, 1H), 4.37 (s, 1H), 4.05 (s, 2H), 3.83 (d, 1H), 3.59 (br s, 2H), 1.28 (overlapping m, NHCH_2_C*H*_3_ & TEAC: N­(CH_2_C*H*_3_)_3_). ^13^CNMR (150 MHz, D_2_O) δ: 163.13, 158.01, 155.70, 142.30, 121.72, 89.51, 87.69, 77.30, 73.57, 66.26, 55.59, 16.54. ^31^P NMR (202 MHz, D_2_O) δ: 2.80. LC/ESI-MS (*m*/*z*): positive mode 376.1020 [M + H]^+^ and negative mode 374.0859 [M – H]^−^. Purity determined by HPLC-UV (254 nm)-ESI-MS: 97.2%.

#### ((2*R*,3*S*,4*R*,5*R*)-3,4-Dihydroxy-5-(6-(isobutylamino)-9*H*-purin-9-yl)­tetrahydrofuran-2-yl)­methyl dihydrogen Phosphate (**8c**)

The compound was synthesized using **7c** (150 mg, 0.46 mmol) and afforded a white powder (18 mg, 10%), mp: 191.0–193.0 °C. ^1^H NMR (DMSO-*d*_6_) δ: 8.46 (s, 1H), 8.20 (s, 1H), 7.79 (s, 1H), 5.92 (d, *J* = 6.1 Hz, 1H), 4.66 (t, *J* = 5.5 Hz, 1H), 4.24 (t, *J* = 3.8 Hz, 2H), 4.04 (s, 1H), 3.83 (t, *J* = 5.0 Hz, 3H), 3.30 (s, 2H), 1.96 (s, 1H), 0.89 (d, *J* = 6.7 Hz, 6H). ^13^C NMR (DMSO-*d*_6_) δ: 154.71, 152.54, 148.94, 139.02, 118.98, 86.56, 84.30, 73.99, 71.14, 64.11, 64.08, 27.82, 20.09. ^31^P NMR (DMSO-*d*_6_) δ: 1.11. LC–MS (*m*/*z*): positive mode 404.3 [M + H]^+^. Purity by HPLC-UV (254 nm)-ESI-MS: 97.9%.

#### ((2*R*,3*S*,4*R*,5*R*)-3,4-Dihydroxy-5-(6-(iso-pentylamino)-9*H*-purin-9-yl)­tetrahydrofuran-2-yl)­methyl dihydrogen Phosphate (**8d**)

The compound was synthesized using **7d** (100 mg, 0.30 mmol) and afforded a white solid (20 mg, 18%), mp: 114 °C. ^1^H NMR (500 MHz, D_2_O) δ: 8.42 (s, 1H), 8.16 (s, 1H), 6.09 (d, 1H, *J* = 5.85 Hz), 4.74 (t, 1H, *J* = 5.48 Hz), 4.49 (m, 1H), 4.37 (br s, 1H), 4.09 (br s, 2H), 3.50 (br s, 2H), 1.69 (m, 1H), 1.54 (q, 2H, *J* = 7.12 Hz), 0.92 (d, 6H, *J* = 6.65 Hz). ^13^C NMR (125 MHz, D_2_O) δ: 157.23, 155.58, 150.59, 141.84, 121.59, 89.62, 87.00, 77.23, 73.34, 66.94, 41.93, 40.16, 28.02, 24.56. ^31^P NMR (202 MHz, D_2_O) δ: 1.64. LC/ESI-MS (*m*/*z*): positive mode 418.1479 [M + H]^+^ and negative mode 416.1338 [M – H]^−^. Purity by HPLC-UV (254 nm)-ESI-MS: 96.0%.

#### ((2*R*,3*S*,4*R*,5*R*)-3,4-Dihydroxy-5-(6-((2,4,4-trimethylpentan-2-yl)­amino)-9*H*-purin-9-yl)­tetra-hydrofuran-2-yl)­methyl dihydrogen Phosphate (**8e**)

The compound was synthesized using **7e** (100 mg, 0.26 mmol) and afforded a white powder (20 mg, 13%), mp: 183 °C. ^1^H NMR (500 MHz, D_2_O) δ: 8.44 (s, 1H), 8.22 (s, 1H), 6.10 (d, 1H, *J* = 5.95 Hz), 4.75 (t, 1H, *J* = 5.55 Hz), 4.49 (m, 1H), 4.37 (m, 1H), 4.07 (dd, 2H, *J* = 2.89, 4.80 Hz), 1.97 (s, 2H), 1.56 (s, 6H), 0.91 (s, 9H). ^13^C NMR (125 MHz, D_2_O) δ: 157.23, 155.37, 150.39, 141.66, 122.35, 89.34, 87.20, 87.15, 77.25, 73.45, 66.96, 58.73, 52.52, 33.76, 33.67, 33.48, 32.12, 32.09. ^31^P NMR (202 MHz, D_2_O) δ: 1.93. LC/ESI-MS (*m*/*z*): positive mode 460.1875 [M + H]^+^ and negative mode 458.1786 [M – H]^−^. Purity by HPLC-UV (254 nm)-ESI-MS: 98.0%.

#### ((2*R*,3*S*,4*R*,5*R*)-5-(6-(Hexylamino)-9*H*-purin-9-yl)-3,4-dihydroxytetrahydrofuran-2-yl)-methyl dihydrogen Phosphate (**8f**)

The compound was synthesized using **7f** (100 mg, 0.28 mmol) and afforded a white powder (30 mg, 22%), mp: 109 °C. ^1^H NMR (500 MHz, D_2_O) δ: 8.40 (s, 1H), 8.16 (s, 1H), 6.09 (d, *J* = 5.80 Hz, 1H), 4.73 (t, 1H, *J* = 5.46 Hz), 4.49 (m, 1H), 4.37 (br s, 1H), 4.12 (q, 2H, *J* = 3.70, 4.12 Hz), 3.47 (br s, 2H), 1.62 (m, 2H), 1.35 (m, 2H), 1.25 (m, 4H), 0.82 (t, 3H, *J* = 6.94 Hz). ^13^C NMR (125 MHz, D_2_O) δ: 157.15, 155.41, 150.58, 141.80, 121.58, 89.69, 86.83, 77.23, 73.28, 72.42, 67.15, 33.64, 31.24, 28.57, 24.75, 16.11. ^31^P NMR (202 MHz, D_2_O) δ: 0.92. LC/ESI-MS (*m*/*z*): positive mode 432.1643 [M + H]^+^ and negative mode 430.1497 [M – H]^−^. Purity determined by HPLC-UV (254 nm)-ESI-MS: 96.0%.

#### ((2*R*,3*S*,4*R*,5*R*)-5-(6-(Dimethylamino)-9*H*-purin-9-yl)-3,4-dihydroxytetrahydrofuran-2-yl)-methyl dihydrogen Phosphate (**8g**)

The compound was synthesized using **7g** (290 mg, 1.00 mmol) and afforded a white powder (11 mg, 3%), mp: 87 °C (*lit*.^94^ 223 °C (decomposition)). ^1^H NMR (500 MHz, D_2_O) δ: 8.38 (s, 1H), 8.11 (s, 1H), 6.09 (d, 1H, *J* = 5.54 Hz), 4.71 (t, 1H, *J* = 5.30 Hz), 4.49 (t, 1H, *J* = 4.37 Hz), 4.38 (br s, 1H), 4.13 (m, 2H), 3.37 (s, 6H). ^13^C NMR (125 MHz, D_2_O) δ: 156.42, 154.11, 151.72, 140.60, 121.64, 89.69, 86.61, 77.10, 73.05, 67.09, 50.64, 41.54. ^31^P NMR (202 MHz, D_2_O) δ: 0.59. LC/ESI-MS (*m*/*z*): positive mode 376.1016 [M + H]^+^ and negative mode 374.0882 [M – H]^−^. Purity determined by HPLC-UV (254 nm)-ESI-MS: 95.8%.

#### ((2*R*,3*S*,4*R*,5*R*)-5-(6-(Ethyl­(methyl)­amino)-9*H*-purin-9-yl)-3,4-dihydroxytetrahydrofuran-2-yl)-methyl dihydrogen Phosphate (**8h**)

The compound was synthesized using **7h** (300 mg, 1.00 mmol) and afforded a white solid (50 mg, 14%), mp: 173 °C. ^1^H NMR (500 MHz, D_2_O) δ: 8.40 (s, 1H), 8.07 (s, 1H), 6.07 (d, 1H, *J* = 5.46 Hz), 4.72 (t, 1H, *J* = 5.30 Hz), 4.49 (t, 1H, *J* = 4.52 Hz), 4.36 (m, 1H), 4.08 (m, 2H), 3.83 (s, 2H), 3.25 (s, 3H), 1.18 (t, 3H, *J* = 7.11 Hz). ^13^C NMR (125 MHz, D_2_O) δ: 156.38, 154.87, 151.93, 140.62, 121.43, 89.68, 80.40, 77.23, 73.21, 66.74, 48.78, 39.23, 14.73. ^31^P NMR (202 MHz, D_2_O) δ: 2.06. LC/ESI-MS (*m*/*z*): positive mode 390.1165 [M + H]^+^ and negative mode 388.1029 [M – H]^−^. Purity by HPLC-UV (254 nm)-ESIMS: 98.2%.

#### ((2*R*,3*S*,4*R*,5*R*)-3,4-Dihydroxy-5-(6-(methyl-(propyl)­amino)-9*H*-purin-9-yl)­tetrahydrofuran-2-yl)­methyl dihydrogen Phosphate (**8i**)

The compound was synthesized using **7i** (320 mg, 1.00 mmol) and afforded a white solid (70 mg, 17%), mp: 148 °C. ^1^H NMR (500 MHz, D_2_O) δ: 8.44 (s, 1H), 8.09 (s, 1H), 6.09 (d, 1H, *J* = 5.57 Hz), 4.74 (t, 1H, *J* = 5.32 Hz), 4.50 (t, 1H, *J* = 4.43 Hz), 4.36 (br s, 1H), 4.06 (m, 2H), 3.82 (br s, 2H), 3.28 (br s, 3H), 1.64 (m, 2H), 0.87 (t, 3H, *J* = 7.39 Hz). ^13^C NMR (125 MHz, D_2_O) δ: 156.83, 154.90, 157.07, 140.60, 121.55, 89.63, 87.1, 77.24, 73.29, 66.58, 55.19, 39.97, 23.19, 12.97. ^31^P NMR (202 MHz, D_2_O) δ: 2.66. LC/ESI-MS (*m*/*z*): positive mode 404.1316 [M + H]^+^ and negative mode 402.1187 [M – H]^−^. Purity by HPLC-UV (254 nm)-ESI-MS: 99.5%.

#### ((2*R*,3*S*,4*R*,5*R*)-5-(6-(Diethylamino)-9*H*-purin-9-yl)-3,4-dihydroxytetrahydrofuran-2-yl)-methyl dihydrogen Phosphate (**8j**)

The compound was synthesized using **7j** (320 mg, 1.00 mmol) and afforded a white powder (150 mg, 36%), mp: 194 °C. ^1^H NMR (500 MHz, D_2_O) δ: 8.53 (s, 1H), 8.13 (s, 1H), 6.11 (d, 1H, *J* = 5.63 Hz), 4.76 (t, 1H, *J* = 5.36 Hz), 4.51 (t, 1H, *J* = 4.49 Hz), 4.35 (br s, 1H), 4.01 (m, 2H), 3.84 (br s, 4H), 1.23 (t, 6H, *J* = 7.09 Hz). ^13^C NMR (125 MHz, D_2_O) δ: 156.10, 155.11, 152.24, 140.95, 121.33, 89.54, 87.43, 77.26, 73.37, 66.16, 46.64, 15.46. ^31^P NMR (202 MHz, D_2_O) δ: 4.03. LC/ESI-MS (*m*/*z*): positive mode 404.1321 [M + H]^+^ and negative mode 402.1185 [M – H]^−^. Purity determined by HPLC-UV (254 nm)-ESI-MS: 99.3%.

#### ((2*R*,3*S*,4*R*,5*R*)-5-(6-(Ethyl­(propyl)­amino)-9*H*-purin-9-yl)-3,4-dihydroxytetrahydrofuran-2-yl)­methyl dihydrogen Phosphate (**8k**)

The compound was synthesized using **7k** (330 mg, 1.00 mmol) and afforded a white solid (100 mg, 14%), mp: 165 °C. ^1^H NMR (500 MHz, D_2_O) δ: 8.44 (s, 1H), 8.10 (s, 1H), 6.09 (d, 1H, *J* = 5.60 Hz), 4.73 (t, 1H, *J* = 5.34 Hz), 4.49 (m, 1H), 4.36 (m, 1H), 4.06 (m, 2H), 3.76 (br d, 4H, *J* = 65.6 Hz), 1.63 (m, 2H), 1.19 (t, 3H, *J* = 7.08 Hz), 0.89 (t, 3H, *J* = 7.40 Hz). ^13^C NMR (125 MHz, D_2_O) δ: 156.26, 154.99, 152.19, 140.65, 121.33, 89.60, 87.09, 77.40, 73.28, 66.56, 53.12, 47.03, 23.53, 15.37, 13.11. ^31^P NMR (202 MHz, D_2_O) δ: 2.58. LC/ESI-MS (*m*/*z*): positive mode 418.1479 [M + H]^+^ and negative mode 416.1320 [M – H]^−^. Purity by HPLC-UV (254 nm)-ESI-MS: 97.0%.

#### ((2*R*,3*S*,4*R*,5*R*)-5-(6-(Dipropylamino)-9*H*-purin-9-yl)-3,4-dihydroxytetrahydrofuran-2-yl)-methyl dihydrogen Phosphate (**8l**)

The compound was synthesized using **7l** (350 mg, 1.00 mmol) and afforded a white solid (180 mg, 43%), mp: 178 °C. ^1^H NMR (500 MHz, D_2_O) δ: 8.41 (s, 1H), 8.10 (s, 1H), 6.09 (d, 1H, *J* = 5.59 Hz), 4.72 (t, 1H, *J* = 5.32 Hz), 4.49 (t, 1H, *J* = 4.49 Hz), 4.36 (s, 1H), 4.09 (m, 2H), 3.73 (s, 4H), 1.60 (m, 4H), 0.87 (t, *J* = 7.37 Hz, 6H). ^13^C NMR (125 MHz, D_2_O) δ: 156.50, 154.93, 152.21, 140.48, 121.43, 89.61, 86.95, 77.24, 73.25, 66.78, 53.53, 50.88, 23.44, 13.09. ^31^P NMR (202 MHz, D_2_O) δ: 1.91. LC/ESI-MS (*m*/*z*): positive mode 432.1640 [M + H]^+^ and negative mode 430.1502 [M – H]^−^. Purity by HPLC-UV (254 nm)-ESI-MS: 99.2%.

#### ((2*R*,3*S*,4*R*,5*R*)-5-(6-(Dibutylamino)-9*H*-purin-9-yl)-3,4-dihydroxytetrahydrofuran-2-yl)­methyl dihydrogen Phosphate (**8m**)

The compound was synthesized using **7m** (150 mg 0.40 mmol) and afforded a white powder (55 mg, 30%), mp: 177.5–179.0 °C. ^1^H NMR (DMSO-*d*_6_) δ: 8.38 (s, 1H), 8.20 (s, 1H), 5.95 (d, *J* = 6.3 Hz, 1H), 4.63 (dd, *J* = 6.3, 4.9 Hz, 2H), 4.20 (dd, *J* = 4.9, 2.9 Hz, 2H), 4.05 (d, *J* = 3.6 Hz, 3H), 3.89 (dt, *J* = 6.8, 4.5 Hz, 3H), 3.83–3.40 (m, 3H), 1.61 (p, *J* = 7.5 Hz, 4H), 1.33 (h, *J* = 7.5 Hz, 4H), 0.91 (t, *J* = 7.4 Hz, 6H). ^13^C NMR (DMSO-*d*_6_) δ: 153.49, 151.99, 150.58, 137.95, 118.82, 86.44, 83.93, 83.88, 73.77, 70.98, 64.57, 64.53, 19.53, 13.86. ^31^P NMR (DMSO-*d*_6_) δ: 0.95. LC–MS (*m*/*z*): positive mode 460.2 [M + H]^+^. Purity by HPLC-UV (254 nm)-ESI-MS: 95.0%.

#### ((2*R*,3*S*,4*R*,5*R*)-3,4-Dihydroxy-5-(6-(phenylamino)-9*H*-purin-9-yl)­tetrahydrofuran-2-yl)­methyl dihydrogen Phosphate (**8n**), CAS: 105740–46–3

The compound was synthesized using **7n** (100 mg, 0.29 mmol) and afforded a white powder (67 mg, 55%), mp: 172.0–174.0 °C. ^1^H NMR (DMSO-*d*_6_) δ: 9.88 (s, 1H), 8.69 (s, 1H), 8.40 (s, 1H), 7.99–7.90 (m, 2H), 7.40–7.26 (m, 2H), 7.08–6.99 (m, 1H), 6.00 (d, *J* = 6.1 Hz, 1H), 4.72 (dd, *J* = 6.1, 4.8 Hz, 1H), 4.27 (dd, *J* = 4.8, 3.0 Hz, 2H), 4.08 (q, *J* = 3.6 Hz, 2H), 3.86 (dd, *J* = 6.7, 3.8 Hz, 4H). ^13^C NMR (DMSO-*d*_6_) δ: 152.05, 149.90, 140.36, 139.67, 128.36, 122.52, 120.74, 119.79, 86.74, 84.53, 74.19, 71.20, 63.99. ^31^P NMR (DMSO-*d*_6_) δ: 1.25. LC–MS (*m*/*z*): positive mode 424.2 [M + H]^+^. Purity by HPLC-UV (254 nm)-ESI-MS: 99.4%.

#### ((2*R*,3*S*,4*R*,5*R*)-5-(6-(Benzylamino)-9*H*-purin-9-yl)-3,4-dihydroxytetrahydrofuran-2-yl)­methyl dihydrogen Phosphate (**8o**), CAS: 13484–66–7

The compound was synthesized using **7o** (100 mg, 0.28 mmol) and afforded a white powder (62 mg, 51%), mp: 168.0–170.0 °C. ^1^H NMR (DMSO-*d*_6_) δ: 8.50 (s, 1H), 8.20 (s, 1H), 7.33 (d, *J* = 7.5 Hz, 2H), 7.28 (t, *J* = 7.6 Hz, 2H), 7.22–7.17 (m, 1H), 5.93 (d, *J* = 6.2 Hz, 1H), 4.76–4.65 (m, 3H), 4.24 (dd, *J* = 4.9, 2.9 Hz, 2H), 4.04 (q, *J* = 3.6 Hz, 3H), 3.82 (dd, *J* = 6.9, 3.7 Hz, 4H). ^13^C NMR (DMSO-*d*_6_) δ: 154.37, 152.54, 149.16, 140.10, 139.38, 128.18, 127.06, 126.54, 119.08, 86.60, 84.37, 74.07, 71.22, 64.02, 42.86. ^31^P NMR (DMSO-*d*_6_) δ: 1.15. LC–MS (*m*/*z*): positive mode 438.3 [M + H]^+^. Purity by HPLC-UV (254 nm)-ESI-MS: 99.5%.

#### ((2*R*,3*S*,4*R*,5*R*)-5-(6-Benzamido-9*H*-purin-9-yl)-3,4-dihydroxytetrahydrofuran-2-yl)­methyl dihydrogen Phosphate (**8p**), CAS: 40871–55–4

The compound was synthesized using **7p** (100 mg, 0.27 mmol) and afforded a white powder (47 mg, 39%), mp: 173.0–174.5 °C. ^1^H NMR (DMSO-*d*_6_) δ: 8.89 (s, 1H), 8.74 (s, 1H), 8.09–8.03 (m, 2H), 7.66–7.61 (m, 1H), 7.54 (t, *J* = 7.8 Hz, 2H), 6.07 (d, *J* = 6.2 Hz, 1H), 4.76 (dd, *J* = 6.2, 4.7 Hz, 2H), 4.27 (dd, *J* = 4.8, 2.8 Hz, 2H), 4.09 (q, *J* = 3.4 Hz, 2H), 3.85 (dd, *J* = 6.6, 3.6 Hz, 3H) (The proton of N*H* is missing due to exchangeable proton with DMSO-*d*_6_). ^13^C NMR (DMSO-*d*_6_) δ: 165.68, 152.60, 151.60, 150.11, 143.11, 133.44, 132.35, 128.45, 128.42, 125.32, 86.91, 84.78, 74.22, 71.30, 63.92. ^31^P NMR (DMSO-*d*_6_) δ: 1.09. LC–MS (*m*/*z*): positive mode 452.3 [M + H]^+^. Purity by HPLC-UV (254 nm)-ESI-MS: 99.7%.

#### ((2*R*,3*S*,4*R*,5*R*)-5-(6-((3-(1*H*-Imidazole-1-yl)-propyl)­amino)-9*H*-purin-9-yl)-3,4-dihydroxytetra-hydrofuran-2-yl)­methyl dihydrogen Phosphate (**8q**)

The compound was synthesized using **7q** (100 mg, 0.26 mmol) and afforded a white powder (20 mg, 17%), mp: 196 °C. ^1^H NMR (500 MHz, D_2_O) δ: 8.52 (s, 1H), 8.44 (s, 1H), 8.16 (s, 1H), 7.38 (s, 1H), 7.21 (s, 1H), 6.09 (m, 1H), 4.83 (br s, 1H), 4.53 (m, 1H), 4.38 (s, 1H), 4.34 (m, 2H), 4.07 (m, 2H), 3.69 (br s, 2H), 2.31 (m, 2H). ^13^C NMR (125 MHz, D_2_O) δ: 157.11, 155.47, 142.36, 142.20, 137.55, 124.24, 122.97, 89.75, 87.44, 87.38, 77.29, 73.54, 66.68, 49.91, 40.62, 30.91. ^31^P NMR (202 MHz, D_2_O) δ: 2.27. LC/ESI-MS (*m*/*z*): positive mode 456.1390 [M + H]^+^ and negative mode 454.1248 [M + H]^−^. Purity by HPLC-UV (254 nm)-ESI-MS: 98.7%.

#### ((2*R*,3*S*,4*R*,5*R*)-3,4-Dihydroxy-5-(6-((4-phenylbutyl)­amino)-9*H*-purin-9-yl)­tetrahydrofuran-2-yl)­methyl dihydrogen Phosphate (**8r**)

The compound was synthesized using **7r** (100 mg, 0.25 mmol) and afforded a white powder (46 mg, 38%), mp: 170.0–172.0 °C. ^1^H NMR (DMSO-*d*_6_) δ: 8.45 (s, 1H), 8.20 (s, 1H), 7.78 (s, 1H), 7.25 (t, *J* = 7.5 Hz, 2H), 7.21–7.17 (m, 2H), 7.16–7.12 (m, 1H), 5.92 (d, *J* = 6.1 Hz, 1H), 4.66 (t, *J* = 5.5 Hz, 3H), 4.24 (dd, *J* = 4.9, 3.0 Hz, 2H), 4.04 (q, *J* = 3.6 Hz, 1H), 3.82 (dd, *J* = 6.8, 3.8 Hz, 3H), 3.51 (s, 2H), 2.63–2.57 (m, 2H), 1.71–1.53 (m, 4H). ^13^C NMR (DMSO-*d*_6_) δ: 154.50, 152.55, 148.92, 142.20, 139.03, 128.27, 128.15, 125.54, 119.02, 86.54, 84.33, 74.02, 71.19, 64.05, 40.06, 34.87, 28.76, 28.44. ^31^P NMR (DMSO-*d*_6_) δ: 1.18. LC/ESI-MS (*m*/*z*): positive mode 480.300 [M + H]^+^. Purity determined by HPLC-UV (254 nm)-ESIMS: 97.9%.

#### ((2*R*,3*S*,4*R*,5*R*)-3,4-Dihydroxy-5-(6-(methyl­(4-phenylbutyl)­amino)-9*H*-purin-9-yl)­tetrahydrofuran-2-yl)­methyl dihydrogen Phosphate (**8s**)

The compound was synthesized using **7s** (150 mg, 0.36 mmol) and afforded a white powder (83 mg, 47%), mp: 176.8–178.8 °C. ^1^H NMR (DMSO-*d*_6_) δ: 8.42 (s, 1H), 8.21 (s, 1H), 7.24 (t, *J* = 7.6 Hz, 2H), 7.21–7.10 (m, 3H), 5.96 (d, *J* = 6.1 Hz, 1H), 4.63 (t, *J* = 5.5 Hz, 2H), 4.49–3.72 (m, 9H), 3.24 (s, 3H), 2.61 (t, *J* = 7.5 Hz, 2H), 1.72–1.53 (m, 4H). ^13^C NMR (DMSO-*d*_6_) δ: 153.84, 151.96, 150.54, 142.07, 137.98, 128.25, 128.20, 125.60, 119.00, 86.52, 83.93, 73.90, 70.96, 64.37, 34.89, 28.13. ^31^P NMR (DMSO-*d*_6_) δ: 0.98. LC–MS (*m*/*z*): positive mode 494.3 [M + H]^+^. Purity by HPLC-UV (254 nm)-ESI-MS: 98.8%.

#### ((2*R*,3*S*,4*R*,5*R*)-5-(6-(Ethyl­(4-phenylbutyl)-amino)-9*H*-purin-9-yl)-3,4-dihydroxytetrahydro-furan-2-yl)­methyl dihydrogen Phosphate (**8t**)

The compound was synthesized using **7t** (100 mg, 0.23 mmol) and afforded a white solid (30 mg, 28%), mp: 187 °C. ^1^H NMR (500 MHz, D_2_O) δ: 8.33 (s, 1H), 8.06 (s, 1H), 7.09 (m, 5H), 6.03 (d, 1H, *J* = 5.40 Hz), 4.63 (t, 1H, *J* = 5.23 Hz), 4.45 (t, 1H, *J* = 4.52 Hz), 4.33 (s, 1H), 4.09 (br s, 2H), 3.70 (br s, 4H), 2.47 (s, 2H), 1.53 (br s, 4H), 1.11 (t, 3H, *J* = 7.01 Hz). ^13^C NMR (125 MHz, D_2_O) δ: 156.13, 154.81, 152.07, 145.29, 140.38, 131.14, 131.09, 128.50, 121.39, 89.67, 86.63, 77.18, 73.10, 66.98, 51.21, 47.06, 37.63, 30.43, 29.52, 15.41. ^31^P NMR (202 MHz, D_2_O) δ: 1.34. LC/ESI-MS (*m*/*z*): positive mode 508.1940 [M + H]^+^ and negative mode 506.1846 [M + H]^−^. Purity by HPLC-UV (254 nm)-ESI-MS: 97.9%.

#### ((2*R*,3*S*,4*R*,5*R*)-3,4-Dihydroxy-5-(6-((4-phenylbutyl)­(propyl)­amino)-9*H*-purin-9-yl)­tetrahydrofuran-2-yl)­methyl dihydrogen Phosphate (**8u**)

The compound was synthesized using **7u** (150 mg 0.34 mmol) and afforded a white solid (59 mg, 33%), mp: 178.5–180.5 °C. ^1^H NMR (DMSO-*d*_6_) δ: 8.41 (s, 1H), 8.20 (s, 1H), 7.32–7.16 (m, 5H), 5.95 (d, *J* = 6.2 Hz, 1H), 4.69–4.61 (m, 1H), 4.21 (dd, *J* = 4.9, 2.9 Hz, 2H), 4.05 (d, *J* = 3.5 Hz, 3H), 3.87 (dd, *J* = 6.6, 3.9 Hz, 3H), 3.70 (s, 4H), 2.61 (t, *J* = 7.3 Hz, 2H), 1.78–1.49 (m, 6H), 0.87 (t, *J* = 7.3 Hz, 3H). ^13^C NMR (DMSO-*d*_6_) δ: 153.50, 151.97, 150.61, 142.09, 138.07, 128.28, 128.18, 125.59, 118.79, 86.44, 84.02, 73.86, 71.09, 64.42, 34.92, 28.21, 11.01. ^31^P NMR (DMSO-*d*_6_) δ: 0.99. LC–MS (*m*/*z*): positive mode 522.4 [M + H]^+^. Purity by HPLC-UV (254 nm)-ESI-MS: 99.1%.

#### ((2*R*,3*S*,4*R*,5*R*)-5-(6-(Butyl­(4-phenylbutyl)­amino)-9*H*-purin-9-yl)-3,4-dihydroxytetrahydrofuran-2-yl)­methyl dihydrogen Phosphate (**8v**)

The compound was synthesized using **7v** (120 mg, 0.26 mmol) and afforded a white solid (72 mg, 52%), mp: 199.0–201.0 °C. ^1^H NMR (DMSO-*d*_6_) δ: 8.44 (s, 1H), 8.19 (s, 1H), 7.36–7.01 (m, 5H), 5.94 (d, *J* = 6.3 Hz, 1H), 4.68 (dd, *J* = 6.4, 4.8 Hz, 1H), 4.23 (dd, *J* = 4.8, 2.7 Hz, 3H), 4.03 (q, *J* = 3.4 Hz, 5H), 3.86–3.77 (m, 4H), 2.61 (t, *J* = 7.2 Hz, 2H), 1.73–1.52 (m, 6H), 1.31 (q, *J* = 7.5 Hz, 2H), 0.89 (t, *J* = 7.3 Hz, 3H). ^13^C NMR (DMSO-*d*_6_) δ: 153.48, 151.94, 150.65, 142.07, 138.12, 128.27, 125.58, 118.75, 86.37, 84.39, 74.00, 71.32, 64.11, 34.89, 28.19, 19.50, 13.84. ^31^P NMR (DMSO-*d*_6_) δ: 1.19. LC–MS (*m*/*z*): positive mode 536.5 [M + H]^+^. Purity by HPLC-UV (254 nm)-ESI-MS: 99.8%.

#### ((2*R*,3*S*,4*R*,5*R*)-5-(6-(Bis­(4-phenylbutyl)­amino)-9*H*-purin-9-yl)-3,4-dihydroxytetrahydrofuran-2-yl)­methyl dihydrogen Phosphate (**8w**)

The compound was synthesized using **7w** (100 mg, 0.19 mmol) and afforded a white solid (7 mg, 6%), mp: 121.0–123.0 °C. ^1^H NMR (DMSO-*d*_6_) δ: 8.44 (s, 1H), 8.19 (s, 1H), 7.25 (t, *J* = 7.5 Hz, 4H), 7.21–7.11 (m, 6H), 5.94 (d, *J* = 6.2 Hz, 1H), 4.65 (dd, *J* = 6.1, 4.9 Hz, 1H), 4.22 (dd, *J* = 4.8, 2.9 Hz, 2H), 4.03 (q, *J* = 3.6 Hz, 2H), 3.83 (dt, *J* = 6.3, 3.2 Hz, 4H), 3.68 (s, 4H), 2.59 (t, *J* = 7.2 Hz, 4H), 1.77–1.50 (m, 8H). ^13^C NMR (DMSO-*d*_6_) δ: 153.45, 151.94, 150.61, 142.07, 138.16, 128.27, 128.18, 125.59, 118.76, 86.47, 84.25, 74.02, 71.27, 64.16, 45.22, 40.06, 34.91, 28.15. ^31^P NMR (DMSO-*d*_6_) δ: 1.18. LC–MS (*m*/*z*): positive mode 612.7 [M + H]^+^. Purity by HPLC-UV (254 nm)-ESI-MS: 99.2%.

#### ((2*R*,3*S*,4*R*,5*R*)-3,4-Dihydroxy-5-(6-((3-phenylpropyl)­amino)-9*H*-purin-9-yl)­tetrahydro-furan-2-yl)­methyl dihydrogen Phosphate (**16a**)

The compound was synthesized using **15a** (200 mg, 0.47 mmol) and afforded a white powder (40 mg, 18%), mp: 142C °C. ^1^H NMR (500 MHz, D_2_O) δ: 8.45 (s, 1H), 8.14 (s, 1H), 7.18 (br s, 4H), 7.10 (br s, 1H), 6.07 (s, 1H), 4.75 (s, 2H), 4.49 (s, 1H), 4.36 (s, 1H), 4.04 (s, 2H), 3.55 (br s, 1H), 2,72 (s, 2H), 2.01 (s, 2H). ^13^C NMR (125 MHz, D_2_O) δ: 157.32, 155.45, 144.86, 141.87, 131.2, 131.18, 128.51, 121.65, 89.52, 87.29, 77.30, 73.44, 72.42, 66.61, 35.28, 32.57. ^31^P NMR (202 MHz, D_2_O) δ: 2.95 LC/ESI-MS (*m*/*z*): positive mode 466.1487 [M + H]^+^ and negative mode 464.1360 [M – H]^−^. Purity by HPLC-UV (254 nm)-ESI-MS: 97.6%.

#### ((2*R*,3*S*,4*R*,5*R*)-3,4-Dihydroxy-5-(6-((3-(3-methoxyphenyl)­propyl)­amino)-9*H*-purin-9-yl)­tetra-hydrofuran-2-yl)­methyl dihydrogen Phosphate (**16b**)

The compound was synthesized using **15b** (250 mg, 0.60 mmol) and afforded a white solid (100 mg, 34%). ^1^H NMR (600 MHz, D_2_O) δ: 8.50 (s, 1H), 8.27 (s, 1H), 7.08 (s, 1H), 6.81–6.57 (m, 3H), 6.13 (d, 1H, *J* = 4.38 Hz), 4.75 (t, 1H, *J* = 5.37 Hz), 4.51 (t, 1H, *J* = 3.89 Hz), 4.40 (br s, 1H), 4.15 (br m, 2H), 3.69 (s, 3H), 3.60 (m, 2H), 2.76 (br s, 2H), 2.15 (br t, 2H, *J* = 6.21 Hz). ^13^C NMR (125 MHz, D_2_O) δ: 165.75, 161.45, 149.40, 146.08, 143.59, 132.50, 123.95, 120.15, 118.21, 116.69, 113.61, 90.35, 87.16, 77.52, 73.26, 67.21, 57.89, 44.31, 35.28, 31.03. ^31^P NMR (202 MHz, D_2_O) δ: 0.41. LC/ESI-MS (*m*/*z*): positive mode 496.1557 [M + H]^+^ and negative mode 494.1417 [M – H]^−^. Purity by HPLC-UV (254 nm)-ESI-MS: 96.5%.

#### ((2*R*,3*S*,4*R*,5*R*)-3,4-Dihydroxy-5-(6-((3-(4-methoxyphenyl)­propyl)­amino)-9*H*-purin-9-yl)­tetra-hydrofuran-2-yl)­methyl dihydrogen Phosphate (**16c**)

The compound was synthesized using **15c** (200 mg, 0.44 mmol) and afforded a white solid (20 mg, 10%), mp: 95 °C. ^1^H NMR (600 MHz, D_2_O) δ: 8.43 (s, 1H), 8.11 (s, 1H), 7.05 (d, 2H, *J* = 7.81 Hz), 6.71 (d, 2H, *J* = 7.81 Hz), 6.05 (d, 1H, *J* = 5.80 Hz), 4.72 (t, 1H, *J* = 5.76 Hz), 4.49 (t, 1H, *J* = 4.43 Hz), 4.35 (br s, 1H), 4.05 (br s, 2H), 3.70 (s, 3H), 3.60 (m, 2H), 2.65 (t, 2H, *J* = 7.06 Hz), 1.98 (m, 2H). ^13^C NMR (125 MHz, D_2_O) δ: 159.42, 157.14, 155.40, 146.53, 141.70, 137.34, 132.21, 132.01, 116.39, 89.65, 86.97, 77.29, 73.28, 66.95, 58.15, 43.14, 34.52, 32.63. ^31^P NMR (202 MHz, D_2_O) δ: 1.21. LC/ESI-MS (*m*/*z*): positive mode 496.1572 [M + H]^+^ and negative mode 494.1434 [M – H]^−^. Purity by HPLC-UV (254 nm)-ESIMS: 95.0%.

#### ((2*R*,3*S*,4*R*,5*R*)-5-(6-((6-Benzamidohexyl)-amino)-9*H*-purin-9-yl)-3,4-dihydroxytetrahydro-furan-2-yl)­methyl dihydrogen Phosphate (**16d**)

The compound was synthesized using **15d** (180 mg, 0.35 mmol) and afforded a white powder (30 mg, 15%), mp: 111 °C. ^1^H NMR (500 MHz, D_2_O) δ: 8.38 (s, 1H), 8.12 (s, 1H), 7.60 (d, 2H, *J* = 8.23 Hz), 7.49 (t, 1H, *J* = 7.44 Hz), 7.37 (t, 2H, *J* = 7.37 Hz), 6.07 (d, 1H, *J* = 5.80 Hz), 4.71 (t, 1H, *J* = 5.43 Hz), 4.48 (m, 1H), 4.38 (br s, 1H), 4.12 (m, 2H), 3.50 (br s, 2H), 3.37 (t, 2H, *J* = 6.62 Hz), 1.69 (m, 2H), 1.63 (m, 2H), 1.44 (m, 4H). ^13^C NMR (125 MHz, D_2_O) δ: 173.54, 157.23, 155.39, 155.35, 141.78, 136.45, 134.68, 131.38, 129.57, 89.69, 89.92, 86.86, 77.23, 73.32, 67.11, 42.57, 42.56, 30.93, 30.75, 28.42, 28.34. ^31^P NMR (202 MHz, D_2_O) δ: 0.86. LC/ESI-MS (*m*/*z*): positive mode 551.2001 [M + H]^+^ and negative mode 549.1846 [M + H]^−^. Purity by HPLC-UV (254 nm)-ESI-MS: 98.1%.

#### ((2*R*,3*S*,4*R*,5*R*)-3,4-Dihydroxy-5-(6-(4-phenylbutoxy)-9*H*-purin-9-yl)­tetrahydrofuran-2-yl)­methyl dihydrogen Phosphate (**21a**)

The compound was synthesized using **20a** (100 mg, 0.25 mmol) and afforded a white solid (10 mg, 7%), mp: 180 °C. ^1^H NMR (500 MHz, D_2_O) δ: 8.67 (s, 1H), 8.39 (s, 1H), 7.15 (m, 5H), 6.19 (d, 1H, *J* = 5.77 Hz), 4.53 (m, 4H, overlapping peaks: 2x C*H*OH & C*H*CH_2_), 4.39 (br s, 1H), 4.08 (m, 2H), 2.66 (t, 2H, *J* = 7.05 Hz), 1.89 (m, 4H). ^13^C NMR (125 MHz, D_2_O) δ: 163.99, 154.84, 154.11, 145.56, 144.63, 131.31, 131.12, 128.51, 123.63, 90.02, 87.37, 77.38, 73.45, 70.80, 66.70, 37.31, 30.18, 29.37. ^31^P NMR (202 MHz, D_2_O) δ: 2.56. LC/ESI-MS (*m*/*z*): positive mode 481.1477 [M + H]^+^ and negative mode 479.1366 [M – H]^−^. Purity by HPLC-UV (254 nm)-ESI-MS: 96.0%.

#### ((2*R*,3*S*,4*R*,5*R*)-5-(6-(Butylthio)-9*H*-purin-9-yl)-3,4-dihydroxytetrahydrofuran-2-yl)­methyl dihydrogen Phosphate (**21b**)

The compound was synthesized using **20b** (100 mg, 0.29 mmol) and afforded a white powder (79 mg, 65%), mp: 192.0–193.5 °C. ^1^H NMR (DMSO-*d*_6_) δ: 8.80 (s, 1H), 8.71 (s, 1H), 6.01 (d, *J* = 6.1 Hz, 1H), 5.07 (s, 3H), 4.73 (dd, *J* = 6.2, 4.7 Hz, 2H), 4.26 (dd, *J* = 4.9, 2.7 Hz, 1H), 4.08 (q, *J* = 3.5 Hz, 1H), 3.90–3.77 (m, 2H), 3.41–3.27 (m, 2H), 1.78–1.64 (m, 2H), 1.44 (h, *J* = 7.3 Hz, 2H), 0.92 (t, *J* = 7.4 Hz, 3H). ^13^C NMR (126 MHz, DMSO) δ: 159.72, 151.54, 148.54, 142.96, 130.79, 86.90, 84.82, 74.23, 71.25, 63.94, 31.17, 27.50, 21.34, 13.46. ^31^P NMR (DMSO-*d*_6_) δ: 1.18 (d, *J* = 7.5 Hz). LC–MS (*m*/*z*): positive mode 421.2 [M + H]^+^. Purity by HPLC-UV (254 nm)-ESI-MS: 99.5%.

#### ((2*R*,3*S*,4*R*,5*R*)-5-(6-(Cyclohexylthio)-9*H*-purin-9-yl)-3,4-dihydroxytetrahydrofuran-2-yl)­methyl dihydrogen Phosphate (**21c**)

The compound was synthesized using **20c** (100 mg, 0.27 mmol) and afforded a white powder (93 mg, 77%), mp: 191.0–193.0 °C. ^1^H NMR (DMSO-*d*_6_) δ: 8.78 (s, 1H), 8.71 (s, 1H), 6.01 (d, *J* = 6.2 Hz, 1H), 4.72 (dd, *J* = 6.2, 4.8 Hz, 1H), 4.27–4.16 (m, 4H), 4.07 (q, *J* = 3.4 Hz, 2H), 3.88–3.79 (m, 3H), 2.14–2.02 (m, 2H), 1.74 (dt, *J* = 13.0, 4.3 Hz, 2H), 1.64–1.27 (m, 6H). ^13^C NMR (DMSO-*d*_6_) δ: 159.54, 151.60, 148.65, 142.95, 130.60, 86.89, 84.75, 74.23, 71.24, 63.95, 40.97, 32.75, 32.70, 25.43, 25.11. ^31^P NMR (DMSO-*d*_6_) δ: 1.06 (d, *J* = 7.2 Hz). LC–MS (*m*/*z*): positive mode 447.2 [M + H]^+^. Purity by HPLC-UV (254 nm)-ESI-MS: 99.9%.

#### ((2*R*,3*S*,4*R*,5*R*)-5-(6-Amino-8-(methylamino)-9*H*-purin-9-yl)-3,4-dihydroxytetra-hydrofuran-2-yl)­methyl dihydrogen Phosphate (**25a**)

The compound was synthesized using **24a** (100 mg, 0.34 mmol) and afforded a white powder (30 mg, 23%), mp: 197 °C. ^1^H NMR (600 MHz, D_2_O) δ: 8.05 (s, 1H), 6.08 (d, 1H, *J* = 7.86 Hz), 4.69 (dd, 1H, *J* = 5.93 7.69 Hz), 4.44 (dd, 1H, *J* = 2.04, 5.73 Hz), 4.36 (br s, 1H), 4.17 (d m, 2H), 3.06 (s, 3H). ^13^C NMR (150 MHz, D_2_O) δ: 155.87, 153.59, 152.44, 151.33, 118.88, 89.16, 87.31, 73.31, 73.08, 67.50, 31.80. ^31^P NMR (202 MHz, D_2_O) δ: 0.21. LC/ESI-MS (*m*/*z*): positive mode 377.0966 [M + H]^+^ and negative mode 375.0813 [M – H]^−^. Purity determined by HPLC-UV (254 nm)-ESI-MS: 99.5%.

#### ((2*R*,3*S*,4*R*,5*R*)-5-(6-Amino-8-(butylamino)-9*H*-purin-9-yl)-3,4-dihydroxytetrahydrofuran-2-yl)­methyl dihydrogen Phosphate (**25b**)

The compound was synthesized using **24b** (100 mg, 0.30 mmol) and afforded a white powder (5 mg, 4%), mp: 180 °C. ^1^H NMR (500 MHz, D_2_O) δ: 7.99 (d, 1H, *J* = 1.08 Hz), 6.00 (d, 1H, *J* = 7.70 Hz), 4.73 (dd, 1H, *J* = 5.94, 7.82 Hz), 4.45 (dd, 1H, *J* = 2.50, 5.86 Hz), 4.33 (t, 1H, *J* = 2.41 Hz), 4.13 (m, 2H), 3.45 (m, 2H), 1.65 (m, 2H), 1.37 (q, 2H, *J* = 7.51 Hz), 0.91 (m, 3H). ^13^C NMR (125 MHz, D_2_O) δ: 155.20, 153.89, 152.36, 151.55, 119.14, 89.14, 87.33, 73.34, 73.02, 67.33, 45.24, 33.52, 22.37, 16.12. ^31^P NMR (202 MHz, D_2_O) δ: 1.14. LC/ESI-MS (*m*/*z*): positive mode 419.1438 [M + H]^+^ and negative mode 417.1295 [M – H]^−^. Purity determined by HPLC-UV (254 nm)-ESI-MS: 100%.

#### ((2*R*,3*S*,4*R*,5*R*)-5-(6-Amino-8-(cyclopropylamino)-9*H*-purin-9-yl)-3,4-dihydroxytetrahydrofuran-2-yl)­methyl dihydrogen Phosphate (**25c**)

The compound was synthesized using **24c** (100 mg, 0.31 mmol) and afforded a white powder (26 mg, 21%), mp: 163.0–165.0 °C. ^1^H NMR (DMSO-*d*_6_) δ: 7.90 (s, 1H), 6.68 (s, 1H), 6.46 (s, 2H), 5.77 (d, *J* = 6.6 Hz, 1H), 4.76 (t, *J* = 6.1 Hz, 1H), 4.26 (dd, *J* = 5.6, 3.3 Hz, 2H), 3.94 (q, *J* = 3.9 Hz, 2H), 3.85–3.77 (m, 4H), 2.80–2.71 (m, 1H), 0.91–0.76 (m, 1H), 0.70–0.51 (m, 3H). ^13^C NMR (DMSO-*d*_6_) δ: 152.44, 151.90, 150.05, 148.80, 117.07, 86.11, 83.58, 70.39, 70.14, 64.40, 25.24, 6.84, 6.13. ^31^P NMR (DMSO-*d*_6_) δ: 0.85. LC–MS (*m*/*z*): positive mode 403.4 [M + H]^+^. Purity by HPLC-UV (254 nm)-ESI-MS: 95.6%.

#### ((2*R*,3*S*,4*R*,5*R*)-5-(6-Amino-8-((4-phenyl-butyl)­amino)-9*H*-purin-9-yl)-3,4-dihydroxytetra-hydrofuran-2-yl)­methyl dihydrogen Phosphate (**25d**)

The compound was synthesized using **24d** (270 mg, 0.63 mmol) and afforded a white solid (10 mg, 3%), mp: decomposition >170 °C. ^1^H NMR (500 MHz, D_2_O) δ: 8.03 (s, 1H), 7.16 (m, 4H), 7.05 (t, 1H, *J* = 6.60 Hz), 5.99 (d, 1H, *J* = 7.64 Hz), 4.67 (m, 1H), 4.43 (dd, 1H, *J* = 2.01, 5.65 Hz), 4.33 (s, 1H), 4.15 (d m, 2H), 3.46 (d m, 2H), 2.58 (m, 2H), 1.66 (m, 4H). ^13^C NMR (125 MHz, D_2_O) δ: 154.83, 152.86, 152.03, 150.36, 145.74, 131.34, 131.10, 128.41, 118.79, 89.23, 87.25, 73.55, 73.04, 67.56, 45.01, 37.51, 30.41, 29.88. ^31^P NMR (202 MHz, D_2_O) δ: 0.36. LC/ESI-MS (*m*/*z*): positive mode 495.1741 [M + H]^+^ and negative mode 493.1621 [M – H]^−^. Purity by HPLC-UV (254 nm)-ESI-MS: 98.7%.

#### ((2*R*,3*S*,4*R*,5*R*)-5-(6-Amino-8-hydroxy-9*H*-purin-9-yl)-3,4-dihydroxytetrahydrofuran-2-yl)­methyl dihydrogen Phosphate (**27a**), CAS: 25030–04–0

The compound was synthesized using **26a** (120 mg, 0.40 mmol) and afforded a white powder (19 mg, 13%), mp: 258–260 °C. ^1^H NMR (DMSO-*d*_6_) δ: 10.32 (s, 1H), 8.03 (s, 1H), 6.48 (s, 2H), 5.68 (d, *J* = 5.2 Hz, 1H), 5.27 (s, 2H), 4.90 (t, *J* = 5.2 Hz, 1H), 4.24 (t, *J* = 5.0 Hz, 1H), 4.18–3.99 (m, 2H), 3.98–3.91 (m, 1H), 3.89–3.71 (m, 2H). ^13^C NMR (DMSO-*d*_6_) δ: 151.38, 150.85, 147.01, 146.74, 103.41, 85.78, 82.06, 70.43, 69.90, 65.81. ^31^P NMR (DMSO-*d*_6_) δ: −0.13. LC–MS (*m*/*z*): positive mode 364.10 [M + H]^+^. Purity by HPLC-UV (254 nm)-ESI-MS: 100%.

#### ((2*R*,3*S*,4*R*,5*R*)-5-(6-Amino-8-butoxy-9*H*-purin-9-yl)-3,4-dihydroxytetrahydrofuran-2-yl)­methyl dihydrogen Phosphate (**27b**)

The compound was synthesized using **26b** (100 mg, 0.29 mmol) and afforded a white powder (55 mg, 45%), mp: 236.0–238.0 °C. ^1^H NMR (DMSO-*d*_6_) δ: 8.04 (s, 1H), 6.81 (s, 2H), 5.71 (d, *J* = 5.6 Hz, 1H), 4.91 (t, *J* = 5.5 Hz, 1H), 4.54–4.41 (m, 3H), 4.21 (dd, *J* = 5.5, 4.2 Hz, 2H), 4.02–3.82 (m, 3H), 3.75–3.55 (m, 2H), 1.86–1.72 (m, 2H), 1.43 (h, *J* = 7.4 Hz, 2H), 0.94 (t, *J* = 7.4 Hz, 3H). ^13^C NMR (DMSO-*d*_6_) δ: 153.88, 153.71, 150.78, 149.11, 114.67, 86.46, 83.09, 70.80, 70.56, 69.80, 64.46, 30.19, 18.50, 13.51. ^31^P NMR (DMSO-*d*_6_) δ: 0.76. LC–MS (*m*/*z*): positive mode 420.20 [M + H]^+^. Purity by HPLC-UV (254 nm)-ESI-MS: 98.7%.

#### ((2*R*,3*S*,4*R*,5*R*)-5-(6-Amino-8-(cyclopentyloxy)-9*H*-purin-9-yl)-3,4-dihydroxytetrahydrofuran-2-yl)­methyl dihydrogen Phosphate (**27c**)

The compound was synthesized using **26c** (80 mg, 0.23 mmol) and afforded a white powder (17 mg, 17%), mp: 96.0–98.0 °C. ^1^H NMR (DMSO-*d*_6_) δ: 8.03 (s, 1H), 6.77 (s, 2H), 5.69 (d, *J* = 5.3 Hz, 1H), 5.50–5.41 (m, 1H), 4.81 (t, *J* = 5.4 Hz, 1H), 4.18 (t, *J* = 5.0 Hz, 2H), 3.92 (q, *J* = 5.8 Hz, 2H), 3.87–3.82 (m, 3H), 3.66–3.61 (m, 1H), 2.02–1.84 (m, 4H), 1.81–1.70 (m, 2H), 1.67–1.56 (m, 2H). ^13^C NMR (DMSO-*d*_6_) δ: 153.67, 153.37, 150.74, 149.09, 114.78, 86.44, 82.98, 82.91, 70.94, 70.74, 64.51, 32.30, 32.22, 23.16, 23.13. ^31^P NMR (DMSO-*d*_6_) δ: 0.60. LC–MS (*m*/*z*): positive mode 432.1 [M + H]^+^. Purity by HPLC-UV (254 nm)-ESI-MS: 98.1%.

#### ((2*R*,3*S*,4*R*,5*R*)-5-(6-Amino-8-chloro-9*H*-purin-9-yl)-3,4-dihydroxy-tetrahydrofuran-2-yl)-methyl dihydrogen Phosphate (**28**)

The compound was synthesized using **22** (100 mg, 0.33 mmol) and afforded a white powder (90 mg, 69%), mp: 150 °C. ^1^H NMR (600 MHz, D_2_O) δ: 8.23 (s, 1H), 6.12 (d, 1H, *J* = 5.47 Hz), 5.25 (br s, 1H), 4.59 (br s, 1H), 4.29 (br s, 1H), 4.12 (d m, 2H). ^13^C NMR (150 MHz, D_2_O) δ: 157.21, 155.79, 152.85, 141.97, 120.49, 91.06, 86.56, 73.64, 72.50, 61.75. ^31^P NMR (202 MHz, D_2_O) δ: 1.76. LC/ESI-MS (*m*/*z*): positive mode 382.0383 [M + H]^+^ and negative mode 380.0162 [M – H]^−^. Purity determined by HPLC-UV (254 nm)-ESI-MS: 100%.

#### ((2*R*,3*S*,4*R*,5*R*)-5-(6-Amino-8-(methylthio)-9*H*-purin-9-yl)-3,4-dihydroxytetrahydrofuran-2-yl)­methyl dihydrogen Phosphate (**31a**)

The compound was synthesized using **30a** (100 mg, 0.32 mmol) and afforded a white solid (100 mg, 81%), mp: 145 °C. ^1^H NMR (600 MHz, D_2_O) δ: 8.16 (s, 1H), 6.06 (d, 1H, *J* = 4.75 Hz), 5.13 (br s, 1H), 4.52 (br s, 1H), 4.27 (br s, 1H), 4.13 (d, 2H, *J* = 20.08 Hz), 2.74 (br s, 3H). ^13^C NMR (150 MHz, D_2_O) δ: 156.34, 155.56, 154.51, 153.90, 121.76, 90.56, 86.69, 73.41, 72.57, 66.87, 17.23. ^31^P NMR (202 MHz, D_2_O) δ: 2.78 LC/ESI-MS (*m*/*z*): positive mode 394.0582 [M + H]^+^ and negative mode 392.0446 [M – H]^−^. Purity by HPLC-UV (254 nm)-ESI-MS: 100%.

#### ((2*R*,3*S*,4*R*,5*R*)-5-(6-Amino-8-(ethylthio)-9*H*-purin-9-yl)-3,4-dihydroxytetrahydrofuran-2-yl)­methyl dihydrogen Phosphate (**31b**), CAS: 81609–36–1

The compound was synthesized using **30b** (100 mg, 0.31 mmol) and afforded a white powder (7 mg, 6%), mp: 166.0–168.0 °C. ^1^H NMR (D_2_O) δ: 8.35 (d, *J* = 1.0 Hz, 1H), 6.07 (dd, *J* = 5.7, 0.9 Hz, 1H), 5.19–5.13 (m, 1H), 4.82–4.79 (m, 1H), 4.79 (s, 3H), 4.60 (t, *J* = 5.1 Hz, 1H), 4.29 (q, *J* = 4.7 Hz, 1H), 4.24–4.12 (m, 2H), 3.41–3.27 (m, 2H), 1.45–1.39 (m, 3H). ^13^C NMR (D_2_O) δ: 157.78, 152.91, 150.82, 146.17, 121.89, 91.57, 86.75, 74.34, 72.64, 67.29, 30.06, 16.59. ^31^P NMR (D_2_O) δ: 0.57. LC–MS (*m*/*z*): positive mode 408.20 [M + H]^+^. Purity by HPLC-UV (254 nm)-ESI-MS: 99.9%.

#### ((2*R*,3*S*,4*R*,5*R*)-5-(6-Amino-8-(*tert*-butylthio)-9*H*-purin-9-yl)-3,4-dihydroxytetrahydrofuran-2-yl)­methyl dihydrogen Phosphate (**31c**)

The compound was synthesized using **30c** (100 mg, 0.28 mmol) and afforded a white powder (64 mg, 52%), mp: >300 °C. ^1^H NMR (DMSO-*d*_6_) δ: 8.14 (s, 1H), 7.31 (s, 2H), 6.02 (d, *J* = 5.6 Hz, 1H), 5.17 (t, *J* = 5.5 Hz, 2H), 4.34 (dd, *J* = 5.6, 3.8 Hz, 2H), 4.10–3.90 (m, 3H), 3.78–3.59 (m, 2H), 1.46 (s, 9H). ^13^C NMR (DMSO-*d*_6_) δ: 155.23, 152.55, 150.06, 145.73, 119.66, 89.05, 83.47, 70.92, 70.58, 64.42, 50.23, 30.82. ^31^P NMR (DMSO-*d*_6_) δ: 0.81. LC–MS (*m*/*z*): positive mode 436.4 [M + H]^+^. Purity by HPLC-UV (254 nm)-ESI-MS: 98.9%.

#### ((2*R*,3*S*,4*R*,5*R*)-5-(6-Amino-8-(propylthio)-9*H*-purin-9-yl)-3,4-dihydroxytetrahydrofuran-2-yl)­methyl dihydrogen Phosphate (**31d**)

The compound was synthesized using **30d** (100 mg, 0.29 mmol) and afforded a white powder (24 mg, 20%), mp: 76.0–78.0 °C. ^1^H NMR (DMSO-*d*_6_) δ: 8.09 (s, 1H), 7.10 (s, 2H), 5.77 (d, *J* = 5.8 Hz, 1H), 5.10 (t, *J* = 5.7 Hz, 3H), 4.28 (dd, *J* = 5.6, 3.9 Hz, 2H), 4.09–3.83 (m, 3H), 3.74–3.64 (m, 1H), 3.37–3.18 (m, 2H), 1.73 (h, *J* = 7.3 Hz, 2H), 0.99 (t, *J* = 7.4 Hz, 3H). ^13^C NMR (DMSO-*d*_6_) δ: 154.24, 151.65, 151.11, 148.60, 119.26, 88.51, 83.56, 70.80, 70.42, 64.29, 34.05, 22.26, 13.04. ^31^P NMR (DMSO-*d*_6_) δ: 1.01–0.72 (m). LC–MS (*m*/*z*): positive mode 422.2 [M + H]^+^. Purity by HPLC-UV (254 nm)-ESI-MS: 98.5%.

#### ((2*R*,3*S*,4*R*,5*R*)-5-(6-Amino-8-(isopentylthio)-9*H*-purin-9-yl)-3,4-dihydroxytetrahydrofuran-2-yl)­methyl dihydrogen Phosphate (**31e**)

The compound was synthesized using **30e** (100 mg, 0.27 mmol) and afforded a white powder (49 mg, 40%), mp: 72.0–74.0 °C. ^1^H NMR (DMSO-*d*_6_) δ: 8.09 (s, 1H), 7.10 (s, 2H), 5.75 (d, *J* = 5.9 Hz, 1H), 5.11 (t, *J* = 5.7 Hz, 2H), 4.27 (dd, *J* = 5.5, 3.8 Hz, 2H), 4.07–3.87 (m, 3H), 3.75–3.63 (m, 2H), 3.35–3.24 (m, 2H), 1.76–1.65 (m, 1H), 1.65–1.55 (m, 2H), 0.91 (d, *J* = 6.6 Hz, 6H). ^13^C NMR (DMSO-*d*_6_) δ: 154.27, 151.69, 151.11, 148.64, 119.31, 88.51, 83.55, 70.77, 70.37, 64.35, 45.25, 37.69, 30.37, 26.86, 22.07. ^31^P NMR (DMSO-*d*_6_) δ: 0.78. LC–MS (*m*/*z*): positive mode 450.3 [M + H]^+^. Purity by HPLC-UV (254 nm)-ESI-MS: 99.5%.

#### ((2*R*,3*S*,4*R*,5*R*)-5-(6-Amino-8-((2-methylbutyl)­thio)-9*H*-purin-9-yl)-3,4-dihydroxytetrahydrofuran-2-yl)­methyl dihydrogen Phosphate (**31f**)

The compound was synthesized using **30f** (100 mg, 0.27 mmol) and afforded a white powder (84 mg, 62%), mp: 168.0–170.0 °C. ^1^H NMR (DMSO-*d*_6_) δ: 8.09 (s, 1H), 7.08 (s, 2H), 5.76 (d, *J* = 5.9 Hz, 1H), 5.13 (t, *J* = 5.7 Hz, 1H), 4.28 (dd, *J* = 5.6, 3.8 Hz, 4H), 4.04–3.89 (m, 3H), 3.73–3.65 (m, 1H), 3.33 (dd, *J* = 12.7, 6.0 Hz, 1H), 3.23 (dd, *J* = 12.7, 7.1 Hz, 1H), 1.83–1.71 (m, 1H), 1.56–1.44 (m, 1H), 1.33–1.20 (m, 1H), 0.99 (d, *J* = 6.9 Hz, 3H), 0.89 (t, *J* = 7.4 Hz, 3H). ^13^C NMR (DMSO-*d*_6_) δ: 154.20, 151.63, 151.12, 148.97, 119.24, 88.56, 83.53, 70.81, 70.38, 64.28, 38.79, 34.08, 27.90, 18.47, 11.04. ^31^P NMR (DMSO-*d*_6_) δ: 0.77 (d, *J* = 9.5 Hz). LC–MS (*m*/*z*): positive mode 450.3 [M + H]^+^. Purity by HPLC-UV (254 nm)-ESI-MS: 97.9%.

#### ((2*R*,3*S*,4*R*,5*R*)-5-(6-Amino-8-(pentylthio)-9*H*-purin-9-yl)-3,4-dihydroxytetrahydrofuran-2-yl)­methyl dihydrogen Phosphate (**31g**)

The compound was synthesized using **30g** (100 mg, 0.27 mmol) and afforded a white powder (74 mg, 61%), mp: 75.0–77.0 °C. ^1^H NMR (DMSO-*d*_6_) δ: 8.09 (s, 1H), 7.10 (s, 2H), 5.75 (d, *J* = 5.8 Hz, 1H), 5.10 (t, *J* = 5.7 Hz, 1H), 4.28 (dd, *J* = 5.5, 3.9 Hz, 2H), 4.05–3.84 (m, 4H), 3.73–3.62 (m, 2H), 3.35–3.23 (m, 2H), 1.71 (p, *J* = 7.3 Hz, 2H), 1.44–1.25 (m, 4H), 0.87 (t, *J* = 7.2 Hz, 3H). ^13^C NMR (DMSO-*d*_6_) δ: 154.25, 151.67, 151.12, 148.68, 119.29, 88.52, 83.57, 70.83, 70.42, 64.31, 32.12, 30.24, 28.51, 21.60, 13.81. ^31^P NMR (DMSO-*d*_6_) δ: 0.82. LC–MS (*m*/*z*): positive mode 450.3 [M + H]^+^. Purity by HPLC-UV (254 nm)-ESI-MS: 99.7%.

#### ((2*R*,3*S*,4*R*,5*R*)-5-(6-Amino-8-((5-methyl-hexyl)­thio)-9*H*-purin-9-yl)-3,4-dihydroxytetra-hydrofuran-2-yl)­methyl dihydrogen Phosphate (**31h**)

The compound was synthesized using **30h** (100 mg, 0.25 mmol) and afforded a white solid (30 mg, 24%), mp: >180 °C (decomposition). ^1^H NMR (600 MHz, D_2_O) δ: 8.15 (s, 1H), 6.09 (d, *J* = 6.15 Hz, 1H), 5.17 (t, 1H, *J* = 6.13 Hz), 4.54 (m, 1H), 4.26 (q, 1H, *J* = 4.91 Hz), 4.16 (m, 2H), 3.27 (m, 2H), 1.71 (m, 2H), 1.46 (m, 1H), 1.40 (m, 2H), 1.15 (q, 2H, *J* = 7.11 Hz), 0.81 (d, 6H, *J* = 6.60 Hz). ^13^C NMR (150 MHz, D_2_O) δ: 156.46, 154.58, 154.36, 153.54, 121.87, 90.94, 86.44, 73.59, 72.64, 67.30, 40.39, 35.88, 31.63, 29.98, 28.40, 24.67, 21.83. ^31^P NMR (202 MHz, D_2_O) δ: 1.43. LC/ESI-MS (*m*/*z*): positive mode 478.1504 [M + H]^+^ and negative mode 476.1402 [M – H]^−^. Purity by HPLC-UV (254 nm)-ESI-MS: 95.5%.

#### ((2*R*,3*S*,4*R*,5*R*)-5-(6-Amino-8-(cyclopentylthio)-9*H*-purin-9-yl)-3,4-dihydroxytetrahydrofuran-2-yl)­methyl dihydrogen Phosphate (**31i**)

The compound was synthesized using **30i** (80 mg, 0.22 mmol) and afforded a white powder (25 mg, 25%), mp: 118.3–119.5 °C. ^1^H NMR (DMSO-*d*_6_) δ: 8.09 (s, 1H), 7.16 (s, 2H), 5.79 (d, *J* = 5.9 Hz, 1H), 5.14 (t, *J* = 5.7 Hz, 1H), 4.26 (dd, *J* = 5.6, 3.3 Hz, 3H), 4.07–3.96 (m, 4H), 3.78–3.71 (m, 1H), 2.96 (d, *J* = 7.3 Hz, 1H), 2.24–2.11 (m, 2H), 1.81–1.55 (m, 6H). ^13^C NMR (DMSO-*d*_6_) δ: 154.49, 151.86, 150.75, 148.39, 119.47, 88.67, 83.45, 70.66, 70.29, 64.61, 45.11, 33.24, 33.03, 24.26, 24.22. ^31^P NMR (DMSO-*d*_6_) δ: 0.77. LC–MS (*m*/*z*): positive mode 448.0 [M + H]^+^. Purity by HPLC-UV (254 nm)-ESI-MS: 98.2%.

#### ((2*R*,3*S*,4*R*,5*R*)-5-(6-Amino-8-(cyclohexylthio)-9*H*-purin-9-yl)-3,4-dihydroxytetrahydrofuran-2-yl)­methyl dihydrogen Phosphate (**31j**)

The compound was synthesized using **30j** (100 mg, 0.26 mmol) and afforded a white powder (32 mg, 27%), mp: 157.0–159.0 °C. ^1^H NMR (DMSO-*d*_6_) δ: 8.10 (s, 1H), 7.16 (s, 2H), 5.80 (d, *J* = 5.7 Hz, 1H), 5.13 (t, *J* = 5.7 Hz, 2H), 4.29 (dd, *J* = 5.5, 3.9 Hz, 2H), 4.02–3.90 (m, 3H), 3.85–3.76 (m, 1H), 3.71–3.64 (m, 1H), 2.57 (d, *J* = 7.1 Hz, 1H), 2.11–1.96 (m, 2H), 1.78–1.66 (m, 2H), 1.58–1.48 (m, 3H), 1.45–1.25 (m, 3H). ^13^C NMR (DMSO-*d*_6_) δ: 154.49, 151.90, 150.75, 147.50, 119.46, 88.70, 83.55, 70.85, 70.40, 64.33, 46.40, 32.83, 32.66, 25.28, 25.01. ^31^P NMR (DMSO-*d*_6_) δ: −0.37. LC–MS (*m*/*z*): positive mode 462.1 [M + H]^+^. Purity by HPLC-UV (254 nm)-ESI-MS: 99.9%.

#### ((2*R*,3*S*,4*R*,5*R*)-5-(6-Amino-8-((cyclohexylmethyl)­thio)-9*H*-purin-9-yl)-3,4-dihydroxytetrahydrofuran-2-yl)­methyl dihydrogen Phosphate (**31k**)

The compound was synthesized using **30k** (100 mg, 0.25 mmol) and afforded a white powder (26 mg, 21%), mp: 171.0–173.0 °C. ^1^H NMR (DMSO-*d*_6_) δ: 8.08 (s, 1H), 7.11 (s, 2H), 5.76 (d, *J* = 5.8 Hz, 1H), 5.13 (t, *J* = 5.7 Hz, 1H), 4.26 (dd, *J* = 5.5, 3.7 Hz, 2H), 4.11–3.89 (m, 3H), 3.76–3.67 (m, 1H), 3.37–3.15 (m, 2H), 2.75 (s, 1H), 1.83 (ddt, *J* = 13.0, 9.1, 3.6 Hz, 2H), 1.75–1.51 (m, 4H), 1.30–0.90 (m, 6H). ^13^C NMR (DMSO-*d*_6_) δ: 154.21, 151.62, 151.15, 149.02, 119.25, 88.51, 83.52, 70.72, 70.30, 64.43, 45.04, 37.11, 31.83, 25.74, 25.40. ^31^P NMR (DMSO-*d*_6_) δ: 0.77. LC–MS (*m*/*z*): positive mode 476.2 [M + H]^+^. Purity by HPLC-UV (254 nm)-ESI-MS: 100%.

#### ((2*R*,3*S*,4*R*,5*R*)-5-(6-Amino-8-(thiophen-2-ylthio)-9*H*-purin-9-yl)-3,4-dihydroxytetrahydrofuran-2-yl)­methyl dihydrogen Phosphate (**31l**)

The compound was synthesized using **30l** (100 mg, 0.26 mmol) and afforded a white powder (40 mg, 33%), mp: 180.0–182.0 °C. ^1^H NMR (DMSO-*d*_6_) δ: 8.14 (s, 1H), 7.78 (dd, *J* = 5.4, 1.3 Hz, 1H), 7.48 (dd, *J* = 3.8, 1.2 Hz, 1H), 7.28 (s, 2H), 7.12 (dd, *J* = 5.4, 3.7 Hz, 1H), 6.09 (d, *J* = 5.8 Hz, 1H), 5.13 (t, *J* = 5.7 Hz, 1H), 4.33 (dd, *J* = 5.6, 3.9 Hz, 2H), 4.10–3.89 (m, 4H), 3.81–3.66 (m, 2H). ^13^C NMR (DMSO-*d*_6_) δ: 155.16, 152.81, 150.57, 145.68, 135.93, 132.63, 128.08, 126.25, 119.30, 88.97, 83.78, 70.84, 70.79, 64.25. ^31^P NMR (DMSO-*d*_6_) δ: 0.85 (t, *J* = 8.2 Hz). LC–MS (*m*/*z*): positive mode 462.1 [M + H]^+^. Purity by HPLC-UV (254 nm)-ESI-MS: 99.4%.

#### ((2*R*,3*S*,4*R*,5*R*)-5-(6-Amino-8-(phenylthio)-9*H*-purin-9-yl)-3,4-dihydroxytetrahydrofuran-2-yl)­methyl dihydrogen Phosphate (**31m**), CAS: 78710–83–5

The compound was synthesized using **30m** (80 mg, 0.21 mmol) and afforded a white powder (27 mg, 28%), mp: 149.0–151.0 °C. ^1^H NMR (DMSO-*d*_6_) δ: 8.23 (d, *J* = 1.3 Hz, 1H), 7.86 (s, 2H), 7.47–7.42 (m, 2H), 7.42–7.37 (m, 2H), 7.37–7.33 (m, 1H), 6.10 (dd, *J* = 5.6, 1.3 Hz, 1H), 5.50 (s, 2H), 5.18–5.11 (m, 1H), 4.32 (s, 1H), 4.19–4.10 (m, 1H), 4.04 (q, *J* = 5.5 Hz, 1H), 3.99–3.89 (m, 1H), (2 protons are missing due to they are exchangeable with DMSO-*d*_6_). ^13^C NMR (DMSO-*d*_6_) δ: 153.88, 151.00, 150.12, 145.46, 131.43, 130.35, 129.62, 128.10, 119.74, 89.45, 83.24, 70.74, 70.36, 65.38. ^31^P NMR (DMSO-*d*_6_) δ: −0.15. LC–MS (*m*/*z*): positive mode 456.20 [M + H]^+^. Purity by HPLC-UV (254 nm)-ESI-MS: 99.4%.

#### ((2*R*,3*S*,4*R*,5*R*)-5-(6-Amino-8-((4-fluorophenyl)­thio)-9*H*-purin-9-yl)-3,4-dihydroxytetrahydrofuran-2-yl)­methyl dihydrogen Phosphate (**31n**)

The compound was synthesized using **30n** (60 mg, 0.15 mmol) and afforded a white powder (38 mg, 54%), mp: 143.0–145.0 °C. ^1^H NMR (DMSO-*d*_6_) δ: 8.21 (s, 1H), 7.80 (s, 2H), 7.58–7.51 (m, 2H), 7.27 (t, *J* = 8.8 Hz, 2H), 6.07 (d, *J* = 5.7 Hz, 1H), 5.14 (t, *J* = 5.6 Hz, 1H), 4.31 (dd, *J* = 5.3, 3.9 Hz, 2H), 4.20–4.10 (m, 2H), 4.08–4.00 (m, 2H), 3.98–3.86 (m, 2H). ^13^C NMR (DMSO-*d*_6_) δ: 163.17, 154.06, 151.31, 150.28, 145.91, 133.73, 133.66, 126.51, 119.68, 116.91, 116.73, 89.34, 83.21, 70.71, 70.39, 65.38. ^31^P NMR (DMSO-*d*_6_) δ: −0.13. LC–MS (*m*/*z*): positive mode 474.10 [M + H]^+^. Purity by HPLC-UV (254 nm)-ESI-MS: 96.7%.

#### ((2*R*,3*S*,4*R*,5*R*)-5-(6-Amino-8-((4-aminophenyl)­thio)-9*H*-purin-9-yl)-3,4-dihydroxytetrahydrofuran-2-yl)­methyl dihydrogen Phosphate (**31o**)

The compound was synthesized using **30o** (100 mg, 0.26 mmol) and afforded a white powder (31 mg, 25%), mp: 146.0–148.0 °C. ^1^H NMR (DMSO-*d*_6_) δ: 8.25 (d, *J* = 1.6 Hz, 1H), 8.18 (s, 2H), 7.32–7.24 (m, 2H), 6.65 (dd, *J* = 8.4, 1.4 Hz, 2H), 6.07 (dd, *J* = 5.8, 1.4 Hz, 1H), 5.40 (s, 5H), 5.09 (t, *J* = 5.6 Hz, 2H), 4.30 (s, 1H), 4.17–4.10 (m, 1H), 4.06 (q, *J* = 5.3, 4.4 Hz, 1H), 3.98–3.89 (m, 1H). ^13^C NMR (DMSO-*d*_6_) δ: 158.65, 152.09, 150.33, 150.18, 148.66, 135.45, 119.55, 115.81, 89.44, 83.48, 71.04, 70.50, 65.60. ^31^P NMR (DMSO-*d*_6_) δ: −0.14. LC–MS (*m*/*z*): positive mode 471.20 [M + H]^+^. Purity by HPLC-UV (254 nm)-ESI-MS: 99.5%.

#### ((2*R*,3*S*,4*R*,5*R*)-5-(6-Amino-8-(benzylthio)-9*H*-purin-9-yl)-3,4-dihydroxytetrahydrofuran-2-yl)­methyl dihydrogen Phosphate (**31p**)

The compound was synthesized using **30p** (100 mg, 0.26 mmol) and afforded a white powder (100 mg, 82%), mp: 149.0–151.0 °C. ^1^H NMR (DMSO-*d*_6_) δ: 8.09 (s, 1H), 7.53–7.47 (m, 2H), 7.31 (dd, *J* = 8.2, 6.8 Hz, 2H), 7.27–7.23 (m, 1H), 7.19 (s, 2H), 5.73 (d, *J* = 5.8 Hz, 1H), 5.05 (t, *J* = 5.7 Hz, 1H), 4.61–4.53 (m, 2H), 4.25 (dd, *J* = 5.5, 3.7 Hz, 2H), 4.03–3.88 (m, 4H), 3.75–3.66 (m, 2H). ^13^C NMR (DMSO-*d*_6_) δ: 154.34, 151.79, 151.21, 148.04, 137.15, 129.19, 128.43, 127.39, 119.20, 88.50, 83.53, 70.71, 70.46, 64.43, 35.77. ^31^P NMR (DMSO-*d*_6_) δ: 0.80. LC–MS (*m*/*z*): positive mode 470.4 [M + H]^+^. Purity by HPLC-UV (254 nm)-ESI-MS: 100%.

#### ((2*R*,3*S*,4*R*,5*R*)-5-(6-Amino-8-(phenethylthio)-9*H*-purin-9-yl)-3,4-dihydroxytetrahydrofuran-2-yl)­methyl dihydrogen Phosphate (**31q**), CAS: 78710–95–9

The compound was synthesized using **30q** (100 mg, 0.25 mmol) and afforded a white powder (56 mg, 46%), mp: 141.0–143.0 °C. ^1^H NMR (DMSO-*d*_6_) δ: 8.09 (s, 1H), 7.35–7.28 (m, 4H), 7.24–7.19 (m, 1H), 7.13 (s, 2H), 5.75 (d, *J* = 5.9 Hz, 1H), 5.12 (t, *J* = 5.7 Hz, 1H), 4.26 (dd, *J* = 5.5, 3.3 Hz, 1H), 4.04–3.97 (m, 3H), 3.80–3.75 (m, 3H), 3.59–3.49 (m, 3H), 3.03 (t, *J* = 7.7 Hz, 2H). ^13^C NMR (DMSO-*d*_6_) δ: 154.28, 151.67, 151.12, 148.49, 139.80, 128.68, 128.36, 126.36, 119.37, 88.48, 83.33, 70.58, 70.32, 64.71, 34.86, 33.39. ^31^P NMR (DMSO-*d*_6_) δ: 0.79 (d, *J* = 7.1 Hz). LC–MS (*m*/*z*): positive mode 484.5 [M + H]^+^. Purity by HPLC-UV (254 nm)-ESI-MS: 98.5%.

#### ((2*R*,3*S*,4*R*,5*R*)-5-(6-Amino-8-(naphthalen-1-ylthio)-9*H*-purin-9-yl)-3,4-dihydroxytetrahydrofuran-2-yl)­methyl dihydrogen Phosphate (**31r**)

The compound was synthesized using **30r** (100 mg, 0.24 mmol) and afforded a white powder (13 mg, 10%), mp: 188.0–190.0 °C. ^1^H NMR (DMSO-*d*_6_) δ: 8.32–8.24 (m, 1H), 8.14 (s, 1H), 8.04–7.98 (m, 2H), 7.70–7.57 (m, 3H), 7.52 (dd, *J* = 8.2, 7.3 Hz, 1H), 7.21 (s, 2H), 6.14 (d, *J* = 5.8 Hz, 1H), 5.26 (t, *J* = 5.7 Hz, 1H), 4.33 (dd, *J* = 5.5, 3.4 Hz, 1H), 4.10–3.99 (m, 3H), 3.87–3.76 (m, 4H). ^13^C NMR (DMSO-*d*_6_) δ: 155.02, 152.68, 150.69, 145.17, 133.81, 132.14, 131.72, 129.54, 128.73, 127.70, 127.47, 126.70, 126.18, 124.41, 119.76, 89.22, 83.55, 70.76, 70.55, 64.60. ^31^P NMR (DMSO-*d*_6_) δ: 0.84. LC–MS (*m*/*z*): positive mode 506.5 [M + H]^+^. Purity by HPLC-UV (254 nm)-ESI-MS: 97.7%.

#### ((2*R*,3*S*,4*R*,5*R*)-5-(6-Amino-8-methyl-9*H*-purin-9-yl)-3,4-dihydroxytetrahydrofuran-2-yl)­methyl dihydrogen Phosphate (**33a**), CAS: 68045–12–5

The compound was synthesized using **32a** (60 mg, 0.21 mmol) and afforded a white powder (21 mg, 28%), mp: 87.0–89.0 °C (*lit*.^65^ 208 °C). ^1^H NMR (D_2_O) δ: 8.38 (d, *J* = 0.9 Hz, 1H), 6.08 (d, *J* = 5.9 Hz, 1H), 5.19–5.12 (m, 1H), 4.84–4.79 (m, 2H), 4.79–4.73 (m, 2H), 4.62 (dd, *J* = 5.6, 4.4 Hz, 1H), 4.35–4.28 (m, 1H), 4.21–4.12 (m, 2H), 2.72 (d, *J* = 0.9 Hz, 3H). ^13^C NMR (D_2_O) δ: 157.69, 152.23, 151.77, 146.39, 120.29, 91.40, 86.59, 74.73, 72.67, 67.40, 16.97. ^31^P NMR (D_2_O) δ: 0.37. LC–MS (*m*/*z*): positive mode 362.10 [M + H]^+^. Purity by HPLC-UV (254 nm)-ESI-MS: 99.2%.

#### ((2*R*,3*S*,4*R*,5*R*)-5-(6-Amino-8-propyl-9*H*-purin-9-yl)-3,4-dihydroxytetrahydrofuran-2-yl)­methyl dihydrogen Phosphate (**33b**)

The compound was synthesized using **32c** (150 mg, 0.49 mmol) and afforded a white powder (14 mg, 7%), mp: >300 °C. ^1^H NMR (DMSO-*d*_6_) δ: 8.09 (s, 1H), 7.07 (s, 2H), 5.76 (d, *J* = 6.0 Hz, 1H), 5.09 (t, *J* = 5.8 Hz, 1H), 4.27 (dd, *J* = 5.5, 3.1 Hz, 2H), 3.99 (d, *J* = 6.4 Hz, 3H), 3.87–3.72 (m, 3H), 2.91–2.76 (m, 2H), 1.77 (h, *J* = 7.4 Hz, 2H), 0.98 (t, *J* = 7.4 Hz, 3H). ^13^C NMR (DMSO-*d*_6_) δ: 155.21, 152.37, 151.68, 150.33, 118.02, 87.99, 83.34, 70.65, 70.57, 64.63, 29.30, 20.91, 13.71. ^31^P NMR (DMSO-*d*_6_) δ: 0.95. LC–MS (*m*/*z*): positive mode 390.1 [M + H]^+^. Purity by HPLC-UV (254 nm)-ESI-MS: 98.0%.

#### ((2*R*,3*S*,4*R*,5*R*)-5-(6-Amino-8-(pent-1-yn-1-yl)-9*H*-purin-9-yl)-3,4-dihydroxytetrahydrofuran-2-yl)­methyl dihydrogen Phosphate (**33c**)

The compound was synthesized using **32d** (70 mg, 0.21 mmol) and afforded a white powder (38 mg, 44%), mp: 175.6–177.6 °C. ^1^H NMR (DMSO-*d*_6_) δ: 8.16 (s, 1H), 7.42 (s, 2H), 5.93 (d, *J* = 5.8 Hz, 1H), 5.10 (t, *J* = 5.6 Hz, 1H), 4.30 (t, *J* = 4.5 Hz, 2H), 4.03–3.95 (m, 4H), 3.75–3.67 (m, 2H), 2.57 (t, *J* = 6.9 Hz, 2H), 1.62 (h, *J* = 7.2 Hz, 2H), 1.02 (t, *J* = 7.4 Hz, 3H). ^13^C NMR (DMSO-*d*_6_) δ: 155.73, 153.43, 148.95, 133.82, 118.83, 97.34, 88.74, 83.51, 70.74, 70.61, 64.53, 64.50, 21.05, 20.45, 13.27. ^31^P NMR (DMSO-*d*_6_) δ: 0.80. LC–MS (*m*/*z*): positive mode 414.0 [M + H]^+^. Purity by HPLC-UV (254 nm)-ESI-MS: 98.1%.

#### ((2*R*,3*S*,4*R*,5*R*)-5-(6-Amino-8-(hex-1-yn-1-yl)-9*H*-purin-9-yl)-3,4-dihydroxytetrahydrofuran-2-yl)­methyl dihydrogen Phosphate (**33d**)

The compound was synthesized using **32e** (50 mg, 0.14 mmol) and afforded a white powder (2 mg, 3%), mp: 197.0–199.0 °C. ^1^H NMR (D_2_O) δ: 8.24 (s, 1H), 6.17 (d, *J* = 6.1 Hz, 1H), 5.27 (t, *J* = 6.1 Hz, 1H), 4.83 (s, 4H), 4.75 (dd, *J* = 4.1, 2.4 Hz, 2H), 4.53 (t, *J* = 5.4 Hz, 1H), 4.26 (q, *J* = 5.2 Hz, 1H), 4.11 (dt, *J* = 11.3, 5.6 Hz, 1H), 4.02 (dt, *J* = 11.9, 6.3 Hz, 1H), 2.61 (t, *J* = 7.0 Hz, 2H), 1.67 (p, *J* = 7.2 Hz, 2H), 1.50 (h, *J* = 7.4 Hz, 2H), 0.95 (t, *J* = 7.4 Hz, 3H). ^13^C NMR (D_2_O) δ: 157.95, 156.35, 151.63, 138.55, 121.31, 104.53, 91.16, 86.55, 73.58, 72.70, 71.45, 66.99, 32.02, 24.35, 21.31, 15.68. ^31^P NMR (D_2_O) δ: 2.54. LC–MS (*m*/*z*): positive mode 428.1 [M + H]^+^. Purity by HPLC-UV (254 nm)-ESI-MS: 95.6%.

#### ((2*R*,3*S*,4*R*,5*R*)-5-(6-Amino-8-pentyl-9*H*-purin-9-yl)-3,4-dihydroxytetrahydrofuran-2-yl)­methyl dihydrogen Phosphate (**33e**)

The compound was synthesized using **32f** (60 mg 0.18 mmol) and afforded a white powder (9 mg, 12%), mp: 137.0–139.0 °C. ^1^H NMR (D_2_O) δ: 8.40 (d, *J* = 0.9 Hz, 1H), 6.07 (d, *J* = 5.9 Hz, 1H), 5.26 (t, *J* = 5.7 Hz, 1H), 4.85–4.81 (m, 2H), 4.80–4.75 (m, 2H), 4.65 (dd, *J* = 5.6, 4.2 Hz, 1H), 4.32 (q, *J* = 4.6 Hz, 1H), 4.17 (hept, *J* = 5.8 Hz, 2H), 3.10–3.02 (m, 2H), 1.84 (p, *J* = 7.2 Hz, 2H), 1.45–1.31 (m, 4H), 0.89 (t, *J* = 7.0 Hz, 3H). ^13^C NMR (D_2_O) δ: 161.19, 152.13, 151.89, 146.29, 120.55, 91.41, 86.72, 74.57, 72.84, 67.34, 33.28, 30.08, 29.52, 24.51, 16.02. ^31^P NMR (D_2_O) δ: 0.58. LC–MS (*m*/*z*): positive mode 418.20 [M + H]^+^. Purity by HPLC-UV (254 nm)-ESI-MS: 96.3%.

#### ((2*R*,3*S*,4*R*,5*R*)-5-(6-Amino-8-phenyl-9*H*-purin-9-yl)-3,4-dihydroxytetrahydrofuran-2-yl)­methyl dihydrogen Phosphate (**33f**), CAS: 1018828–70–0

The compound was synthesized using **32g** (80 mg 0.23 mmol) and afforded a white powder (55 mg, 56%), mp: 169.0–171.0 °C. ^1^H NMR (DMSO-*d*_6_) δ: 8.28 (s, 1H), 7.98 (s, 2H), 7.81–7.71 (m, 2H), 7.63 (q, *J* = 3.0 Hz, 3H), 5.75 (d, *J* = 5.8 Hz, 1H), 5.68–5.30 (m, 2H), 5.24 (t, *J* = 5.7 Hz, 1H), 4.28 (t, *J* = 4.6 Hz, 1H), 4.23–4.12 (m, 1H), 4.10–3.92 (m, 2H), (2 protons are missing due to they are exchangeable with DMSO-*d*_6_). ^13^C NMR (DMSO-*d*_6_) δ: 154.07, 151.78, 149.99, 149.89, 130.36, 129.47, 129.05, 128.84, 118.94, 89.46, 83.21, 70.42, 70.31, 65.32. ^31^P NMR (DMSO-*d*_6_) δ: −0.09. LC–MS (*m*/*z*): positive mode 424.20 [M + H]^+^. Purity by HPLC-UV (254 nm)-ESI-MS: 98.4%.

#### ((2*R*,3*S*,4*R*,5*R*)-5-(6-Amino-8-cyclohexyl-9*H*-purin-9-yl)-3,4-dihydroxytetrahydrofuran-2-yl)­methyl dihydrogen Phosphate (**36**)

The compound was synthesized using **32h** (60 mg, 0.17 mmol) and afforded a white powder (13 mg, 18%), mp: 119–121 °C. ^1^H NMR (D_2_O) δ: 8.38 (s, 1H), 6.10 (d, *J* = 5.9 Hz, 1H), 5.28 (t, *J* = 5.8 Hz, 1H), 4.83–4.80 (m, 2H), 4.64 (dd, *J* = 5.7, 4.2 Hz, 1H), 4.35–4.27 (m, 1H), 4.23–4.11 (m, 2H), 3.81 (d, *J* = 11.1 Hz, 1H), 3.70 (d, *J* = 2.1 Hz, 1H), 3.17–3.07 (m, 1H), 2.10–1.98 (m, 2H), 1.92–1.81 (m, 2H), 1.76 (d, *J* = 13.5 Hz, 1H), 1.68–1.55 (m, 2H), 1.54–1.41 (m, 2H), 1.35–1.24 (m, 1H). ^13^C NMR (D_2_O) δ: 164.75, 152.07, 151.87, 146.20, 120.62, 91.17, 86.71, 74.53, 72.85, 67.50, 39.04, 34.51, 34.02, 28.22, 28.09. ^31^P NMR (D_2_O) δ: 0.40. LC–MS (*m*/*z*): positive mode 430.20 [M + H]^+^. Purity by HPLC-UV (254 nm)-ESI-MS: 95.0%.

#### ((2*R*,3*S*,4*R*,5*R*)-5-(8-(Cyclohexylthio)-6-((4-phenylbutyl)­amino)-9*H*-purin-9-yl)-3,4-dihydroxytetrahydrofuran-2-yl)­methyl dihydrogen Phosphate (**42a**)

The compound was synthesized using **41a** (60 mg, 0.12 mmol) and afforded the white powder (13 mg, 18%), mp: 185–187 °C. ^1^H NMR (500 MHz, DMSO-*d*_6_) δ: 8.14 (s, 1H), 7.69 (s, 1H), 7.25 (t, *J* = 7.5 Hz, 2H), 7.21–7.14 (m, 2H), 5.85 (s, 1H), 5.40 (s, 1H), 5.14 (t, *J* = 5.6 Hz, 1H), 4.29 (t, *J* = 4.7 Hz, 1H), 4.07–3.98 (m, 1H), 3.95–3.86 (m, 1H), 3.82–3.71 (m, 2H), 3.50 (s, 5H), 3.18 (s, 1H), 2.61 (s, 2H), 2.03 (s, 2H), 1.77–1.16 (m, 12H). ^13^C NMR (126 MHz, DMSO-*d*_6_) δ: 153.21, 151.86, 146.95, 142.15, 128.24, 128.15, 125.57, 88.80, 82.95, 70.38, 65.48, 46.65, 34.86, 32.81, 32.65, 28.41, 25.25, 24.98. ^31^P NMR (202 MHz, DMSO-*d*_6_) δ: −0.10 (t, *J* = 7.1 Hz). LC–MS (*m*/*z*): positive mode 594.40 [M + H]^+^. Purity by HPLC-UV (254 nm)-ESI-MS: 97.3%.

#### ((2*R*,3*S*,4*R*,5*R*)-3,4-Dihydroxy-5-(8-(naphthalen-1-ylthio)-6-((4-phenylbutyl)­amino)-9*H*-purin-9-yl)­tetrahydrofuran-2-yl)­methyl dihydrogen Phosphate (**42b**)

The compound was synthesized using **41b** (50 mg, 0.09 mmol) and afforded the white powder (3 mg, 5%), mp: 82–84 °C. ^1^H NMR (600 MHz, DMSO-*d*_6_) δ: 8.27 (d, *J* = 8.2 Hz, 1H), 8.22 (s, 1H), 8.01 (t, *J* = 8.8 Hz, 2H), 7.90 (s, 1H), 7.62 (p, *J* = 7.0 Hz, 3H), 7.52 (t, *J* = 7.7 Hz, 1H), 7.23 (t, *J* = 7.5 Hz, 2H), 7.14 (t, *J* = 7.2 Hz, 3H), 6.18 (s, 1H), 5.24 (d, *J* = 6.0 Hz, 1H), 4.34 (dd, *J* = 5.4, 3.8 Hz, 1H), 4.21–4.14 (m, 1H), 4.07 (s, 1H), 3.98 (dd, *J* = 11.0, 6.4 Hz, 1H), 3.40 (s, 2H), 2.55 (s, 2H), 1.53 (s, 3H). ^13^C NMR (151 MHz, DMSO-*d*_6_) δ: 153.2, 149.7, 144.8, 142.1, 133.8, 132.0, 131.5, 129.5, 128.8, 128.2, 128.1, 127.8, 127.4, 126.7, 126.2, 125.6, 124.3, 120.2, 89.4, 83.2, 83.2, 70.7, 70.4, 65.4, 65.4, 63.3, 42.9, 34.8, 28.5, 28.4. ^31^P NMR (243 MHz, DMSO-*d*_6_) δ: −0.07 (t, *J* = 5.9 Hz). LC–MS (*m*/*z*): positive mode 638.20 [M + H]^+^. Purity by HPLC-UV (254 nm)-ESI-MS: 95.0%.

#### ((2*R*,3*S*,4*R*,5*R*)-5-(8-(Butylthio)-6-(methyl­(4-phenylbutyl)­amino)-9*H*-purin-9-yl)-3,4-dihydroxytetrahydrofuran-2-yl)­methyl dihydrogen Phosphate (**42c**)

The compound was synthesized using **41c** (60 mg, 0.12 mmol) and afforded the white powder (7 mg, 10%), mp: 108–110 °C. ^1^H NMR (600 MHz, DMSO-*d*_6_) δ: 8.14 (s, 1H), 7.25 (dd, *J* = 8.3, 7.0 Hz, 2H), 7.16 (dd, *J* = 7.1, 1.8 Hz, 3H), 5.77 (d, *J* = 5.7 Hz, 1H), 5.40 (s, 1H), 5.09 (t, *J* = 5.6 Hz, 1H), 4.26 (dd, *J* = 5.5, 4.0 Hz, 1H), 4.17–4.09 (m, 2H), 4.05–3.98 (m, 2H), 3.90 (dt, *J* = 10.8, 6.8 Hz, 2H), 3.44 (s, 7H), 2.62 (t, *J* = 7.5 Hz, 2H), 1.74–1.57 (m, 6H), 1.39 (q, *J* = 7.4 Hz, 2H), 0.87 (t, *J* = 7.4 Hz, 3H). ^13^C NMR (151 MHz, DMSO-*d*_6_) δ: 152.15, 151.87, 150.86, 147.45, 142.04, 128.21, 128.17, 125.65, 119.52, 88.58, 82.95, 82.90, 70.30, 65.47, 65.44, 39.94, 39.80, 34.99, 31.54, 30.98, 28.25, 21.25, 13.38. ^31^P NMR (243 MHz, DMSO-*d*_6_) δ: −0.12. LC–MS (*m*/*z*): positive mode 582.50 [M + H]^+^. Purity by HPLC-UV (254 nm)-ESI-MS: 97.7%.

#### ((2*R*,3*S*,4*R*,5*R*)-3,4-Dihydroxy-5-(8-(methylamino)-6-((4-phenylbutyl)­amino)-9*H*-purin-9-yl)­tetrahydrofuran-2-yl)­methyl dihydrogen Phosphate (**42d**)

The compound was synthesized using **41d** (100 mg, 0.23 mmol) and afforded a white powder (11 mg, 9%), mp: 145.0–147.0 °C. ^1^H NMR (600 MHz, DMSO-*d*_6_) δ: 7.95 (s, 1H), 7.25 (t, *J* = 7.6 Hz, 2H), 7.20–7.17 (m, 2H), 7.17–7.13 (m, 1H), 6.84 (t, *J* = 6.1 Hz, 1H), 6.45 (d, *J* = 5.5 Hz, 1H), 5.89 (d, *J* = 7.4 Hz, 1H), 4.65 (dd, *J* = 7.4, 5.5 Hz, 1H), 4.20 (dd, *J* = 5.6, 2.5 Hz, 1H), 4.00 (p, *J* = 2.4 Hz, 2H), 3.96–3.80 (m, 5H), 3.51 (s, 2H), 2.96 (d, *J* = 3.0 Hz, 3H), 2.68–2.56 (m, 2H), 1.68–1.52 (m, 4H). ^13^C NMR (151 MHz, DMSO-*d*_6_) δ: 151.76, 151.37, 149.49, 148.65, 142.32, 128.29, 128.16, 125.53, 116.97, 85.90, 83.92, 70.59, 69.77, 64.44, 45.28, 34.92, 29.31, 28.50. ^31^P NMR (243 MHz, DMSO-*d*_6_) δ: 0.82. LC–MS (*m*/*z*): positive mode 509.3 [M + H]^+^. Purity by HPLC-UV (254 nm)-ESI-MS: 95.1%.

#### ((2*R*,3*S*,4*R*,5*R*)-5-(8-(Butylthio)-6-((4-phenylbutyl)­amino)-9*H*-purin-9-yl)-3,4-dihydroxytetrahydrofuran-2-yl)­methyl dihydrogen Phosphate (**42e**)

The compound was synthesized using **41e** (100 mg, 0.21 mmol) and afforded a white powder (33 mg, 28%), mp: 75.0–77.0 °C. ^1^H NMR (500 MHz, DMSO-*d*_6_) δ: 8.13 (q, *J* = 8.8, 7.6 Hz, 1H), 7.60 (t, *J* = 6.1 Hz, 1H), 7.25 (t, *J* = 7.5 Hz, 2H), 7.20–7.13 (m, 3H), 5.79 (d, *J* = 5.6 Hz, 1H), 5.42 (s, 1H), 5.13 (t, *J* = 5.7 Hz, 1H), 4.33–4.22 (m, 1H), 4.16–4.08 (m, 1H), 4.07–4.00 (m, 1H), 3.96–3.85 (m, 2H), 3.51 (s, 2H), 3.35–3.24 (m, 4H), 2.63–2.58 (m, 2H), 1.69 (p, *J* = 7.3 Hz, 2H), 1.65–1.58 (m, 4H), 1.41 (h, *J* = 7.5 Hz, 2H), 0.89 (t, *J* = 7.4 Hz, 3H). ^13^C NMR (126 MHz, DMSO-*d*_6_) δ: 153.03, 151.60, 148.16, 142.15, 128.24, 128.14, 125.56, 88.62, 82.92, 70.34, 65.41, (NH*C*H_2_ is overlapped by DMSO-*d*_6_), 34.86, 32.00, 30.88, 28.40, 21.19, 13.39. ^31^P NMR (202 MHz, DMSO-*d*_6_) δ: −0.04. LC–MS (*m*/*z*): positive mode 568.3 [M + H]^+^. Purity by HPLC-UV (254 nm)-ESI-MS: 95.4%.

#### ((2*R*,3*S*,4*R*,5*R*)-5-(6,8-Bis­(methylamino)-9*H*-purin-9-yl)-3,4-dihydroxytetrahydrofuran-2-yl)­methyl dihydrogen Phosphate (**47a**)

The compound was synthesized using **44a** (81 mg, 0.26 mmol) and afforded the white powder (14 mg, 14%), mp: 144–146 °C. ^1^H NMR (600 MHz, DMSO-*d*_6_) δ: 8.05 (s, 1H), 6.59 (s, 1H), 5.80 (d, *J* = 6.1 Hz, 1H), 5.33 (s, 1H), 4.81 (t, *J* = 5.9 Hz, 1H), 4.21 (dd, *J* = 5.7, 3.9 Hz, 1H), 4.15–3.98 (m, 4H), 3.98–3.85 (m, 3H), 2.94 (d, *J* = 4.4 Hz, 3H), 2.92 (d, *J* = 2.9 Hz, 3H). ^13^C NMR (151 MHz, DMSO-*d*_6_) δ: 157.88, 157.67, 152.16, 148.53, 116.19, 86.81, 82.77, 70.10, 70.02, 65.54, 29.29, 27.51. ^31^P NMR (243 MHz, DMSO-*d*_6_) δ: −0.07. LC–MS (*m*/*z*): positive mode 391.2 [M + H]^+^. Purity by HPLC-UV (254 nm)-ESI-MS: 95.0%.

#### ((2*R*,3*S*,4*R*,5*R*)-5-(8-(Butylamino)-6-(methylamino)-9*H*-purin-9-yl)-3,4-dihydroxytetra-hydrofuran-2-yl)­methyl dihydrogen Phosphate (**47b**)

The compound was synthesized using **44b** (320 mg, 1.00 mmol) and afforded the white powder (90 mg, 22%), mp: 157 °C. ^1^H NMR (500 MHz, D_2_O) δ: 7.99 (s, 1H), 5.97 (d, 1H, *J* = 7.68 Hz), 4.69 (dd, 1H, *J* = 5.95, 7.53 Hz), 4.44 (dd, 1H, *J* = 2.51, 5.79 Hz), 4.34 (m, 1H), 4.17 (d m, 2H), 3.42 (m, 2H), 3.01 (s, 3H), 1.63 (m, 2H), 1.37 (m, 2H), 0.92 (t, 3H, *J* = 7.40 Hz). ^13^C NMR (125 MHz, D_2_O) δ: 154.66, 153.51, 150.97, 150.34, 118.72, 89.18, 87.10, 73.42, 72.91, 67.45, 45.25, 33.49, 30.30, 22.35, 16.05. ^31^P NMR (202 MHz, D_2_O) δ: 0.38. LC–MS (*m*/*z*): positive mode 433.1587 [M + H]^+^ and negative mode 431.1456 [M – H]^−^. Purity by HPLC-UV (254 nm)-ESI-MS: 99.2%.

#### ((2*R*,3*S*,4*R*,5*R*)-5-(8-(Butylamino)-6-(ethyl-amino)-9*H*-purin-9-yl)-3,4-dihydroxytetrahydro-furan-2-yl)­methyl dihydrogen Phosphate (**47c**)

The compound was synthesized using **44c** (80 mg, 0.22 mmol) and afforded the white powder (60 mg, 56%), mp: 186 °C. ^1^H NMR (500 MHz, D_2_O) δ: 7.48 (s, 1H), 5.99 (d, 1H, *J* = 7.77 Hz), 4.70 (m, 1H), 4.44 (dd, 1H, *J* = 2.37, 5.78 Hz), 4.33 (br s, 1H), 4.15 (m, 2H), 3.43 (m, 4H), 1.64 (m, 2H), 1.37 (m, 2H), 1.26 (m, 3H), 0.91 (d, 3H, *J* = 7.36 Hz). ^13^C NMR (125 MHz, D_2_O) δ: 154.72, 153.32, 151.47, 151.32, 150.65, 118.90, 89.09, 87.24, 87.18, 73.34, 72.99, 67.47, 45.26, 38.74, 33.52, 22.37, 16.62, 16.17. ^31^P NMR (202 MHz, D_2_O) δ: 0.29. LC–MS (*m*/*z*): positive mode 447.1739 [M + H]^+^ and negative mode 445.1617 [M – H]^−^. Purity by HPLC-UV (254 nm)-ESI-MS: 95.0%.

#### ((2*R*,3*S*,4*R*,5*R*)-5-(6-(Dimethylamino)-8-((4-phenylbutyl)­amino)-9*H*-purin-9-yl)-3,4-dihydroxy-tetrahydrofuran-2-yl)­methyl dihydrogen Phosphate (**47d**)

The compound was synthesized using **44d** (100 mg, 0.23 mmol) and afforded the white powder (30 mg, 22%), mp: 163 °C. ^1^H NMR (500 MHz, D_2_O) δ: 7.90 (s, 1H), 7.05 (m, 5H), 5.99 (d, 1H, *J* = 7.80 Hz), 4.67 (m, 1H), 4.44 (dd, 1H, *J* = 1.82, 5.61 Hz), 4.30 (br s, 1H), 4.16 (d m, 2H), 3.45 (d m, 2H), 3.14 (s, 6H), 2.52 (m, 2H), 1.63 (m, 4H, overlapping peaks (C*H*_2_)_2_ – aryl). ^13^C NMR (125 MHz, D_2_O) δ: 154.15, 153.47, 152.19, 150.91, 145.61, 131.14, 121.00, 128.29, 119.88, 88.92, 87.21, 73.49, 73.12, 67.31, 44.93, 41.17, 41.13, 37.40, 30.28, 30.21. ^31^P NMR (202 MHz, D_2_O) δ: 1.31. LC–MS (*m*/*z*): positive mode 523.2049 [M + H]^+^ and negative mode 521.1933 [M – H]^−^. Purity by HPLC-UV (254 nm)-ESI-MS: 97.0%.

#### ((2*R*,3*S*,4*R*,5*R*)-5-(6-(Diethylamino)-8-(methylamino)-9*H*-purin-9-yl)-3,4-dihydroxytetra-hydrofuran-2-yl)­methyl dihydrogen Phosphate (**47e**)

The compound was synthesized using **44e** (80 mg, 0.23 mmol) and afforded the white powder (6 mg, 5%), mp: >219 °C (decomposition). ^1^H NMR (500 MHz, D_2_O) δ: 8.0 (s, 1H), 6.05 (d, 1H, *J* = 7.86 Hz), 4.66 (dd, 1H, *J* = 5.91, 7.72 Hz), 4.43 (dd, 1H, *J* = 2.17, 5.77 Hz), 4.35 (m, 1H), 4.16 (d m, 2H), 3.85 (m, 4H), 3.05 (s, 3H), 1.21 (t, 6H, *J* = 7.08 Hz). ^13^C NMR (125 MHz, D_2_O) δ: 155.88, 154.45, 153.90, 153.36, 121.65, 90.90, 86.33, 73.63, 72.55, 67.38, 46.40, 31.76, 15.61. ^31^P NMR (202 MHz, D_2_O) δ: 0.65. LC–MS (*m*/*z*): positive mode 433.1592 [M + H]^+^ and negative mode 431.1480 [M – H]^−^. Purity by HPLC-UV (254 nm)-ESI-MS: 97.9%.

#### ((2*R*,3*S*,4*R*,5*R*)-5-(8-(Butylamino)-6-(diethylamino)-9*H*-purin-9-yl)-3,4-dihydroxytetra-hydrofuran-2-yl)­methyl dihydrogen Phosphate (**47f**)

The compound was synthesized using **44f** (100 mg, 0.30 mmol) and afforded the white powder (30 mg, 25%), mp: 192 °C. ^1^H NMR (500 MHz, D_2_O) δ: 7.99 (s, 1H), 6.04 (d, 1H, *J* = 7.84 Hz), 4.69 (dd, 1H, *J* = 5.92, 7.74 Hz), 4.44 (dd, 1H, *J* = 2.29, 5.78 Hz), 4.34 (m, 1H), 4.19 (d m, 2H), 3.83 (q, 4H, *J* = 7.06 Hz), 3.54–3.42 (d m, 2H), 1.65 (m, 2H), 1.34 (m, 2H), 1.19 (t, 6H, *J* = 7.06 Hz), 0.90 (t, 3H, *J* = 7.40 Hz). ^13^C NMR (125 MHz, D_2_O) δ: 154.17, 152.53, 152.39, 150.42, 119.72, 88.92, 87.21, 73.42, 73.03, 67.44, 46.27, 45.16, 33.65, 22.31, 16.02, 15.69. ^31^P NMR (202 MHz, D_2_O) δ: 0.72. LC–MS (*m*/*z*): positive mode 475.2070 [M + H]^+^ and negative mode 473.1916 [M – H]^−^. Purity by HPLC-UV (254 nm)-ESI-MS: 98.6%.

#### ((2*R*,3*S*,4*R*,5*R*)-5-(8-(Butylthio)-6-(methylamino)-9*H*-purin-9-yl)-3,4-dihydroxytetrahydro-furan-2-yl)­methyl dihydrogen Phosphate (**48a**)

The compound was synthesized using **45a** (200 mg, 0.54 mmol) and afforded the white powder (130 mg, 51%), mp: 162 °C. ^1^H NMR (500 MHz, D_2_O) δ: 8.09 (s, 1H), 6.04 (d, 1H, *J* = 6.22 Hz), 5.12 (t, 1H, *J* = 6.22 Hz), 4.59 (m, 1H), 4.25 (q, 1H, *J* = 4.98 Hz), 4.14 (m, 2H), 3.20 (m, 2H), 3.01 (s, 3H), 1.68 (m, 2H), 1.42 (m, 2H), 0.89 (t, 3H, *J* = 7.39 Hz). ^13^C NMR (125 MHz, D_2_O) δ: 156.07, 154.55, 153.08, 152.08, 122.10, 90.84, 86.38, 73.58, 72.62, 67.22, 35.68, 33.45, 30.19, 24.07, 15.66. ^31^P NMR (202 MHz, D_2_O) δ: 1.33. LC–MS (*m*/*z*): positive mode 450.1195 [M + H]^+^ and negative mode 448.1047 [M – H]^−^. Purity by HPLC-UV (254 nm)-ESI-MS: 97.8%.

#### ((2*R*,3*S*,4*R*,5*R*)-5-(8-(Butylthio)-6-(diethyl-amino)-9*H*-purin-9-yl)-3,4-dihydroxytetrahydro-furan-2-yl)­methyl dihydrogen Phosphate (**48b**)

The compound was synthesized using **45b** (100 mg, 0.24 mmol) and afforded the white powder (50 mg, 41%), mp: 167 °C. ^1^H NMR (500 MHz, D_2_O) δ: 8.11 (s, 1H), 6.12 (d, 1H, *J* = 6.40 Hz), 5.12 (t, 1H, *J* = 6.28 Hz), 4.52 (m, 1H), 4.25 (q, 1H, *J* = 4.95 Hz), 4.15 (d m, 2H), 3.87 (br s, 4H), 3.22 (d m, 2H), 1.70 (m, 2H), 1.40 (m, 2H), 1.22 (t, 6H, *J* = 7.01 Hz), 0.87 (t, 3H, *J* = 7.38 Hz). ^13^C NMR (125 MHz, D_2_O) δ: 154.65, 154.06, 153.94, 151.08, 122.28, 90.69, 86.35, 73.57, 72.62, 67.32, 46.69, 30.25, 34.08, 24.20, 15.71, 15.52. ^31^P NMR (202 MHz, D_2_O) δ: 1.00. LC–MS (*m*/*z*): positive mode 492.1673 [M + H]^+^ and negative mode 490.1527 [M – H]^−^. Purity by HPLC-UV (254 nm)-ESI-MS: 96.0%.

#### ((2*R*,3*S*,4*R*,5*R*)-5-(6-(Diethylamino)-8-(naphthalen-1-ylthio)-9*H*-purin-9-yl)-3,4-dihydroxytetrahydrofuran-2-yl)­methyl dihydrogen Phosphate (**48c**)

The compound was synthesized using **45c** (100 mg, 0.21 mmol) and afforded the white powder (11 mg, 9%), mp: 120–122 °C. ^1^H NMR (500 MHz, DMSO-*d*_6_) δ: 8.30 (dd, *J* = 7.6, 1.8 Hz, 1H), 8.15 (s, 1H), 8.06 (d, *J* = 8.3 Hz, 1H), 8.03–8.00 (m, 1H), 7.82 (dd, *J* = 7.2, 1.2 Hz, 1H), 7.62–7.58 (m, 2H), 7.55 (dd, *J* = 8.3, 7.2 Hz, 1H), 6.10 (d, *J* = 5.8 Hz, 1H), 5.19 (t, *J* = 5.6 Hz, 2H), 4.34 (dd, *J* = 5.5, 3.9 Hz, 2H), 4.24–4.13 (m, 2H), 4.12–4.07 (m, 1H), 4.03–3.92 (m, 2H), 3.56 (q, *J* = 6.7, 6.2 Hz, 4H), 1.22–0.66 (m, 6H). ^13^C NMR (151 MHz, DMSO-*d*_6_) δ: 159.51, 151.73, 151.66, 144.87, 133.86, 133.22, 133.05, 130.19, 128.54, 127.23, 126.60, 126.49, 125.93, 125.07, 119.40, 89.04, 83.17, 70.59, 70.40, 65.46, 40.06, 12.87. ^31^P NMR (243 MHz, DMSO-*d*_6_) δ: −0.07. LC–MS (*m*/*z*): positive mode 562.20 [M + H]^+^. Purity by HPLC-UV (254 nm)-ESI-MS: 97.3%.

#### ((2*R*,3*S*,4*R*,5*R*)-3,4-Dihydroxy-5-(8-phenyl-6-((4-phenylbutyl)­amino)-9*H*-purin-9-yl)­tetrahydrofuran-2-yl)­methyl dihydrogen Phosphate (**49**)

The compound was synthesized using **46** (60 mg, 0.13 mmol) and afforded the white powder (10 mg, 14%), mp: 130–132 °C. ^1^H NMR (600 MHz, DMSO-*d*_6_) δ: 8.28 (d, *J* = 3.4 Hz, 1H), 7.79–7.70 (m, 2H), 7.65–7.57 (m, 3H), 7.25 (q, *J* = 7.8 Hz, 2H), 7.19 (d, *J* = 7.5 Hz, 2H), 7.17–7.13 (m, 1H), 5.74 (d, *J* = 5.8 Hz, 1H), 5.28 (t, *J* = 5.6 Hz, 4H), 4.29 (dd, *J* = 5.4, 3.6 Hz, 1H), 4.24–4.10 (m, 2H), 4.06–3.91 (m, 3H), 3.52 (s, 2H), 2.61 (q, *J* = 5.3, 3.5 Hz, 2H), 1.70–1.57 (m, 4H). ^13^C NMR (151 MHz, DMSO-*d*_6_) δ: 157.93, 153.74, 150.94, 149.32, 142.16, 130.20, 129.45, 129.30, 128.82, 128.29, 128.17, 125.59, 119.34, 89.38, 83.14, 70.39, 70.30, 65.43, 40.06, 34.84, 28.59, 28.40. ^31^P NMR (243 MHz, DMSO-*d*_6_) δ: −0.10. LC–MS (*m*/*z*): positive mode 556.50 [M + H]^+^. Purity by HPLC-UV (254 nm)-ESI-MS: 95.3%.

#### ((2*R*,3*S*,4*R*,5*R*)-5-(2-Chloro-6-((4-phenylbutyl)­amino)-9*H*-purin-9-yl)-3,4-dihydroxytetrahydrofuran-2-yl)­methyl dihydrogen Phosphate (**53**)

The compound was synthesized using **52** (100 mg, 0.23 mmol) and afforded the white powder (39 mg, 33%), mp: 159.5–161.0 °C. ^1^H NMR (500 MHz, DMSO-*d*_6_) δ: 8.50 (s, 1H), 7.31–7.10 (m, 5H), 5.82 (d, *J* = 6.2 Hz, 2H), 4.78–4.52 (m, 3H), 4.21 (dd, *J* = 4.8, 2.7 Hz, 2H), 4.05 (q, *J* = 3.4 Hz, 1H), 3.95–3.74 (m, 3H), 3.46 (s, 2H), 2.60 (s, 2H), 1.61 (q, *J* = 4.6, 3.2 Hz, 4H). ^13^C NMR (151 MHz, DMSO-*d*_6_) δ: 154.99, 153.31, 149.88, 142.11, 139.41, 128.27, 128.14, 125.55, 117.96, 86.47, 84.64, 74.25, 71.19, 63.96, 45.59, 34.72, 28.35, 28.28. ^31^P NMR (DMSO-*d*_6_) δ: 1.23. LC–MS (*m*/*z*): positive mode 514.4 [M + H]^+^. Purity by HPLC-UV (254 nm)-ESI-MS: 99.6%.

#### ((2*R*,3*S*,4*R*,5*R*)-5-(2-Amino-6-((4-phenylbutyl)­amino)-9*H*-purin-9-yl)-3,4-dihydroxytetrahydrofuran-2-yl)­methyl dihydrogen Phosphate (**56**)

The compound was synthesized using **55** (100 mg, 0.24 mmol) and afforded the white powder (37 mg, 26%), mp: 117.0–119.0 °C. ^1^H NMR (600 MHz, DMSO-*d*_6_) δ: 7.98 (s, 1H), 7.34–7.08 (m, 6H), 5.83 (s, 2H), 5.74 (d, *J* = 6.2 Hz, 1H), 4.55 (t, *J* = 5.6 Hz, 1H), 4.20 (dd, *J* = 4.9, 3.1 Hz, 2H), 3.97 (q, *J* = 3.7 Hz, 2H), 3.90–3.74 (m, 3H), 3.51 (s, 1H), 3.42 (d, *J* = 13.6 Hz, 2H), 2.60 (t, *J* = 7.1 Hz, 2H), 1.68–1.50 (m, 4H). ^13^C NMR (151 MHz, DMSO-*d*_6_) δ: 160.22, 154.89, 142.28, 135.42, 128.29, 128.16, 125.55, 85.94, 83.80, 73.55, 71.05, 64.10, 45.32, 34.92, 29.02, 28.48. ^31^P NMR (DMSO-*d*_6_) δ: 1.09. LC–MS (*m*/*z*): positive mode 495.3 [M + H]^+^. Purity by HPLC-UV (254 nm)-ESI-MS: 95.8%.

#### ((2*R*,3*S*,4*R*,5*R*)-5-(2-Bromo-6-chloro-9*H*-purin-9-yl)-3,4-dihydroxytetrahydrofuran-2-yl)­methyl dihydrogen Phosphate (**58**)

The compound was synthesized using **57** (100 mg, 0.27 mmol) and afforded the white powder (25 mg, 21%), mp: 136–140 °C. ^1^H NMR (600 MHz, D_2_O) δ: 8.82 (s, 1H), 6.20 (d, *J* = 5.0 Hz, 1H), 4.77–4.75 (m, 1H, *partially overlapped with D*_*2*_ *O peak*), 4.51 (t, *J* = 4.6 Hz, 1H), 4.42–4.39 (m, 1H), 4.21–4.11 (m, 2H), 3.20 (q, *J* = 7.3 Hz, 1H, CH_2_–Et_3_N^+^), 1.27 (t, *J* = 7.3 Hz, 1H, CH_3_–Et_3_N^+^). ^13^C NMR (151 MHz, D_2_O) δ: 155.53, 153.52, 145.47, 133.80, 91.08, 86.97, 77.40, 73.04, 67.12, 49.52 (Et_3_N^+^), 11.07 (Et_3_N^+^). ^31^P NMR (243 MHz, D_2_O) δ: 0.56. LC–MS (*m*/*z*): positive mode 446.6 [M + H]^+^. Purity by HPLC-UV (254 nm)-ESI-MS: 85.0%.

#### ((2*R*,3*S*,4*R*,5*R*)-5-(2-Bromo-6-((4-phenylbutyl)­amino)-9*H*-purin-9-yl)-3,4-dihydroxytetrahydrofuran-2-yl)­methyl dihydrogen Phosphate (**59**)

To **58** (20 mg, 0.05 mmol) in 5 mL dry EtOH, triethylamine (0.012 mL, 0.09 mmol) and 4-phenylbutylamine (0.014 mL, 0.09 mmol) were added. The mixture was reflux at 90 °C for 6 h, after the reaction was completed, cooled to rt and the solvent was evaporated *in vacuum*. The crude mixture was purified by reversed-phase HPLC to afford the white powder (11 mg, 45%), mp: 181–183 °C. ^1^H NMR (600 MHz, D_2_O) δ: 8.45 (s, 1H), 7.39–7.36 (m, 1H), 7.32–7.29 (m, 1H), 7.21 (s br, 2H), 7.12 (s br, 1H), 6.05 (d, *J* = 5.5 Hz, 1H), 4.50–4.48 (m, 1H), 4.41–4.38 (m, 1H), 4.16–4.12 (m, 2H), 3.55–3.49 (m, 2H), 2.68–2.64 (m, 2H), 1.77–1.68 (m, 4H). ^13^C NMR (151 MHz, D_2_O) δ: 162.94, 159.10, 156.09, 145.38, 140.66, 131.17, 131.03, 128.44, 118.90, 91.32, 87.68, 79.21, 74.52, 67.06, 37.25. ^31^P NMR (243 MHz, D_2_O) δ: 0.52. LC–MS (*m*/*z*): positive mode 558.0 [M + H]^+^. Purity by HPLC-UV (254 nm)-ESI-MS: 99.0%.

#### ((2*R*,3*S*,4*R*,5*R*)-3,4-Dihydroxy-5-(3*H*-imidazo­[2,1-*i*]­purin-3-yl)­tetrahydrofuran-2-yl)­methyl dihydrogen Phosphate (**61a**), CAS: 37482–16–9

The compound was synthesized using **60a** (60 mg, 0.21 mmol) and afforded the white powder (12 mg, 15%), mp: 96.0–99.0 °C (*lit*.^74^ 194–198 °C). ^1^H NMR (600 MHz, D_2_O) δ: 9.43 (s, 1H), 8.84 (s, 1H), 8.30 (d, *J* = 2.3 Hz, 1H), 7.95 (d, *J* = 2.2 Hz, 1H), 6.37 (d, *J* = 5.1 Hz, 1H), 4.88 (t, *J* = 5.1 Hz, 1H), 4.79–4.73 (m, 4H), 4.57 (t, *J* = 4.6 Hz, 1H), 4.43 (p, *J* = 3.2 Hz, 1H), 4.24–4.12 (m, 2H). ^13^C NMR (151 MHz, D_2_O) δ: 146.10, 145.69, 140.44, 139.59, 124.56, 121.29, 116.95, 91.21, 86.99, 77.37, 72.95, 66.94. ^31^P NMR (243 MHz, D_2_O) δ: 0.46. LC–MS (*m*/*z*): positive mode 372.20 [M + H]^+^. Purity by HPLC-UV (254 nm)-ESI-MS: 98.6%.

#### ((2*R*,3*S*,4*R*,5*R*)-5-(2-(Butylthio)-3*H*-imidazo­[2,1-*i*]­purin-3-yl)-3,4-dihydroxytetrahydrofuran-2-yl)­methyl dihydrogen Phosphate (**61b**)

The compound was synthesized using **60b** (60 mg, 0.16 mmol) and afforded the brownish powder (22 mg, 30%), mp: 109.0–111.0 °C. ^1^H NMR (600 MHz, D_2_O) δ: 9.32 (d, *J* = 1.0 Hz, 1H), 8.25 (d, *J* = 2.2 Hz, 1H), 7.92 (d, *J* = 2.1 Hz, 1H), 6.26–6.19 (m, 1H), 5.32 (t, *J* = 5.6 Hz, 1H), 4.84–4.79 (m, 2H), 4.79–4.74 (m, 2H), 4.69 (dd, *J* = 5.6, 4.4 Hz, 1H), 4.33 (q, *J* = 4.8 Hz, 1H), 4.20 (t, *J* = 5.7 Hz, 2H), 3.48–3.32 (m, 2H), 1.77 (p, *J* = 7.4 Hz, 2H), 1.47 (h, *J* = 7.4 Hz, 2H), 0.91 (t, *J* = 7.4 Hz, 3H). ^13^C NMR (151 MHz, D_2_O) δ: 159.47, 147.39, 138.99, 138.25, 124.78, 122.06, 116.82, 92.06, 86.75, 74.50, 72.85, 67.44, 35.68, 33.55, 24.05, 15.64. ^31^P NMR (243 MHz, D_2_O) δ: 0.39. LC–MS (*m*/*z*): positive mode 460.30 [M + H]^+^. Purity by HPLC-UV (254 nm)-ESI-MS: 97.8%.

### Biological Assays

#### Chemicals and Materials

ATP, calcium chloride, magnesium chloride, 4-(2-hydroxyethyl)­piperazine-1-ethanesulfonic acid (HEPES), ammonium heptamolybdate, dimethyl sulfoxide (DMSO), malachite green, α,β-methylene ATP, α,β-methylene ADP, β,γ-methyleneadenosine ATP and poly­(vinyl alcohol) were obtained from Sigma (Steinheim, Germany). Disodium hydrogen phosphate and sulfuric acid were purchased from Carl Roth (Karlsruhe, Germany). *N* ^6^-[6-(Fluoresceinyl-5′-carboxamido)­hexyl]-ATP (PSB-170621A) was obtained from Jena Bioscience (Jena, Germany). *Polyacrylamide*-coated capillary [30 cm (10 cm effective length) × 50 μM (id), × 360 μm (od)] was purchased from Chromatographie Service GmbH (Langerwehe, Germany).

#### Human CD39 Preparation

Human umbilical cords were obtained as previously described.^22^ They were minced and homogenized with a polytron in 95 mM NaCl, 0.1 mM phenylmethylsulfonyl fluoride (PMSF) and 45 mM Tris solution, pH 7.6. The homogenates were then filtered through a cheese cloth, centrifuged for 15 min at 600 *g*, and the supernatants were subsequently centrifuged for 1 h at 100,000*g*. The pellets were resuspended in 5 mM Tris buffer solution, pH 8.0 and 10% glycerol. All purification steps were performed at 4 °C. The preparations were kept at −80 °C.

#### Fluorescence-Based Capillary Electrophoresis Assay for CD39

The enzyme activity assay was performed as previously described.^22^ The test compounds were initially investigated at a concentration of 10 μM (*n* = 3), 0.5 μM fluorescent substrate PSB-017621A (*K*_m_ = 19.6 μM), and 40 ng protein from human umbilical cord membrane preparations containing CD39 were added to initiate the reaction. The reaction buffer contained 10 mM HEPES, 2 mM CaCl_2_, 1 mM MgCl_2_, pH 7.4 in a final volume of 100 μL. Incubation at 37 °C for 4 min, followed by termination of the enzymatic reaction by heating at 90 °C for 5 min. The samples were then diluted 1:20 with reaction buffer to perform separation of nucleotides by capillary electrophoresis (CE) followed by laser-induced fluorescence (LIF) detection. Concentration-inhibition curves were generated at concentrations ranging from 0.01 to 300 μM (*n* = 3), plotted with GraphPad Prism 8 software (GraphPad software, San Diego, CA, USA) and the *K*_i_ value was calculated using the Cheng-Prusoff equation for competitive inhibitors: $K_{i}=\frac{{IC}_{50}}{1+\frac{\left[S\right]}{K_{m}}}$ .

Analysis was carried out using a P/ACE MDQ capillary electrophoresis system (Beckman Instruments, Fullerton, CA, USA) using a *polyacrylamide*-coated capillary [30 cm (10 cm effective length) × 50 μm (id), × 360 μm (od)]. Before each run, the capillary was rinsed with the background electrolyte (50 mM phosphate buffer (pH 6.5)) for 1 min at 30 psi. Electrokinetic injection of samples by applying a voltage of −6 kV for 30 s at the capillary outlet, and separation of the fluorescent nucleotide derivatives by voltage application of −15 kV. Detection was performed at an excitation wavelength of 488 nm and an emission wavelength of 520 nm. Data collection and peak area analysis were performed by the P/ACE MDQ software 32 KARAT obtained from Beckman Coulter (Fullerton, CA, USA).

#### Malachite Green Assay for CD39 and Human NTPDases2, −3 and −8

The ATPase activity in membrane preparations was determined essentially as previously described with a few adaptations.^95^ The reaction buffer contained 10 mM HEPES, 2 mM CaCl_2_, 1 mM MgCl_2_, pH 7.4 in a final volume of 50 μL in transparent 96-well half area plates. Human umbilical cord membrane preparations (250 ng) expressing high amounts of CD39 or the respective recombinant COS-7-cell membrane preparations expressing the appropriate NTPDase isoenzyme (ca. 100 ng of protein depending on enzyme activity, adjusted to ensure 10–20% of substrate conversion)^26,76^ with or without 50 μM inhibitor were preincubated at 37 °C and gentle shaking (Eppendorf Thermomixer comfort at 500 rpm) for 5 min. The reaction was initiated by the addition of 50 μM ATP (*K*_m_ (CD39) = 17 μM; *K*_m_ (NTPDase2) = 70 μM; *K*_m_ (NTPDase3) = 75 μM; *K*_m_ (NTPDase8) = 46 μM).^77^ After 15 min of incubation at 37 °C with gentle shaking, the reaction was terminated by adding the detection reagents (20 μL malachite green solution, 0.6 mM, and 30 μL ammonium molybdate solution, 20 mM in 1.5 M H_2_SO_4_). The formation of (inorganic) phosphate was quantified after 20 min of gentle shaking at 25 °C by measuring the absorption of the malachite green-phosphomolybdate complex at 600 nm using a BMG PheraStar FS plate reader (BMG Labtech GmbH, Ortenberg, Germany). Enzyme-independent background was corrected by subtracting the absorbance of control samples containing heat-inactivated membrane protein (90 °C, 15 min), and the inhibition was calculated as follows: $\%Inhibition=\frac{\left(B-T\right)}{B}\times 100\%$ , where *B* is the average corrected absorption of the positive control without inhibitor and *T* the corrected absorption in the presence of test compound.

Full concentration–inhibition curves were determined with inhibitor concentrations ranging from 0.1 to 300 μM in the presence of max. 2% DMSO. Three independent experiments were performed (*n* = 3) and curves were calculated using GraphPad Prism 8. The *K*_i_ value was calculated using the Cheng-Prusoff equation for competitive inhibitors: $K_{i}=\frac{{IC}_{50}}{1+\frac{\left[S\right]}{K_{m}}}$ .

Further assays were performed as previously described.^22,43,44^ Detailed assay procedures for selectivity studies at human CD38, soluble CD39, CD73, and NPP1–5, membrane preparations of human melanoma cells, flow cytometry, and T cell activation and proliferation assay, can be found in Supporting Information.

#### Determination of Inhibition type

The inhibition mode of the CD39 inhibitor **42b** was determined using a range of substrate and inhibitor concentrations. Initial velocities were measured using the malachite green assay and analyzed with GraphPad Prism 9 by global nonlinear regression. Apparent *K*_m_ values, *K*_m_ app, and *V*_max_ values were obtained from the fitted parameters. The analysis showed an inhibitor concentration-dependent increase in *K*_m_ app, whereas *V*_max_ remained essentially unchanged, consistent with competitive inhibition. For graphical validation, the same data set was additionally evaluated using a Hanes-Woolf plot, which yielded parallel lines across different inhibitor concentrations. The inhibition constant was determined from the global fit, resulting in a *K*_i_ value of 0.197 ± 0.024 μM.

#### Quantitative determination of ATP Degradation in Activated T Cells by HPLC

Activated human T cells were used as a source of CD39. T cells were analyzed on day 4 after stimulation with anti-CD3/anti-CD28 antibodies. Cells were preincubated with **42b** (10, 30, and RM50 μM) for 15 min, followed by the addition of 1 μM etheno-ATP (eATP). After incubation for 30 min, the conversion of eATP into eADP and eAMP was quantified by reversed-phase HPLC.

#### Molecular Modeling

Molecular docking was carried out in Schrödinger Maestro using a previously reported homology model of human CD39 based on rat CD39 (PDB 3ZX3),^22,44^ which included the bound Ca^2+^ ion and coordinating water molecules. The receptor was preprocessed with the Protein Preparation Wizard, assigning bond orders, adding hydrogens, optimizing protonation states, and energy-minimizing the structure. Ligands were prepared with LigPrep to generate low-energy 3D conformers at pH 7.4. The docking grid was centered on the nucleotide-binding pocket, defined by the ligand position in the homology model, while retaining the Ca^2+^ ion and coordinating waters. Static ligand docking was performed in extra-precision (XP) mode without applying constraints. The resulting poses were visually inspected and the most plausible binding mode was selected. UCSF ChimeraX^79^ was utilized for visualization and figure generation.

## Supplementary Material







## Notes

Figures and Tables for selectivity studies of selected AMP derivatives at human ectonucleotidases, potency of AMP derivatives as inhibitors at soluble and human membrane-bound CD39, potency of AMP derivatives as inhibitors of soluble human CD73, concentration–inhibition curves of selected inhibitors at soluble and human membrane-bound CD39, proposed binding modes of 8-butylthio-AMP (**1b**) and 8-cyclohexylthio-*N* ^6^-(4-phenylbutyl)­amino-AMP (**42a**), cell viability data, biological assays, synthetic methods, yields, melting points, purities, ^1^H and ^13^C NMR spectra of intermediates (PDF)
Molecular models of human CD39 in complex with PSB-21378 (**42a**) (PDB)
Molecular models of human CD39 in complex with PSB-24379 (**42b**) (PDB)

## Abbreviations

8-BuS-AMP: 8-butylthio-AMP
ARL 67156: *N* ^6^-diethyl- *d* -β,γ-dibromomethylene-ATP
CD39: nucleoside triphosphate diphosphohydrolase-1 (NTPDase1)
CD73: ecto-5′-nucleotidase
CE: capillary electrophoresis
DAD: diode array detector
DCC: *N,N′*-dicyclohexylcarbodiimide
DMSO-*d*_6_: deuterated dimethyl sulfoxide
EtOH: ethanol
HEPES: 4-(2-hydroxyethyl)­piperazine-1-ethanesulfonic acid
HMDS: hexamethyldisilazane
HOBt: 1-hydroxybenzotriazole
LIF: laser-induced fluorescence
mCPBA: meta-chloroperoxybenzoic acid
MeOH: methanol
mp: melting point
NaOMe: sodium methoxide
NPP1: nucleotide pyrophosphatase/phosphodiesterase1
proton-sponge: 1,8-bis­(dimethylamino)­naphthalene
PSB: Pharmaceutical Sciences Bonn
satd.: saturated
SEM: standard error of the mean
TEAC: triethylammonium hydrogen carbonate
TMSBr: trimethylsilyl bromide.
