# Supplementary material for: Nucleotide-Derived Competitive Inhibitors of Ectonucleotidase CD39—A Promising Extracellular Target for Immunotherapy of Cancer
Source: J Med Chem. 2026 May 1;69(9):10828–64. doi: 10.1021/acs.jmedchem.6c00054 (PMC13181798; doi:10.1021/acs.jmedchem.6c00054)
Supplement: Supplementary file 1 [file jm6c00054_si_001.pdf]

Supporting Information

Nucleotide-derived Competitive Inhibitors of Ectonucleotidase CD39 - A Promising Extracellular Target for Immunotherapy of Cancer

Chunyang Bi,[a] Florian Schwermer,[a] Laura Schäkel,[a] Salahuddin Mirza,[a] Helay Baburi,[a] Patrick Riziki,[a] Constanze C. Schmies,[a] Riekje Winzer,[b] Riham Idris,[a] Georg Rolshoven,[a] Julia Schilling,[a] Leon Luckenbach,[a] Julie Pelletier,[c] Luca Svolacchia Brusoni,[a] Haneen Al Hroub,[a] Ghazl Al Hamwi,[a] Vittoria Lopez,[a] Areso Ahmadsay,[a] Luca Raulien,[a] Katharina Sylvester,[a] Jean Sévigny,[c,d] Eva Tolosa,[b] Andreas Guse,[e] and Christa E. Müller[a,*]

[a] PharmaCenter Bonn, Pharmaceutical Institute, Pharmaceutical Sciences Bonn (PSB), Pharmaceutical & Medicinal Chemistry, University of Bonn, 53121 Bonn, Germany
[b] Department of Immunology, University Medical Center Hamburg-Eppendorf, 20246 Hamburg, Germany
[c] Axe maladies infectieuses et immunitaires, Centre de Recherche du CHU de Québec – Université Laval, Québec City, Quebec G1V 4G2, Canada
[d] Départment de Microbiologie-Infectiologie et d'Immunologie, Centres PROTEO-ULaval et ARThrite, Faculté de Médecine, Université Laval, Quebec City, Quebec G1V 0A6, Canada
[e] Calcium Signaling Group, Department of Biochemistry and Molecular Cell Biology, University Medical Center Hamburg-Eppendorf, 20246 Hamburg, Germany

***Correspondence:**
Christa E. Müller
PharmaCenter Bonn, Pharmaceutical Institute, Pharmaceutical Sciences Bonn (PSB), Pharmaceutical & Medicinal Chemistry, University of Bonn, 53121 Bonn, Germany
E-mail: christa.mueller@uni-bonn.de
Tel: +49-228-73-2301
Fax: +49-228-73-2567

Table of Contents

Table S1. Selectivity studies of selected AMP derivatives at human ectonucleotidases S3
Table S2. Potency of AMP derivatives as inhibitors at soluble as compared to membrane-bound human CD39 .... S4
Table S3. Potency of AMP derivatives as inhibitors of soluble human CD73 .... S5
Figure S1. Proposed binding modes of 8-butylthio-AMP (**1b**, blue) and 8-cyclohexylthio-N^6-(4-phenylbutyl)amino-AMP (**42a**, pink) within the substrate binding pocket of the human CD39 homology model based on PDB 3ZX3.[2,3,1] Docking was performed in Schrödinger Maestro and the figure was created using UCSF ChimeraX[4] (see Experimental). S6
Figure S2. Concentration-inhibition curves of 8-cyclohexylthio-N^6-(4-phenylbutyl)amino-AMP (**42a**), 8-naphthylthio-N^6-(4-phenylbutyl)-AMP (**42b**), and 8-butylthio-N^6-(4-phenylbutyl)-AMP (**42e**) at soluble human CD39, and structure of membrane-bound and soluble human CD39 lacking the transmembrane helices. S7
1 **Biology** S8
1.1 **Capillary Electrophoresis Assay for Soluble Human CD39** S8
1.2 CD73 Assay S8
1.3 NPP1 Assay S9
1.4 NPP4 Assay S9
1.5 NPP3 and NPP5 Assays S9
1.6 CD38 Assay S10
1.7 T cell activation and proliferation assay S10
1.8 Flow cytometry S10
1.9 Membrane preparations of human melanoma cells S10
2 **Chemistry** S12
2.1 General Procedures S12
2.2 Synthesis of Intermediates S17
3 **LC-MS spectra of selected AMP derivatives** S72
4 **^{1}H, ^{13}C, and ^{31}P NMR spectra of selected AMP derivatives** S100
5 **References** S125

Table S1. Selectivity studies of selected AMP derivatives at human ectonucleotidases

Enzyme	K_i ± SEM (µM) (or % inhibition at 50 µM)[c]						
	**8r**	**25a**	**31j**	**31r**	**42a**	**42b**	**42e**
CD39[a,b]	**1.40** ± 0.12[a] **3.73** ± 0.39[b]	**0.660** ± 0.072[a] **4.89** ± 1.23[b]	**0.768** ± 0.052[b]	**0.735** ± 0.056[b]	**0.180** ± 0.104 (n = 6)[b]	**0.0774** ± 0.0355 (n = 10)[b]	**0.277** ± 0.074 (n = 6)[b]
CD73	**0.337** ± 0.111	> 50 (28%)	**1.89** ± 0.10	**0.739** ± 0.090	**1.62** ± 0.56	**0.240** ± 0.051	**0.817** ± 0.032
NTPDase2	**1.97** ± 0.62	**529** ± 365	**82.9** ± 17.7	**24.3** ± 5.9	**1.89** ± 1.38	**1.30** ± 0.03	**2.62** ± 0.28
NTPDase3	**60.6** ± 3.5	**65.5** ± 11.0	**54.8** ± 9.2	**4.74** ± 0.45	**0.922** ± 0.278	**1.80** ± 0.40	**29.1** ± 2.3
NTPDase8	> 50 (-11%)	> 50 (-26%)	> 50 (0%)	> 50 (8%)	> 50 (21%)	28.3 ± 3.2	> 50 (38%)
NPP1	**23.7** ± 7.6	**29.5** ± 0.0	> 50 (9%)	> 50 (29%)	≥ 50 (50%)	> 50 (-5%)	> 50 (15%)
NPP3	**112** ± 34	**172** ± 37	> 50 (19%)	> 50 (14%)	**5.99** ± 2.58	> 50 (9%)	> 50 (23%)
NPP4	**24.6** ± 1.1	**35.6** ± 4.0	> 50 (4%)	> 50 (19%)	≥ 50 (50%)	n.d.	> 50 (14%)
NPP5	**99.8** ± 14.6	**207** ± 34	> 50 (18%)	> 50 (10%)	> 50 (34%)	n.d.	> 50 (2%)
CD38	**99.7** ± 13.4	**228** ± 46	> 50 (23%)	≥ 50 (44%)	≥ 50 (50%)	n.d.	≥ 50 (42%)

[a]Fluorescence capillary electrophoresis assay: screening at 10 µM was performed and concentration-inhibition curves were determined for potent inhibitors using the fluorescent substrate PSB-170621A (0.5 µM) and human membrane-bound CD39 (n = 3). [b]Malachite green assay: screening at 10 µM was performed and concentration inhibition curves were determined for potent inhibitors using the natural substrate ATP (50 µM) and human membrane-bound CD39 (n = 3). [c]K_i values of inhibitors for which concentration-dependent inhibition was determined are shown in bold.

Table S2. Potency of AMP derivatives as inhibitors at soluble as compared to membrane-bound human CD39

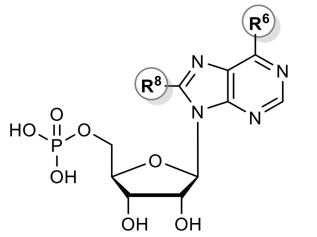

1b, 8r, 25a, 31j, 31r, 42d, 42e

Compound	R^6	R^8	K_i ± SEM (µM)[c]		Ratio
			soluble[a]	membrane-bound[b]	membrane-bound/soluble
**1b** **(8-BuS-AMP)**[1]	NH_2	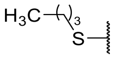	**0.733** ± 0.386	**0.847**± 0.194	1.2
**8r**	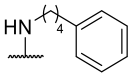	H	**5.93** ± 3.31	**7.08** ± 0.68	1.2
**25a**	NH_2	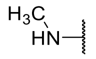	**2.29** ± 0.36	**4.89** ± 1.23	2.1
**31j**	NH_2	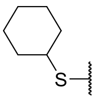	**0.232** ± 0.011	**0.768** ± 0.052	3.3
**31r**	NH_2	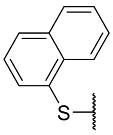	**0.469** ± 0.031	**0.735** ± 0.056	1.6
**42a**	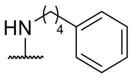	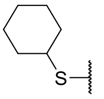	**0.0112** ± 0.0046	**0.180** ± 0.104	16.1
**42b**	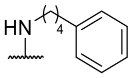	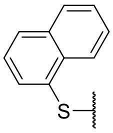	**0.0231** ± 0.0214	**0.0774** ± 0.0355	3.4
**42d**	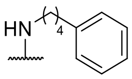	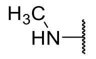	**8.60** ± 1.19	**1.54** ± 0.36	0.2
**42e**	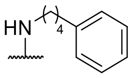	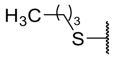	**0.0589** ± 0.0031	**0.277** ± 0.074	4.7

[a]Fluorescence capillary electrophoresis assay: concentration-inhibition curves were determined using the fluorescent substrate PSB-170621A (0.5 µM) and human soluble CD39 (n = 3). [b]Malachite green assay: concentration inhibition curves were determined using the natural substrate ATP (50 µM) and human membrane-bound CD39 (n = 3). [c]K_i values of inhibitors are shown in bold.

Table S3. Potency of AMP derivatives as inhibitors of soluble human CD73

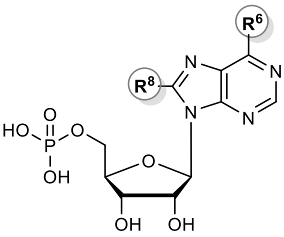

1b,
8r, 8u, 8v,
31d, 31i, 31j, 31r,
42a-e
47a-d,
48a, 48b,
49

Compound	R[6]	R[8]	K_i ± SEM (µM)[a]
**1b** **(8-BuS-AMP)**[1]	NH_2	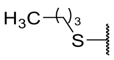	**1.19** ± 0.41
**8r**	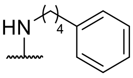	H	**0.337** ± 0.111
**8u**	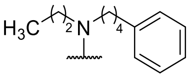	H	**0.857** ± 0.102
**8v**	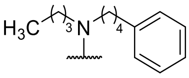	H	**10.3** ± 2.7
**31d**	NH_2	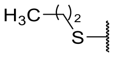	**1.77** ± 0.22
**31i**	NH_2	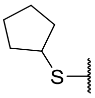	**2.33** ± 0.24
**31j**	NH_2	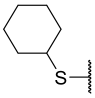	**1.86** ± 0.10
**31r**	NH_2	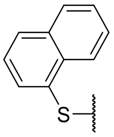	**0.739** ± 0.090
**42a**	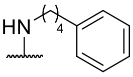	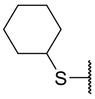	**1.62** ± 0.56
**42b**	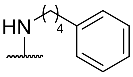	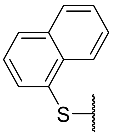	**0.240** ± 0.051
**42c**	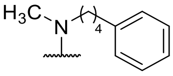	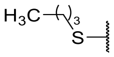	**0.839** ± 0.123
**42d**	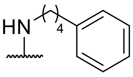	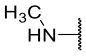	**20.2** ± 5.6
**42e**	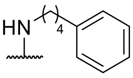	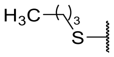	**0.817** ± 0.032
**47a**	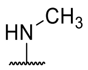	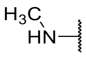	**0.753** ± 0.256

**47b**	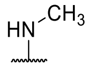	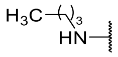	**1.12** ± 0.25
**47c**	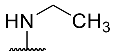	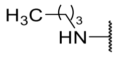	**19.6** ± 11.7
**47d**	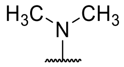	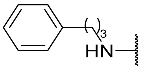	**3.23** ± 0.59
**48a**	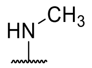	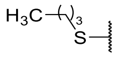	**0.239** ± 0.0230
**48b**	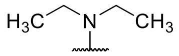	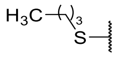	**7.06** ± 1.02
**48c**	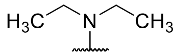	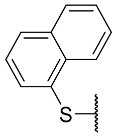	**4.29** ± 0.06
**49**	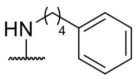	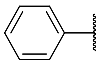	**5.12** ± 0.73

[a]Concentration-inhibition curves were determined using [2,8-^{3}H]AMP (5.0 µM) and soluble human CD73 (n = 3).

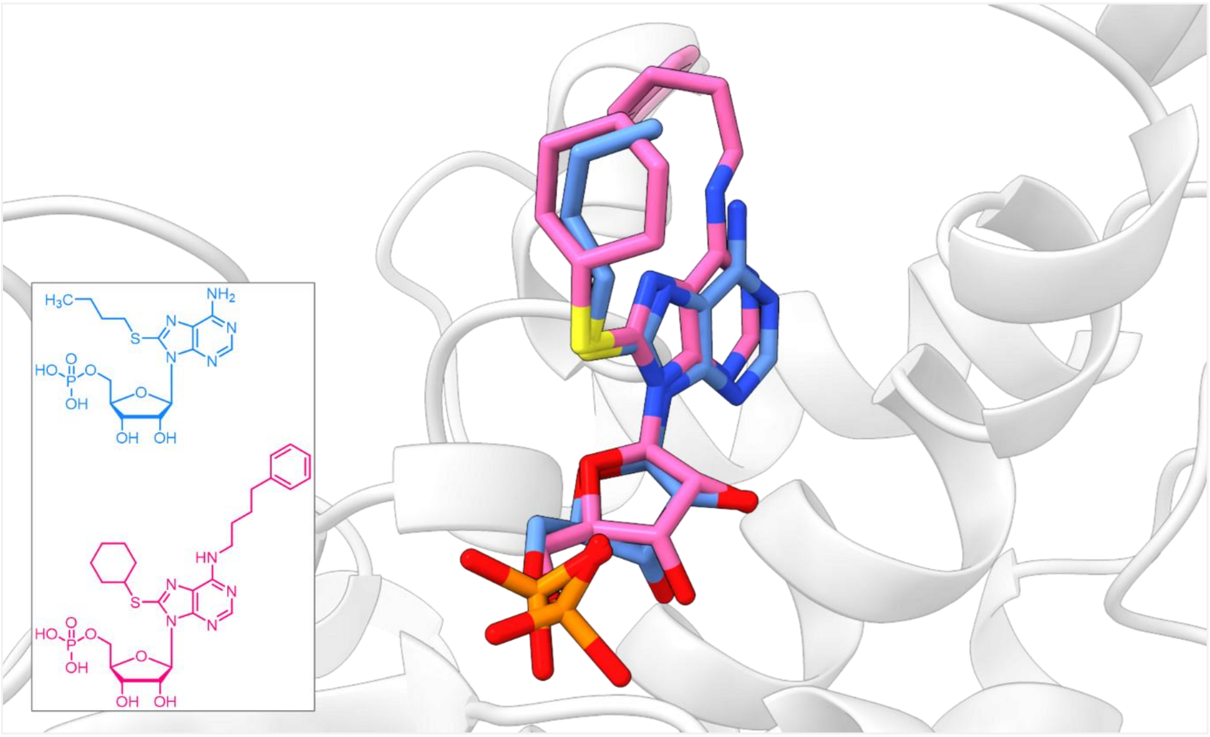

Figure S1. Proposed binding modes of 8-butylthio-AMP (**1b**, blue) and 8-cyclohexylthio-*N*^{6}-(4-phenylbutyl)amino-AMP (**42a**, pink) within the substrate binding pocket of the human CD39 homology model based on PDB 3ZX3.[2,3,1] Docking was performed in Schrödinger Maestro and the figure was created using UCSF ChimeraX[4] (see Experimental).

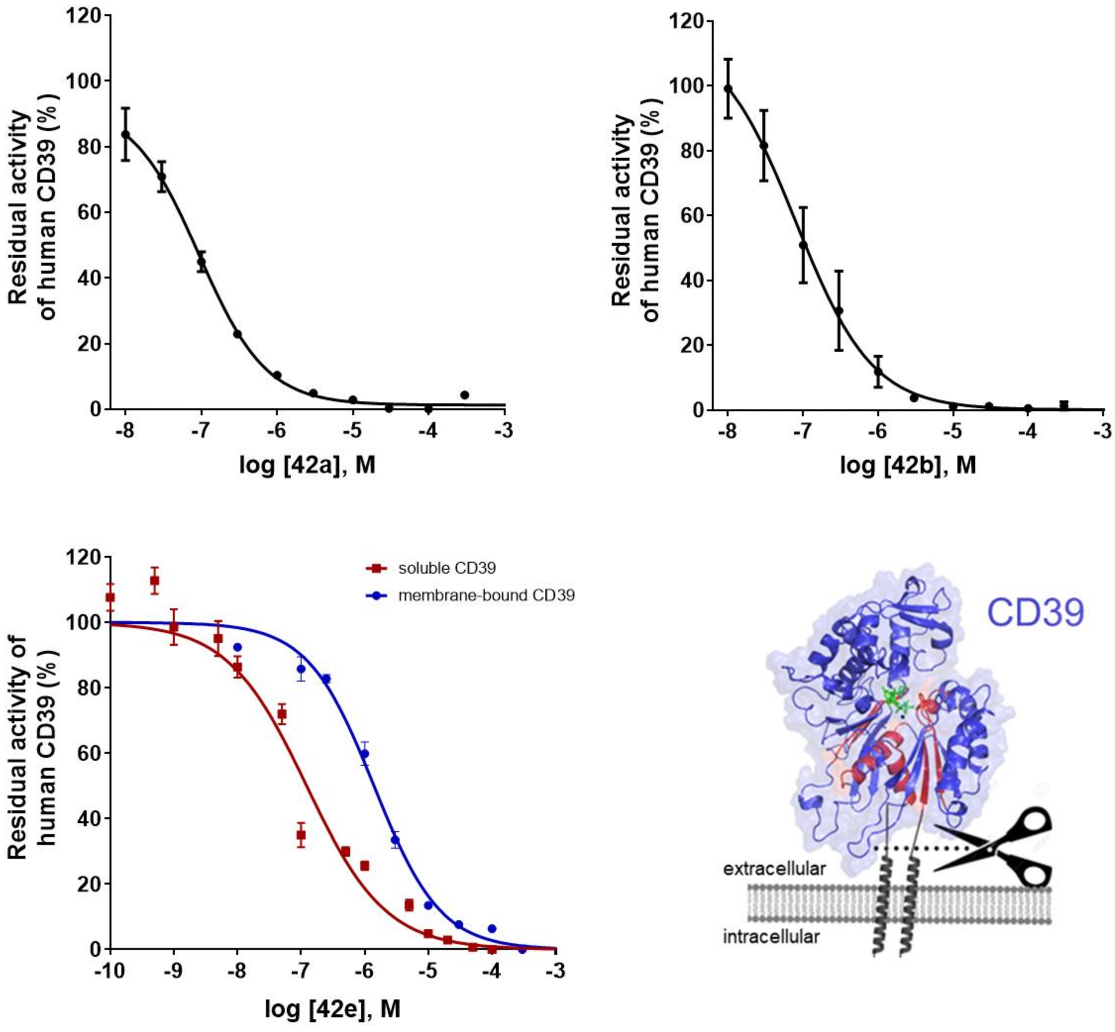

Figure S2. Concentration-inhibition curves of 8-cyclohexylthio-N^6-(4-phenylbutyl)amino-AMP (**42a**), 8-naphthylthio-N^6-(4-phenylbutyl)-AMP (**42b**), and 8-butylthio-N^6-(4-phenylbutyl)-AMP (**42e**) at soluble human CD39, and structure of membrane-bound and soluble human CD39 lacking the transmembrane helices.

1 Biology

1.1 Capillary Electrophoresis Assay for Soluble Human CD39

The enzyme activity experiments were performed as previously described.[3] The test compounds were initially investigated at a concentration of 50 µM (n = 3), 100 µM ATP (K_m = 67.9 µM), and 150 ng human recombinant soluble CD39 were added to initiate the reaction. The reaction buffer contained 10 mM HEPES, 2 mM $CaCl_2$, 1 mM $MgCl_2$, pH 7.4 in a final volume of 100 µL. Incubation at 37 °C for 30 min, followed by termination of the enzymatic reaction by heating at 90 °C for 5 min. The samples were then diluted 1:20 with reaction buffer to perform separation of nucleotides by capillary electrophoresis. Reaction with soluble CD39 was conducted using DAD-detector with an absorbance maximum of 254 nm. Concentration-inhibition curves were generated at concentrations ranging from 0.01 to 300 µM (n = 3), plotted with GraphPad Prism 7 software and the K_i value was calculated using the Cheng-Prusoff equation for competitive inhibitors.

Analysis was carried out using a P/ACE MDQ capillary electrophoresis system (Beckman Instruments, Fullerton, CA, USA) using a *polyacrylamide*-coated capillary [30 cm (10 cm effective length) × 50 µm (id), × 360 µm (od)]. Before each run, the capillary was rinsed with the background electrolyte (50 mM phosphate buffer, pH 6.5) for 1 min at 30 psi. Electrokinetic injection of samples by applying a voltage of -6 kV for 30 s at the capillary outlet, and separation of the fluorescent nucleotide derivatives by voltage application of -15 kV. Detection was performed at an excitation wavelength of 488 nm and an emission wavelength of 520 nm. Data collection and peak area analysis were performed by the P/ACE MDQ software 32 KARAT obtained from Beckman Coulter (Fullerton, CA, USA).

1.2 CD73 Assay

The assay was performed as previously described.[5] Briefly, the assay was performed with 0.09 µg/mL of soluble human CD73,[6] the respective test compound, and 5.0 µM [2,8-^{3}H]AMP (specific activity 7.4 x 108 Bq/mmol, 20 mCi/mmol) as radioactive substrate in assay buffer consisting of 25 mM Tris buffer, 140 mM NaCl, 25 mM NaH_2PO_4 pH 7.4. The enzymatic reaction was performed for 25 min at 37 °C in a shaking water bath. Then, 500 µL of cold precipitation buffer (100 mM $LaCl_3$, 100 mM sodium acetate, pH 4.0) were added to precipitate free phosphate and unconverted [2,8-^{3}H]AMP. After 30 min on ice, filtration through GF/B glass fiber filters using a cell harvester was used to separate AMP from adenosine. After washing each reaction vial three times with 400 µl of cold (4°C) demineralized water, aliquots of the filtrate were taken, and 5 ml of the scintillation cocktail (ULTIMA Gold XR9) was added. The

amount of formed adenosine was quantified by liquid scintillation counting (TRICARB 2900 TR, Packard/PerkinElmer).

1.3 NPP1 Assay

Inhibition of NPP1 was determined as previously described.[7] *p*-Nitrophenyl-5'-thymidine monophosphate (*p*-Nph-5'-TMP) was used as an artificial substrate which results in the formation of the *p*-nitrophenolate anion with an absorption maximum of 400 nm. Purified soluble NPP1 (0.36 µg, expressed in insect cells) was mixed with test compound (20 µM final concentration for initial screening, 0.1 – 200 µM for determining concentration-dependent inhibition curves), 2% DMSO and 400 µM of *p*-Nph-5'-TMP as a substrate in a final volume of 100 µL. The mixture was incubated for 30 min at 37°C with gentle shaking, and the enzyme reaction was terminated by the addition of 20 µL of 1 M NaOH. The absorption was measured at 405 nm using a BMG PheraStar FS plate reader (BMG Labtech GmbH, Ortenberg, Germany).

1.4 NPP4 Assay

Diadenosine tetraphosphate (AP_4A) was employed as a substrate which is cleaved by NPP4 to ATP and AMP. The reaction product ATP was quantified by luciferin-luciferase reaction.[8] A mixture of 1.4 µg of NPP4 (soluble form expressed in insect cells and purified), 10 µM of test compound, 2% DMSO and 20 µM of AP_4A as a substrate were incubated for 60 min at 37 °C with gentle shaking. The reaction was terminated by heating at 90°C for 5 min, and after cooling down on ice, 50 µl of D-luciferin dissolved in buffer (300 mM Tris-HCl, 15 mM $MgCl_2$, 100 ng D-luciferin, pH 7.8) and 50 µl luciferase (50 ng dissolved in H_2O) were added. The firefly luciferase reacts with D-luciferin in the presence of ATP produced by NPP4. The resulting luminescence was measured between 10-14 min at 560 nm using a BMG PheraStar FS plate reader (BMG Labtech GmbH, Ortenberg, Germany).

1.5 NPP3 and NPP5 Assays

The assays were performed in analogy to published procedures.[9] The enzymatic activity of human NPP3 and NPP5 (soluble forms expressed in insect cells and purified) was measured using 1,N^6-etheno-nicotinamide adenine dinucleotide (ε-NAD^+) as a substrate, which is hydrolyzed to fluorescent 1,N^6-etheno-AMP (ε-AMP). The enzymatic reactions were performed in reaction buffer (10 mM *N*-cyclohexyl-2-aminoethanesulfonic acid (CHES), 2 mM $CaCl_2$, and 1 mM $MgCl_2$, pH 9.0 in H_2O). Purified NPP3 (90 ng) or NPP5 (400 ng), 20 µM of *ε*-NAD^+ and 10 *µ*M of the test compound were incubated for 30 min at 37°C. The relative fluorescence at 270 nm excitation and 420 nm emission was detected by a fluorescence microplate reader (Flexstation, Medical Devices LLC. USA, Softmax Pro software to collect the data).

1.6 CD38 Assay

The assay operation was analogous to the NPP3 and NPP5 assays. The enzymatic reactions were performed in 10 mM HEPES reaction buffer (pH 7.2) using 8 ng of human CD38 (soluble form expressed in insect cells).

1.7 T cell activation and proliferation assay

PBMCs were isolated from buffy coats that were obtained from the blood bank of the University Medical Center Hamburg-Eppendorf (UKE). For measuring T cell proliferation, PBMCs were labeled with the proliferation dye eFluor 670. Briefly, cells were centrifuged (450 × g, 5 min) and resuspended in a 2 µM eFluor 670 working solution (100 µL per 1 × 10^6 cells; Thermo Fisher Scientific). The suspension was incubated for 10 min at 37 °C, after which labeling was stopped by adding chilled complete RPMI medium (RPMI supplemented with 10% FBS, 1% L-glutamine, and 1% penicillin-streptomycin) at fivefold the previous volume. The cells were then placed on ice for 5 min and washed twice with full RPMI. T cells were isolated from PBMCs by negative selection using the EasySep Human T Cell Enrichment Kit (Stemcell Technologies). T cells were stimulated with plate-bound αCD3 (1 µg/mL) and soluble αCD28 (5 µg/mL; both from BioLegend) in the presence of the adenosine deaminase inhibitor EHNA (10 µM; Tocris) in serum-free X-VIVO 15 medium (Lonza). ATP (50 µM; Sigma-Aldrich) and the respective AMP-derived inhibitor were added at the onset of stimulation. Cultures were maintained for four days at 37 °C in a humidified incubator with 5% CO_2. Following incubation, eFluor 670 dilution (as a proliferation indicator) and CD25 surface expression (as an activation marker) were quantified by flow cytometry.

Flow cytometry

For flow cytometric analysis, cells were stained for 30 min at 4 °C with fluorochrome-conjugated antibodies specific for human CD25 (clone BC96, BV421), CD8 (clone RPA-T8, BV510), and CD4 (clone RPA-T4, FITC) (all from BioLegend). To exclude nonviable cells, an amine-reactive viability dye (Alexa Fluor 750 succinimidyl ester, 1 µg/mL; Thermo Fisher Scientific) was included during antibody incubation. The antibody panel was optimized to minimize spectral overlap, and fluorescence compensation was established using single-stained human peripheral blood mononuclear cells (PBMC controls. Samples were acquired on a FACSCanto II flow cytometer (BD Biosciences) equipped with violet (405 nm), blue (488 nm), and red (633 nm) lasers, and analyzed with FlowJo software (BD Biosciences).

1.8 Membrane preparations of human melanoma cells

Membrane preparations from Ma-Mel-65 melanoma cells were generated from cultures expanded to approximately 90% confluency. Cells from a single 175 cm² flask were distributed into twenty 150 mm culture dishes and maintained at 37 °C under humidified conditions with

5% CO_2. Upon reaching ~90% confluency, culture medium was removed, cells were rinsed with phosphate-buffered saline (PBS), and the plates were stored at −20 °C until further processing. Frozen cells were harvested by scraping into 1 mL of ice-cold buffer (25 mM Tris-HCl, 1 mM EDTA, 0.32 M sucrose, 100 µM phenylmethylsulfonyl fluoride (PMSF); pH 7.4). The scraping step was repeated to maximize cell recovery, after which the pooled suspensions were homogenized using an Ultraturrax homogenizer (two 30 s cycles at high speed). Nuclei, large organelles, and cell debris, were removed by subsequent centrifugation of the homogenate at 1000 × g for 10 min at 4°C. The resulting supernatant was collected and further centrifuged at 48,000 × g for 1 h at 4°C. The obtained membrane pellets were washed once with the same buffer under identical centrifugation conditions. After two additional washing steps, the final pellets were resuspended in 0.1 mL of 50 mM Tris-HCl buffer (pH 7.4) per culture dish. The crude membrane preparations were stored at −80°C until use. All steps were performed rapidly and maintained at 4°C to preserve enzymatic activity and prevent internalization. Protein concentrations were determined using the Lowry method, yielding 9.1 µg/µL for melanoma membrane preparations.

2 Chemistry

2.1 General Procedures

General procedure for the synthesis of *N*⁶-substituted nucleosides 7a-w, 14a-d.

To a solution of 6-chloro-9-(*β-D*-ribofuranosyl)purine (**6**, 500 mg, 1.74 mmol, 1 equiv.) in EtOH (10 mL), appropriate alkyl or aryl amines (1.5 equiv.) and Et_3N (2 equiv.) were added. The mixture was refluxed for 3 h or overnight and monitored by TLC (MeOH/DCM 1:9). After the reaction was completed, cooled to rt and the solvent was evaporated *in vacuum*. The crude compound was purified by silica gel column chromatography (2-10% MeOH in DCM).

General procedure for the synthesis of *N*-substituted 4-phenylbutan-1-amines 10c-e.

To a solution of 1-bromo-4-phenylbutane (1 equiv.) in MeOH (or only appropriate amine solution, 10 mL), appropriate amine (5 equiv.) was added. The mixture was refluxed overnight and monitored by TLC (MeOH/DCM 1:9). After the reaction was completed, cooled to rt and the solvent was evaporated *in vacuum*. The crude compound was purified by silica gel column chromatography (4% MeOH in DCM).

General procedure for the synthesis of 3-(3-methoxyphenyl)propan-1-amine (13b) or 3-(4-methoxyphenyl)propan-1-amine (13c).

A solution of propionic acid (**11a** or **11b**, 1 equiv.) and 4-methylmorpholine (1.1 equiv.) in THF (20 mL) was cooled to 0 °C, then *iso*-butyl chloroformate (1.1 equiv.) was added slowly. After 30 min of stirring at 0 °C, a 7 M solution of NH_3 in MeOH (2 equiv.) was added dropwise. The mixture was allowed to warm up to rt and stirred for 2 h. The reaction was quenched by 10% aqueous K_2CO_3. The crude product was extracted with ethyl acetate (3 × 50 mL). The organic layers were combined, washed with H_2O and brine, and dried over $MgSO_4$, followed by filtration and evaporation to dryness to get **12a** or **12b**.

Then to the intermediate **12a** or **12b** (1 equiv.) in THF (50 mL) at 0 °C, lithium aluminum hydride (2 equiv.) was added carefully. The mixture was refluxed for 30 min and monitored by TLC (MeOH/DCM, 1:3). After the reaction was completed, it was quenched carefully with sequential addition of H_2O (10 mL), 15% aqueous NaOH (40 mL), followed by extraction with diethyl ether (3 × 50 mL). The organic layers were combined, dried over Na_2SO_4, and concentrated *in vacuum*, followed by purification by silica gel column chromatography (10% MeOH in DCM).

General procedure for the synthesis of 2′,3′-isopropylidene-nucleosides 15a-e.

To a solution of nucleoside (1 equiv.) in acetone (20 mL), 2,2-dimethoxypropane (5 equiv.) and H_2SO_4 (1.7 equiv.) were added. The reaction mixture was stirred at rt for 15-30 min and monitored by TLC (MeOH/DCM, 1:9). After the reaction was completed, Et_3N was added dropwise till the pH = 7, then the solvent was evaporated *in vacuum*. The crude compound was purified by silica gel column chromatography (2% MeOH in DCM).

General procedure for the synthesis of 6-thio substituted nucleosides 20b-c.

To a solution of 6-chloro-9-(*β-D*-ribofuranosyl)purine (**6**, 400 mg, 1.40 mmol, 1 equiv.) in 10 mL EtOH, NaOMe (6 equiv.) and appropriate thiol compound (6 equiv.) were added. The mixture was refluxed overnight and monitored by TLC (MeOH/DCM, 1:9). After the reaction was completed, cooled to rt and the solvent was evaporated *in vacuum*. The crude compound was purified by silica gel column chromatography (6% MeOH in DCM).

General procedure for the synthesis of 8-amino substituted nucleosides 24a-d, 44a-f

To 8-bromonucleoside **23** or **43a-d** (1 equiv.), an appropriate (aqueous) amine solution (10 mL), Et_3N (20 equiv.) was added. The mixture was refluxed for 12-36 h and monitored by TLC (MeOH/DCM 1:9). After the reaction was completed, cooled to rt and the solvent was evaporated *in vacuum*. The crude compound was purified by silica gel column chromatography (8-20% MeOH in DCM).

General procedure for the synthesis of 8-oxy substituted nucleosides 26b-c.

To **23** (1 equiv.) in an 8 mL appropriate alkyl alcohol solution, NaOH (3 equiv.) was added. The mixture was stirred at 50 °C for 2 h and monitored by TLC (MeOH/DCM 1:9). After the reaction was completed, cooled to rt, 10 g silica gel was added, and the mixture was evaporated *in vacuum*. The crude compound was purified by silica gel column chromatography (10% MeOH in DCM).

General procedure for the synthesis of 8-thio substituted nucleosides 30a and 30h.

To a solution of 8-thioadenosine (**29**, 1 equiv.) in 10 mL H_2O/EtOH (1:1), the appropriate alkylhalide (3 equiv.) was added and the solution was basified slightly with 2 M NaOH. The reaction was refluxed for 1-30 h and monitored by TLC (MeOH/DCM 1:9). After the reaction was completed, cooled to rt and the mixture was evaporated *in vacuum*. The crude compound was purified by silica gel column chromatography (6% MeOH in DCM).

General procedure for the synthesis of 8-thio substituted nucleosides 30c-g, 30j, 30l-r, and 45a-c.

To a solution of **23**, **43a** or **43d** (1 equiv.) in 10 mL EtOH, NaOMe (3-6 equiv.) and appropriate thiol compound (2-12 equiv.) were added. The mixture was refluxed for 3 h or overnight and

monitored by TLC (MeOH/DCM 1:9). After the reaction was completed, cooled to rt, 10 g silica gel was added, and the solvent was evaporated *in vacuum*. The crude compound was purified by silica gel column chromatography (8% MeOH in DCM).

General procedure for the synthesis of 8-thio substituted nucleosides 30i and 30k.

To a solution of **23** (1 equiv.) in EtOH (10 mL), thiourea (2 equiv.) was added, and the mixture was refluxed overnight. Then bromocyclopentane or cyclohexylmethyl bromide (2 equiv.) and H_2O (10 mL) were added and basified slightly with 2 M NaOH. The mixture was refluxed for 3 h and monitored by TLC (MeOH/DCM 1:9). After the reaction was completed, cooled to rt, 10 g silica gel was added, and the solvent was evaporated *in vacuum*. The crude compound was purified by silica gel column chromatography (7% MeOH in DCM).

General procedure for the synthesis of nucleosides 32c and 32f.

To a solution of **32b** or **32d** (1 equiv.) in MeOH/THF (1:1, 10 mL), 20 mg 10 wt. % Pd/C (10%) was added. The mixture was shaken with hydrogen (45 psi) for 2 h at rt in a Parr apparatus and the reaction progress was monitored by TLC (MeOH/DCM 1:9). After the reaction was completed, the suspension was filtered on celite. The filter cake was washed with MeOH/THF (1:1, 20 mL) and evaporated *in vacuum*. The crude compound was purified by silica gel column chromatography (7% MeOH in DCM).

General procedure for the synthesis of 8-thio substituted nucleosides 32d-e by Sonogashira coupling.

To a solution of **23** (1 equiv.) in anhydrous DMF (10 mL) under argon, 4% Pd $(PPh_3)_2Cl_2$, 8% CuI, Et_3N (4 equiv.) and 1-hexyne or 1-pentyne (5 equiv.) were added. The mixture was stirred at 100 °C under argon for 6 h and monitored by TLC (MeOH/DCM 1:9). After the reaction was completed, cooled to rt and the mixture was concentrated *in vacuum*. The residue was dissolved in $CHCl_3$ (150 mL), the organic layer was washed with H_2O (70 mL), brine (70 mL) and dried over anhydrous Mg_2SO_4. The solvent was then evaporated *in vacuum*. The crude compound was purified by silica gel column chromatography (6% MeOH in DCM).

General procedure for the bromination of the N^6-position of 8-thio substituted nucleosides (38a-c).

To a solution of 8-thio-substituted adenosine (1 equiv.) in CH_2Br_2 (10 mL), TMSBr (5 equiv.) and *tert*-butyl nitrite (10 equiv.) were added. The mixture was stirred overnight increasing the temperature from 0 °C to rt under argon and the reaction progress was monitored by TLC (MeOH/DCM, 1:9). After the reaction was completed, H_2O (20 mL) was added to quench the reaction. The mixture was extracted with EtOAc (100 mL × 2) and $CHCl_3$ (100 mL). The

collected organic layers were dried over $MgSO_4$ and evaporated *in vacuum*. The crude compound was purified by silica gel column chromatography (4% MeOH in DCM).

General procedure for the synthesis of 2′,3′,5′-tri-*O*-acetyl-nucleosides 39a-b.

To a solution of 8-substituted nucleoside **24a** or **37** (1 equiv.) in MeCN (10 mL), acetic anhydride (3 equiv.), DMAP (4-dimethylaminopyridine, 0.1 equiv.) and DMEA (*N*,*N*-dimethylethylamine, 4 equiv.) were added. The mixture was stirred for 15 min and monitored by TLC (MeOH/DCM, 1:9). After the reaction was completed, the solvent was evaporated *in vacuum*. The crude compound was purified by silica gel column chromatography (3% MeOH in DCM).

General procedure for the synthesis of 6-bromo-2′,3′,5′-tri-*O*-acetyl-nucleosides 40a-b.

To a solution of **39a** or **39b** (1 equiv.) in CH_2Br_2 (5 mL), $SbBr_3$ (1 equiv.), BTEA-Br (benzyltriethylammonium bromide, 1.5 equiv.), $NaNO_2$ (20 equiv.), DCA (dichloroacetic acid, 1.5 equiv.) and AcOH (0.5 equiv.) were added. The mixture was stirred at rt overnight under argon and monitored by TLC (MeOH/DCM, 1:9). After the reaction was completed, celite (3 g) and $CHCl_3$ (10 mL) were added, and the suspension was stirred for 10 min. The mixture was filtered, and the filter cake was washed with $CHCl_3$ (120 mL). The filtrate was then evaporated *in vacuum*, and the crude compound was purified by silica gel column chromatography (1% MeOH in DCM).

General procedure for the amination of the N^6 position of 8-thio-substituted and N^6-brominated nucleosides (41a-c).

To a solution of N^6-bromo-8-thio-substituted nucleoside (1 equiv.) in absolute EtOH (10 mL), 4-phenylbutylamine or methyl(4-phenylbutyl)amine (1.5 equiv.) and Et_3N (0.48 mL, 3.48 mmol, 2 equiv.) were added. The mixture was refluxed for 3 h and monitored by TLC (MeOH/DCM 1:9). After the reaction was completed, cooled to rt and the solvent was evaporated *in vacuum*. The crude compound was purified by silica gel column chromatography (5% MeOH in DCM).

General procedure for the synthesis of N^6,8-disubstituted AMP derivatives 41a-e.

To a solution of **38a-c**, **40a,** or **40b** (1 equiv.) in absolute EtOH (10 mL), 4-phenylbutylamine or methyl(4-phenylbutyl)amine (1.5-2 equiv.) and Et_3N (2 equiv.) were added. The mixture was refluxed for 3 h and monitored by TLC (MeOH/DCM, 1:9). After the reaction was completed, cooled to rt and the solvent was evaporated *in vacuum*. The acetyl-protected nucleosides **40a** and **40b** were treated with 20% NaOMe in MeOH (10 mL) and stirred at rt for 48 h, monitored by TLC (MeOH/DCM, 1:9). After the reaction was completed, 10 g silica gel was added, and

the solvent was evaporated *in vacuum*. The crude mixture was purified by silica gel column chromatography (4% MeOH in DCM).

General procedure for the synthesis of 8-bromonucleosides (43a-d).

To the solution of adenosine or N^6-substituted adenosine derivatives (1 equiv.) in 1 M sodium acetate buffer (pH = 4, 5 mL) and H_2O (15 mL), Br_2 (2.5 equiv.) was added dropwise. The mixture was stirred at rt overnight and monitored by TLC (MeOH/DCM, 1:9). After the reaction was completed, the mixture was decolorized by the addition of 1 M $NaHSO_3$, and then neutralized with 2 M NaOH. The solvent was evaporated *in vacuum*, and the crude compound was purified by silica gel column chromatography (5% MeOH in DCM).

General procedure for the synthesis of 1,N^6-etheno-AMP derivatives (60a-b).

To a solution of appropriate adenosine derivative (1 equiv.) in 2 M aqueous chloroacetaldehyde (10 mL), CH_3CO_2Na (5 equiv.) was added. The mixture was stirred at 50 °C for 5 h and the reaction progress was monitored by TLC (MeOH/DCM, 1:9). After the reaction was completed, the solvent was evaporated *in vacuum.* Then the crude product was suspended in MeOH (10 mL), 5 g silica gel was added, and the mixture was evaporated *in vacuum*. Finally, the mixture was purified by silica gel column chromatography (12% or 4% MeOH in DCM).

2.2 Synthesis of Intermediates

(2*R*,3*R*,4*S*,5*R*)-2-(6-Amino-2-hydrazineyl-9*H*-purin-9-yl)-5-(hydroxymethyl)tetrahydro-furan-3,4-diol (2c)

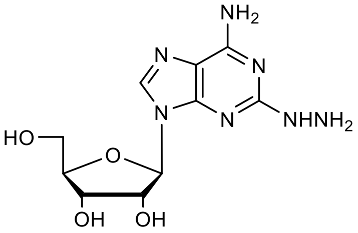

2-Chloroadenosine (**2b**, 500 mg, 1.70 mmol) was dissolved in hydrazine hydrate (7.50 mL) and the reaction was stirred at rt for 8 h. The reaction mixture was diluted with 2-propanol (10 mL). After evaporation, the residue was taken up in H_2O followed by lyophilization. Purification by silica gel column chromatography (25% MeOH in DCM) yielded the desired product (490 mg, 100%), mp: 162 °C. ^{1}H-NMR (500 MHz, DMSO-*d*$_6$) *δ* 7.93 (s, 1H), 7.21 (s, 1H), 6.82 (s, 2H), 5.77 (d, 1H, *J* = 6.29 Hz), 5.31 (br s, 1H), 5.17 (br s, 1H), 5.11 (br s, 1H), 4.58 (t, 1H, *J* =,5.63 Hz), 4.13 (m, 1H), 3.91 (q, 1H, *J* = 3.76 Hz), 3.65-3.52 (d m, 2H). ^{13}C-NMR (125 MHz, DMSO-*d*$_6$) *δ* 162.07, 156.4, 151.31, 136.76, 114.27, 87.27, 85.56, 73.23, 70.85, 61.85. LC/ESI-MS (m/z): positive mode 298.2 $[M + H]^+$. Purity by HPLC-UV (254 nm)-ESIMS: 99.5%.

(2*R*,3*R*,4*S*,5*R*)-2-(6-Amino-2-mercapto-9*H*-purin-9-yl)-5-(hydroxymethyl)tetrahydro-furan-3,4-diol (2d)

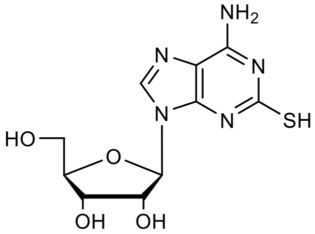

To a solution of adenosine (**4**, 5.00 g, 3.70 mmol) in glacial CH_3COOH (50 mL), a 35% aqueous H_2O_2 (5 mL) was added. The mixture was stirred at 50 °C overnight. Active carbon (10.00 g) was added, and the mixture was stirred at 50 °C until the reaction mixture was free of peroxide as detected by MQuan™ peroxide test strips. The active carbon was removed by filtration followed by evaporation of the filtrate to dryness. The remaining residue was co-evaporated with H_2O repeatedly and finally dissolved in H_2O. The resulting precipitate was collected by filtration and dissolved in 5 M NaOH (45 mL). The mixture was refluxed for 15 min, cooled to rt and adjusted pH to 9.0 with concentrated HCl. After evaporation to a small volume, formed NaCl salt was removed by filtration and washed with MeOH. This process was repeated followed by evaporation to dryness which afforded an amber-colored gum. The residue was dissolved in a mixture solution of $H_2O/CH_3OH/CS_2$ (1:7:2, 150 mL) and was autoclaved at 120 °C. After 5 h, the reaction mixture was cooled down and the resulting precipitate was filtered

and washed with H_2O and MeOH yielding the desired product as yellow powder (1.40 g, 26%), mp: 207 °C. ^{1}H-NMR (500 MHz, DMSO-d_6) δ 11.90 (br s, 1H), 8.21 (s, 1H), 5.75 (d, 1H, J = 5.92 Hz), 4.45 (t, 1H, J = 5.10 Hz), 4.09 (br s, 1H), 3.92 (br s, 1H), 3.64-3.52 (dd, 2H, J = 11.97, 64.49 Hz). ^{13}C-NMR (125 MHz, DMSO-d_6) δ 173.91, 161.60, 155.94, 139.82, 112.79, 87.20, 85.93, 73.80, 70.59, 61.58. LC/ESI-MS (m/z): positive mode 300.0 $[M + H]^+$. Purity by HPLC-UV (254 nm)-ESI-MS: 82%.

(2*R*,3*R*,4*S*,5*R*)-2-(6-Amino-2-(methylthio)-9*H*-purin-9-yl)-5-(hydroxymethyl)tetrahydro-furan-3,4-diol (2e)

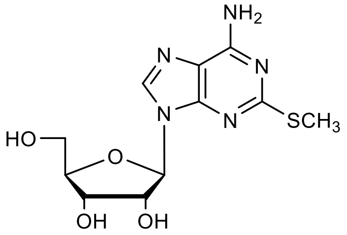

2-Thioadenosine (**2d**, 500 mg, 1.70 mmol) was resuspended in a mixture of H_2O/EtOH (1:1). The solution was basified with 0.5 M NaOH (5 mL). CH_3I (0.30 mL, 5.00 mmol) was added, and the reaction was stirred at rt for 20 min. Addition of ethyl acetate resulted in precipitation. Filtration yielded the desired product as white powder (500 mg, 100%), mp: 247 °C. ^{1}H-NMR (500 MHz, DMSO-d_6) δ 8.21 (s, 1H), 7.31 (s, 2H), 6.09 (d, 1H, J = 5.89 Hz), 4.55 (t, 1H, J = 5.48 Hz), 4.13 (dd, 1H, J = 3.72, 4.90 Hz), 3.90 (q, 1H, J = 4.14 Hz), 3.63-3.51 (d m, 2H), 2.46 (s, 3H). ^{13}C-NMR (125 MHz, DMSO-d_6) δ 164.28, 155.58, 150.31, 138.39, 117.01, 87.55, 85.64, 73.63, 70.57, 61.78, 13.80. LC-MS (m/z): positive mode 314.2 $[M + H]^+$. Purity by HPLC-UV (254 nm)-ESI-MS: 90%.

(3a*R*,4*R*,6*R*,6a*R*)-4-(6-Amino-2-(methylthio)-9*H*-purin-9-yl)-6-(hydroxymethyl)tetra-hydrofuro[3,4-*d*][1,3]dioxole-2,2-diol (5)

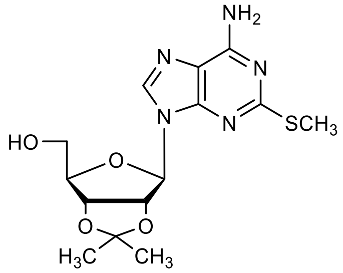

The compound was synthesized using **2e** (500 mg, 1.67 mmol) and afforded an orange-yellow oil (640 mg, >100%). ^{1}H-NMR (500 MHz, DMSO-d_6) δ 8.18 (s, 1H) 7.35 (br s, 2H), 6.09 (d, 1H, J = 2.70 Hz), 5.41 (dd, 1H, J = 2.70, 6.22 Hz), 4.96 (m, 1H), 4.15 (m, 1H), 4.05 (q, 1H, J = 5.26 Hz), 3.54 (m, 2H), 2.47 (s, 3H), 1.53 (s, 3H), 1.32 (s, 3H). ^{13}C-NMR (125 MHz, DMSO-d_6) δ 164.53, 155.58, 149.66, 138.98, 116.88, 113.11, 89.21, 86.72, 83.24, 81.46, 61.57, 27.11,

25.26. LC/ESI-MS (m/z): positive mode 354.0 [M + H]$^+$. Purity by HPLC-UV (254 nm)-ESI-MS: 98.4%.

(2*R*,3*S*,4*R*,5*R*)-2-(Hydroxymethyl)-5-(6-(methylamino)-9*H*-purin-9-yl)tetrahydrofuran-3,4-diol (7a)

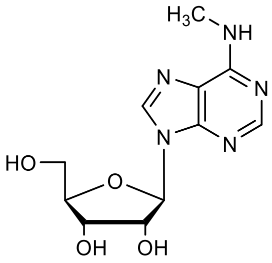

The compound was synthesized using 33% aqueous methylamine (0.10 mL, 2.40 mmol, 1.4 equiv.) and afforded a white solid (700 mg, >100%), mp: 132 °C (*lit.*[10] 130-132 °C). ^{1}H-NMR (500 MHz, DMSO-d_6) δ 8.32 (s, 1H), 8.21 (br s, 1H), 7.77 (br s, 1H), 5.87 (d, 1H, J = 6.17 Hz), 5.40 (br s, 1H), 5.14 (br s, 1H), 4.59 (t, 1H, J = 5.33 Hz), 4.14 (dd, 1H, J = 3.21, 4.75 Hz), 3.95 (q, 1H, J = 3.51 Hz), 3.66-3.54 (d m, 2H), 3.05 (m, 3H). ^{13}C-NMR (125 MHz, DMSO-d_6) δ 156.52, 152.46, 148.22, 139.74, 119.98, 88.05, 86.02, 73.65, 70.77, 61.79, 24.44. LC/ESI-MS (m/z): positive mode 282.3 [M + H]$^+$. Purity by HPLC-UV (254 nm)-ESI-MS: 99.3%.

(2*R*,3*R*,4*S*,5*R*)-2-(6-(Ethylamino)-9*H*-purin-9-yl)-5-(hydroxymethyl)tetrahydrofuran-3,4-diol (7b), CAS: 14357-08-5

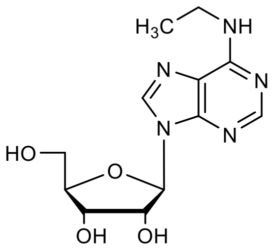

The compound was synthesized using 70% aqueous ethylamine (0.10 mL, 1.76 mmol, 1.0 equiv.) and afforded a white solid (600 mg, >100%), mp: 159 °C (*lit.*[11] 191-192 °C). ^{1}H-NMR (500 MHz, DMSO-d_6) δ 8.32 (s, 1H), 8.18 (br s, 1H), 7.81 (br s, 1H), 5.87 (d, 1H, J = 6.16 Hz), 5.40 (d, 1H, J = 6.22 Hz), 5.14 (d, 1H, J = 4.55 Hz), 4.59 (q, 1H, J = 5.97 Hz), 4.14 (q, 1H, J = 4.40 Hz), 3.95 (q, 1H, J = 3.50 Hz), 3.66-3.55 (d m, 2H), 3.04 (m, 2H), 1.16 (m, 3H). ^{13}C-NMR (125 MHz, DMSO-d_6) δ 154.67, 152.47, 148.57, 139.72, 119.82, 88.07, 86.02, 73.63, 70.78, 61.80, 34.24, 12.63. LC/ESI-MS (m/z): positive mode 296.1 [M + H]$^+$. Purity by HPLC-UV (254 nm)-ESI-MS: 97.4%.

(2*R*,3*S*,4*R*,5*R*)-2-(Hydroxymethyl)-5-(6-(isobutylamino)-9*H*-purin-9-yl)tetrahydrofuran-3,4-diol (7c)

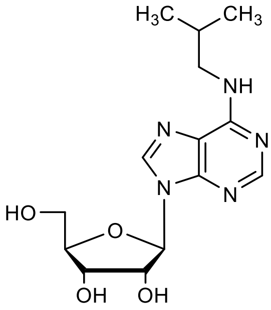

The compound was synthesized using isobutylamine (0.26 mL, 2.61 mmol) and afforded a white solid (653 mg, 96%), mp: 151.5-153.5 °C. ^{1}H NMR (DMSO-d_6) δ 8.33 (s, 1H), 8.19 (s, 1H), 7.86 (s, 1H), 5.88 (d, J = 6.2 Hz, 1H), 5.40 (t, J = 4.6 Hz, 2H), 5.14 (d, J = 4.7 Hz, 1H), 4.62 (q, J = 5.9 Hz, 1H), 4.21 – 4.09 (m, 1H), 3.97 (q, J = 3.4 Hz, 1H), 3.74 – 3.62 (m, 1H), 3.62 – 3.51 (m, 1H), 3.30 (s, 2H), 1.97 (s, 1H), 0.89 (d, J = 6.8 Hz, 6H). ^{13}C NMR (DMSO-d_6) δ 154.84, 152.25, 148.20, 139.55, 119.64, 87.94, 85.88, 73.43, 70.64, 61.66, 27.78, 20.05. LC-MS (*m/z*): positive mode 324.0 [M + H]$^+$. Purity by HPLC-UV (254 nm)-ESI-MS: 99.6%.

(2*R*,3*S*,4*R*,5*R*)-2-(Hydroxymethyl)-5-(6-(isopentylamino)-9*H*-purin-9-yl)tetrahydro-furan-3,4-diol (7d)

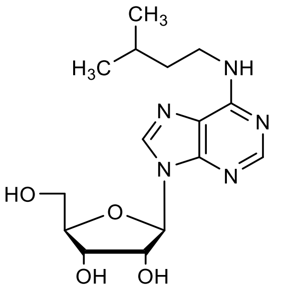

The compound was synthesized using isopentylamine (1.00 mL, 8.6 mmol, 4.6 equiv.) and afforded a white powder (600 mg, 95%), mp: 158 °C. ^{1}H-NMR (500 MHz, DMSO-d_6) δ 8.31 (s, 1H), 8.19 (s, 1H), 7.80 (s, 1H), 5.86 (d, 1H, J = 6.2 Hz), 5.39 (m, 2H), 5.14 (d, 1H, J = 4.6 Hz), 4.60 (q, 1H, J = 6.0 Hz), 4.14 (t, 1H, J = 4.7 Hz), 3.95 (q, 1H, J = 3.4 Hz), 3.66-3.54 (d m, 2H), 3.49 (br s, 2H), 1.62 (m, 1H), 1.48 (q, 2H, J = 7.0 Hz), 0.89 (d, 6H, J = 6.6 Hz). ^{13}C-NMR (126 MHz, DMSO-d_6) δ 154.81, 152.55, 148.35, 139.74, 119.91, 88.12, 86.06, 73.61, 70.83, 61.85, 38.16, 25.45, 22.66 (missing: NHCH_2). LC/ESI-MS (m/z): positive mode 338.1 [M + H]$^+$. Purity by HPLC-UV (254 nm)-ESI-MS: 97%.

(2*R*,3*S*,4*R*,5*R*)-2-(Hydroxymethyl)-5-(6-((2,4,4-trimethylpentan-2-yl)amino)-9*H*-purin-9-yl)tetrahydrofuran-3,4-diol (7e), CAS: 37676-69-0

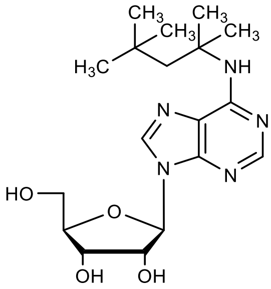

The compound was synthesized using 2,4,4-trimethylpentan-2-amine (0.40 mL, 2.60 mmol, 1.5 equiv) and afforded a white powder (220 mg, 34%), mp: 110 °C. ^{1}H-NMR (500 MHz, DMSO-d_6) δ 8.31 (s, 1H) 8.21 (s, 1H), 6.69 (s, 1H), 5.86 (d, 1H, J = 6.2 Hz), 5.41 (br s, 1H), 5.37 (dd, 1H, J = 4.6, 7.2 Hz), 5.16 (d, 1H, J = 3.3 Hz), 4.62 (br s, 1H), 4.13 (br s, 1H), 3.95 (q, 1H, J = 3.5 Hz), 3.66-3.54 (d m, 2H), 2.00 (s, 2H), 1.54 (s, 6H), 0.92 (s, 9H). ^{13}C-NMR (125 MHz, DMSO-d_6) δ 154.78, 151.89, 148.22, 139.77, 120.43, 88.13, 86.12, 73.57, 70.86, 61.88, 55.56, 50.23, 31.65, 31.40, 29.97. LC/ESI-MS (m/z): positive mode 379.9 [M + H]$^{+}$. Purity by HPLC-UV (254 nm)-ESI-MS: 92.0%.

(2*R*,3*R*,4*S*,5*R*)-2-(6-(Hexylamino)-9*H*-purin-9-yl)-5-(hydroxymethyl)tetrahydrofuran-3,4-diol (7f), CAS: 15824-83-6

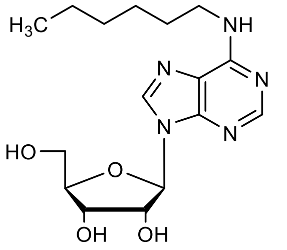

The compound was synthesized using *N*-hexylamine (0.25 mL, 1.90 mmol, 1.0 equiv) and afforded a white powder (660 mg, 99%), mp: 116 °C. ^{1}H-NMR (500 MHz, DMSO-d_6) δ 8.31 (s, 1H), 8.19 (s, 1H), 7.80 (s, 1H), 5.86 (d, 1H, J = 6.2 Hz), 5.39 (m, 2H, overlapping 2 × CHO*H*), 5.14 (d, 1H, J = 4.6 Hz), 4.60 (q, 1H, J = 6.0 Hz), 4.14 (t, 1H, J = 4.7 Hz), 3.95 (q, 1H, J = 3.4 Hz), 3.66-3.54 (d m, 2H), 3.46 (br s, 2H), 1.57 (m, 2H), 1.28 (m, 4H), 1.18 (t, 2H, J = 7.0 Hz), 0.85 (t, 3H, J = 6.7 Hz). ^{13}C-NMR (126 MHz, DMSO-d_6) δ 154.83, 152.50, 148.36, 139.69, 119.89, 88.10, 86.04, 73.61, 70.81, 61.83, 48.73, 45.77, 31.18, 26.20, 22.21, 14.04. LC/ESI-MS (m/z): positive mode 351.9 [M + H]$^{+}$. Purity by HPLC-UV (254 nm)-ESI-MS: 98.0%.

(2*R*,3*R*,4*S*,5*R*)-2-(6-(Dimethylamino)-9*H*-purin-9-yl)-5-(hydroxymethyl)tetrahydro-furan-3,4-diol (7g), CAS 2620-62-4

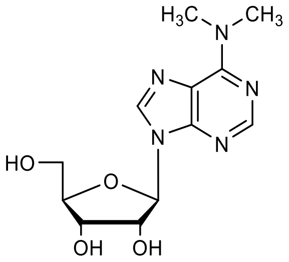

The compound was synthesized using *N*-dimethylamine (0.10 mL, 1.75 mmol, 1.0 equiv) and afforded a white powder (520 mg, >100%), mp: 186 °C (*lit.*[12] 184 °C). ^{1}H-NMR (500 MHz, DMSO-*d*$_6$) *δ* 8.35 (s, 1H), 8.20 (s, 1H), 5.90 (d, 1H, *J* = 5.97 Hz), 5.39 (d, 1H, *J* = 6.17 Hz), 5.32 (dd, 1H, *J* = 4.62, 6.95 Hz), 5.13 (d, 1H, *J* = 4.78 Hz), 4.56 (q, 1H, *J* = 5.99 Hz), 4.14 (m, 1H), 3.95 (q, 1H, *J* = 3.55 Hz), 3.66-3.55 (d m, 2H), 3.45 (br s, 6H). ^{13}C-NMR (125 MHz, DMSO-*d*$_6$) *δ* 154.46, 151.82, 150.05, 138.69, 119.94, 87.94, 85.88, 73.64, 70.65, 61.68, 11.57. LC/ESI-MS (m/z): positive mode 296.0 [M + H]$^+$. Purity by HPLC-UV (254 nm)-ESIMS: 98%.

(2*R*,3*R*,4*S*,5*R*)-2-(6-(Ethyl(methyl)amino)-9*H*-purin-9-yl)-5-(hydroxymethyl)tetra-hydrofuran-3,4-diol (7h), CAS: 402724-55-4

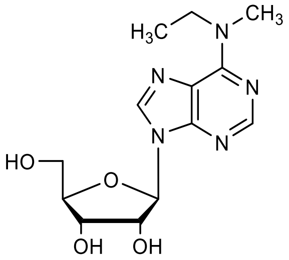

The compound was synthesized using *N*-ethylmethylamine (0.20 mL, 1.75 mmol, 1.0 equiv) and afforded a white powder (930 mg, >100%), mp: 101 °C. ^{1}H-NMR (500 MHz, DMSO-*d*$_6$) *δ* 8.35 (s, 1H), 8.20 (s, 1H), 5.90 (d, 1H, *J* = 6.00 Hz), 5.39 (d, 1H, *J* = 6.19 Hz), 5.32 (dd, 1H, *J* = 4.61, 6.96 Hz), 5.13 (d, 1H, *J* = 4.76 Hz), 4.57 (q, 1H, *J* = 5.99 Hz), 4.14 (m, 1H), 4.04 (br s, 2H), 3.95 (q, 1H, *J* = 3.51 Hz), 3.66-3.54 (d m, 2H), 3.39 (br s, 3H), 1.17 (t, 3H, *J* = 7.00 Hz). ^{13}C-NMR (125 MHz, DMSO-*d*$_6$) *δ* 153.82, 151.89, 150.02, 138.82, 119.69, 87.91, 85.88, 73.59, 70.66, 61.69, 44.78, 35.47, 12.56. LC/ESI-MS (m/z): positive mode 310.0 [M + H]$^+$. Purity by HPLC-UV (254 nm)-ESI-MS: 98.0%.

(2*R*,3*S*,4*R*,5*R*)-2-(Hydroxymethyl)-5-(6-(methyl(propyl)amino)-9*H*-purin-9-yl)tetra-hydrofuran-3,4-diol (7i), CAS: 402724-38-3

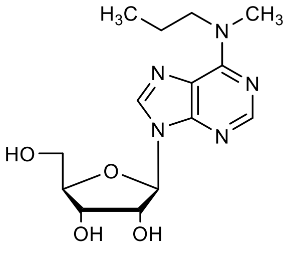

The compound was synthesized using *N*-methylpropylamine (0.18 mL, 1.75 mmol, 1.0 equiv) and afforded a white powder (660 mg, >100%), mp: 178 °C. ^{1}H-NMR (500 MHz, DMSO-d_6) δ 8.35 (s, 1H), 8.19 (s, 1H), 5.89 (d, 1H, J = 5.97 Hz), 5.41 (d, 1H, J = 6.16 Hz), 5.33 (m, 1H), 5.14 (d, 1H, J = 4.64 Hz), 4.57 (q, 1H, J = 5.76 Hz), 4.14 (d, 1H, J = 3.62 Hz), 3.95 (d, 1H, J = 3.13 Hz), 3.66-3.54 (d m, 2H), 3.16 (br s, 2H) [bulb underneath previous peaks: NCH_3)], 1.64 (q, 2H, J = 7.30 Hz), 0.87 (t, 3H, J = 7.34 Hz). ^{13}C-NMR (125 MHz, DMSO-d_6) δ 154.16, 151.88, 150.10, 138.79, 119.71, 87.92, 85.92, 73.62, 70.71, 61.74, 51.32, 48.75, 21.58, 11.06. LC/ESI-MS (m/z): positive mode 324.1 [M + H]$^+$. Purity by HPLC-UV (254 nm)-ESI-MS: 97.7%.

(2*R*,3*R*,4*S*,5*R*)-2-(6-(Diethylamino)-9*H*-purin-9-yl)-5-(hydroxymethyl)tetrahydrofuran-3,4-diol (7j), CAS: 2139-60-8

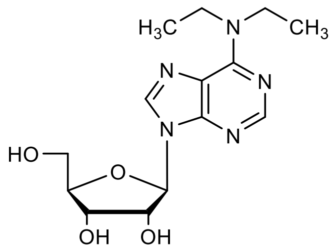

The compound was synthesized using *N*-diethylamine (0.30 mL, 3.40 mmol, 2.0 equiv) and afforded a white powder (500 mg, 100%), mp: 180 °C. ^{1}H-NMR (500 MHz, DMSO-d_6) δ 8.34 (s, 1H), 8.19 (s, 1H), 5.89 (d, 1H, J = 6.04 Hz), 5.39 (d, 1H, J = 6.19 Hz), 5.33 (dd, 1H, J = 4.59, 7.02 Hz), 5.13 (d, 1H, J = 4.61 Hz), 4.58 (q 1H, J = 6.04 Hz), 4.14(td, 1H, J = 3.36, 4.82 Hz), 4.03 (br s, 4H), 3.95 (q, 1H, J = 3.54 Hz), 3.66-3.54 (d m, 2H), 1.19 (t, 6H, J = 6.95 Hz). ^{13}C-NMR (125 MHz, DMSO-d_6) δ 153.27, 151.95, 150.06, 138.96, 119.47,87.94, 85.91, 73.57, 70.70, 61.73, 42.56, 13.48. LC/ESI-MS (m/z): positive mode 324.1 [M + H]$^+$. Purity by HPLC-UV (254 nm)-ESI-MS: 99.2%.

(2*R*,3*R*,4*S*,5*R*)-2-(6-(Ethyl(propyl)amino)-9*H*-purin-9-yl)-5-(hydroxymethyl)tetra-hydrofuran-3,4-diol (7k)

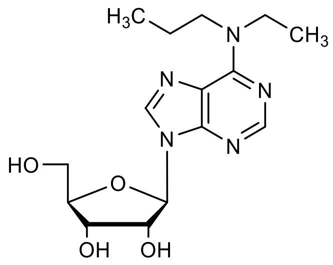

The compound was synthesized using *N*-ethylpropylamine (0.20 mL, 1.75 mmol, 1.0 equiv) and afforded a white powder (380 mg, 65%), mp: 160 °C. ^{1}H-NMR (500 MHz, CD_3OD), δ 8.15 (d, 2H, J = 2.01 Hz), 5.93 (d, 1H, J = 6.55 Hz), 4.74 (dd, 1H, J = 5.15, 6.48 Hz), 4.30 (dd, 1H, J = 2.45, 5.09 Hz), 4.16 (q, 1H, J = 2.40 Hz), 3.88-3.72 (d m, 2H), overlapping with 4.10-3.72 (br s, 4H, 2 × NCH_2), 1.73 (m, 2H), 1.25 (t, 3H, J = 7.04 Hz), 0.95 (t, 3H, J = 7.39 Hz). ^{13}C-

NMR (151 MHz, CD_3OD) δ 155.40, 152.72, 150.70, 140.17, 121.60, 91.21, 88.17, 75.17, 72.77, 63.58, 51.25, 44.72, 22.52, 13.90, 11.36. LC/ESI-MS (m/z): positive mode 310.0 $[M + H]^+$. Purity by HPLC-UV (254 nm)-ESI-MS: 97.2%.

(2*R*,3*R*,4*S*,5*R*)-2-(6-(Dipropylamino)-9*H*-purin-9-yl)-5-(hydroxymethyl)tetrahydro-furan-3,4-diol (7l), CAS: 17270-24-5

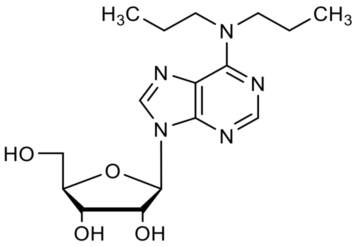

The compound was synthesized using *N*-dipropylamine (0.25 mL, 1.75 mmol, 1.0 equiv) and afforded a white powder (650 mg, >100%), mp: 145 °C. ^{1}H-NMR (500 MHz, DMSO-d_6) δ 8.35 (s, 1H), 8.18 (br s, 1H), 5.89 (d, 1H, J = 6.05 Hz), 5.40 (d, 1H, J = 5.91 Hz), 5.33 (dd, 1H, J = 4.63, 6.97 Hz), 5.14 (d, 1H, J = 4.60 Hz), 4.58 (q, 1H, J = 5.66 Hz), 4.13 (q, 1H, J = 4.53 Hz), 4.06 (m, 4H), 3.95 (q, 1H, J = 3.50 Hz), 3.65-3.54 (d m, 2H), 1.64 (m, 4H), 0.89 (t, 6H, J = 7.37 Hz). ^{13}C-NMR (125 MHz, DMSO-d_6) δ 153.80, 151.88, 150.10, 138.89, 119.50, 87.92, 85.92, 73.56, 70.73, 61.92, 56.17, 48.74, 18.70, 11.18. LC/ESI-MS (m/z): positive mode 352.1 $[M + H]^+$. Purity by HPLC-UV (254 nm)-ESI-MS: 98.3%.

(2*R*,3*R*,4*S*,5*R*)-2-(6-(Dibutylamino)-9*H*-purin-9-yl)-5-(hydroxymethyl)tetrahydrofuran-3,4-diol (7m), CAS: 81609-38-3

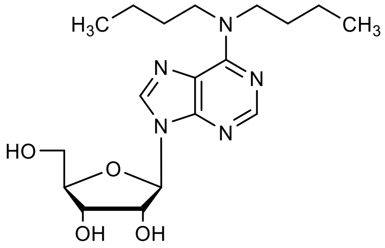

The compound was synthesized using dibutylamine (0.44 mL, 2.61 mmol) and afforded a light grey solid (590 mg, 89%), mp: 141.0-143.0 °C. ^{1}H NMR (DMSO-d_6) δ 8.35 (s, 1H), 8.19 (s, 1H), 5.90 (d, J = 6.0 Hz, 1H), 5.40 (d, J = 6.3 Hz, 1H), 5.33 (dd, J = 7.0, 4.5 Hz, 1H), 5.14 (d, J = 4.7 Hz, 1H), 4.63 – 4.53 (m, 1H), 4.27 – 4.03 (m, 2H), 3.96 (q, J = 3.5 Hz, 2H), 3.76 – 3.61 (m, 2H), 3.59 – 3.49 (m, 1H), 2.92 – 2.80 (m, 1H), 1.66 – 1.52 (m, 4H), 1.39 – 1.28 (m, 4H), 0.95 – 0.86 (m, 6H). ^{13}C NMR (DMSO-d_6) δ 153.57, 151.71, 149.92, 138.62, 119.33, 87.72, 85.74, 73.41, 70.54, 61.56, 46.44, 27.47, 19.50, 19.24, 13.82, 13.42. LC-MS (*m/z*): positive mode 380.1 $[M + H]^+$. Purity by HPLC-UV (254 nm)-ESI-MS: 99.2%.

(2*R*,3*S*,4*R*,5*R*)-2-(Hydroxymethyl)-5-(6-(phenylamino)-9*H*-purin-9-yl)tetrahydrofuran-3,4-diol (7n), CAS: 23589-16-4

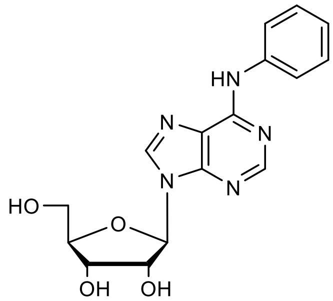

The compound was synthesized using **6** (300 mg, 1.05 mmol), aniline (0.14 mL, 1.58 mmol) and afforded a white solid (259 mg, 72%), mp: 192.3-194.3 °C. ^{1}H NMR (DMSO-d_6) δ 9.92 (s, 1H), 8.54 (s, 1H), 8.40 (s, 1H), 8.02 – 7.88 (m, 2H), 7.42 – 7.26 (m, 2H), 7.10 – 6.99 (m, 1H), 5.96 (d, J = 5.9 Hz, 1H), 5.47 (d, J = 6.1 Hz, 1H), 5.33 – 5.13 (m, 2H), 4.71 – 4.59 (m, 1H), 4.24 – 4.12 (m, 1H), 3.99 (q, J = 3.7 Hz, 1H), 3.78 – 3.50 (m, 2H). ^{13}C NMR (DMSO-d_6) δ 152.17, 151.91, 149.33, 140.68, 139.51, 128.38, 122.72, 120.90, 120.35, 87.86, 85.84, 73.60, 70.53, 61.55. LC-MS (*m/z*): positive mode 344.1 [M + H]$^+$. Purity by HPLC-UV (254 nm)-ESI-MS: 98.8%.

(2*R*,3*R*,4*S*,5*R*)-2-(6-(Benzylamino)-9*H*-purin-9-yl)-5-(hydroxymethyl)tetrahydrofuran-3,4-diol (7o), CAS: 4294-16-0

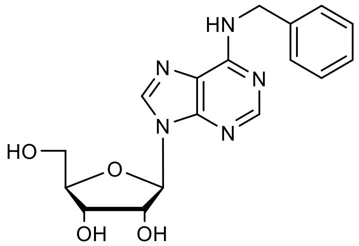

The compound was synthesized using benzylamine (0.27 mL, 2.61 mmol) and afforded a yellowish solid (609 mg, 98%), mp: 163.0-165.0 °C. ^{1}H NMR (DMSO-d_6) δ 8.37 (s, 1H), 8.20 (s, 1H), 7.37 – 7.26 (m, 5H), 5.90 (d, J = 6.1 Hz, 1H), 5.27 (d, J = 122.2 Hz, 3H), 4.81 – 4.55 (m, 3H), 4.15 (dd, J = 5.0, 3.1 Hz, 1H), 3.97 (q, J = 3.5 Hz, 1H), 3.74 (s, 1H), 3.62 (ddd, J = 71.4, 12.1, 3.7 Hz, 2H). ^{13}C NMR (DMSO-d_6) δ 154.50, 152.33, 148.47, 142.92, 139.92, 128.19, 128.11, 127.20, 127.10, 126.59, 119.77, 87.94, 85.88, 73.48, 70.63, 61.65, 45.22. LC-MS (*m/z*): positive mode 358.1 [M + H]$^+$. Purity by HPLC-UV (254 nm)-ESI-MS: 99.6%.

(2*R*,3*R*,4*S*,5*R*)-2-(6-(Benzoylamino)-9*H*-purin-9-yl)-5-(hydroxymethyl)tetrahydrofuran-3,4-diol (7p), CAS: 4546-55-8

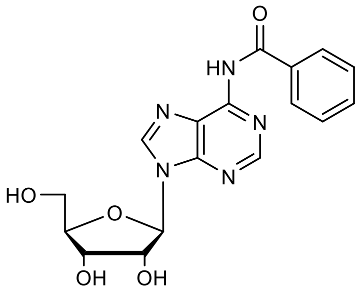

To a solution of adenosine (**4**, 500 mg, 1.87 mmol, 1 eq.) in anhydrous pyridine (10 mL), chlorotrimethylsilane (1.78 mL, 14.03 mmol, 7.5 eq.) was added, and the mixture was stirred at rt for 15 min. Benzoyl chloride (1.39 mL, 9.35 mmol, 5 eq.) was subsequently added, and the mixture was stirred at rt for 2 h. The mixture was cooled to 0 °C, and H_2O (2 mL) was added to quench the reaction. Aqueous ammonia (5 mL, 28% NH_3 in H_2O) was subsequently added for the deprotection of 2′-, 3′- and 5′-trimethylsilyl groups, and the mixture was stirred at rt for 30 min. The reaction progress was monitored by TLC (MeOH/DCM 1:9). After the reaction was completed, the mixture was concentrated, and the residue was lyophilized. The crude compound was finally purified by silica gel column chromatography using 10% MeOH in DCM yielding the product as a white solid (688 mg, 99%), mp: 133.0-135.0 °C (*lit.*[13] 133-134 °C). ^{1}H NMR (600 MHz, DMSO-d_6) δ 11.20 (s, 1H), 8.73 (d, *J* = 23.5 Hz, 2H), 8.10 – 7.99 (m, 2H), 7.99 – 7.89 (m, 1H), 7.69 – 7.45 (m, 4H), 6.05 (d, *J* = 5.8 Hz, 1H), 5.13 (s, 1H), 4.65 (t, *J* = 5.4 Hz, 1H), 4.20 (dd, *J* = 4.8, 3.6 Hz, 1H), 3.99 (q, *J* = 3.9 Hz, 1H), 3.70 (dd, *J* = 11.9, 4.1 Hz, 1H), 3.59 (dd, *J* = 11.9, 4.0 Hz, 1H). ^{13}C NMR (151 MHz, DMSO-d_6) δ 165.72, 152.20, 151.57, 150.41, 143.15, 133.39, 132.48, 132.45, 129.23, 128.48, 128.42, 125.88, 87.62, 85.74, 73.69, 70.38, 61.34. LC/ESI-MS (*m/z*): positive mode 372.1 $[M + H]^+$. Purity by HPLC-UV (254 nm)-ESI-MS: 98.3%.

(2*R*,3*R*,4*S*,5*R*)-2-(6-((3-(1*H*-Imidazol-1-yl)propyl)amino)-9*H*-purin-9-yl)-5-(hydroxyl-methyl)tetrahydrofuran-3,4-diol (7q)

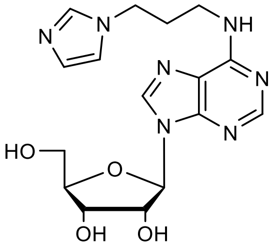

The compound was synthesized using 3-(1H-imidazol-1-yl)propan-1-amine (0.30 mL, 2.60 mmol). The crude product was extracted with H_2O from ethyl acetate. Lyophilization of the H_2O layer yielding the product as brown solid (600 mg, 95%), mp: 100 °C. ^{1}H-NMR (500 MHz, DMSO-d_6) δ 8.35 (s, 1H), 8.20 (s, 1H), 7.98 (s, 1H), 7.71 (d, 1H, *J* = 27.30 Hz), 7.21 (d, 1H, *J* = 21.59 Hz), 6.91 (s, 1H), 5.88 (d, 1H, *J* = 6.14 Hz), 5.40 (br s, 1H), 5.18 (br s, 1H), 4.59 (t, 1H, *J* = 5.51 Hz), 4.14 (m, 1H), 4.07 (t, 2H, *J* = 6.86 Hz), 4.04 (t, 2H, *J* = 6.94 Hz), 3.95 (q, 1H, *J* =

3.40 Hz), 3.66-3.54 (dm, 2H), 2.69 (m, 2H). ^{13}C-NMR (125 MHz, DMSO-d_6) δ 154.87, 152.50, 148.52, 139.93, 137.43, 128.03, 119.70, 119.51, 88.09, 86.07, 73.70, 70.82, 61.63, 43.23, 36.32, 28.84. LC/ESI-MS (m/z): positive mode 376.0 [M + H]$^+$. Purity by HPLC-UV (254 nm)-ESI-MS: 97.2%.

(2*R*,3*S*,4*R*,5*R*)-2-(Hydroxymethyl)-5-(6-((4-phenylbutyl)amino)-9*H*-purin-9-yl)tetra-hydrofuran-3,4-diol (7r), CAS: 101565-58-6

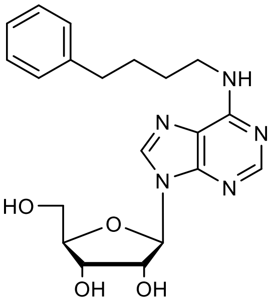

The compound was synthesized using **6** (2.00 g, 6.98 mmol), 4-phenylbutylamine (1.65 mL, 10.47 mmol) and afforded a light grey solid (2.73 g, 98%), mp: 115.5-117.5 °C. ^{1}H NMR (600 MHz, DMSO-d_6) δ 8.31 (s, 1H), 8.18 (s, 1H), 7.85 (s, 1H), 7.42 (d, J = 8.4 Hz, 2H), 7.15 (d, J = 8.4 Hz, 2H), 5.86 (d, J = 6.2 Hz, 1H), 5.38 (m, 2H), 5.14 (d, J = 4.6 Hz, 1H), 4.60 (q, J = 5.8 Hz, 1H), 4.14 (q, J = 4.6 Hz, 1H), 3.95 (q, J = 3.5 Hz, 1H), 3.60-3.51 (dm, 2H), 3.50 (s, 2H), 2.57 (t, J = 6.8 Hz, 2H), 1.59 (s, 4H). ^{13}C NMR (126 MHz, CD_3OD) δ 154.85, 152.49, 148.39, 141.80, 139.75, 132.59, 131.18, 130.77, 118.76, 88.11, 86.05, 73.63, 70.81, 61.84, 34.27, 28.84, 28.30. LC-MS (*m/z*): positive mode 399.9 [M + H]$^+$. Purity by HPLC-UV (254 nm)-ESI-MS: 98.8%.

(2*R*,3*S*,4*R*,5*R*)-2-(Hydroxymethyl)-5-(6-(methyl(4-phenylbutyl)amino)-9*H*-purin-9-yl)tetrahydrofuran-3,4-diol (7s)

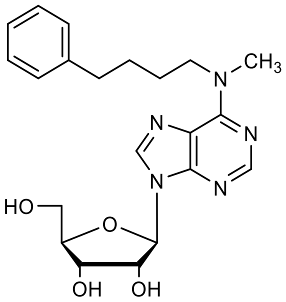

The compound was synthesized using methyl(4-phenylbutyl)amine (0.38 mL, 2.09 mmol) and afforded a white solid (614 mg, 85%), mp: 168.0-169.0 °C. ^{1}H NMR (DMSO-d_6) δ 8.37 (s, 1H), 8.20 (s, 1H), 7.25 (t, J = 7.5 Hz, 2H), 7.20 – 7.11 (m, 3H), 5.91 (d, J = 6.0 Hz, 1H), 5.42 (d, J = 6.2 Hz, 1H), 5.35 (dd, J = 6.9, 4.6 Hz, 1H), 5.16 (d, J = 4.7 Hz, 1H), 4.58 (q, J = 5.8 Hz, 1H), 4.52 – 4.03 (m, 3H), 3.96 (q, J = 3.6 Hz, 2H), 3.82 – 3.45 (m, 4H), 2.61 (t, J = 7.5 Hz, 2H), 1.73 – 1.54 (m, 4H). ^{13}C NMR (DMSO-d_6) δ 153.97, 151.75, 149.92, 142.09, 138.64, 128.27,

128.24, 125.66, 119.56, 87.79, 85.77, 73.48, 70.55, 61.58, 34.89, 28.12. LC-MS (*m/z*): positive mode 414.0 [M + H]$^+$. Purity by HPLC-UV (254 nm)-ESI-MS: 99.4%.

(2*R*,3*R*,4*S*,5*R*)-2-(6-(Ethyl(4-phenylbutyl)amino)-9*H*-purin-9-yl)-5-(hydroxymethyl)-tetrahydrofuran-3,4-diol (7t)

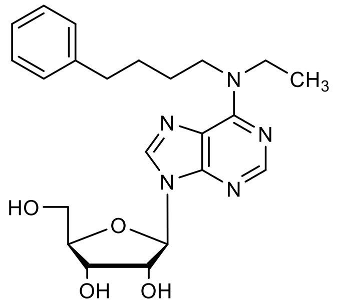

The compound was synthesized using *N*,*N*-ethyl(4-phenylbutyl)amine (**10b**, 680 mg, 3.82 mmol, 1 equiv) and afforded a white powder (930 mg, 58%), mp: 60.5 °C. ^{1}H-NMR (500 MHz, DMSO-*d*$_6$) *δ* 8.35 (s, 1H), 8.18 (s, 1H), 7.24-7.15 (m, 5H), 5.89 (d, 1H, *J* = 6.03 Hz), 5.39 (d, 1H, *J* = 6.19 Hz), 5.32 (dd, 1H, *J* = 4.61, 6.99 Hz), 5.13 (d, 1H, *J* = 4.74 Hz), 4.58 (q, 1H, *J* = 6.04 Hz), 4.14 (m, 1H), 4.06 (br s, 2H), 3.95 (q, 1H, *J* = 3.52 Hz), 3.75 (br s, 2H), 3.66-3.55 (d m, 2H), 2.62 (t, 2H, *J* = 7.29 Hz), 1.62 (m, 4H), 1.16 (t, 3H, *J* = 6.81 Hz). ^{13}C-NMR (125 MHz, DMSO-*d*$_6$) *δ* 153.52, 151.92, 150.09, 142.24, 138.92, 128.41, 125.77, 119.49, 87.94, 85.92, 75.59, 70.71, 61.74, 48.74, 47,45, 35.07, 28.37, 13.90. LC/ESI-MS (m/z): positive mode 428.1 [M + H]$^+$. Purity by HPLC-UV (254 nm)-ESI-MS: 97.4%.

(2*R*,3*R*,4*S*,5*R*)-2-(6-(Butyl(4-phenylbutyl)amino)-9*H*-purin-9-yl)-5-(hydroxymethyl)tetrahydrofuran-3,4-diol (7u)

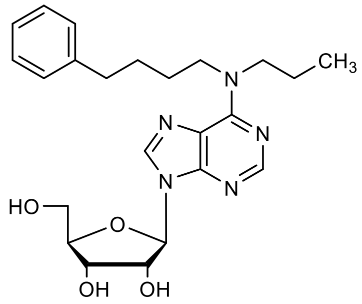

The compound was synthesized using **10c** (499 mg, 2.61 mmol) and afforded a light brown solid (719 mg, 94%), mp: 73.0-74.5 °C. ^{1}H NMR (DMSO-*d*$_6$) *δ* 8.36 (s, 1H), 8.19 (s, 1H), 7.35 – 7.09 (m, 5H), 5.90 (d, *J* = 6.0 Hz, 1H), 5.47 – 5.30 (m, 2H), 5.16 (d, *J* = 4.6 Hz, 1H), 4.59 (q, *J* = 5.7 Hz, 1H), 4.15 (q, *J* = 4.3 Hz, 2H), 3.96 (q, *J* = 3.5 Hz, 2H), 3.67 (dt, *J* = 12.1, 3.9 Hz, 2H), 3.55 (ddd, *J* = 11.6, 6.6, 3.6 Hz, 1H), 2.93 – 2.75 (m, 1H), 2.61 (dt, *J* = 11.1, 7.4 Hz, 2H), 1.61 (ddp, *J* = 23.1, 15.2, 7.6 Hz, 6H), 0.88 (dt, *J* = 11.3, 7.4 Hz, 3H). ^{13}C NMR (DMSO-*d*$_6$) *δ* 153.63, 151.75, 149.96, 142.11, 138.75, 128.33, 128.29, 128.22, 125.65, 119.36, 87.78, 85.78,

73.45, 70.59, 61.60, 46.75, 34.93, 34.51, 28.21, 27.79, 19.16, 10.95. LC-MS (*m/z*): positive mode 442.4 [M + H]$^+$. Purity by HPLC-UV (254 nm)-ESI-MS: 99.0%.

(2*R*,3*R*,4*S*,5*R*)-2-(6-(Butyl(4-phenylbutyl)amino)-9*H*-purin-9-yl)-5-(hydroxymethyl)tetrahydrofuran-3,4-diol (7v)

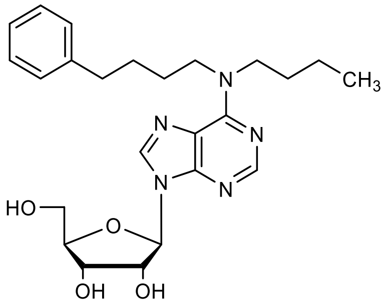

The compound was synthesized using **6** (300 mg, 1.05 mmol), **10d** (325 mg, 1.58 mmol) and afforded a grey semi-solid (491 mg, >100%). ^{1}H NMR (DMSO-*d*$_6$) δ 8.36 (s, 1H), 8.19 (s, 1H), 7.35 – 7.07 (m, 5H), 5.90 (d, *J* = 6.0 Hz, 1H), 5.47 – 5.28 (m, 2H), 5.15 (d, *J* = 4.7 Hz, 1H), 4.59 (q, *J* = 5.8 Hz, 1H), 4.35 – 4.03 (m, 3H), 3.96 (q, *J* = 3.5 Hz, 1H), 3.86 – 3.48 (m, 4H), 2.62 (t, *J* = 7.3 Hz, 2H), 1.74 – 1.50 (m, 6H), 1.31 (q, *J* = 7.5 Hz, 2H), 0.90 (t, *J* = 7.4 Hz, 3H). ^{13}C NMR (DMSO-*d*$_6$) δ 153.57, 151.73, 149.93, 142.07, 138.69, 128.26, 128.18, 125.62, 119.33, 87.74, 85.75, 73.41, 70.56, 61.58, 34.88, 30.65, 28.16, 19.50, 13.83. LC-MS (*m/z*): positive mode 456.2 [M + H]$^+$. Purity by HPLC-UV (254 nm)-ESI-MS: 98.8%.

(2*R*,3*R*,4*S*,5*R*)-2-(6-(Bis(4-phenylbutyl)amino)-9*H*-purin-9-yl)-5-(hydroxymethyl)tetrahydrofuran-3,4-diol (7w)

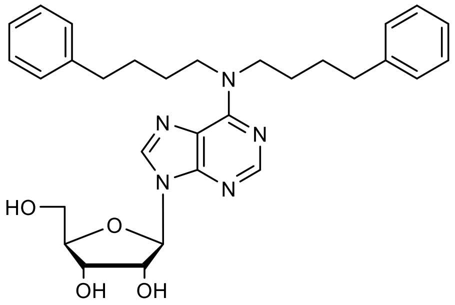

The compound was synthesized using **6** (300 mg, 1.05 mmol), **10e** (445 mg, 1.58 mmol) and afforded a yellowish solid (517 mg, 93%), mp: 110.0-111.5 °C. ^{1}H NMR (DMSO-*d*$_6$) δ 8.36 (s, 1H), 8.19 (s, 1H), 7.31 – 7.11 (m, 10H), 5.90 (d, *J* = 6.0 Hz, 1H), 5.40 (d, *J* = 6.1 Hz, 1H), 5.32 (dd, *J* = 7.0, 4.6 Hz, 1H), 5.14 (d, *J* = 4.7 Hz, 1H), 4.59 (q, *J* = 5.7 Hz, 1H), 4.43 – 3.91 (m, 4H), 3.90 – 3.49 (m, 4H), 2.61 (t, *J* = 7.2 Hz, 4H), 1.73 – 1.56 (m, 8H). ^{13}C NMR (DMSO-*d*$_6$) δ 153.55, 151.69, 149.93, 142.04, 138.68, 128.25, 128.22, 128.16, 125.59, 119.31, 87.74, 85.72, 73.40, 70.52, 61.55, 47.31, 34.87, 34.59, 30.62, 28.12, 27.93, 26.11. LC-MS (*m/z*): 532.5 [M + H]$^+$. Purity by HPLC-UV (254 nm)-ESI-MS: 100%.

N-Ethyl-4-phenylbutan-1-amine (10b), CAS 34059-12-6

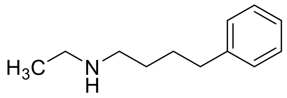

4-Phenylbutylbromide (0.40 mL, 2.34 mmol) was dissolved in 2 M ethylamine in MeOH (10 mL), the mixture was refluxed at 60 °C for 18 h and monitored by TLC (MeOH/DCM, 1:9). After the reaction was completed, cooled to rt and the solvent was evaporated *in vacuum*. The crude compound was purified by silica gel column chromatography (8% MeOH in DCM) and afforded a white solid (380 mg, 92%), mp: 156 °C. ^{1}H NMR (500 MHz, DMSO-*d*$_6$) *δ* 7.28 (m, 5H), 2.89 (m, 4H), 2.59 (t, 2H, *J* = 7.1 Hz), 1.59 (m, 4H), 1.16 (t, 3H, *J* = 7.3 Hz). ^{13}C NMR (126 MHz, DMSO-*d*$_6$) *δ* 141.68, 128.43, 128.41, 125.94, 46.25, 42.07, 34.60, 27.87, 25.67, 11.07. LC-MS (m/z): positive mode 178.2 [M + H]$^+$. Purity by HPLC-UV (254 nm)-ESI-MS: 79.0%.

4-Phenyl-N-propylbutan-1-amine (10c)

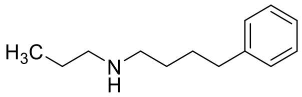

The compound was synthesized using 1-bromo-4-phenylbutane (1.00 mL, 5.70 mmol), propylamine (2.34 mL, 28.5 mmol) and afforded a yellowish solid (854 mg, 78%), mp: 183.0-184.8 °C. ^{1}H NMR (DMSO-*d*$_6$) *δ* 7.29 (t, *J* = 7.6 Hz, 2H), 7.23 – 7.15 (m, 3H), 2.89 (s, 2H), 2.85 – 2.79 (m, 2H), 2.60 (t, *J* = 6.9 Hz, 2H), 1.75 – 1.44 (m, 7H), 0.90 (t, *J* = 7.5 Hz, 3H). ^{13}C NMR (DMSO-*d*$_6$) *δ* 141.57, 128.32, 128.28, 125.81, 48.37, 46.62, 34.48, 27.76, 25.07, 18.97, 10.92. LC-MS (*m/z*): positive mode 191.8 [M + H]$^+$. Purity by HPLC-UV (254 nm)-ESI-MS: 97.7%.

N-Butyl-4-phenylbutan-1-amine (10d), CAS: 143996-03-6

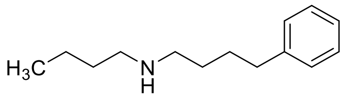

The compound was synthesized using 1-bromo-4-phenylbutane (1.00 mL, 5.70 mmol), butylamine (2.82 mL, 28.50 mmol) and afforded a white solid (888 mg, 76%), mp: 203.5-205.5 °C (*lit.*[14] *oil*). ^{1}H NMR (DMSO-*d*$_6$) *δ* 7.40 – 7.08 (m, 5H), 3.02 – 2.77 (m, 4H), 2.60 (t, *J* = 7.0 Hz, 2H), 1.87 – 1.40 (m, 7H), 1.32 (h, *J* = 7.4 Hz, 2H), 0.88 (t, *J* = 7.4 Hz, 3H). ^{13}C NMR (DMSO-*d*$_6$) *δ* 141.55, 128.25, 125.78, 46.61, 46.51, 34.45, 27.74, 27.47, 25.07, 19.23, 13.44. LC-MS (*m/z*): positive mode 205.9 [M + H]$^+$. Purity by HPLC-UV (254 nm)-ESI-MS: 98.9%.

Bis(4-phenylbutyl)amine (10e), CAS: 94875-96-4

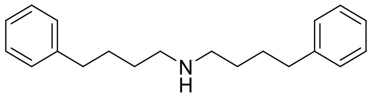

The compound was synthesized using1-bromo-4-phenylbutane (0.50 mL, 2.90 mmol), 4-phenylbutylamine (2.29 mL, 14.50 mmol) and afforded a yellowish oil (690 mg, 85%). ^{1}H NMR (DMSO-d_6) δ 7.36 – 7.03 (m, 10H), 2.60 – 2.53 (m, 4H), 2.51 (s, 5H), 1.64 – 1.49 (m, 4H), 1.41 (q, J = 7.4 Hz, 4H). ^{13}C NMR (DMSO-d_6) δ 142.20, 128.19, 128.12, 125.52, 48.96, 35.02, 28.85, 28.68. LC-MS (*m/z*): positive mode 281.9 [M + H]$^+$. Purity by HPLC-UV (254 nm)-ESI-MS: 100%.

3-(3-Methoxyphenyl)propan-1-amine (13b)

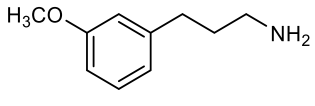

The compound was synthesized using 3-(3-methoxyphenyl)propionic acid (**11a**, 2.00 g, 11.08 mmol) and afforded a yellow liquid (0.69 g, 38%). ^{1}H-NMR (500 MHz, DMSO-d_6) δ 7.16 (m, 1H), 6.73 (m, 3H), 3.72 (s, 3H), 2.54 (dt, 4H, J = 7.25, 11.65 Hz), 1.62 (m, 2H). ^{13}C-NMR (125 MHz, DMSO- d_6) δ 159.40, 144.06, 129.30, 120.7, 114.08, 111.12, 55.00, 41.27, 35.07, 32.76. LC/ESI-MS (m/z): positive mode 166.0 [M + H]$^+$. Purity by HPLC-UV (254 nm)-ESI-MS: 100%.

3-(4-Methoxyphenyl)propan-1-amine (13c)

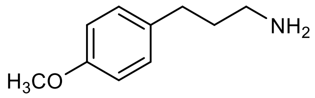

The compound was synthesized using 3-(4-methoxyphenyl)propionic acid (**11b**, 2.00 g, 11.08 mmol) and afforded a colorless liquid (0.84 g, 46%). ^{1}H-NMR (500 MHz, DMSO-d_6) δ 7.08 (d, 2H, J = 8.63 Hz), 6.81 (d, 2H, J = 8.63 Hz), 3.69 (s, 3H), 2.50 (m, 4H), 1.57 (dt, 2H, J = 6.94, 14.15 Hz). ^{13}C-NMR (125 MHz, DMSO-d_6) δ 157.40, 134.29, 129.26, 113.27, 55.08, 48.72, 41.26, 33.57, 31.79, 18.69. LC/ESI-MS (m/z): positive mode 165.9 [M + H]$^+$. Purity by HPLC-UV (254 nm)-ESI-MS: 98.1%.

(2*R*,3*S*,4*R*,5*R*)-2-(Hydroxymethyl)-5-(6-((3-phenylpropyl)amino)-9*H*-purin-9-yl)tetra-hydrofuran-3,4-diol (14a)

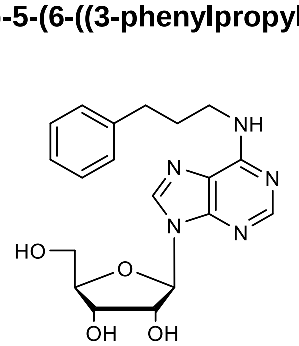

The compound was synthesized using 3-phenylpropylamine (0.25 mL, 1.80 mmol, 1.0 equiv) and afforded a white powder (1.10 g, >100%), mp: 103 °C. ^{1}H-NMR (500 MHz, DMSO-d_6) δ 8.33 (s, 1H), 8.19 (br s, 1H), 7.89 (d, 2H, J = 29.21 Hz), 7.28 (dt, 3H, J = 7.52, 21.51 Hz), 5.87 (d, 1H, J = 6.16 Hz), 5.41 (d, 1H, J = 6.24, Hz), 5.38 (m, 1H), 5.15 (d, 1H, J = 4.59 Hz), 4.60

(q, 1H, *J* = 5.92 Hz), 4.14 (q, 1H, *J* = 4.65 Hz), 3.95 (q, 1H, *J* = 3.34 Hz), 3.66-3.55 (d m, 2H), 3.50 (m, 2H), 2.63 (t, 2H, *J* = 7.71 Hz), 1.87 (m, 2H). ^{13}C-NMR (125 MHz, DMSO-d_6) δ 154.84, 152.50, 141.98, 140.97, 139.78, 128.58, 128.44, 128.40, 126.19, 125.83, 119.96, 88.08, 86.03, 73.63, 70.80, 61.82, 45.66, 31.97, 28.88. LC/ESI-MS (m/z): positive mode 386.2 $[M + H]^+$. Purity by HPLC-UV (254 nm)-ESI-MS: 91.0%.

(2*R*,3*S*,4*R*,5*R*)-2-(Hydroxymethyl)-5-(6-((3-(3-methoxyphenyl)propyl)amino)-9*H*-purin-9-yl)tetrahydrofuran-3,4-diol (14b)

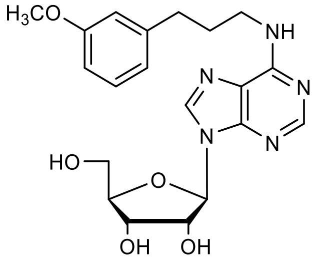

The compound was synthesized using 3-methoxybenzenepropanamine (**13b**, 290 mg, 1.75 mmol, 1.0 equiv) and afforded a white powder (340 mg, 47%), mp: 132 °C. ^{1}H-NMR (500 MHz, DMSO-d_6) δ 8.33 (s, 1H), 8.19 (br s, 1H), 7.94 (s, 1H), 7.17 (t, 1H, *J* = 8.05 Hz), 6.78-6.71 (m, 3H), 5.87 (d, 1H, *J* = 6.19 Hz), 5.41 (overlapping d and t, 2H, C*H*OH & C*H*OH), 5.16 (d, 1H, *J* = 4.62 Hz), 4.60 (q, 1H, *J* = 6.09 Hz), 4.13 (td, 1H, *J* = 3.11, 4.75 Hz), 3.95 (q, 1H), 3.71 (s, 3H), 3.68-3.52 (m, 2H), 3.16 (d, 1H, *J* = 5.24 Hz), 2.61 (m, 2H), 1.89 (m, 2H). ^{13}C-NMR (125 MHz, DMSO-d_6) δ 159.44, 154.86, 152.52, 148.39, 143.60, 139.82, 129.42, 120.73, 119.94, 114.06, 111.39, 88.11, 86.08, 7.63, 70.84, 61.85, 55.04, 48.77, 32.83, 30.82. LC/ESI-MS (m/z): positive mode 416.0 $[M + H]^+$. Purity by HPLC-UV (254 nm)-ESI-MS: 94%.

(2*R*,3*S*,4*R*,5*R*)-2-(Hydroxymethyl)-5-(6-((3-(4-methoxyphenyl)propyl)amino)-9*H*-purin-9-yl)tetrahydrofuran-3,4-diol (14c)

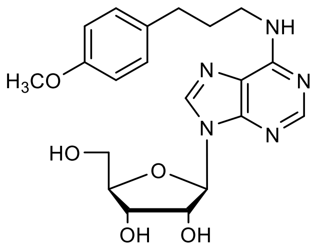

The compound was synthesized using 4-methoxybenzenepropanamine (**13c**, 290 mg, 1.75 mmol, 1.0 equiv) and afforded a white powder (310 mg, 43%), mp: 130 °C. ^{1}H-NMR (500 MHz, DMSO-d_6) δ 8.33 (s, 1H), 8.19 (br s, 1H), 7.92 (br s, 1H), 7.12 (d, 2H, *J* = 8.17 Hz), 6.82 (d, 2H, *J* = 8.25 Hz), 5.88 (s, 1H), 5.42 (s, 2H, overlapping CHO*H* & CHO*H*), 5.17 (s, 1H), 4.60 (s, 1H), 4.13 (s, 1H), 3.95 (s, 1H), 3.70 (s, 3H), 3.65-3.55 (d m, 2H), 3.48 (br s, 2H), 2.57 (s, 2H), 1.85 (s, 2H). ^{13}C-NMR (125 MHz, DMSO-d_6) δ 157.55, 154.91, 152.55, 148.42, 139.83, 133.84,

129.39, 120.01, 113.08, 88.14, 86.10, 73.64, 70.86, 61.88, 55.14, 48.79, 31.91, 31.19. LC/ESI-MS (m/z): positive mode 416.0 $[M + H]^+$. Purity by HPLC-UV (254 nm)-ESI-MS: 98.0%.

***N*-(6-((9-((2*R*,3*R*,4*S*,5*R*)-3,4-Dihydroxy-5-(hydroxymethyl)tetrahydrofuran-2-yl)-9*H*-purin-6-yl)amino)hexyl)benzamide (14d)**

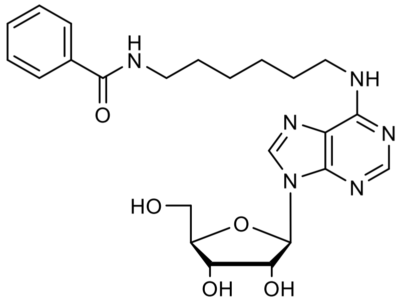

The compound was synthesized using **19** (1.10 g, 5.20 mmol) and purified by RP-HPLC (20-100% MeOH in H_2O in 20 min, 20 mL/min) yielding white powder (0.30 g, 17%), mp: 114 °C. ^{1}H-NMR (500 MHz, DMSO-*d₆*) *δ* 8.38 (t, 1H, *J* = 5.62 Hz), 8.31 (s, 1H), 8.18 (s, 1H), 7.81 (m, 2H), 7.48 (m, 1H), 7.43 (m, 2H), 5.87 (d, *J* = 6.14 Hz, 1H), 5.38 (d, 2H, *J* = 6.13 Hz), 5.13 (d, 1H, *J* = 4.61 Hz), 4.60 (q, 1H, *J* = 5.85 Hz), 4.14 (td, 1H, *J* = 3.01, 4.76 Hz), 3.95 (q, 1H, *J* = 3.46 Hz), 3.66-3.55 (d m, 2H), 3.46 (br s, 2H), 3.23 (m, 2H), 1.59 (m, 2H), 1.51 (m, 2H), 1.34 (m, 4H). ^{13}C-NMR (125 MHz, DMSO-d_6) *δ* 166.21, 154.82, 152.49, 148.38, 145.29, 139.71, 134.90, 131.05, 128.31, 127.23, 119.86, 88.10, 86.04, 73.60, 70.80, 61.83, 45.94, 39.29, 29.27, 29.20, 26.44, 26.32. LC/ESI-MS (m/z): positive mode 471.0 $[M + H]^+$. Purity by HPLC-UV (254 nm)-ESI-MS: 95.7%.

(3a*R*,4*R*,6*R*,6a*R*)-4-(Hydroxymethyl)-6-(6-((3-phenylpropyl)amino)-9*H*-purin-9-yl)-tetrahydrofuro[3,4-*d*][1,3]dioxole-2,2-diol (15a)

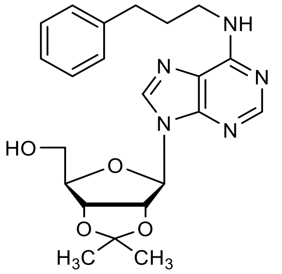

The compound was synthesized using **14a** (500 mg, 1.74 mmol) and afforded a colorless oil (470 mg, 68%). ^{1}H-NMR (500 MHz, DMSO-d_6) *δ* 8.32 (s, 1H), 8.21 (s, 1H), 7.90 (s, 1H), 7.22 (m, 5H), 6.12 (d, 1H, *J* = 3.07 Hz), 5.33 (dd, 1H, *J* = 3.06, 6.13 Hz), 5.19 (t, 1H, *J* = 5.34 Hz), 4.96 (dd, 1H, *J* = 2.51, 6,16 Hz), 4.21 (m, 1H), 3.53 (d m, 2H), 2.64 (m, 2H), 1.90 (m, 2H), 1.54 (s, 3H), 1.32 (s, 3H), 1.05 (t, 2H, *J* = 6.99 Hz). ^{13}CNMR (125 MHz, DMSO-d_6) *δ* 154.77, 152.71, 148.17, 141.93, 139.52, 128.39, 128.37, 125.79, 119.25, 113.18, 89.74, 86.50, 83.37, 81.49,

62.72, 56.14, 32.76, 30.86, 27.22, 25.34. LC/ESI-MS (m/z): positive mode 426.2 $[M + H]^+$. Purity by HPLCUV (254 nm)-ESI-MS: 84.2%.

(3a*R*,4*R*,6*R*,6a*R*)-4-(Hydroxymethyl)-6-(6-((3-(3-methoxyphenyl)propyl)amino)-9*H*-purin-9-yl)tetrahydrofuro[3,4-*d*][1,3]dioxole-2,2-diol (15b)

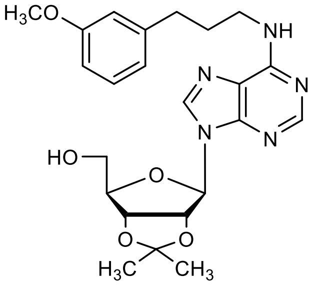

The compound was synthesized using **14b** (600 mg, 1.45 mmol) and afforded a colorless oil (300 mg, 86%). 1H-NMR (500 MHz, DMSO-*d*$_6$) δ 8.32 (s, 1H), 8.21 (br s, 1H), 7.89 (br s, 1H), 7.17 (t, 1H, *J* = 8.03 Hz), 6.78 (br m, 2H), 6.71 (d m, 1H), 6.12 (d, 1H, *J* = 3.08 Hz), 5.33 (dd, 1H, *J* = 3.09, 6.15 Hz), 5.19 (t, 1H, *J* = 4.94 Hz), 4.96 (dd, 1H, *J* = 2.50, 6.19 Hz), 4.20 (d m, 1H), 3.71 (s, 3H), 3.58-3.48 (br m, 4H, overlapping C*H*$_2$OH & NHC*H*$_2$), 2.68 (t, 2H), 1.89 (m, 2H), 1.54 (s, 3H), 1.32 (s, 3H). ^{13}C-NMR (125 MHz, DMSO-*d*$_6$) δ 159.41, 154.82, 152.78, 143.54, 139.53, 129.35, 120.67, 114.02, 113.16, 111.35, 89.77, 86.53, 83.40, 81.50, 61.73, 55.00, 32.78, 30.74, 27.22, 25.34. LC/ESI-MS (m/z): positive mode 456.1 $[M + H]^+$. Purity by HPLC-UV (254 nm)-ESI-MS: 92.8%.

(3a*R*,4*R*,6*R*,6a*R*)-4-(Hydroxymethyl)-6-(6-((3-(4-methoxyphenyl)propyl)amino)-9*H*-purin-9-yl)tetrahydrofuro[3,4-*d*][1,3]dioxole-2,2-diol (15c)

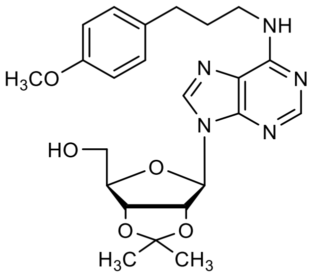

The compound was synthesized using **14c** (300 mg, 0.75 mmol) and afforded a colorless oil (110 mg, 30%). ^{1}H-NMR (500 MHz, DMSO-*d*$_6$) δ 8.32 (s, 1H), 8.21 (br s, 1H), 7.88 (br s, 1H), 7.12 (d, 2H, *J* = 8.61 Hz), 6.82 (d, 2H, *J* = 8.63 Hz), 6.11 (d, 1H, *J* = 3.08 Hz), 5.33 (dd, 1H, *J* = 3.12, 6.16 Hz), 5.19 (t, 1H, *J* = 5.60 Hz), 4.95 (dd, 1H, *J* = 2.53, 6.17 Hz), 4.20 (m, 1H), 3.70 (s, 3H), 3.57-3.49 (br m, 4H, overlapping C*H*$_2$OH & NHC*H*$_2$), 2.57 (m, 2H), 1.86 (m, 2H), 1.54 (s, 3H), 1.32 (s, 3H). ^{13}C-NMR (125 MHz, DMSO-*d*$_6$) δ 157.52, 154.80, 152.74, 139.53, 133.70, 129.35, 113.84, 113.84, 113.18, 89.78, 86.54, 83.41, 81.51, 61.74, 59.87, 55.10, 35.10, 31.86, 27.23, 25.35. LC/ESI-MS (m/z): positive mode 456.1 $[M + H]^+$. Purity by HPLC-UV (254 nm)-ESI-MS: 90.4%.

***N*-(6-((9-((3a*R*,4*R*,6*R*,6a*R*)-2,2-Dihydroxy-6-(hydroxymethyl)tetrahydrofuro[3,4-*d*][1,3]dioxol-4-yl)-9*H*-purin-6-yl)amino)hexyl)benzamide (15d)**

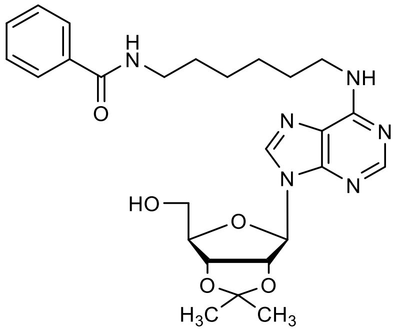

The compound was synthesized using **14d** (200 mg, 0.43 mmol) and afforded a colorless oil (190 mg, 84%). ^{1}H-NMR (500 MHz, DMSO-*d*$_6$) δ 8.39 (t, 1H, *J* = 5.45 Hz), 8.30 (s, 1H), 8.20 (br s, 1H), 7.84 (br s, 1H), 7.80 (m, 2H), 7.46 (m, 3H), 6.10 (d, 1H, *J* = 3.07 Hz), 5.32 (dd, 1H, *J* = 2.99, 5.98 Hz), 5.23 (br s, 1H), 4.95 (dd, 1H, *J* = 2.47, 6.13 Hz), 4.20 (m, 1H), 3.51 (m, 2H), 3.23 (d, 2H, *J* = 6.20 Hz), 3.16 (d, 2H, *J* = 4.94 Hz), 1.59 (m, 2H), 1.53 (s, 3H), 1.51(m, 2H), 1.33 (m, 4H), 1.31 (s, 3H). ^{13}C-NMR (125 MHz, DMSO-*d*$_6$) δ 166.33, 154.82, 152.85, 148.15, 139.57, 134.91, 131.15, 128.40, 127.28, 119.68, 113.26, 89.84, 86.57, 83.45, 81.55, 61.79, 56.23, 56.00, 29.31, 29.20, 27.27, 26.48, 26.37, 25.38. LC/ESI-MS (m/z): positive mode 511.1 [M + H]$^+$. Purity by HPLC-UV (254 nm)-ESI-MS: 96.8%.

***N*-(6-Aminohexyl)benzamide (19)**

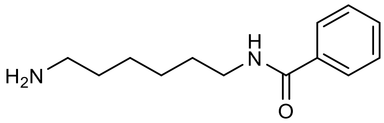

To a solution of benzoic acid (0.48 g, 3.95 mmol) in anhydrous THF (20 mL), HOBt (0.50 g, 3.95 mmol) and DCC (0.80 g, 3.95 mmol) were added. After activation, *N*-boc-1,6-hexandiamine (1.00 g, 3.95 mmol) in THF (10 mL) was added and the reaction was stirred at rt overnight. DCU (*N,N*-dichlorourethane) was filtered off and the filtrate was evaporated *in vacuum*. The intermediate **18** was purified by silica gel column chromatography (5% MeOH in DCM) yielding colorless oil (1.15 g, 91%). LC/ESI-MS (m/z): positive mode 321.3 [M + H]$^+$. Purity by HPLC-UV (254 nm)-ESI-MS: 90.4%. Compound **18** was taken up in DCM (10 mL) and TFA (0.6 mL, 6% w/w) was added. The reaction was stirred at rt for 2 days. TFA and DCM were added on a regular basis since both chemicals evaporated quickly due to the hot weather and high temperature in the lab. The reaction was carefully monitored by TLC (MeOH/DCM, 1:9). Evaporation followed by extraction with ethyl acetate afforded the compound as oil (2.40 g, 100%). ^{1}H-NMR (500 MHz, DMSO-*d*$_6$) δ 8.42 (t, 1H, *J* = 5.42 Hz), 7.81 (d, 2H, *J* = 7.08 Hz), 7.64 (br s, 2H), 7.50 (t, 1H, *J* = 7.89 Hz), 7.44 (t, 2H, *J* = 7.49 Hz), 3.25 (q, 2H, *J* = 6.86 Hz), 2.76 (dd, 2H, *J* = 6.68, 14.07 Hz), 1.51 (m, 4H), 1.32 (m, 4H). ^{13}C-NMR (125 MHz, DMSO-*d*$_6$) δ 134.87, 131.77, 128.39, 127.26, 116.65, 114.73, 33.50, 29.12, 27.17, 26.13, 25.67, 24.62,

21.20. LC/ESI-MS (m/z): positive mode 220.8 $[M + H]^+$. Purity by HPLC-UV (254 nm)-ESI-MS: 90.4%.

(2*R*,3*S*,4*R*,5*R*)-2-(Hydroxymethyl)-5-(6-(4-phenylbutoxy)-9*H*-purin-9-yl)tetrahydro-furan-3,4-diol (20a)

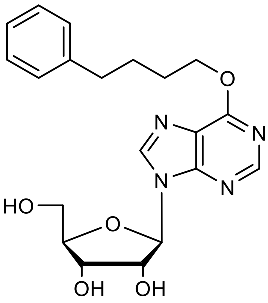

To 4-phenylbutanol (7 mL), small pieces of sodium were carefully added until a milky solution was generated. The sodium alkoxide was added to a suspension of **6** (500 mg, 1.74 mmol) in 4-phenylbutanol (5 mL). The reaction was refluxed for 2 h and the solvent was evaporated. Purification by silica gel column chromatography (10% MeOH in DCM) yielding the product as white solid (200 mg, 31%), mp: 90 °C. ^{1}H-NMR (500 MHz, DMSO-d_6), δ 8.59 (s, 1H), 8.51 (s, 1H), 7.21 (m, 5H), 5.97 (d, 1H, J = 5.75 Hz), 5.47 (d, 1H, J = 5.76 Hz), 5.19 (d, 1H, J = 4.82 Hz), 5.11 (t, 1H, J = 5.62 Hz), 4.58 (m, 3H, overlapping OCH_2 & CHCH_2), 4.16 (m, 1H), 3.96 (q, 1H, J = 3.78 Hz), 3.66-3.54 (d m, 2H), 2.65 (t, 2H, J = 7.58 Hz), 1.81 (m, 2H), 1.72 (m, 2H). ^{13}C-NMR (126 MHz, DMSO-d_6) δ 160.31, 151.95, 151.74, 142.43, 142.03, 128.42, 128.37, 125.82, 121.25, 87.92, 85.83, 73.88, 70.47, 66.57, 61.46, 34.85, 28.09, 27.47. LC/ESI-MS (m/z): positive mode 401.1 $[M + H]^+$. Purity by HPLC-UV (254 nm)-ESI-MS: 95%.

(2*R*,3*R*,4*S*,5*R*)-2-(6-(Butylthio)-9*H*-purin-9-yl)-5-(hydroxymethyl)tetrahydrofuran-3,4-diol (20b), CAS: 70421-25-9

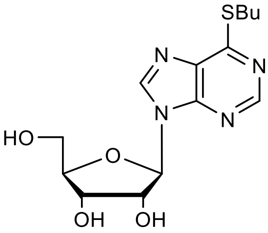

The compound was synthesized using 1-butanethiol (0.90 mL, 8.40 mmol) and afforded a yellowish solid (301 mg, 63%), mp: 51.0-53.0 °C (*lit.*[15] 60-63 °C). ^{1}H NMR (DMSO-d_6) δ 8.72 (s, 1H), 8.69 (s, 1H), 5.99 (d, J = 5.6 Hz, 1H), 5.48 (d, J = 5.9 Hz, 1H), 5.19 (d, J = 5.0 Hz, 1H), 5.08 (t, J = 5.6 Hz, 1H), 4.60 (q, J = 5.5 Hz, 1H), 4.23 – 4.16 (m, 1H), 4.01 – 3.93 (m, 1H), 3.74 – 3.64 (m, 1H), 3.63 – 3.53 (m, 1H), 3.42 – 3.33 (m, 2H), 1.76 – 1.65 (m, 2H), 1.44 (h, J = 7.4 Hz, 2H), 0.92 (t, J = 7.4 Hz, 3H). ^{13}C NMR (DMSO-d_6) δ 160.02, 151.44, 148.07, 143.08, 131.18,

87.76, 85.67, 73.72, 70.23, 61.22, 31.18, 27.49, 21.32, 13.45. LC-MS (*m/z*): positive mode 341.2 [M + H]$^+$. Purity by HPLC-UV (254 nm)-ESI-MS: 97.7%.

(2*R*,3*R*,4*S*,5*R*)-2-(6-(Cyclohexylthio)-9*H*-purin-9-yl)-5-(hydroxymethyl)tetrahydrofuran-3,4-diol (20c)

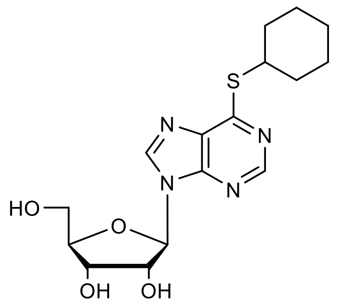

The compound was synthesized using cyclohexyl mercaptan (1.03 mL, 8.40 mmol) and afforded a yellowish solid (240 mg, 47%), mp: 78.0-80.0 °C (*lit.*[16] 165 °C). ^{1}H NMR (DMSO-d_6) δ 8.72 (s, 1H), 8.68 (s, 1H), 5.98 (d, J = 5.6 Hz, 1H), 5.48 (d, J = 5.9 Hz, 1H), 5.18 (d, J = 5.0 Hz, 1H), 5.08 (dd, J = 6.0, 5.1 Hz, 1H), 4.59 (q, J = 5.6 Hz, 1H), 4.25 – 4.13 (m, 2H), 3.97 (q, J = 3.9 Hz, 1H), 3.73 – 3.65 (m, 1H), 3.61 – 3.53 (m, 1H), 2.18 – 2.02 (m, 2H), 1.79 – 1.69 (m, 2H), 1.66 – 1.27 (m, 6H). ^{13}C NMR (126 MHz, DMSO) δ 159.84, 151.50, 148.18, 143.08, 130.99, 87.75, 85.66, 73.72, 70.23, 61.22, 41.01, 32.71, 32.67, 25.42, 25.09. LC-MS (*m/z*): positive mode 367.1 [M + H]$^+$. Purity by HPLC-UV (254 nm)-ESI-MS: 98.1%.

(2*R*,3*R*,4*S*,5*R*)-2-(6-Amino-8-chloro-9*H*-purin-9-yl)-5-(hydroxymethyl)tetrahydrofuran-3,4-diol (22)

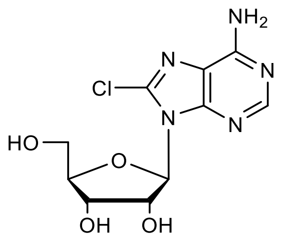

To a solution of adenosine (500 mg, 1.90 mmol) in DMF (4 mL), benzoyl chloride (0.20 mL, 2.00 mmol) was added. mCPBA (450 mg, 2.60 mmol) was dissolved in DMF (2 mL) and the solution was added to the reaction mixture. The reaction was stirred at rt for 30 min and then poured into cold H_2O. The resulting precipitate was filtered and washed with H_2O. The combined filtrate was washed with diethyl ether (3 × 20 mL) and evaporated to dryness. The resulting yellow syrup was purified by silica gel column chromatography (10% MeOH in DCM). Appropriate fractions were combined and evaporated, followed recrystallization with ethyl acetate yielding the desired compound as a white solid (200 mg, 40%), mp: 138 °C (*lit.*[17] 189-191 °C). ^{1}H-NMR (500 MHz, DMSO-d_6) δ 8.20 (s, 1H), 7.80 (br s, 2H), 5.85 (d, 1H, J = 6.59 Hz), 5.03 (dd, 1H, J = 5.20, 6.61 Hz), 4.19 (dd, 1H, J = 2.69, 5.24 Hz), 3.97 (m, 1H), 3.67-3.52

(d m, 2H). ^{13}C-NMR (125 MHz, DMSO-d_6) δ 154.44, 151.67, 149.67, 137.55, 118.05, 89.46, 86.77, 71.35, 70.83, 62.10. LC/ESI-MS (m/z): positive mode 302.1[M + H]$^+$. Purity by HPLC-UV (254 nm)-ESI-MS: 97.4%.

(2*R*,3*R*,4*S*,5*R*)-2-(6-Amino-8-bromo-9*H*-purin-9-yl)-5-(hydroxymethyl)tetrahydrofuran-3,4-diol (23)

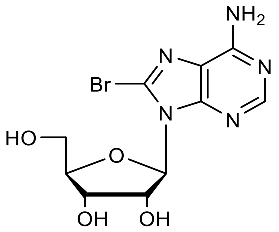

To a solution of adenosine (3.00 g, 11.23 mmol, 1 equiv.) in 1 M sodium acetate buffer (pH = 4, 5 mL) and H_2O (15 mL), Br_2 (1.44 mL, 28.08 mmol, 2.5 equiv.) was added dropwise. The mixture was stirred at rt overnight and monitored by TLC (MeOH/DCM, 1:9). After the reaction was completed, the mixture was decolorized by the addition of 1 M $NaHSO_3$, and then neutralized with 2 M NaOH. The solvent was concentrated *in vacuum* and cooled to 4 °C for crystallization, then filtered and washed with 5 mL H_2O to afford a yellowish solid (2.29 g, 59%), mp: 228.0-230.0 °C (*lit.*[18] >200 °C). ^{1}H NMR (600 MHz, DMSO-d_6) δ 8.11 (s, 1H), 7.53 (s, 2H), 5.82 (d, J = 6.7 Hz, 1H), 5.48 (dd, J = 3.9, 8.4 Hz, 1H), 5.45 (d, J = 7.2 Hz, 1H), 5.21 (d, J = 4.9 Hz, 1H), 5.07 (dd, J = 3.8, 6.8 Hz, 1H), 4.18 (m, 1H), 3.97 (d, J = 3.2 Hz, 1H), 3.67-3.51 (m, 2H). ^{13}C NMR (126 MHz, DMSO-d_6) δ 155.34, 152.61, 150.05, 127.35, 119.86, 90.59, 86.90, 71.30, 71.06, 62.30. LC-MS (*m/z*): negative mode 346.1 [M - H]$^-$. Purity by HPLC-UV (254 nm)-ESI-MS: 98.8%.

(2*R*,3*R*,4*S*,5*R*)-2-(6-Amino-8-(methylamino)-9*H*-purin-9-yl)-5-(hydroxymethyl)tetra-hydrofuran-3,4-diol (24a)

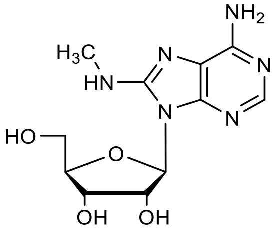

The compound was synthesized using **23** (600 mg, 1.73 mmol), 10 mL 40% aqueous methylamine and afforded a white solid (230 mg, 45%), mp: 215.0-217.0 °C (*lit.*[19] 217-218 °C). ^{1}H NMR (DMSO-d_6) δ 7.89 (s, 1H), 6.93 (q, J = 4.6 Hz, 1H), 6.51 (s, 2H), 5.88 (dd, J = 24.5, 6.2 Hz, 2H), 5.23 (d, J = 6.6 Hz, 1H), 5.13 (d, J = 4.1 Hz, 1H), 4.71 – 4.60 (m, 1H), 4.18 – 4.09 (m, 1H), 3.96 (d, J = 2.4 Hz, 1H), 3.70 – 3.56 (m, 2H), 2.88 (d, J = 4.5 Hz, 3H). ^{13}C NMR (DMSO-d_6) δ 152.39, 152.05, 149.82, 148.48, 117.12, 86.50, 85.68, 70.95, 70.72, 61.64,

29.11. LC-MS (*m/z*): positive mode 297.2 [M + H]$^+$. Purity by HPLC-UV (254 nm)-ESI-MS: 98.9%.

(2*R*,3*R*,4*S*,5*R*)-2-(6-Amino-8-(butylamino)-9*H*-purin-9-yl)-5-(hydroxymethyl)tetra-hydrofuran-3,4-diol (24b)

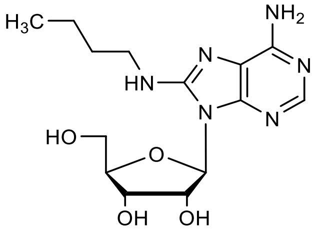

The compound was synthesized using **23** (500 mg, 1.44 mmol), 10 mL butylamine and afforded a white solid (480 mg, 100%), mp: 168 °C. ^{1}H-NMR (500 MHz, DMSO-*d$_6$*) *δ* 7.87 (s, 1H), 6.83 (t, 1H, *J* = 5.4 Hz), 6.44 (s, 2H), 5.89 (d, 1H, *J* = 7.3 Hz), 5.83 (dd, 1H, *J* = 4.3, 5.7 Hz), 5.19 (d, 1H, *J* = 6.7 Hz), 5.11 (d, 1H, *J* = 4.0 Hz), 4.62 (m, 1H), 4.10 (m, 1H), 3.95 (q, 1H, *J* = 2.3 Hz), 3.62 (dt, 2H, *J* = 2.7, 5.8 Hz), 3.37 (dd, 1H, *J* = 6.8, 12.7 Hz, overlapping with H_2O), 3.28 (dd, 1H, *J* = 6.9, 12.7 Hz, overlapping with H_2O), 1.56 (m, 2H), 1.34 (m, 2H), 0.9 (t, 3H, *J* = 7.3 Hz). ^{13}C-NMR (125 MHz, DMSO-*d$_6$*) *δ* 152.43, 151.51, 149.94, 148.56, 117.23, 86.50, 85.81, 71.13, 70.85, 61.80, 42.19, 31.03, 19.83, 13.95. LC/ESI-MS (m/z): positive mode 339.1 [M + H]$^+$. Purity by HPLC-UV (254 nm)-ESI-MS: 94.7%.

(2*R*,3*R*,4*S*,5*R*)-2-(6-Amino-8-(cyclopropylamino)-9*H*-purin-9-yl)-5-(hydroxymethyl)tetrahydrofuran-3,4-diol (24c)

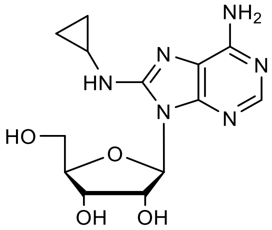

The compound was synthesized using **23** (400 mg, 1.16 mmol), 10 mL cyclopropylamine and afforded a light brown solid (100 mg, 27%), mp: 200.0-202.0 °C. ^{1}H NMR (DMSO-*d*$_6$) *δ* 7.90 (s, 1H), 6.53 (s, 2H), 5.87 (dd, *J* = 8.0, 4.0 Hz, 2H), 5.26 – 5.06 (m, 2H), 4.56 (q, *J* = 5.6 Hz, 1H), 4.09 (d, *J* = 5.3 Hz, 1H), 3.94 (q, *J* = 2.3 Hz, 1H), 3.62 (q, *J* = 2.8 Hz, 2H), 2.99 (q, *J* = 7.3 Hz, 1H), 1.15 (t, *J* = 7.3 Hz, 1H), 0.69 – 0.66 (m, 2H), 0.57 – 0.47 (m, 2H). ^{13}C NMR (DMSO-*d*$_6$) *δ* 152.56, 151.56, 149.81, 148.63, 117.05, 86.32, 85.60, 70.90, 70.63, 61.57, 24.86, 6.69, 6.03. LC-MS (*m/z*): positive mode 323.2 [M + H]$^+$. Purity by HPLC-UV (254 nm)-ESI-MS: 98.2%.

(2*R*,3*R*,4*S*,5*R*)-2-(6-Amino-8-((4-phenylbutyl)amino)-9*H*-purin-9-yl)-5-(hydroxyl-methyl)tetrahydrofuran-3,4-diol (24d), CAS: 402724-85-0

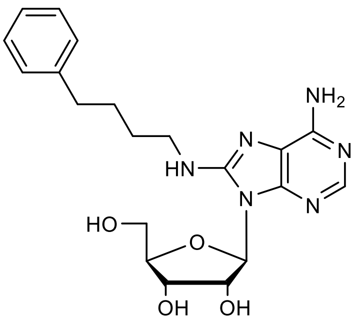

To a solution of **23** (500 mg, 1.44 mmol, 1 equiv.) in H_2O/EtOH (1:3, 15 mL), 4-phenylbutylamine (0.3 ml, 2.2 mmol, 1.5 equiv) and Et_3N (0.4 mL, 2.9 mmol, 2 equiv.) were added. The mixture was refluxed for 36 h and monitored by TLC (MeOH/DCM, 1:9). After the reaction was completed, cooled to rt and the solvent was evaporated *in vacuum*. The crude mixture was purified by silica gel column chromatography (8% MeOH in DCM) yielding a white solid (270 mg, 54%), mp: 108 °C. ^{1}H-NMR (500 MHz, DMSO-d_6) δ 7.87 (s, 1H), 7.25 (t, 2H, J = 7.6 Hz), 7.19 (d, 2H, J = 7.1 Hz), 7.15 (t, 1H, J = 7.3 Hz), 6.89 (t, 1H, J = 5.5 Hz), 6.44 (s, 2H), 5.88 (d, 1H, J = 7.4 Hz), 5.85 (t, 1H, J = 4.9 Hz), 5.20 (d, 1H, J = 6.8 Hz), 5.12 (d, 1H, J = 4.0 Hz), 4.63 (q, 1H, J = 6.9 Hz), 4.10 (m, 1H), 3.95 (d, 1H, J = 2.1 Hz), 3.61 (m, 2H), 2.60 (t, 2H, J = 7.0 Hz), 1.61 (m, 4H). ^{13}C-NMR (125 MHz, DMSO-d_6) δ 152.46, 151.54, 149.94, 148.60, 142.39, 128.48, 128.42, 125.82, 117.26, 86.55, 85.87, 71.16, 70.90, 61.87, 56.22, 42.27, 35.08, 28.62. LC/ESI-MS (m/z): positive mode 415.0 $[M + H]^+$. Purity by HPLC-UV (254 nm)-ESI-MS: 95.5%.

(2*R*,3*R*,4*S*,5*R*)-2-(6-Amino-8-methoxy-9*H*-purin-9-yl)-5-(hydroxymethyl)tetrahydrofuran-3,4-diol (26a), CAS: 3969-27-5

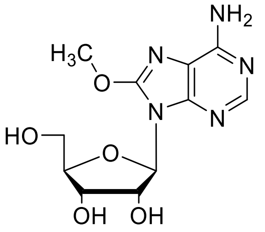

To a solution of 8-bromoadenosine (**23**, 500 mg, 1.44 mmol, 1 equiv.) in MeOH (15 mL), NaOMe (778 mg, 14.40 mmol, 10 equiv.) was added. The mixture was refluxed overnight, and the reaction progress was monitored by TLC (MeOH/DCM, 1:9). After the reaction was completed, cooled to rt, 5 g silica gel was added, and the solvent was evaporated *in vacuum*. The crude compound was purified by silica gel column chromatography (12% MeOH in DCM) yielding a yellowish solid (195 mg, 46%), mp: 166.5-168.5 °C (*lit.*[20] 206-208 °C). ^{1}H NMR (DMSO-d_6) δ 8.02 (s, 1H), 6.95 (s, 2H), 5.71 (d, J = 6.6 Hz, 1H), 5.47 – 5.28 (m, 2H), 5.10 (d, J = 4.7 Hz, 1H), 4.96 – 4.83 (m, 1H), 4.20 – 4.07 (m, 4H), 3.97 – 3.86 (m, 1H), 3.62 (dt, J = 12.0, 4.1 Hz, 1H), 3.58 – 3.41 (m, 1H). ^{13}C NMR (DMSO-d_6) δ 154.39, 154.03, 150.53, 148.69,

114.87, 86.67, 85.96, 71.04, 70.84, 62.18, 57.20. LC-MS (*m/z*): positive mode 298.2 $[M + H]^+$. Purity by HPLC-UV (254 nm)-ESI-MS: 100%.

(2*R*,3*R*,4*S*,5*R*)-2-(6-Amino-8-butoxy-9*H*-purin-9-yl)-5-(hydroxymethyl)tetrahydrofuran-3,4-diol (26b), CAS: 255716-03-1

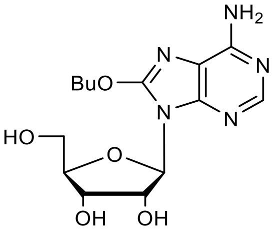

The compound was synthesized using **23** (200 mg, 0.58 mmol), 8 mL butanol and afforded a yellowish solid (112 mg, 57%), mp: 180.0-182.0 °C (*lit.*[21] 173 °C). ^{1}H NMR (DMSO-d_6) δ 8.02 (s, 1H), 6.90 (s, 2H), 5.72 (d, J = 6.6 Hz, 1H), 5.38 (dd, J = 8.2, 4.3 Hz, 1H), 5.31 (d, J = 6.2 Hz, 1H), 5.10 (d, J = 4.6 Hz, 1H), 4.87 (q, J = 6.1 Hz, 1H), 4.52 – 4.45 (m, 2H), 4.17 – 4.08 (m, 1H), 3.90 (q, J = 3.9 Hz, 1H), 3.63 (dt, J = 12.0, 4.1 Hz, 1H), 3.53 – 3.43 (m, 1H), 1.85 – 1.71 (m, 2H), 1.44 (h, J = 7.4 Hz, 2H), 0.95 (t, J = 7.4 Hz, 3H). ^{13}C NMR (DMSO-d_6) δ 153.91, 153.85, 150.42, 148.57, 114.88, 86.64, 85.85, 71.04, 70.82, 69.85, 62.18, 30.24, 18.48, 13.53. LC-MS (*m/z*): positive mode 340.2 $[M + H]^+$. Purity by HPLC-UV (254 nm)-ESI-MS: 98.4%.

(2*R*,3*R*,4*S*,5*R*)-2-(6-Amino-8-(cyclopentyloxy)-9*H*-purin-9-yl)-5-(hydroxymethyl)tetrahydrofuran-3,4-diol (26c)

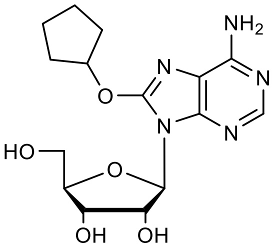

The compound was synthesized using **23** (300 mg, 0.87 mmol), 8 mL cyclopentanol and afforded a yellowish solid (86 mg, 28%), mp: 63.0-65.0 °C. ^{1}H NMR (DMSO-d_6) δ 8.01 (s, 1H), 6.89 (s, 2H), 5.70 (d, J = 6.4 Hz, 1H), 5.51 – 5.44 (m, 1H), 5.31 (d, J = 6.2 Hz, 1H), 5.11 (d, J = 4.7 Hz, 1H), 4.85 – 4.78 (m, 1H), 4.15 – 4.06 (m, 1H), 3.91 – 3.85 (m, 1H), 3.62 (dt, J = 12.0, 4.2 Hz, 1H), 3.53 – 3.40 (m, 2H), 2.03 – 1.83 (m, 4H), 1.70 – 1.55 (m, 4H). ^{13}C NMR (DMSO-d_6) δ 153.85, 153.33, 150.35, 148.54, 114.99, 86.61, 85.69, 82.98, 71.07, 70.78, 62.17, 34.98, 32.18, 23.10. LC-MS (*m/z*): positive mode 352.1 $[M + H]^+$. Purity by HPLC-UV (254 nm)-ESI-MS: 98.2%.

(2*R*,3*R*,4*S*,5*R*)-2-(6-Amino-8-mercapto-9*H*-purin-9-yl)-5-(hydroxymethyl)tetrahydrofuran-3,4-diol (29)

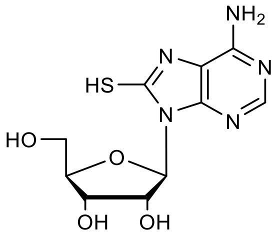

To a solution of **23** (500 mg, 1.40 mmol) in DMF (5 mL), NaHS (800 mg, 14.40 mmol) was added. The mixture was stirred at 100 °C for 5 h until TLC analysis (MeOH/DCM, 1:4) indicated that the reaction was completed. The mixture was cooled down to rt and treated with MeOH followed by filtration. The filtrate was evaporated and co-evaporated with MeOH. The remaining residue was taken up in H_2O, neutralized with 1 M HCl and lyophilized. The crude product was taken up in H_2O and extracted with ethyl acetate. The organic layers were combined, dried over $MgSO_4$, and reduced *in vacuum* yielding the desired product as brown solid (260 mg, 59%), mp: 216 °C. ^{1}H-NMR (500 MHz, DMSO-*d$_6$*) δ 12.52 (s, 1H), 8.11 (s, 1H), 6.95 (br s, 2H), 6.33 (d, 1H, *J* = 6.3 Hz), 5.22 (d, 1H, *J* = 6.1 Hz), 5.18 (dd, 1H, *J* = 4.1, 8.2 Hz), 5.08 (d, 1H, *J* = 4.7 Hz), 4.99 (q, 1H, *J* = 5.9 Hz), 4.21 (m, 1H), 3.89 (q, 1H, *J* = 3.9 Hz), 3.65-3.49 (d m, 2H). ^{13}C-NMR (125 MHz, DMSO-*d$_6$*) δ 168.21, 152.20, 148.44, 148.18, 107.33, 88.95, 85.90, 71.01, 70.95, 62.44. LC/ESI-MS (m/z): positive mode 300.0 $[M + H]^+$. Purity by HPLC-UV (254 nm)-ESI-MS: 99%.

(2*R*,3*R*,4*S*,5*R*)-2-(6-Amino-8-(methylthio)-9*H*-purin-9-yl)-5-(hydroxymethyl)tetra-hydrofuran-3,4-diol (30a), CAS 29836-01-9

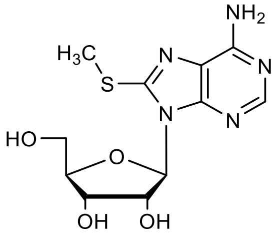

The compound was synthesized using methyliodide (0.30 mL, 4.30 mmol, 3 equiv.). The precipitate was filtered off and was washed with EtOH and afforded a white solid (360 mg, 80%), mp: 234 °C (*lit.*[22] 235-237 °C). ^{1}H-NMR (500 MHz, DMSO-*d$_6$*) δ 8.04 (s, 1H), 7.21 (s, 2H), 5.72 (d, 1H, *J* = 6.86 Hz), 5.56 (dd, 1H, *J* = 3.70, 8.65 Hz), 5.36 (d, 1H, *J* = 5.07 Hz), 5.15 (br s, 1H), 4.98 (m, 1H), 4.15 (br s, 1H), 3.95 (m, 1H), 3.52-3.66 (d m, 2H), 2.71 (s, 3H). ^{13}C-NMR (125 MHz, DMSO-*d$_6$*) δ 154.58, 151.32, 150.85, 149.84, 119.70, 88.89, 88.72, 71.43, 71.07, 62.34, 14.77. LC/ESI-MS (m/z): positive mode 314.1 $[M + H]^+$. Purity by HPLC-UV (254 nm)-ESI-MS: 96.8%.

(2*R*,3*R*,4*S*,5*R*)-2-(6-Amino-8-(ethylthio)-9*H*-purin-9-yl)-5-(hydroxymethyl)tetrahydrofuran-3,4-diol (30b), CAS: 63614-44-8

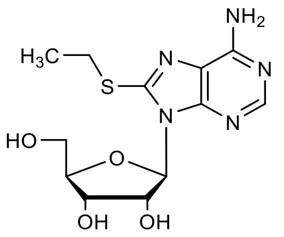

To a solution of 8-bromoadenosine (**23**, 400 mg, 1.16 mmol, 1 equiv.) in anhydrous DMF (10 mL), sodium ethanethiolate (293 mg, 3.48 mmol, 3 equiv.) was added. The mixture was stirred at rt overnight and the reaction progress was monitored by TLC (MeOH/DCM, 1:9). After the reaction was completed, 5 g silica gel was added, and the solvent was evaporated *in vacuum*. The crude compound was purified by silica gel column chromatography (8% MeOH in DCM) yielding a yellowish solid (266 mg, 70%), mp: 175.0-177.0 °C (*lit.*[23] 176 °C). ^{1}H NMR (DMSO-d_6) δ 8.05 (s, 1H), 7.27 (s, 2H), 5.76 (d, J = 6.9 Hz, 1H), 5.62 (dd, J = 8.9, 3.7 Hz, 1H), 5.38 (d, J = 6.5 Hz, 1H), 5.16 (d, J = 4.4 Hz, 1H), 5.07 – 4.92 (m, 1H), 4.25 – 4.07 (m, 1H), 4.00 – 3.90 (m, 1H), 3.72 – 3.44 (m, 2H), 2.89 (s, 1H), 2.73 (s, 1H), 1.35 (q, J = 6.8, 6.2 Hz, 3H). ^{13}C NMR (DMSO-d_6) δ 154.56, 151.27, 150.39, 148.48, 119.63, 88.85, 86.58, 71.25, 70.97, 62.21, 26.76, 14.81. LC-MS (*m/z*): positive mode 328.0 [M + H]$^+$. Purity by HPLC-UV (254 nm)-ESI-MS: 98.7%.

(2*R*,3*R*,4*S*,5*R*)-2-(6-Amino-8-(*tert*-butylthio)-9*H*-purin-9-yl)-5-(hydroxymethyl)tetrahydrofuran-3,4-diol (30c), CAS: 127236-49-1

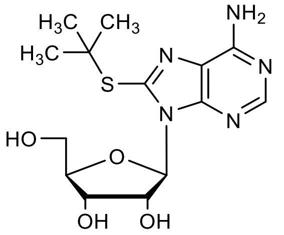

The compound was synthesized using **23** (600 mg, 1.73 mmol), 2-methyl-2-propanethiol (0.59 mL, 5.19 mmol) and afforded a yellowish solid (125 mg, 20%), mp: 217.0-218.0 °C. ^{1}H NMR (DMSO-d_6) δ 8.09 (s, 1H), 7.44 (s, 2H), 6.06 (d, J = 6.9 Hz, 1H), 5.65 (dd, J = 9.2, 3.5 Hz, 1H), 5.27 (d, J = 6.5 Hz, 1H), 5.12 (d, J = 4.1 Hz, 1H), 5.07 – 4.97 (m, 1H), 4.23 – 4.14 (m, 1H), 4.01 – 3.89 (m, 1H), 3.73 – 3.62 (m, 1H), 3.58 – 3.47 (m, 1H), 1.45 (s, 9H). ^{13}C NMR (DMSO-d_6) δ 155.48, 152.08, 149.44, 145.73, 119.81, 89.17, 86.51, 71.33, 71.11, 62.30, 50.32, 30.80. LC-MS (*m/z*): positive mode 356.2 [M + H]$^+$. Purity by HPLC-UV (254 nm)-ESI-MS: 97.6%.

(2*R*,3*R*,4*S*,5*R*)-2-(6-Amino-8-(propylthio)-9*H*-purin-9-yl)-5-(hydroxymethyl)tetrahydrofuran-3,4-diol (30d)

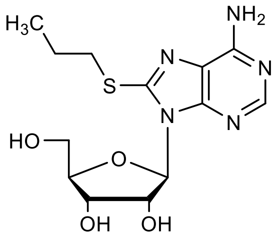

The compound was synthesized using **23** (500 mg, 1.44 mmol), 1-propanethiol (0.39 mL, 4.32 mmol) and afforded a milk white solid (183 mg, 37%), mp: 189.5-191.0 °C. ^{1}H NMR (DMSO-d_6) δ 8.05 (d, J = 1.2 Hz, 1H), 7.24 (s, 2H), 5.78 (dd, J = 6.9, 1.1 Hz, 1H), 5.66 – 5.56 (m, 1H), 5.36 (dd, J = 6.6, 1.2 Hz, 1H), 5.15 (dd, J = 4.4, 1.2 Hz, 1H), 5.09 – 4.94 (m, 1H), 4.22 – 4.10 (m, 1H), 3.96 (dd, J = 4.1, 2.5 Hz, 1H), 3.74 – 3.63 (m, 1H), 3.58 – 3.48 (m, 1H), 3.30 – 3.21 (m, 2H), 1.84 – 1.63 (m, 2H), 1.05 – 0.95 (m, 3H). ^{13}C NMR (DMSO-d_6) δ 154.52, 151.24, 150.40, 148.65, 119.58, 88.85, 86.57, 71.24, 70.96, 62.20, 34.23, 22.26, 12.99. LC-MS (*m/z*): positive mode 342.1[M + H]$^{+}$. Purity by HPLC-UV (254 nm)-ESI-MS: 100%.

(2*R*,3*R*,4*S*,5*R*)-2-(6-Amino-8-(isopentylthio)-9*H*-purin-9-yl)-5-(hydroxymethyl)tetrahydrofuran-3,4-diol (30e)

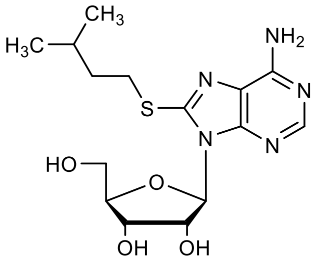

The compound was synthesized using **23** (500 mg, 1.44 mmol), 3-methyl-1-butanethiol (0.54 mL, 4.32 mmol) and afforded a yellowish solid (208 mg, 39%), mp: 155.0-156.0 °C. ^{1}H NMR (DMSO-d_6) δ 8.06 (s, 1H), 7.23 (s, 2H), 5.77 (d, J = 6.9 Hz, 1H), 5.60 (dd, J = 8.9, 3.8 Hz, 1H), 5.36 (d, J = 6.4 Hz, 1H), 5.15 (d, J = 4.4 Hz, 1H), 5.06 – 4.92 (m, 1H), 4.24 – 4.10 (m, 1H), 4.02 – 3.93 (m, 1H), 3.67 (dt, J = 12.2, 3.8 Hz, 1H), 3.59 – 3.47 (m, 1H), 3.36 – 3.30 (m, 1H), 3.29 – 3.24 (m, 1H), 1.70 (dq, J = 13.3, 6.7 Hz, 1H), 1.63 – 1.54 (m, 2H), 0.91 (d, J = 6.6 Hz, 6H). ^{13}C NMR (DMSO-d_6) δ 154.53, 151.25, 150.38, 148.62, 119.60, 88.85, 86.56, 71.24, 70.96, 62.19, 37.67, 30.57, 26.78, 22.05, 22.02. LC-MS (*m/z*): positive mode 370.2 [M + H]$^{+}$. Purity by HPLC-UV (254 nm)-ESI-MS: 100%.

(2*R*,3*R*,4*S*,5*R*)-2-(6-Amino-8-((2-methylbutyl)thio)-9*H*-purin-9-yl)-5-(hydroxymethyl)tetrahydrofuran-3,4-diol (30f)

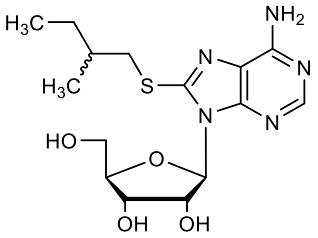

The compound was synthesized using **23** (500 mg, 1.44 mmol), 2-methyl-1-butanethiol (0.53 mL, 4.32 mmol) and afforded a yellowish solid (254 mg, 48%), mp: 165.0-166.5 °C. ^{1}H NMR (DMSO-d_6) δ 8.05 (s, 1H), 7.24 (s, 2H), 5.79 (d, J = 6.9 Hz, 1H), 5.62 (dd, J = 8.9, 3.7 Hz, 1H), 5.38 (d, J = 6.4 Hz, 1H), 5.17 (d, J = 4.3 Hz, 1H), 5.05 – 4.96 (m, 1H), 4.23 – 4.12 (m, 1H), 4.03 – 3.92 (m, 1H), 3.67 (dt, J = 12.1, 3.8 Hz, 1H), 3.59 – 3.44 (m, 1H), 3.35 – 3.31 (m, 1H), 3.25 (dd, J = 12.7, 7.0 Hz, 1H), 1.85 – 1.73 (m, 1H), 1.62 – 1.44 (m, 1H), 1.33 – 1.21 (m, 1H), 0.99 (d, J = 6.7 Hz, 3H), 0.89 (t, J = 7.4 Hz, 3H). ^{13}C NMR (DMSO-d_6) δ 154.49, 151.22, 150.43, 148.99, 119.54, 88.85, 86.60, 71.26, 71.00, 62.21, 38.97, 34.10, 27.85, 18.46, 11.05. LC-MS (*m/z*): positive mode 370.3 [M + H]$^+$. Purity by HPLC-UV (254 nm)-ESI-MS: 100%.

(2*R*,3*R*,4*S*,5*R*)-2-(6-Amino-8-(pentylthio)-9*H*-purin-9-yl)-5-(hydroxymethyl)tetrahydrofuran-3,4-diol (30g), CAS: 68807-85-2

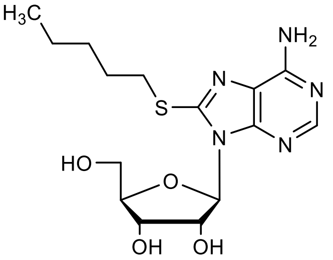

The compound was synthesized using **23** (400 mg, 1.16 mmol), 1-pentanethiol (0.43 mL, 3.48 mmol) and afforded a yellowish solid (194 mg, 45%), mp: 172.5-174.5 °C. ^{1}H NMR (DMSO-d_6) δ 8.05 (s, 1H), 7.25 (s, 2H), 5.77 (d, J = 6.9 Hz, 1H), 5.62 (dd, J = 8.9, 3.7 Hz, 1H), 5.37 (d, J = 6.4 Hz, 1H), 5.16 (d, J = 4.3 Hz, 1H), 5.09 – 4.94 (m, 1H), 4.22 – 4.11 (m, 1H), 4.00 – 3.92 (m, 1H), 3.67 (dt, J = 12.2, 3.7 Hz, 1H), 3.59 – 3.45 (m, 1H), 3.36 – 3.31 (m, 1H), 3.29 – 3.24 (m, 1H), 1.70 (p, J = 7.3 Hz, 2H), 1.41 – 1.28 (m, 4H), 0.87 (t, J = 7.2 Hz, 3H). ^{13}C NMR (DMSO-d_6) δ 154.53, 151.25, 150.40, 148.69, 119.60, 88.85, 86.58, 71.25, 70.98, 62.21, 32.32, 30.19, 28.49, 21.59, 13.80. LC-MS (*m/z*): positive mode 370.1 [M + H]$^+$. Purity by HPLC-UV (254 nm)-ESI-MS: 99.8%.

(2*R*,3*R*,4*S*,5*R*)-2-(6-Amino-8-((5-methylhexyl)thio)-9*H*-purin-9-yl)-5-(hydroxymethyl)-tetrahydrofuran-3,4-diol (30h)

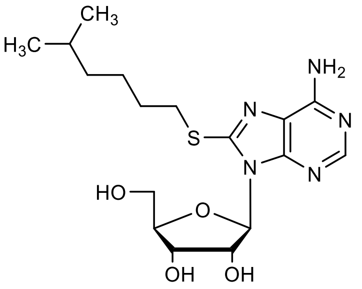

The compound was synthesized using 1-bromo-5-methylhexane (0.22 mL, 1.34 mmol, 2 equiv.) and afforded a white solid (80 mg, 30%), mp: 180 °C. ^{1}H-NMR (500 MHz, DMSO-d_6) δ 8.04 (s, 1H), 7.22 (s, 2H), 5.77 (d, 1H, J = 6.9 Hz), 5.59 (dd, 1H, J = 3.7, 8.9 Hz), 5.35 (d, 1H, J = 6.2 Hz), 5.14 (d, 1H, J = 4.3 Hz), 4.99 (q, 1H, J = 6.2 Hz), 4.15 (m, 1H), 3.95 (td, 1H, J = 2.2, 3.8 Hz), 3.66-3.51 (d m, 2H), 3.28 (m, 2H, SCH_2 overlapping with H_2O), 1.67 (m, 2H), 1.49 (m, 1H), 1.39 (m, 2H), 1.16 (m, 2H), 0.84 (s, 3H), 0.83 (s, 3H). ^{13}C-NMR (126 MHz, DMSO-d_6) δ 154.69, 151.41, 150.56, 148.86, 119.76, 89.03, 86.74, 71.43, 71.14, 62.37, 37.95, 32.58, 29.25, 27.47, 25.98, 22.58. LC/ESI-MS (m/z): positive mode 398.0 [M + H]$^+$. Purity by HPLC-UV (254 nm)-ESI-MS: 99%.

(2*R*,3*R*,4*S*,5*R*)-2-(6-Amino-8-(cyclopentylthio)-9*H*-purin-9-yl)-5-(hydroxymethyl)tetrahydrofuran-3,4-diol (30i)

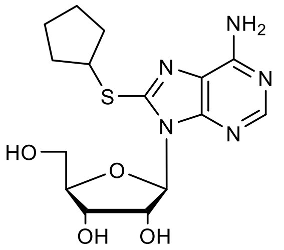

The compound was synthesized using **23** (500 mg, 1.44 mmol), thiourea (219 mg, 2.88 mmol) and bromocyclopentane (0.15 mL, 2.88 mmol) and afforded a white solid (90 mg, 17%), mp: 66.0-68.0 °C. ^{1}H NMR (DMSO-d_6) δ 8.06 (s, 1H), 7.30 (s, 2H), 6.55 (s, 1H), 5.81 (d, J = 6.9 Hz, 1H), 5.70 – 5.60 (m, 1H), 5.37 (d, J = 6.4 Hz, 1H), 5.16 (d, J = 4.3 Hz, 1H), 5.07 – 4.96 (m, 1H), 4.22 – 4.12 (m, 1H), 3.99 – 3.93 (m, 1H), 3.71 – 3.60 (m, 1H), 3.57 – 3.49 (m, 1H), 2.25 – 2.05 (m, 2H), 1.84 – 1.54 (m, 6H). ^{13}C NMR (DMSO-d_6) δ 154.76, 151.46, 150.08, 148.36, 119.73, 88.99, 86.61, 71.27, 71.03, 62.24, 46.21, 33.35, 32.90, 24.28, 24.17. LC-MS (*m/z*): positive mode 367.9 [M + H]$^+$. Purity by HPLC-UV (254 nm)-ESI-MS: 97.0%.

(2*R*,3*R*,4*S*,5*R*)-2-(6-Amino-8-(cyclohexylthio)-9*H*-purin-9-yl)-5-(hydroxymethyl)tetrahydrofuran-3,4-diol (30j), CAS: 171502-16-2

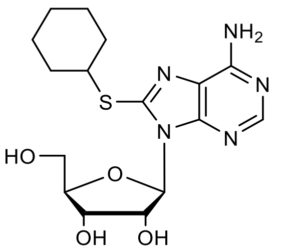

The compound was synthesized using **23** (600 mg, 1.73 mmol), cyclohexanethiol (0.63 mL, 5.19 mmol) and afforded a yellowish solid (124 mg, 19%), mp: 149.5-151.5 °C (*lit.*[24] 207-208 °C). ^{1}H NMR (DMSO-d_6) δ 8.06 (s, 1H), 7.32 (s, 2H), 5.85 (d, *J* = 6.9 Hz, 1H), 5.65 (ddd, *J* = 9.1, 3.6, 1.2 Hz, 1H), 5.36 (d, *J* = 6.4 Hz, 1H), 5.16 (d, *J* = 4.2 Hz, 1H), 5.00 (q, *J* = 6.3 Hz, 1H), 4.23 – 4.10 (m, 1H), 3.96 (q, *J* = 3.4 Hz, 1H), 3.85 – 3.74 (m, 1H), 3.67 (dt, *J* = 12.1, 3.7 Hz, 1H), 3.52 (ddd, *J* = 12.5, 9.0, 3.9 Hz, 1H), 2.16 – 1.92 (m, 2H), 1.78 – 1.66 (m, 2H), 1.59 – 1.25 (m, 6H). ^{13}C NMR (DMSO-d_6) δ 154.79, 151.49, 150.04, 147.42, 119.71, 88.96, 86.60, 71.27, 71.04, 62.24, 46.62, 32.95, 32.55, 25.28, 25.17, 25.00. LC-MS (*m/z*): positive mode 381.9 [M + H]$^+$. Purity by HPLC-UV (254 nm)-ESI-MS: 99.9%.

(2*R*,3*R*,4*S*,5*R*)-2-(6-Amino-8-((cyclohexylmethyl)thio)-9*H*-purin-9-yl)-5-(hydroxymethyl)tetrahydrofuran-3,4-diol (30k), CAS: 121059-93-6

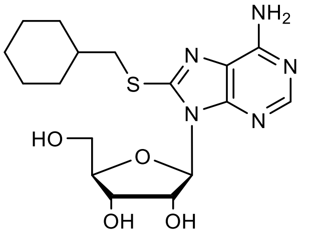

The compound was synthesized using **23** (300 mg, 0.87 mmol), thiourea (132 mg, 1.74 mmol) and cyclohexylmethyl bromide (0.24 mL, 1.74 mmol) and afforded a white solid (195 mg, 57%), mp: 183.8-185.8 °C. ^{1}H NMR (DMSO-d_6) δ 8.05 (s, 1H), 7.25 (s, 2H), 5.78 (d, *J* = 6.9 Hz, 1H), 5.62 (dd, *J* = 8.9, 3.7 Hz, 1H), 5.38 (d, *J* = 6.4 Hz, 1H), 5.16 (d, *J* = 4.3 Hz, 1H), 5.06 – 4.95 (m, 1H), 4.22 – 4.10 (m, 1H), 4.02 – 3.92 (m, 1H), 3.74 – 3.63 (m, 1H), 3.58 – 3.47 (m, 1H), 3.30 – 3.19 (m, 2H), 1.91 – 1.77 (m, 2H), 1.74 – 1.56 (m, 4H), 1.26 – 1.11 (m, 3H), 1.07 – 0.98 (m, 2H). ^{13}C NMR (DMSO-d_6) δ 154.47, 151.20, 150.45, 149.03, 119.54, 88.83, 86.59, 71.26, 70.99, 62.21, 37.12, 31.81, 31.76, 25.73, 25.39. LC-MS (*m/z*): positive mode 396.0 [M + H]$^+$. Purity by HPLC-UV (254 nm)-ESI-MS: 99.0%.

(2*R*,3*R*,4*S*,5*R*)-2-(6-Amino-8-(thiophen-2-ylthio)-9*H*-purin-9-yl)-5-(hydroxymethyl)tetrahydrofuran-3,4-diol (30l)

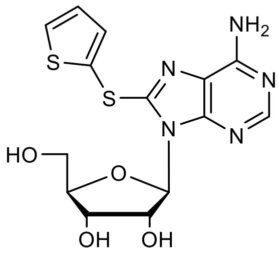

The compound was synthesized using **23** (500 mg, 1.44 mmol), 2-thiophenethiol (0.40 mL, 4.32 mmol) and afforded a yellowish solid (453 mg, 82%), mp: 121.0-123.0 °C. ^{1}H NMR (DMSO-d_6) δ 8.11 (s, 1H), 7.79 (dd, J = 5.3, 1.3 Hz, 1H), 7.49 – 7.42 (m, 3H), 7.13 (dd, J = 5.4, 3.6 Hz, 1H), 6.13 (d, J = 6.9 Hz, 1H), 5.58 (dd, J = 8.8, 3.7 Hz, 1H), 5.42 (d, J = 6.3 Hz, 1H), 5.22 (d, J = 4.5 Hz, 1H), 5.11 – 4.98 (m, 1H), 4.25 – 4.15 (m, 1H), 4.04 – 3.95 (m, 1H), 3.74 – 3.64 (m, 1H), 3.60 – 3.51 (m, 1H). ^{13}C NMR (DMSO-d_6) δ 155.47, 152.46, 149.89, 145.68, 135.88, 132.70, 128.13, 126.28, 119.57, 89.39, 86.73, 70.96, 62.15, 56.00. LC-MS (*m/z*): positive mode 382.1 [M + H]$^+$. Purity by HPLC-UV (254 nm)-ESI-MS: 99.4%.

(2*R*,3*R*,4*S*,5*R*)-2-(6-Amino-8-(phenylthio)-9*H*-purin-9-yl)-5-(hydroxymethyl)tetrahydrofuran-3,4-diol (30m)

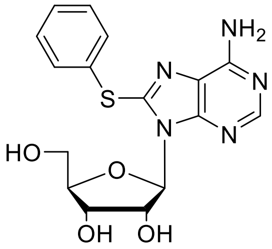

The compound was synthesized using **23** (300 mg 0.87 mmol), thiophenol (0.27 mL, 2.61 mmol) and afforded a yellowish solid (120 mg, 37%), mp: 115.0-117.0 °C. ^{1}H NMR (DMSO-d_6) δ 8.14 (d, J = 1.0 Hz, 1H), 7.53 (s, 2H), 7.43 – 7.35 (m, 4H), 7.35 – 7.31 (m, 1H), 6.10 (d, J = 6.9 Hz, 1H), 5.60 (dd, J = 8.9, 3.6 Hz, 1H), 5.36 (dd, J = 6.3, 1.0 Hz, 1H), 5.18 – 5.12 (m, 1H), 5.06 (q, J = 6.2 Hz, 1H), 4.23 – 4.17 (m, 1H), 3.96 (d, J = 3.0 Hz, 1H), 3.75 – 3.65 (m, 1H), 3.58 – 3.49 (m, 1H). ^{13}C NMR (DMSO-d_6) δ 155.65, 152.62, 149.82, 144.29, 132.03, 129.81, 129.57, 127.81, 119.99, 89.56, 86.67, 71.37, 71.01, 62.16. LC-MS (*m/z*): positive mode 376.2 [M + H]$^+$. Purity by HPLC-UV (254 nm)-ESI-MS: 99.7%.

(2*R*,3*R*,4*S*,5*R*)-2-(6-Amino-8-((4-fluorophenyl)thio)-9*H*-purin-9-yl)-5-(hydroxymethyl)tetrahydrofuran-3,4-diol (30n)

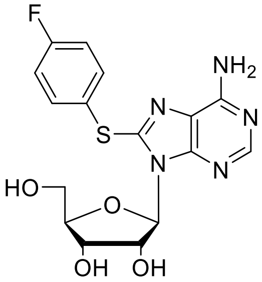

The compound was synthesized using **23** (300 mg 0.87 mmol), 4-fluorothiophenol (0.56 mL, 5.22 mmol) and afforded a yellowish solid (192 mg, 56%), mp: 107.0-109.0 °C. ^{1}H NMR (DMSO-d_6) δ 8.13 (s, 1H), 7.59 – 7.43 (m, 4H), 7.32 – 7.20 (m, 2H), 6.07 (d, J = 6.9 Hz, 1H), 5.57 (dd, J = 8.8, 3.8 Hz, 1H), 5.37 (d, J = 6.2 Hz, 1H), 5.16 (d, J = 4.4 Hz, 1H), 5.05 (q, J = 6.2 Hz, 1H), 4.25 – 4.15 (m, 1H), 4.04 – 3.92 (m, 1H), 3.69 (dt, J = 12.2, 3.8 Hz, 1H), 3.61 – 3.50 (m, 1H). ^{13}C NMR (126 MHz, DMSO) δ 161.01, 155.52, 152.51, 149.90, 144.89, 133.18, 133.11, 126.94, 119.90, 116.77, 116.59, 89.46, 86.67, 71.34, 70.97, 62.14. LC-MS (*m/z*): positive mode 394.2 [M + H]$^+$. Purity by HPLC-UV (254 nm)-ESI-MS: 99.9%.

(2*R*,3*R*,4*S*,5*R*)-2-(6-Amino-8-((4-aminophenyl)thio)-9*H*-purin-9-yl)-5-(hydroxymethyl)tetrahydrofuran-3,4-diol (30o)

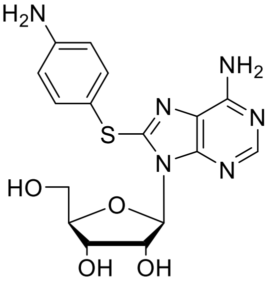

The compound was synthesized using **23** (300 mg 0.87 mmol), 4-aminothiophenol (653 mg, 5.22 mmol) and afforded a yellowish solid (184 mg, 54%), mp: 122.0-124.0 °C. ^{1}H NMR (DMSO-d_6) δ 8.07 (s, 1H), 7.30 (s, 2H), 7.26 – 7.20 (m, 2H), 6.60 – 6.53 (m, 2H), 6.09 (d, J = 6.7 Hz, 1H), 5.47 (s, 2H), 5.37 (d, J = 6.6 Hz, 1H), 5.17 (d, J = 4.3 Hz, 1H), 5.04 (q, J = 6.4 Hz, 1H), 4.32 (t, J = 5.1 Hz, 1H), 4.23 – 4.17 (m, 1H), 3.99 (d, J = 2.7 Hz, 1H), 3.69 (dt, J = 12.1, 3.7 Hz, 1H), 3.60 – 3.50 (m, 1H). ^{13}C NMR (DMSO-d_6) δ 155.09, 151.78, 150.03, 149.96, 148.15, 134.97, 119.61, 114.58, 112.52, 89.21, 86.55, 71.34, 71.01, 62.22. LC-MS (*m/z*): positive mode 391.2 [M + H]$^+$. Purity by HPLC-UV (254 nm)-ESI-MS: 95.5%.

(2*R*,3*R*,4*S*,5*R*)-2-(6-Amino-8-(benzylthio)-9*H*-purin-9-yl)-5-(hydroxymethyl)tetrahydrofuran-3,4-diol (30p), CAS: 121059-93-6

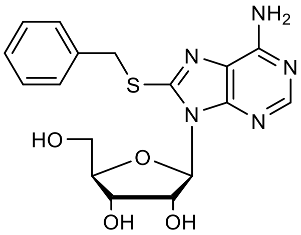

The compound was synthesized using **23** (400 mg, 1.16 mmol), phenylmethanethiol (0.41 mL, 3.48 mmol) and afforded a yellowish solid (314 mg, 70%), mp: 210.0-212.0 °C. ^{1}H NMR (DMSO-d_6) δ 8.06 (s, 1H), 7.51 – 7.47 (m, 2H), 7.35 – 7.29 (m, 4H), 7.29 – 7.24 (m, 1H), 5.74 (d, J = 6.8 Hz, 1H), 5.58 (dd, J = 8.8, 3.8 Hz, 1H), 5.38 (d, J = 6.5 Hz, 1H), 5.16 (d, J = 4.6 Hz, 1H), 5.04 – 4.92 (m, 1H), 4.58 (q, J = 13.1 Hz, 2H), 4.19 – 4.12 (m, 1H), 4.00 – 3.92 (m, 1H), 3.66 (dt, J = 12.2, 3.8 Hz, 1H), 3.56 – 3.47 (m, 1H). ^{13}C NMR (DMSO-d_6) δ 154.58, 151.36, 150.52, 148.08, 137.05, 129.14, 128.42, 127.43, 119.50, 88.86, 86.59, 71.32, 70.90, 62.15, 35.95. LC-MS (*m/z*): positive mode 390.3 [M + H]$^+$. Purity by HPLC-UV (254 nm)-ESI-MS: 99.9%.

(2*R*,3*R*,4*S*,5*R*)-2-(6-Amino-8-(phenethylthio)-9*H*-purin-9-yl)-5-(hydroxymethyl)tetrahydrofuran-3,4-diol (30q)

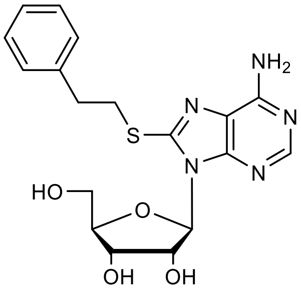

The compound was synthesized using **23** (400 mg, 1.16 mmol), 2-phenylethanethiol (0.47 mL, 3.48 mmol) and afforded a white solid (426 mg, 91%), mp: 176.0-177.5 °C. ^{1}H NMR (DMSO-d_6) δ 8.07 (s, 1H), 7.36 – 7.30 (m, 4H), 7.27 (s, 2H), 7.26 – 7.20 (m, 1H), 5.75 (d, J = 6.9 Hz, 1H), 5.60 (dd, J = 8.8, 3.7 Hz, 1H), 5.37 (d, J = 6.4 Hz, 1H), 5.15 (d, J = 4.4 Hz, 1H), 5.04 – 4.97 (m, 1H), 4.21 – 4.12 (m, 1H), 4.00 – 3.94 (m, 1H), 3.67 (dt, J = 12.2, 3.8 Hz, 1H), 3.62 – 3.47 (m, 3H), 3.03 (t, J = 7.6 Hz, 2H). ^{13}C NMR (DMSO-d_6) δ 154.53, 151.25, 150.44, 148.52, 139.72, 128.65, 128.35, 126.38, 119.65, 88.84, 86.58, 71.25, 70.94, 62.19, 34.84, 33.50. LC-MS (*m/z*): positive mode 404.3 [M + H]$^+$. Purity by HPLC-UV (254 nm)-ESI-MS: 99.9%.

(2*R*,3*R*,4*S*,5*R*)-2-(6-Amino-8-(naphthalen-1-ylthio)-9*H*-purin-9-yl)-5-(hydroxymethyl)tetrahydrofuran-3,4-diol (30r)

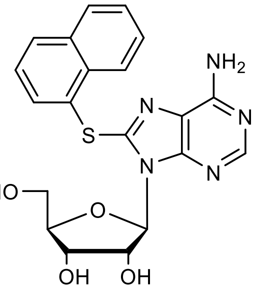

The compound was synthesized using **23** (400 mg, 1.16 mmol), 1-naphthalenethiol (0.49 mL, 3.48 mmol) and afforded a yellowish solid (161 mg, 33%), mp: 134.0-136.0 °C. ^{1}H NMR (DMSO-d_6) δ 8.31 – 8.25 (m, 1H), 8.12 (s, 1H), 8.04 – 7.99 (m, 2H), 7.67 – 7.59 (m, 3H), 7.53 (dd, J = 8.2, 7.3 Hz, 1H), 7.37 (s, 2H), 6.18 (d, J = 6.9 Hz, 1H), 5.63 (dd, J = 9.0, 3.7 Hz, 1H), 5.46 (d, J = 6.4 Hz, 1H), 5.19 (d, J = 4.4 Hz, 1H), 5.14 – 5.09 (m, 1H), 4.24 – 4.18 (m, 1H), 4.04 – 3.97 (m, 1H), 3.76 – 3.67 (m, 1H), 3.60 – 3.52 (m, 1H). ^{13}C NMR (DMSO-d_6) δ 155.28, 152.30, 150.06, 145.06, 133.81, 132.01, 131.44, 129.48, 128.77, 127.76, 127.44, 126.73, 126.18, 124.28, 119.99, 89.56, 86.78, 71.43, 71.05, 62.19. LC-MS (*m/z*): positive mode 426.4 $[M + H]^+$. Purity by HPLC-UV (254 nm)-ESI-MS: 96.1%.

(2*R*,3*R*,4*S*,5*R*)-2-(6-Amino-8-methyl-9*H*-purin-9-yl)-5-(hydroxymethyl)tetrahydrofuran-3,4-diol (32a), CAS: 56973-12-7

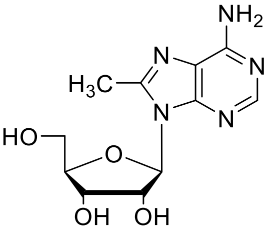

8-Bromoadenosine (**23**, 500 mg, 1.44 mmol, 1 equiv.) was dissolved in hexamethyldisilazane (10 mL) and dry dioxane (20 mL) in a three-necked flask. A catalytic amount of $(NH_4)_2SO_4$ (50 mg) was added to the suspension and the mixture was refluxed at 125 °C for 3 h. Then the mixture was dried *in vacuum* and redissolved in dry THF under argon without purification. Triphenylphosphine (39 mg, 0.14 mmol, 0.1 equiv.), $PdCl_2$ (13 mg, 0.07 mmol, 0.05 equiv.) and 2 M trimethylaluminum in toluene (1.45 mL, 2.89 mmol, 2 equiv.) were subsequently added. The mixture was refluxed under argon for 2.5 h and dried *in vacuum* to yield a green residue. The residue was dissolved in MeOH (50 mL) and refluxed for 4 h with a small amount of NH_4Cl (50 mg) for the deprotection of 2′-, 3′- and 5′-trimethylsiyl groups. The reaction progress was monitored by TLC (MeOH/DCM, 1:9). After the reaction was completed, cooled to rt, 5 g silica gel was added, and the solvent was evaporated *in vacuum*. The crude compound was purified by silica gel column chromatography (20% MeOH in DCM) yielding a milk white solid (124 mg, 31%), mp: 204.5-206.3 °C (*lit.*[25] 208 °C). ^{1}H NMR (DMSO-d_6) δ 8.05 (s, 1H), 7.21 (s, 2H), 5.85 (dd, J = 9.2, 3.4 Hz, 1H), 5.78 (d, J = 7.1 Hz, 1H), 5.35 (d, J = 6.9 Hz, 1H), 5.19 (d, J = 4.5 Hz, 1H), 4.84 (td, J = 7.0, 5.2 Hz, 1H), 4.15 (td, J = 4.7, 2.1 Hz, 1H), 3.99 (td,

J = 3.3, 2.0 Hz, 1H), 3.68 (dt, *J* = 12.3, 3.4 Hz, 1H), 3.54 (ddd, *J* = 12.4, 9.2, 3.4 Hz, 1H), 2.55 (s, 3H). ^{13}C NMR (DMSO-d_6) δ 155.39, 151.27, 149.64, 148.94, 118.10, 88.52, 86.57, 72.03, 70.93, 62.15, 14.31. LC-MS (*m/z*): positive mode 282.2 $[M + H]^+$. Purity by HPLC-UV (254 nm)-ESI-MS: 98.6%.

(2*R*,3*R*,4*S*,5*R*)-2-(8-Allyl-6-amino-9*H*-purin-9-yl)-5-(hydroxymethyl)tetrahydrofuran-3,4-diol (32b)

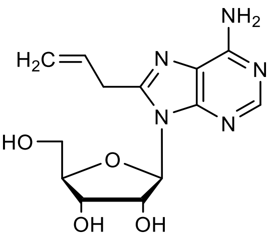

Compound **23** (1.00 g, 2.89 mmol, 1 equiv.) was dissolved in hexamethyldisilazane (12 mL) in a three-necked flask. A catalytic amount of $(NH_4)_2SO_4$ (100 mg) was added to the suspension and the mixture was refluxed at 125 °C for 3 h. Then the mixture was dried *in vacuum*. In NMP (*N*-methyl-2-pyrollidone, 10 mL) under argon, PPh_3 (78 mg, 0.29 mmol, 0.1 equiv.), $PdCl_2$ (27 mg, 0.15 mmol, 0.05 equiv.) and allyltributyltin (1.79 mL, 5.78 mml, 2 equiv.) were added. The reaction was refluxed under argon at 125 °C for 2.5 h, then the crude mixture was dried *in vacuum*. The residue was dissolved in MeOH (50 mL) and refluxed for 4 h with a small amount of NH_4Cl (100 mg). The reaction progress was monitored by TLC (MeOH/DCM, 1:9). After the reaction was completed, cooled to rt, 10 g silica gel was added, and the solvent was evaporated *in vacuum*. The crude compound was purified by silica gel column chromatography (8% MeOH in DCM) and afforded a yellowish solid (557 mg, 63%), mp: 110-112 °C. ^{1}H NMR (600 MHz, DMSO-d_6) δ 8.07 (s, 1H), 7.28 (d, *J* = 8.8 Hz, 2H), 6.12 – 6.04 (m, 1H), 5.95 – 5.89 (m, 1H), 5.77 (d, *J* = 7.1 Hz, 1H), 5.34 (d, *J* = 7.1 Hz, 1H), 5.23 – 5.12 (m, 3H), 4.90 – 4.85 (m, 1H), 4.19 – 4.13 (m, 1H), 4.01 – 3.97 (m, 1H), 3.77 – 3.65 (m, 3H), 3.57 – 3.50 (m, 1H). ^{13}C NMR (151 MHz, DMSO-d_6) δ 155.63, 151.44, 150.10, 149.56, 133.06, 118.36, 117.62, 88.54, 86.74, 72.08, 71.00, 62.23, 31.91. LC-MS (*m/z*): positive mode 308.1 $[M + H]^+$. Purity by HPLC-UV (254 nm)-ESI-MS: 96.5%.

(2*R*,3*R*,4*S*,5*R*)-2-(6-Amino-8-propyl-9*H*-purin-9-yl)-5-(hydroxymethyl)tetrahydrofuran-3,4-diol (32c), CAS: 101904-46-5

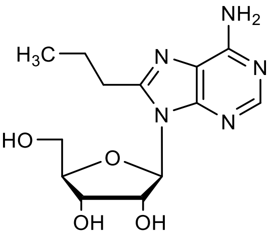

The compound was synthesized using **32b** (200 mg, 0.65 mmol**)** and afforded a white solid (167 mg, 83%). LC-MS (*m/z*): positive mode 310.1 $[M + H]^+$. Purity by HPLC-UV (254 nm)-ESI-MS: 90.1%.

(2*R*,3*R*,4*S*,5*R*)-2-(6-Amino-8-(pent-1-yn-1-yl)-9*H*-purin-9-yl)-5-(hydroxymethyl)tetrahydrofuran-3,4-diol (32d)

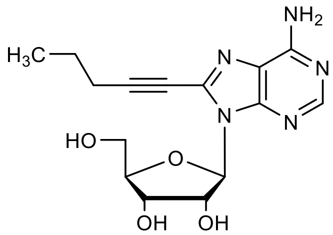

The compound was synthesized using 1-pentyne (0.71 mL, 7.20 mmol) and afforded a yellowish solid (79 mg, 16%). ^{1}H NMR (DMSO-d_6) δ 8.14 (s, 1H), 7.54 (s, 2H), 5.94 (d, *J* = 6.7 Hz, 1H), 5.52 (dd, *J* = 8.7, 3.9 Hz, 1H), 5.39 (d, *J* = 6.2 Hz, 1H), 5.16 (d, *J* = 4.3 Hz, 1H), 5.07 – 4.92 (m, 1H), 4.23 – 4.16 (m, 1H), 4.01 – 3.93 (m, 1H), 3.72 – 3.63 (m, 1H), 3.56 – 3.48 (m, 1H), 2.55 (t, *J* = 6.9 Hz, 2H), 1.62 (h, *J* = 7.2 Hz, 2H), 1.03 (t, *J* = 7.4 Hz, 3H). ^{13}C NMR (DMSO-d_6) δ 155.93, 152.98, 148.31, 134.02, 119.08, 97.35, 89.28, 86.51, 71.46, 70.99, 70.32, 62.21, 21.05, 20.47, 13.25. LC-MS (*m/z*): positive mode 334.1 $[M + H]^+$. Purity by HPLC-UV (254 nm)-ESI-MS: 98.4%.

(2*R*,3*R*,4*S*,5*R*)-2-(6-Amino-8-(hex-1-yn-1-yl)-9*H*-purin-9-yl)-5-(hydroxymethyl)tetrahydrofuran-3,4-diol (32e)

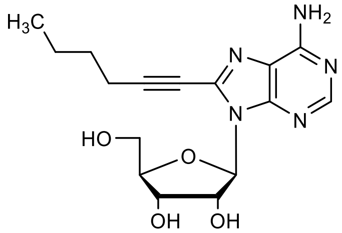

The compound was synthesized using 1-hexyne (0.50 mL, 4.35 mmol) and afforded a yellowish solid (50 mg, 17%), mp: 192.7-194.7 °C. ^{1}H NMR (DMSO-d_6) δ 8.14 (s, 1H), 7.53 (s, 2H), 5.93 (d, *J* = 6.7 Hz, 1H), 5.52 (dd, *J* = 8.7, 3.9 Hz, 1H), 5.38 (d, *J* = 6.3 Hz, 1H), 5.15 (d, *J* = 4.3 Hz, 1H), 5.00 (q, *J* = 6.1 Hz, 1H), 4.27 – 4.14 (m, 1H), 4.05 – 3.93 (m, 1H), 3.72 – 3.63 (m, 1H), 3.58 – 3.48 (m, 1H), 2.58 (t, *J* = 7.0 Hz, 2H), 1.69 – 1.53 (m, 2H), 1.53 – 1.40 (m, 2H), 0.93 (t, *J* = 7.3 Hz, 3H). ^{13}C NMR (DMSO-d_6) δ 155.92, 152.98, 148.31, 134.02, 119.08, 97.46, 89.27, 86.51, 71.44, 70.98, 70.20, 62.20, 29.49, 21.36, 18.24, 13.36. LC-MS (*m/z*): positive mode 348.0 $[M + H]^+$. Purity by HPLC-UV (254 nm)-ESI-MS: 99.0%.

(2*R*,3*R*,4*S*,5*R*)-2-(6-Amino-8-pentyl-9*H*-purin-9-yl)-5-(hydroxymethyl)tetrahydrofuran-3,4-diol (32f)

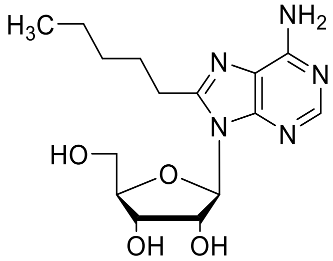

The compound was synthesized using **32d** (66 mg, 0.20 mmol) and afforded a yellowish solid (63 mg, 95%), mp: 75.0-77.0 °C. ^{1}H NMR (DMSO-d_6) δ 8.05 (s, 1H), 7.25 (s, 2H), 5.91 (d, J = 8.5 Hz, 1H), 5.76 (d, J = 7.2 Hz, 1H), 5.35 (d, J = 7.0 Hz, 1H), 5.24 – 5.13 (m, 1H), 4.91 (q, J = 6.2 Hz, 1H), 4.15 (d, J = 5.2 Hz, 1H), 4.00 (q, J = 3.1 Hz, 1H), 3.68 (dd, J = 12.3, 3.3 Hz, 1H), 3.54 (d, J = 12.2 Hz, 1H), 2.86 (p, J = 7.4 Hz, 2H), 1.83 – 1.70 (m, 2H), 1.35 (dddd, J = 15.7, 10.3, 8.3, 4.1 Hz, 4H), 0.88 (t, J = 7.0 Hz, 3H). ^{13}C NMR (DMSO-d_6) δ 155.45, 152.47, 151.13, 149.52, 118.27, 88.37, 86.75, 71.80, 71.09, 62.28, 30.85, 27.23, 27.14, 21.79, 13.83. LC-MS (*m/z*): positive mode 338.0 $[M + H]^+$. Purity by HPLC-UV (254 nm)-ESI-MS: 98.0%.

(2*R*,3*R*,4*S*,5*R*)-2-(6-Amino-8-phenyl-9*H*-purin-9-yl)-5-(hydroxymethyl)tetrahydrofuran-3,4-diol (32g), CAS: 73340-78-0

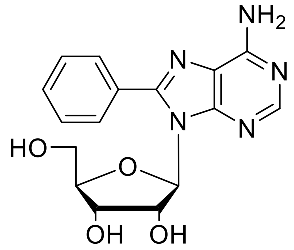

To a solution of 8-bromoadenosine (**23**, 200 mg, 0.58 mmol, 1 equiv.) in dioxane/H_2O (2:1, 9 mL), benzeneboronic acid (106 mg, 0.87 mmol, 1.5 equiv.), $Pd(PPh_3)_2Cl_2$ (41 mg, 0.06 mmol, 0.1 equiv.) and K_2CO_3 (240 mg, 1.74 mmol, 3 equiv.) were added. The mixture was stirred at 90 °C under argon for 2 h, and monitored by TLC (MeOH/DCM, 1:9). After the reaction was completed, cooled to rt, 5 g silica gel was added, and the solvent was evaporated in vacuum. The crude mixture was purified by silica gel column chromatography (12% MeOH in DCM) yielding a yellow solid (189 mg, 95%), mp: 156.0-158.0 °C (*lit.*[26] 142-143 °C). ^{1}H NMR (DMSO-d_6) δ 8.16 (s, 1H), 7.76 (dd, J = 6.7, 2.9 Hz, 2H), 7.59 (p, J = 3.6, 3.1 Hz, 3H), 7.48 (s, 2H), 5.87 – 5.71 (m, 2H), 5.46 (d, J = 6.4 Hz, 1H), 5.29 – 5.07 (m, 2H), 4.17 (t, J = 4.7 Hz, 1H), 3.94 (q, J = 3.3 Hz, 1H), 3.70 (dt, J = 12.1, 3.5 Hz, 1H), 3.55 (ddd, J = 12.5, 9.2, 3.7 Hz, 1H). ^{13}C NMR (DMSO-d_6) δ 156.24, 152.03, 150.98, 149.83, 130.14, 129.68, 129.42, 128.76, 119.14, 89.13, 86.73, 71.25, 71.11, 62.33. LC-MS (*m/z*): positive mode 344.1 $[M + H]^+$. Purity by HPLC-UV (254 nm)-ESI-MS: 98.8%.

(2*R*,3*R*,4*R*,5*R*)-2-(Acetoxymethyl)-5-(6-amino-9*H*-purin-9-yl)tetrahydrofuran-3,4-diyl diacetate (34), CAS: 7387-57-7

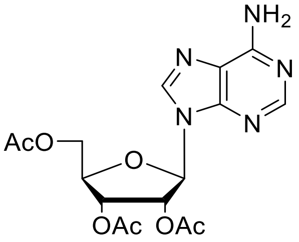

To a solution of adenosine (**4**, 1.00 g, 3.74 mmol, 1 equiv.) in MeCN (10 mL), Et_3N (2.34 mL, 16.83 mmol, 4.5 equiv.), DMAP (68 mg, 0.56 mmol, 0.15 equiv.) and acetic anhydride (1.24 mL, 13.09 mmol, 3.5 equiv.) were added at 0 °C. The mixture was stirred at rt for 30 min and monitored by TLC (MeOH/DCM, 1:9). After the reaction was completed, the solvent volume was reduced *in vacuum*. Then 5 mL EtOH was added, and the crude mixture was stirred at rt for 1 h. The precipitate was filtrated and washed by EtOH (5 mL), and dried in oven at 60 °C overnight yielding a white powder (1.10 g, 75%), mp: 176.0-178.0 °C (*lit.*[27] 173-174 °C). ^{1}H NMR (DMSO-d_6) δ 8.34 (s, 1H), 8.17 (s, 1H), 7.34 (s, 2H), 6.20 (d, J = 5.3 Hz, 1H), 6.03 (t, J = 5.7 Hz, 1H), 5.63 (t, J = 5.4 Hz, 1H), 4.49 – 4.32 (m, 2H), 4.24 (dd, J = 11.8, 5.5 Hz, 1H), 2.12 (s, 3H), 2.02 (d, J = 14.6 Hz, 6H). ^{13}C NMR (DMSO-d_6) δ 169.97, 169.40, 169.22, 156.15, 152.81, 149.07, 139.99, 119.17, 85.56, 79.36, 71.85, 70.05, 62.77, 20.42, 20.32, 20.15. LC-MS (*m/z*): positive mode 394.3 $[M + H]^+$. Purity by HPLC-UV (254 nm)-ESI-MS: 99.9%.

(2*R*,3*R*,4*S*,5*R*)-2-(6-Amino-8-cyclohexyl-9*H*-purin-9-yl)-5-(hydroxymethyl)tetrahydrofuran-3,4-diol (35)

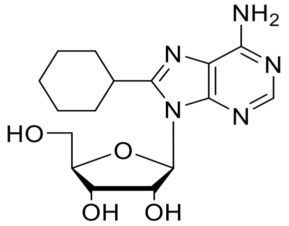

To a solution of compound **34** (300 mg, 0.76 mmol, 1 equiv.) in cyclohexane (10 mL), di-*tert*-butyl peroxide (0.28 mL, 1.52 mmol, 2 equiv.) was added. The mixture was stirred in an autoclave at 140 °C for 24 h and monitored by TLC (MeOH/DCM, 1:9). After the reaction was completed, the solvent volume was reduced *in vacuum*. Then the 2'-, 3'- and 5'-*O*-acetyl groups were removed by stirring the mixture in a solution of 7 N NH_3 in MeOH (10 mL) at rt overnight. After the reaction was completed, 5 g silica gel was added, and the solvent was evaporated *in vacuum*. The crude compound was purified by silica gel column chromatography (8% MeOH in DCM) yielding a brownish solid (130 mg, 49%), mp: 86.0-88.0 °C. ^{1}H NMR (DMSO-d_6) δ 8.05 (d, J = 1.2 Hz, 1H), 7.17 (s, 2H), 5.93 – 5.84 (m, 1H), 5.79 (d, J = 7.2 Hz, 1H), 5.35 (dd, J = 6.9, 1.2 Hz, 1H), 5.18 (dd, J = 4.4, 1.2 Hz, 1H), 5.04 – 4.93 (m, 1H), 4.20 – 4.13 (m, 1H), 4.03 – 3.97 (m, 1H), 3.73 – 3.64 (m, 1H), 3.61 – 3.50 (m, 1H), 3.02 – 2.93 (m, 1H), 1.85 – 1.77

(m, 2H), 1.75 (d, J = 1.1 Hz, 2H), 1.67 – 1.58 (m, 2H), 1.47 – 1.37 (m, 2H), 1.30 – 1.19 (m, 2H). ^{13}C NMR (DMSO-d_6) δ 156.12, 155.54, 151.11, 149.41, 118.29, 87.99, 86.74, 71.56, 71.09, 62.31, 35.34, 31.67, 31.39, 25.52, 25.48, 22.44. LC-MS (*m/z*): positive mode 350.30 [M + H]$^+$. Purity by HPLC-UV (254 nm)-ESI-MS: 98.0%.

(2*R*,3*R*,4*S*,5*R*)-2-(6-Bromo-8-(cyclohexylthio)-9*H*-purin-9-yl)-5-(hydroxymethyl)tetrahydrofuran-3,4-diol (38a)

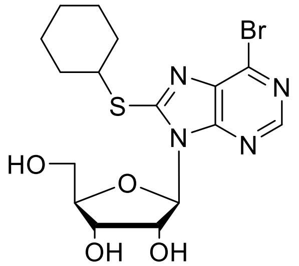

The compound was synthesized using **30j** (163 mg, 0.42 mmol) and afforded a white solid (70 mg, 30%), mp: 77-79 °C. ^{1}H NMR (500 MHz, DMSO-d_6) δ 8.61 (s, 1H), 5.78 (d, J = 6.2 Hz, 1H), 5.44 (d, J = 6.0 Hz, 1H), 5.23 (d, J = 5.0 Hz, 1H), 5.09 (q, J = 5.9 Hz, 1H), 4.85 (dd, J = 6.7, 5.2 Hz, 1H), 4.26 – 4.21 (m, 1H), 4.14 – 4.06 (m, 1H), 3.97 – 3.90 (m, 1H), 3.74 – 3.64 (m, 1H), 3.58 – 3.49 (m, 1H), 2.22 – 2.10 (m, 2H), 1.78 – 1.55 (m, 5H), 1.55 – 1.42 (m, 2H), 1.41 – 1.31 (m, 1H). ^{13}C NMR (126 MHz, DMSO-d_6) δ 157.08, 151.81, 150.22, 138.42, 134.33, 89.06, 86.14, 70.72, 70.32, 61.65, 45.93, 32.39, 32.26, 25.03, 24.97. LC-MS (*m/z*): negative mode 445.10 [M - H]$^-$. Purity by HPLC-UV (254 nm)-ESI-MS: 97.3%.

(2*R*,3*R*,4*S*,5*R*)-2-(6-Bromo-8-(naphthalen-1-ylthio)-9*H*-purin-9-yl)-5-(hydroxymethyl)tetrahydrofuran-3,4-diol (38b)

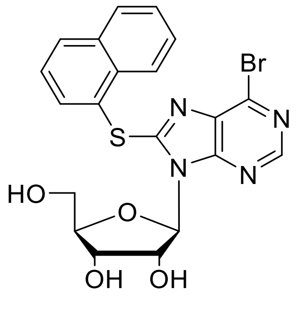

The compound was synthesized using **30r** (224 mg, 0.53 mmol) and afforded a yellowish oil (140 mg, 54%). LC-MS (*m/z*): negative mode 489.10 [M - H]$^-$. Purity by HPLC-UV (254 nm)-ESI-MS: 83.1%.

(2*R*,3*R*,4*S*,5*R*)-2-(6-Bromo-8-(butylthio)-9*H*-purin-9-yl)-5-(hydroxymethyl)tetrahydrofuran-3,4-diol (38c)

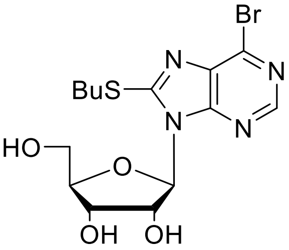

The compound was synthesized using **37** (700 mg, 1.41 mmol) and afforded a yellowish solid (540 mg, 91%), mp: 63.0-65.0 °C. ^{1}H NMR (500 MHz, DMSO-*d*$_6$) *δ* 8.61 (s, 1H), 5.79 (d, *J* = 6.1 Hz, 1H), 5.45 (d, *J* = 6.0 Hz, 1H), 5.24 (d, *J* = 4.8 Hz, 1H), 5.07 (q, *J* = 5.7 Hz, 1H), 4.86 (s, 1H), 4.23 (d, *J* = 4.5 Hz, 1H), 3.99 – 3.91 (m, 1H), 3.72 – 3.64 (m, 1H), 3.59 – 3.52 (m, 1H), 3.50 – 3.38 (m, 2H), 1.77 (p, *J* = 7.2 Hz, 2H), 1.46 (h, *J* = 7.4 Hz, 2H), 0.94 (t, *J* = 7.4 Hz, 3H). ^{13}C NMR (126 MHz, DMSO-*d*$_6$) *δ* 157.87, 152.11, 150.18, 138.27, 134.23, 89.01, 86.17, 70.75, 70.31, 61.65, 31.41, 30.46, 21.13, 13.34. LC-MS (*m/z*): positive mode 421.10 [M + H]$^+$. Purity by HPLC-UV (254 nm)-ESI-MS: 99.5%.

(2*R*,3*R*,4*R*,5*R*)-2-(Acetoxymethyl)-5-(6-amino-8-(methylamino)-9*H*-purin-9-yl)tetrahydrofuran-3,4-diyl diacetate (39a)

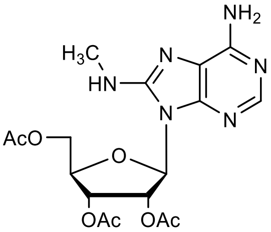

The compound was synthesized using **24a** (1.20 g, 4.05 mmol), acetic anhydride (1.15 mL, 12.15 mmol) and afforded a yellowish oil (1.21 g, 71%). ^{1}H NMR (DMSO-*d*$_6$) *δ* 7.92 (s, 1H), 6.91 (q, *J* = 4.6 Hz, 1H), 6.56 (s, 2H), 6.32 (dd, *J* = 6.3, 4.8 Hz, 1H), 5.98 (d, *J* = 4.7 Hz, 1H), 5.69 (t, *J* = 6.1 Hz, 1H), 4.38 (dd, *J* = 12.0, 3.5 Hz, 1H), 4.26 – 4.21 (m, 1H), 4.16 (dd, *J* = 12.0, 5.7 Hz, 1H), 2.90 (d, *J* = 4.6 Hz, 3H), 2.10 (s, 3H), 2.05 (s, 3H), 1.94 (s, 3H). ^{13}C NMR (DMSO-*d*$_6$) *δ* 171.96, 169.96, 169.38, 152.58, 151.95, 149.46, 148.84, 117.44, 84.62, 78.70, 70.57, 69.71, 62.67, 29.12, 21.03, 20.36, 20.25. LC-MS (*m/z*): positive mode 423.0 [M + H]$^+$. Purity by HPLC-UV (254 nm)-ESI-MS: 96.4%.

(2*R*,3*R*,4*R*,5*R*)-2-(Acetoxymethyl)-5-(6-amino-8-(butylthio)-9*H*-purin-9-yl)tetrahydrofuran-3,4-diyl diacetate (39b)

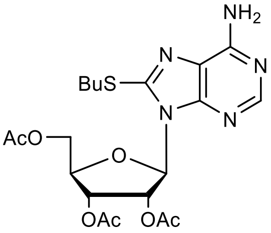

The compound was synthesized using **37** (700 mg, 1.97 mmol), acetic anhydride (0.56 mL, 5.91 mmol) and afforded a yellowish oil (717 mg, 76%). ^{1}H NMR (DMSO-*d*$_6$) δ 8.11 (s, 1H), 7.25 (s, 2H), 6.24 (dd, *J* = 6.2, 4.9 Hz, 1H), 6.02 (d, *J* = 4.9 Hz, 1H), 5.69 (t, *J* = 6.0 Hz, 1H), 4.41 (dd, *J* = 12.1, 3.5 Hz, 1H), 4.38 – 4.33 (m, 1H), 4.18 (dd, *J* = 12.1, 5.5 Hz, 1H), 3.28 – 3.24 (m, 2H), 2.04 (s, 3H), 1.96 (s, 3H), 1.91 (s, 3H), 1.67 (p, *J* = 7.3 Hz, 2H), 1.46 – 1.36 (m, 2H), 0.90 (t, *J* = 7.4 Hz, 3H). ^{13}C NMR (DMSO-*d*$_6$) δ 171.95, 169.92, 169.30, 154.45, 152.00, 150.69, 147.90, 119.12, 86.16, 79.12, 70.81, 69.67, 62.38, 32.31, 30.84, 21.14, 20.39, 20.31, 20.17, 13.38. LC-MS (*m/z*): positive mode 482.1 [M + H]$^+$. Purity by HPLC-UV (254 nm)-ESI-MS: 96.0%.

(2*R*,3*R*,4*R*,5*R*)-2-(Acetoxymethyl)-5-(6-bromo-8-(methylamino)-9*H*-purin-9-yl)tetrahydrofuran-3,4-diyl diacetate (40a)

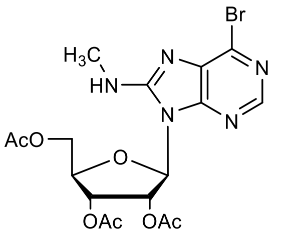

The compound was synthesized using **39a** (1.50 g, 3.55 mmol), BTEA-Br (1.45 g, 5.33 mmol) and afforded a yellow solid (0.82 g, 47%), mp: 53.8-55.8 °C. ^{1}H NMR (DMSO-*d*$_6$) δ 8.86 (s, 1H), 6.23 (d, *J* = 3.6 Hz, 1H), 6.12 (dd, *J* = 6.3, 3.5 Hz, 1H), 5.79 (t, *J* = 6.7 Hz, 1H), 5.75 (s, 1H), 4.42 (dd, *J* = 12.1, 3.3 Hz, 1H), 4.37 – 4.32 (m, 1H), 4.26 (dd, *J* = 12.0, 5.8 Hz, 1H), 3.55 (s, 3H), 2.07 (s, 3H), 2.03 (s, 3H), 1.95 (s, 3H). ^{13}C NMR (DMSO-*d*$_6$) δ 169.95, 169.25, 151.78, 150.76, 149.82, 132.08, 88.80, 79.00, 72.10, 69.18, 62.28, 40.06, 32.72, 20.37, 20.25, 20.17. LC-MS (*m/z*): negative mode 485.0 [M - H]$^-$. Purity by HPLC-UV (254 nm)-ESI-MS: 96.4%.

(2*R*,3*R*,4*R*,5*R*)-2-(Acetoxymethyl)-5-(6-bromo-8-(butylthio)-9*H*-purin-9-yl)tetrahydrofuran-3,4-diyl diacetate (40b)

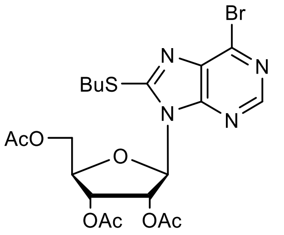

The compound was synthesized using **39b** (717 mg, 1.49 mmol), BTEA-Br (610 mg, 2.24 mmol) and afforded a yellowish oil (413 mg, 51%). ^{1}H NMR (DMSO-*d*$_6$) δ 8.66 (s, 1H), 6.15 (dd, *J* = 6.3, 4.7 Hz, 1H), 6.06 (d, *J* = 4.7 Hz, 1H), 5.68 (t, *J* = 6.1 Hz, 1H), 4.46 – 4.38 (m, 2H), 4.24 – 4.18 (m, 1H), 3.50 – 3.39 (m, 2H), 2.12 (s, 3H), 2.04 (s, 3H), 1.94 (s, 3H), 1.80 – 1.73 (m, 2H), 1.50 – 1.41 (m, 2H), 0.93 (t, *J* = 7.4 Hz, 3H). ^{13}C NMR (DMSO-*d*$_6$) δ 169.88, 169.38,

169.29, 157.11, 151.89, 150.57, 138.44, 134.05, 86.48, 79.38, 70.93, 69.37, 62.23, 31.64, 30.42, 21.10, 20.36, 20.29, 20.16, 13.35. LC-MS (*m/z*): negative mode 544.9 $[M - H]^-$. Purity by HPLC-UV (254 nm)-ESI-MS: 92.9%.

(2*R*,3*R*,4*S*,5*R*)-2-(8-(Cyclohexylthio)-6-((4-phenylbutyl)amino)-9*H*-purin-9-yl)-5-(hydroxymethyl)tetrahydrofuran-3,4-diol (41a)

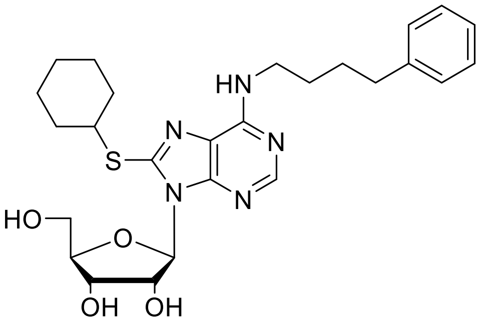

The compound was synthesized using **38a** (65 mg, 0.15 mmol), 4-phenylbutylamine (0.04 mL, 0.23 mmol) and afforded a brownish solid (69 mg, 90%), mp: 58-60 °C. ^{1}H NMR (500 MHz, DMSO-d_6) δ 8.22 – 7.95 (m, 1H), 7.82 (s, 1H), 7.25 (t, *J* = 7.5 Hz, 2H), 7.21 – 7.13 (m, 3H), 5.86 (s, 1H), 5.63 (dd, *J* = 9.1, 3.6 Hz, 1H), 5.33 (d, *J* = 6.4 Hz, 1H), 5.14 (d, *J* = 4.3 Hz, 1H), 5.00 (q, *J* = 6.5 Hz, 1H), 4.21 – 4.14 (m, 1H), 3.96 (s, 1H), 3.81 – 3.71 (m, 1H), 3.72 – 3.63 (m, 1H), 3.59 – 3.42 (m, 2H), 2.60 (d, *J* = 7.0 Hz, 3H), 2.02 (d, *J* = 29.2 Hz, 2H), 1.76 – 1.67 (m, 2H), 1.64 – 1.60 (m, 3H), 1.58 – 1.46 (m, 3H), 1.43 – 1.20 (m, 4H). ^{13}C NMR (126 MHz, DMSO-d_6) δ 153.37, 151.49, 146.95, 142.12, 128.24, 128.14, 125.57, 88.96, 86.58, 71.30, 71.02, 62.23, 46.73, 34.84, 32.92, 32.54, 28.70, 28.38, 25.27, 25.16, 24.98. LC-MS (*m/z*): positive mode 514.50 $[M + H]^+$. Purity by HPLC-UV (254 nm)-ESI-MS: 96.8%.

(2*R*,3*S*,4*R*,5*R*)-2-(Hydroxymethyl)-5-(8-(naphthalen-1-ylthio)-6-((4-phenylbutyl)amino)-9*H*-purin-9-yl)tetrahydrofuran-3,4-diol (41b)

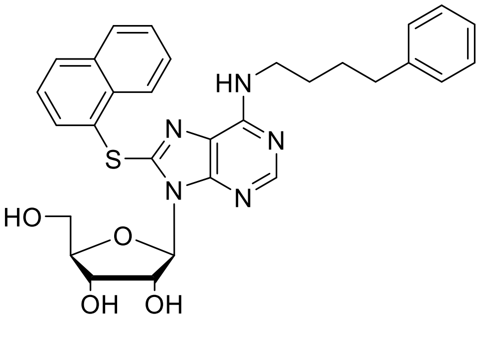

The compound was synthesized using **38b** (116 mg, 0.24 mmol), 4-phenylbutylamine (0.06 mL, 0.36 mmol) and afforded a brownish solid (55 mg, 41%), mp: 74-75 °C. ^{1}H NMR (500 MHz, DMSO-d_6) δ 8.29 – 8.23 (m, 1H), 8.19 (s, 1H), 8.05 – 7.95 (m, 2H), 7.89 (s, 1H), 7.62 (s, 3H), 7.51 (t, *J* = 7.7 Hz, 1H), 7.23 (t, *J* = 7.5 Hz, 2H), 7.14 (t, *J* = 7.2 Hz, 3H), 6.17 (s, 1H), 5.61 (dd,

J = 9.0, 3.6 Hz, 1H), 5.43 (d, *J* = 6.4 Hz, 1H), 5.18 (d, *J* = 4.2 Hz, 1H), 5.11 (q, *J* = 6.2 Hz, 1H), 4.26 – 4.18 (m, 1H), 4.00 (s, 1H), 3.77 – 3.66 (m, 1H), 3.61 – 3.50 (m, 1H), 3.42 (s, 2H), 2.55 (s, 2H), 1.53 (s, 4H). ^{13}C NMR (126 MHz, DMSO-d_6) δ 153.70, 152.31, 149.22, 144.55, 142.08, 133.78, 131.91, 131.19, 129.36, 128.72, 128.21, 128.11, 127.95, 127.37, 126.68, 126.13, 125.53, 124.22, 120.41, 89.57, 86.77, 71.47, 71.02, 62.18, (NH*C*H_2 is overlapping with DMSO-d_6), 34.75, 28.50, 28.34. LC-MS (*m/z*): positive mode 558.30 [M + H]$^+$. Purity by HPLC-UV (254 nm)-ESI-MS: 93.8%.

(2*R*,3*R*,4*S*,5*R*)-2-(8-(Butylthio)-6-(methyl(4-phenylbutyl)amino)-9*H*-purin-9-yl)-5-(hydroxymethyl)tetrahydrofuran-3,4-diol (41c)

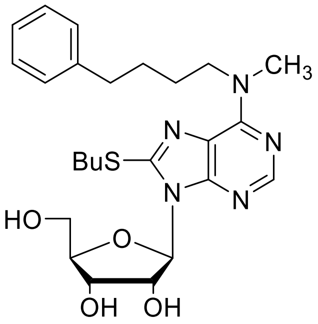

The compound was synthesized using **38c** (100 mg, 0.24 mmol), methyl(4-phenylbutyl)amine (0.07 mL, 0.36 mmol) and afforded a yellowish semi-solid (109 mg, 91%). ^{1}H NMR (600 MHz, DMSO-d_6) δ 8.12 (s, 1H), 7.25 (dd, *J* = 8.2, 7.0 Hz, 2H), 7.15 (t, *J* = 7.4 Hz, 3H), 5.74 (d, *J* = 6.8 Hz, 1H), 5.56 (dd, *J* = 8.7, 3.7 Hz, 1H), 5.37 (d, *J* = 6.4 Hz, 1H), 5.17 (d, *J* = 4.4 Hz, 1H), 5.04 – 4.95 (m, 1H), 4.19 – 4.13 (m, 1H), 4.01 – 3.92 (m, 1H), 3.85 – 3.59 (m, 2H), 3.57 – 3.38 (m, 2H), (C*H*$_3$ is overlapping with H_2O), 3.29 – 3.22 (m, 2H), 2.62 (t, *J* = 7.5 Hz, 2H), 1.73 – 1.56 (m, 6H), 1.39 (h, *J* = 7.4 Hz, 2H), 0.87 (t, *J* = 7.4 Hz, 3H). ^{13}C NMR (151 MHz, DMSO-d_6) δ 152.32, 151.31, 150.52, 147.57, 142.02, 128.21, 128.16, 125.65, 119.80, 88.78, 86.54, 71.08, 70.92, 62.20, 34.98, 31.60, 30.97, 28.23, 21.22, 13.37. LC-MS (*m/z*): positive mode 502.30 [M + H]$^+$. Purity by HPLC-UV (254 nm)-ESI-MS: 98.8%.

(2*R*,3*S*,4*R*,5*R*)-2-(Hydroxymethyl)-5-(8-(methylamino)-6-((4-phenylbutyl)amino)-9*H*-purin-9-yl)tetrahydrofuran-3,4-diol (41d)

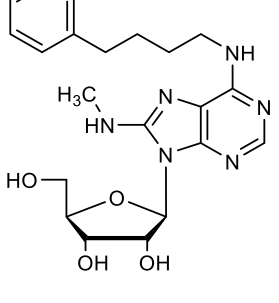

The compound was synthesized using **40a** (810 mg, 1.67 mmol), 4-phenylbutylamine (0.53 mL, 3.34 mmol) and afforded a brown viscous semi-solid (405 mg, 57%). LC-MS (*m/z*): positive mode 429.1 [M + H]$^+$. Purity by HPLC-UV (254 nm)-ESI-MS: 77.7%.

(2*R*,3*R*,4*S*,5*R*)-2-(8-(Butylthio)-6-((4-phenylbutyl)amino)-9*H*-purin-9-yl)-5-(hydroxymethyl)tetrahydrofuran-3,4-diol (41e)

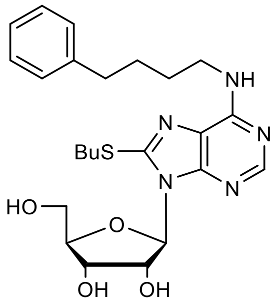

The compound was synthesized using **40b** (410 mg, 0.75 mmol), 4-phenylbutylamine (0.24 mL, 1.50 mmol) and afforded a yellowish solid (321 mg, 88%), mp: 62.0-64.0 °C. ^{1}H NMR (DMSO-d_6) δ 7.74 (s, 1H), 7.36 – 7.07 (m, 5H), 5.86 – 5.69 (m, 1H), 5.63 (dd, *J* = 8.9, 3.7 Hz, 1H), 5.37 (d, *J* = 6.4 Hz, 1H), 5.16 (d, *J* = 4.3 Hz, 1H), 5.06 – 4.90 (m, 1H), 4.21 – 4.11 (m, 1H), 4.04 – 3.78 (m, 2H), 3.74 – 3.42 (m, 4H), 3.30 – 3.19 (m, 2H), 2.68 – 2.57 (m, 2H), 1.75 – 1.54 (m, 6H), 1.41 (h, *J* = 7.3 Hz, 2H), 0.89 (t, *J* = 7.3 Hz, 3H). ^{13}C NMR (DMSO-d_6) δ 153.16, 151.27, 148.27, 142.15, 128.26, 128.17, 125.59, 88.87, 86.61, 71.32, 71.00, 62.22, 34.87, 32.20, 30.90, 28.73, 28.41, 21.18, 13.41. LC-MS (*m/z*): positive mode 488.3 [M + H]$^+$. Purity by HPLC-UV (254 nm)-ESI-MS: 95.0%.

(2*R*,3*R*,4*S*,5*R*)-2-(8-Bromo-6-(methylamino)-9*H*-purin-9-yl)-5-(hydroxymethyl)tetra-hydrofuran-3,4-diol (43a)

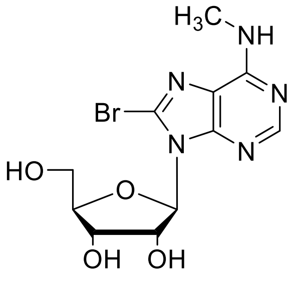

The compound was synthesized using **7a** (1.96 g, 7.00 mmol) and afforded a white solid (0.60 g, 25%), mp: 228 °C. ^{1}H-NMR (500 MHz, DMSO-d_6) δ 8.20 (s, 1H), 8.02 (s, 1H), 5.84 (d, 1H, *J* = 7.08 Hz), 5.45 (q, 1H, *J* = 4.07 Hz), 5.41 (d, 1H, *J* = 6.77 Hz), 5.19 (d, 1H, *J* = 4.60 Hz), 5.07 (dd, 1H, *J* = 6.55, 11.33 Hz), 4.20 (m, 1H), 3.97 (dd, 1H, *J* = 4.07, 5.66 Hz), 3.69-3.49 (d m, 2H), 2.94 (s, 3H). ^{13}C-NMR (125 MHz, DMSO-d_6) δ 154.12, 152.58, 149.04, 126.87, 120.40,

90.57, 86.84, 71.34, 70.99, 62.24, 27.10. LC-MS (*m/z*): positive mode 361.4 $[M + H]^+$. Purity by HPLC-UV (254 nm)-ESI-MS: 95.6%.

(2*R*,3*R*,4*S*,5*R*)-2-(8-Bromo-6-(ethylamino)-9*H*-purin-9-yl)-5-(hydroxymethyl)tetra-hydrofuran-3,4-diol (43b)

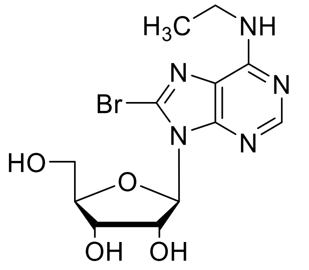

The compound was synthesized using **7b** (2.00 g, 7.00 mmol) and afforded a white solid (0.38 g, 14%). ^{1}H-NMR (500 MHz, DMSO-d_6) δ 8.18 (s, 1H), 8.10 (s, 1H), 5.82 (d, 1H, *J* = 6.71 Hz), 5.49 (dd, 1H, *J* = 3.91, 8.68 Hz), 5.43 (d, 1H, *J* = 6.29 Hz), 5.21 (d, 1H, *J* = 4.45 Hz), 5.07 (td, 1H, *J* = 5.15, 6.54 Hz), 4.19 (td, 1H, *J* = 2.41, 4.89 Hz), 3.7 (td, 1H, *J* = 2.25, 3.94 Hz), 3.67-3.52 (d m, 2H), 3.49 (m, 2H), 1.15 (t, 3H, *J* = 7.13 Hz). ^{13}C-NMR (125 MHz, DMSO-d_6) δ 153.57, 152.62, 149.25, 126.96, 120.32, 90.32, 86.92, 71.39, 71.06, 62.30, 34.81, 14.79. LC-MS (*m/z*): negative mode 373.8 $[M - H]^-$. Purity by HPLC-UV (254 nm)-ESI-MS: 95.4%.

(2*R*,3*R*,4*S*,5*R*)-2-(8-Bromo-6-(dimethylamino)-9*H*-purin-9-yl)-5-(hydroxymethyl)tetra-hydrofuran-3,4-diol (43c)

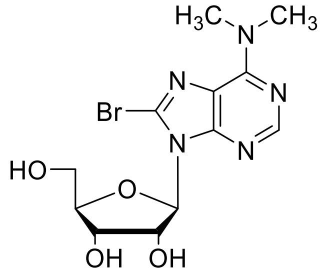

The compound was synthesized using **7g** (2.00 g, 7.00 mmol) and afforded a white solid (0.60 g, 21%), mp: 152 °C. ^{1}H-NMR (500 MHz, DMSO-d_6) δ 8.18 (s, 1H), 5.84 (d, 1H, *J* = 6.47 Hz), 5.41 (overlapping q and d, 2H), 5.19 (d, 1H, *J* = 4.68 Hz), 5.08 (dd, 1H, *J* = 6.48, 11.80 Hz), 4.21 (m, 1H), 3.97 (m, 1H), 3.70-3.49 (d m, 2H), 3.41 (br s, 6H). ^{13}C-NMR (125 MHz, DMSO-d_6) δ 153.29, 151.72, 150.88, 126.06, 120.37, 90.68, 86.80, 71.12, 70.96, 62.25, 56.16, 18.68. LC-MS (*m/z*): positive mode 374.2 $[M + H]^+$. Purity by HPLC-UV (254 nm)-ESI-MS: 96.6%.

(2*R*,3*R*,4*S*,5*R*)-2-(8-Bromo-6-(diethylamino)-9*H*-purin-9-yl)-5-(hydroxymethyl)tetra-hydrofuran-3,4-diol (43d)

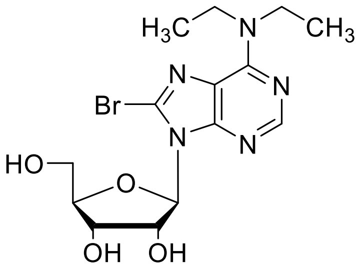

The compound was synthesized using **7j** (1.92 g, 5.90 mmol) and afforded a white solid (0.52 g, 23%). ^{1}H-NMR (500 MHz, DMSO-*d*$_6$) δ 8.17 (s, 1H), 5.84 (d, 1H, *J* = 6.75 Hz), 5.45 (dd, 1H, *J* = 3.87, 8.57 Hz), 5.42 (d, 1H, *J* = 5.89 Hz), 5.20 (d, 1H, *J* = 4.40 Hz), 5.09 (q, 1H, *J* = 5.92 Hz), 4.19 (td, 1H, *J* = 2.45, 4.76 Hz), 3.97 (td, 1H, *J* = 2.97, 4.04 Hz), 4.19-3.7 (br s, 4H, overlapping with previous peaks N(C*H*$_2$CH$_3$)$_2$), 3.67-3.51 (d m, 2H), 1.18 (t, 6H, *J* = 6.89 Hz). ^{13}C-NMR (125 MHz, DMSO-*d*$_6$) δ 152.14, 151.88, 150.94, 126.35, 119.92, 90.70, 86.85, 71.08, 62.29, 56.19, 42.87, 18.70, 13.65. LC-MS (*m/z*): negative mode 402.0 [M - H]$^-$. Purity by HPLC-UV (254 nm)-ESI-MS: 97.6%.

(2*R*,3*R*,4*S*,5*R*)-2-(6,8-Bis(methylamino)-9*H*-purin-9-yl)-5-(hydroxymethyl)tetrahydrofuran-3,4-diol (44a)

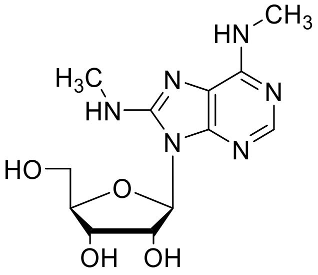

The compound was synthesized using **43a** (92 mg, 0.26 mmol), 40% methylamine in MeOH (10 mL) and afforded a brownish solid (81 mg, 100%), mp: >300 °C. ^{1}H NMR (600 MHz, DMSO-*d*$_6$) δ 7.97 (s, 1H), 6.92 (q, *J* = 4.6 Hz, 1H), 6.86 (q, *J* = 4.7 Hz, 1H), 5.92 (t, *J* = 5.2 Hz, 1H), 5.86 (d, *J* = 7.2 Hz, 1H), 5.23 (d, *J* = 6.5 Hz, 1H), 5.18 – 5.09 (m, 1H), 4.66 (q, *J* = 5.6 Hz, 1H), 4.13 (d, *J* = 5.3 Hz, 1H), 3.96 (q, *J* = 2.5 Hz, 1H), 3.68 – 3.57 (m, 2H), 2.92 (d, *J* = 4.7 Hz, 3H), 2.87 (d, *J* = 4.6 Hz, 3H). ^{13}C NMR (151 MHz, D$_2$O) δ 155.56, 149.93, 149.21, 146.64, 116.88, 89.81, 87.54, 73.79, 73.03, 67.56, 31.96, 31.04. LC-MS (*m/z*): positive mode 311.10 [M + H]$^+$. Purity by HPLC-UV (254 nm)-ESI-MS: 95.4%.

(2*R*,3*R*,4*S*,5*R*)-2-(8-(Butylamino)-6-(methylamino)-9*H*-purin-9-yl)-5-(hydroxymethyl)-tetrahydrofuran-3,4-diol (44b)

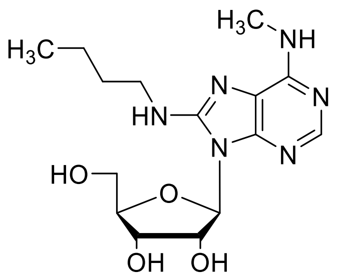

The compound was synthesized using **43a** (400 mg, 1.10 mmol), *N*-butylamine (10 mL) and afforded a yellowish solid (360 mg, 93%), mp: 202 °C. ^{1}H-NMR (500 MHz, DMSO-*d*$_6$) *δ* 7.95 (s, 1H), 6.83 (t, 1H, *J* = 5.51 Hz), 6.77 (q, 1H, *J* = 4.74 Hz), 5.89 (d, 1H, *J* = 7.69 Hz), 5.84 (br s, 1H), 5.19 (br s, 1H), 5.11 (br s, 1H), 4.62 (br s, 1H), 4.11 (br s, 1H), 3.95 (br d, 1H, *J* = 1.98 Hz), 3.62 (br s, 2H), 3.36 (m overlapping with H_2O, 2H), 2.92 (d, 3H, *J* = 4.78 Hz), 1.56 (m, 2H), 1.33 (m, 2H), 0.89 (t, 3H, *J* = 7.38 Hz). ^{13}C-NMR (125 MHz, DMSO-*d*$_6$) *δ* 152.01, 151.35, 148.86, 148.59, 117.62, 86.45, 85.78, 71.09, 70.87, 61.79, 42.17, 31.00, 29.44, 27.44, 19.78, 13.19. LC-MS (*m/z*): positive mode 353.0 [M + H]$^+$. Purity by HPLC-UV (254 nm)-ESI-MS: 91.4%.

(2*R*,3*R*,4*S*,5*R*)-2-(8-(Butylamino)-6-(ethylamino)-9*H*-purin-9-yl)-5-(hydroxymethyl)-tetrahydrofuran-3,4-diol (44c)

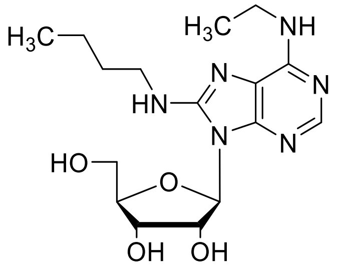

The compound was synthesized using **43b** (380 mg, 1.00 mmol), butylamine (10 mL) and afforded a yellowish solid (230 mg, 64%), mp: 192 °C. ^{1}H-NMR (500 MHz, DMSO-*d*$_6$) *δ* 7.93 (s, 1H), 6.82 (t, 1H, *J* = 5.41 Hz), 6.78 (t, 1H, *J* = 5.93 Hz), 5.88 (d, 1H, *J* = 7.44 Hz), 5.84 (t, 1H, *J* = 4.98 Hz), 5.18 (d, 1H, *J* = 6.64 Hz), 5.11 (d, 1H, *J* = 3.87 Hz), 4.62 (q, 1H, *J* = 6.27 Hz), 4.10 (br s, 1H), 3.95 (d, 1H, *J* = 2.08 Hz), 3.61 (m, 2H), 3.48 (m, 2H), 2.76 (m, 2H), 1.32 (m, 4H), 1.13 (t, 3H, *J* = 7.09 Hz), 0.87 (m, 3H). ^{13}C-NMR (125 MHz, DMSO-*d*$_6$) *δ* 151.43, 151.37, 149.14, 148.60, 117.42, 86.48, 85.83, 71.13, 70.90, 61.82, 56.20, 42.19, 31.03, 19.82 15.51, 13.64. LC-MS (*m/z*): positive mode 366.8 [M + H]$^+$. Purity by HPLC-UV (254 nm)-ESI-MS: 94%.

(2*R*,3*R*,4*S*,5*R*)-2-(6-(Dimethylamino)-8-((4-phenylbutyl)amino)-9*H*-purin-9-yl)-5-(hydroxylmethyl)tetrahydrofuran-3,4-diol (44d)

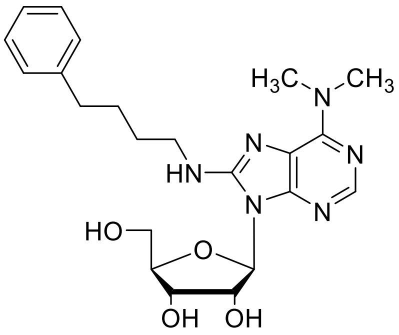

The compound was synthesized using **43c** (350 mg, 0.93 mmol), (4-phenyl)butylamine (10 mL) and afforded a white powder (160 mg, 36%), mp: 164.2 °C. ^{1}H-NMR (500 MHz, DMSO-*d$_6$*) *δ* 7.95 (s, 1H), 7.24-7.16 (d m, 5H), 6.88 (t, 1H, *J* = 5.39 Hz), 5.91 (d, 1H, *J* = 7.42 Hz), 5.83 (t, 1H, *J* = 4.93 Hz), 5.18 (d, 1H, *J* = 6.82 Hz), 5.10 (d, 1H, *J* = 4.10 Hz), 4.61 (m, 2H), 4.10 (q, 2H, *J* = 4.50 Hz), 3.95 (m, 1H), 3.62 (d, 2H, *J* = 4.54 Hz), 3.34 (s, 6H), 2.60 (t, 2H, *J* = 6.89 Hz), 1.62 (m, 4H). ^{13}C-NMR (125 MHz, DMSO-*d$_6$*) *δ* 151.64, 150.63, 150.17, 148.09, 142.34, 128.41, 128.36, 125.76, 117.82, 86.48, 85.79, 71.11, 70.82, 61.78, 42.09, 40.24, 37.91, 34.97, 28.53, 28.45. LC-MS (*m/z*): positive mode 443.2 [M + H]$^+$. Purity by HPLC-UV (254 nm)-ESI-MS: 94%.

(2*R*,3*R*,4*S*,5*R*)-2-(6-(Diethylamino)-8-(methylamino)-9*H*-purin-9-yl)-5-(hydroxyl-methyl)tetrahydrofuran-3,4-diol (44e)

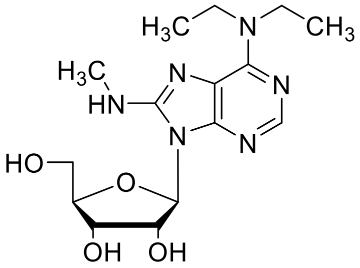

The compound was synthesized using **43d** (520 mg, 1.30 mmol), 40% methylamine in MeOH (10 mL) and afforded a white powder (300 mg, 67%), mp: 115 °C. ^{1}H-NMR (500 MHz, DMSO-*d$_6$*) *δ* 7.94 (d, 1H, *J* = 0.97 Hz), 6.81 (q, 1H, *J* = 4.38 Hz), 5.87 (d, 1H, *J* = 7.23 Hz), 5.85 (m, 2H), 5.17 (d, 1H, *J* = 6.63 Hz), 5.05 (m, 1H), 4.65 (q, 1H, *J* = 6.71 Hz), 4.11 (br s, 1H), 3.95 (d, 1H, *J* = 1.96 Hz), 3.87 (q, 4H, *J* = 6.09 Hz), 3.62 (m, 2H), 3.08 (q, 3H, *J* = 7.26 Hz), 1.16 (m, 6H). ^{13}C-NMR (125 MHz, DMSO-*d$_6$*) *δ* 151.00, 150.62, 150.51, 148.30, 117.27, 86.55, 85.77, 71.08, 70.81, 61.78, 45.90, 42.07, 28.98,14.04, 8.74. LC-MS (*m/z*): positive mode 352.9 [M + H]$^+$. Purity by HPLC-UV (254 nm)-ESI-MS: 98%.

(2*R*,3*R*,4*S*,5*R*)-2-(8-(Butylamino)-6-(diethylamino)-9*H*-purin-9-yl)-5-(hydroxymethyl)-tetrahydrofuran-3,4-diol (44f)

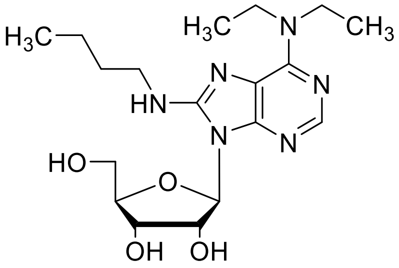

The compound was synthesized using **43d** (740 mg, 1.83 mmol), butylamine (10 mL) and afforded a yellowish solid (700 mg, 100%), mp: 88 °C. ^{1}H-NMR (500 MHz, DMSO-*d*$_6$) *δ* 7.93 (s, 1H), 6.83 (t, 1H, *J* = 5.46 Hz), 5.90 (d, 1H, *J* = 7.46 Hz), 5.84 (t, 1H, *J* = 5.00 Hz), 5.18 (d, 1H, *J* = 6.84 Hz), 5.10 (d, 1H, *J* = 4.08 Hz), 4.10 (m, 1H), 4.08 (m, 1H), 3.95 (q, 1H, *J* = 1.99 Hz), 3.85 (m, 4H), 3.61 (m, 2H), 3.16 (d, 2H, *J* = 5.21 Hz), 1.57 (q, 2H, *J* = 7.16 Hz), 1.33 (m, 2H), 1.15 (t, 6H, *J* = 6.94 Hz), 0.88 (t, 3H, *J* = 7.35 Hz). ^{13}C-NMR (125 MHz, DMSO-*d*$_6$) *δ* 150.56, 150.43, 150.39, 148.27, 117.22, 86.44, 85.80, 71.14, 70.82, 61.82, 56.20, 48.77, 42.19, 41.95, 30.95, 19.74, 18.72, 14.08, 13.87. LC-MS (*m/z*): positive mode 395.1 [M + H]$^+$. Purity by HPLC-UV (254 nm)-ESI-MS: 94%.

(2*R*,3*R*,4*S*,5*R*)-2-(8-(Butylthio)-6-(methylamino)-9*H*-purin-9-yl)-5-(hydroxymethyl-)tetrahydrofuran-3,4-diol (45a)

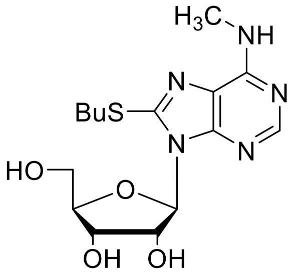

The compound was synthesized using **43a** (500 mg, 1.39 mmol), 1-thionaphthol (0.89 mL, 8.34 mmol) and afforded a white solid (210 mg, 41%), mp: 144 °C. ^{1}H-NMR (500 MHz, DMSO-*d*$_6$) *δ* 8.13 (br s, 1H), 7.63 (br s, 1H), 5.77 (d, 1H, *J* = 6.89 Hz), 5.62 (dd, 1H, *J* = 3.61, 8.93 Hz), 5.37 (d, 1H, *J* = 6.42 Hz), 5.16 (d, 1H, *J* = 4.29 Hz), 4.98 (q, 1H, *J* = 6.50 Hz), 4.15 (m, 1H), 3.96 (q, 1H, *J* = 3.70 Hz), 3.68-3.49 (d m, 2H), 3.26 (m, 2H), 2.96 (br s, 3H), 1.67 (m, 2H), 1.40 (m, 2H), 0.89 (t, 3H, *J* = 7.38 Hz). ^{13}C-NMR (125 MHz, DMSO-*d*$_6$) *δ* 153.80, 151.47, 148.49, 128.29, 127.32, 89.04, 86.79, 71.54, 71.17, 62.41, 32.27, 31.11, 27.17, 21.37, 13.59. LC-MS (*m/z*): positive mode 370.1 [M + H]$^+$. Purity by HPLC-UV (254 nm)-ESI-MS: 90.1%.

(2*R*,3*R*,4*S*,5*R*)-2-(8-(Butylthio)-6-(diethylamino)-9*H*-purin-9-yl)-5-(hydroxymethyl-)tetrahydrofuran-3,4-diol (45b)

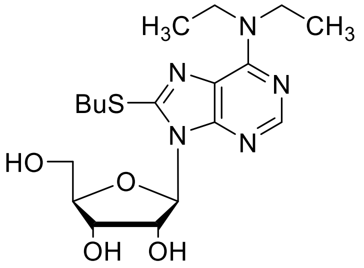

The compound was synthesized using **43d** (740 mg, 1.83 mmol), 1-thionaphthol (0.40 mL, 3.70 mmol) and afforded a white solid (90 mg, 12%), mp: 147 °C. ^{1}H-NMR (500 MHz, DMSO-*d$_6$*) *δ* 8.10 (s, 1H), 5.72 (t, 1H, *J* = 6.89 Hz), 5.60 (dd, 1H, *J* = 3.43, 8.71 Hz), 5.36 (d, 1H, *J* = 5.22 Hz), 5.16 (m, 1H), 4.98 (d, 1H, *J* = 5.24 Hz), 4.15 (s, 1H), 3.95 (m, 1H), 4.15-3.65 (large bulb, 4H, underneath other peaks), 3.65-3.51 (d m, 2H), 3.25 (m, 2H), 1.72 (m, 2H), 1.40 (m, 2H), 1.19 (t, 6H, *J* = 6.69 Hz), 0.89 (t, 3H, *J* = 7.39 Hz). ^{13}C-NMR (125 MHz, DMSO-*d$_6$*) *δ* 151.78, 151.54, 150.81, 147.96, 119.80, 88.99, 86.78, 71.31, 71.16, 62.44, 42.61, 31.88, 31.39, 21.56, 13.60 (missing: N(C*H$_2$*CH$_3$)$_2$). LC-MS (*m/z*): positive mode 412.0 [M + H]$^+$. Purity by HPLC-UV (254 nm)-ESI-MS: 98.5%.

(2*R*,3*R*,4*S*,5*R*)-2-(6-(Diethylamino)-8-(naphthalen-1-ylthio)-9*H*-purin-9-yl)-5-(hydroxymethyl)tetrahydrofuran-3,4-diol (45c)

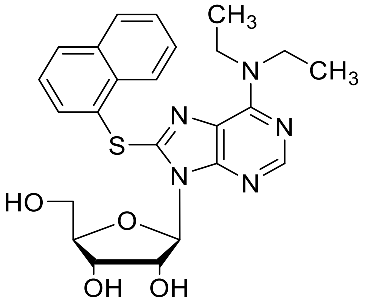

The compound was synthesized using **43d** (310 mg, 0.77 mmol), 1-thionaphthol (1.29 mL, 9.24 mmol) and afforded a white solid (328 mg, 88%), mp: 93-95 °C. ^{1}H NMR (500 MHz, DMSO-*d*$_6$) *δ* 8.36 – 8.26 (m, 1H), 8.13 (s, 1H), 8.08 – 7.97 (m, 2H), 7.83 – 7.77 (m, 1H), 7.64 – 7.50 (m, 3H), 6.09 (dd, *J* = 6.9, 1.4 Hz, 1H), 5.63 – 5.54 (m, 1H), 5.49 – 5.37 (m, 1H), 5.25 – 5.17 (m, 1H), 5.14 – 5.04 (m, 1H), 4.27 – 4.17 (m, 1H), 4.07 – 3.94 (m, 1H), 3.75 – 3.52 (m, 6H), 1.37 – 0.76 (m, 6H). ^{13}C NMR (126 MHz, DMSO-*d*$_6$) *δ* 151.87, 151.28, 151.03, 144.96, 133.85, 133.08, 132.98, 130.12, 128.52, 127.18, 126.65, 126.47, 125.90, 124.99, 119.68, 89.38, 86.73, 71.34, 70.99, 62.20, 55.99, 12.96. LC-MS (*m/z*): positive mode 482.20 [M + H]$^+$. Purity by HPLC-UV (254 nm)-ESI-MS: 92.7%.

(2*R*,3*S*,4*R*,5*R*)-2-(Hydroxymethyl)-5-(8-phenyl-6-((4-phenylbutyl)amino)-9*H*-purin-9-yl)tetrahydrofuran-3,4-diol (46)

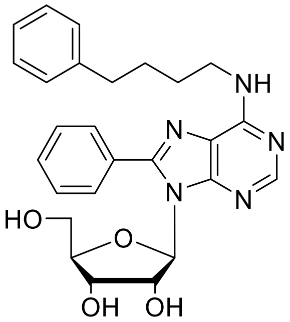

To a solution of **7r** (400 mg, 1.00 mmol, 1 equiv.) in dry DMF (10 mL), iodobenzene (0.22 mL, 2.00 mmol, 2 equiv.), $Pd(OAc)_2$ (11 mg, 0.05 mmol, 0.05 equiv.), CuI (571 mg, 3.00 mmol, 3 equiv.) and Cs_2CO_3 (815 mg, 2.50 mmol, 2.5 equiv.) were added. The mixture was stirred in an autoclave at 120 °C under argon overnight. The reaction progress was monitored by TLC (MeOH/DCM, 1:9). After the reaction was completed, cooled to rt, 1 M HCl (aq., 10 mL) was added. The mixture was then neutralized with 2 M NaOH (aq.) and extracted with EtOAc (50 mL × 3). The collected organic layers were dried over $MgSO_4$ and evaporated *in vacuum*. The crude compound was purified by silica gel column chromatography (4% MeOH in DCM), and afforded a brown solid (134 mg, 28%), mp: 88.0-90.0 °C. ^{1}H NMR (500 MHz, DMSO-d_6) δ 8.23 (s, 1H), 8.08 (s, 1H), 7.75 (dd, J = 6.8, 3.0 Hz, 2H), 7.61 – 7.56 (m, 3H), 7.32 – 7.09 (m, 5H), 5.77 (d, J = 7.0 Hz, 2H), 5.42 (s, 1H), 5.19 (t, J = 6.1 Hz, 1H), 5.10 (s, 1H), 4.18 (dd, J = 5.2, 2.0 Hz, 1H), 3.98 – 3.90 (m, 1H), 3.71 (dd, J = 12.2, 3.7 Hz, 1H), 3.64 – 3.44 (m, 3H), 2.67 – 2.56 (m, 2H), 1.63 (p, J = 3.6 Hz, 4H). ^{13}C NMR (126 MHz, DMSO-d_6) δ 154.59, 151.93, 150.65, 148.95, 142.15, 130.03, 129.61, 129.39, 128.69, 128.25, 128.14, 125.55, 119.49, 89.11, 86.68, 71.27, 71.05, 62.28, (NH*C*H_2 is overlapping with DMSO-d_6), 34.83, 28.67, 28.39. LC-MS (*m/z*): positive mode 476.30 $[M + H]^+$. Purity by HPLC-UV (254 nm)-ESI-MS: 98.4%.

2,6-Dichloro-9-(2,3,5-tri-*O*-acetyl-*β*-*D*-ribofuranosyl)-9*H*-purine (51), CAS: 3056-18-6

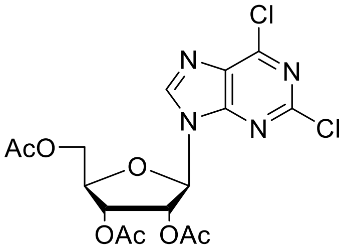

Tetraacetylribose (**50**, 3.37 g, 10.58 mmol, 1 equiv.) was melted at 110 °C, then 2,6-dichloropurine (2.00 g, 10.58 mmol, 1 equiv.) and triflic acid (0.05 mL, 0.53 mmol, 0.05 equiv.) were added. The mixture was stirred at 110 °C and 0.09 MPa for removing the CH_3CO_2H which was produced during the reaction. The reaction was monitored by TLC (MeOH/DCM, 1:9). After 1 h, the reaction was completed, cooled to rt, MeOH (10 mL) and 5 g silica gel were added, and the mixture was concentrated *in vacuum*. The crude mixture was purified by silica

gel column chromatography (1.5% MeOH in DCM), and afforded a brown semi-solid (2.44 g, 52%). ^{1}H NMR (500 MHz, DMSO-d_6) δ 8.91 (s, 1H), 6.32 (d, J = 5.0 Hz, 1H), 5.90 (t, J = 5.4 Hz, 1H), 5.62 (t, J = 5.5 Hz, 1H), 4.46 – 4.37 (m, 2H), 4.30 (dd, J = 12.2, 5.5 Hz, 1H), 2.12 (s, 3H), 2.05 (s, 3H), 2.02 (s, 3H). ^{13}C NMR (126 MHz, DMSO-d_6) δ 169.93, 169.28, 169.14, 152.74, 151.28, 150.23, 146.79, 131.20, 86.19, 79.82, 72.32, 69.73, 62.57, 20.41, 20.29, 20.15. LC-MS (*m/z*): negative mode 447.0 [M - H]$^-$. Purity by HPLC-UV (254 nm)-ESI-MS: 90.0%.

(2*R*,3*R*,4*S*,5*R*)-2-(2-Chloro-6-((4-phenylbutyl)amino)-9*H*-purin-9-yl)-5-(hydroxymethyl)tetrahydrofuran-3,4-diol (52)

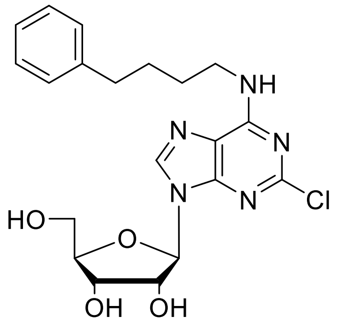

To a solution of **51** (2.00 g, 4.47 mmol, 1 equiv.) in EtOH (10 mL), 4-phenylbutylamine (1.41 mL, 8.94 mmol, 2 equiv.) and Et_3N (1.24 mL, 8.94 mmol, 2 equiv.) were added. The mixture was refluxed overnight and monitored by TLC (MeOH/DCM, 1:9). After the reaction was completed, cooled to rt and the solvent was evaporated *in vacuum*. The crude compound was purified by silica gel column chromatography (3% MeOH in DCM), and afforded a brown viscous semi-solid (0.74 g, 38%). LC-MS (*m/z*): positive mode 434.1 [M + H]$^+$. Purity by HPLC-UV (254 nm)-ESI-MS: 98.2%.

(2*R*,3*R*,4*S*,5*R*)-2-(2-Amino-6-((4-phenylbutyl)amino)-9*H*-purin-9-yl)-5-(hydroxymethyl)tetrahydrofuran-3,4-diol (55)

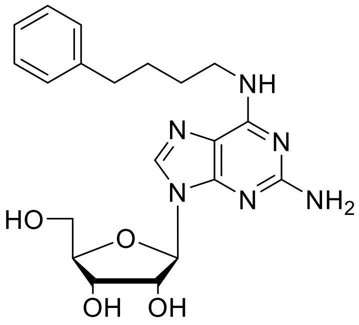

This compound was synthesized using the same procedure as for **41a**. 2-Amino-6-chloropurine riboside (**54**, 300 mg, 0.99 mmol), 4-phenylbutylamine (0.24 mL, 1.49 mmol) were

used, and afforded a yellow solid (305 mg, 74%), mp: 72.0-74.0 °C. ^{1}H NMR (600 MHz, DMSO-d_6) δ 7.89 (s, 1H), 7.45 – 7.02 (m, 6H), 5.72 (d, J = 6.3 Hz, 3H), 5.44 (t, J = 5.5 Hz, 1H), 5.33 (d, J = 6.2 Hz, 1H), 5.07 (d, J = 4.5 Hz, 1H), 4.51 (q, J = 5.9 Hz, 1H), 4.18 – 4.03 (m, 1H), 3.90 (q, J = 3.5 Hz, 1H), 3.64 (dt, J = 12.0, 3.9 Hz, 1H), 3.58 – 3.49 (m, 1H), 3.49 – 3.36 (m, 2H), 2.60 (t, J = 7.1 Hz, 2H), 1.59 (dhept, J = 6.2, 4.0, 3.4 Hz, 4H). ^{13}C NMR (151 MHz, DMSO-d_6) δ 160.45, 155.50, 151.05, 142.76, 136.33, 128.78, 128.67, 126.06, 114.23, 87.52, 85.99, 73.69, 71.19, 62.23, 35.40, 31.16, 29.44, 28.95. LC-MS (*m/z*): positive mode 415.0 [M + H]$^+$. Purity by HPLC-UV (254 nm)-ESI-MS: 98.5%.

(2*R*,3*R*,4*S*,5*R*)-2-(2-Bromo-6-chloro-9*H*-purin-9-yl)-5-(hydroxymethyl)tetrahydrofuran-3,4-diol (57)

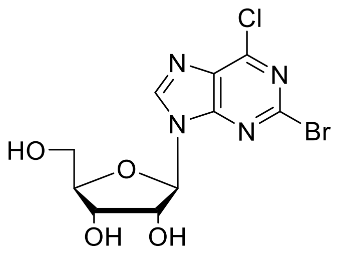

To a solution of (2*R*,3*R*,4*S*,5*R*)-2-(2-amino-6-chloro-9*H*-purin-9-yl)-5-(hydroxymethyl) tetrahydrofuran-3,4-diol (**54**, 400 mg, 1.33 mmol, 1 equiv.) in 20 mL CH_2Br_2, *tert*-butyl nitrite (1.57 mL, 26.5 mmol, 20 equiv.) was added dropwise, TMSBr (3.94 ml, 11.9 mmol, 9 equiv.) was added carefully. The mixture was then stirred at rt overnight and monitored by TLC (MeOH/DCM, 1:9). After the reaction was completed, the mixture was transferred dropwise into a mixture of 200 mL EtOAc and 200 mL saturated aqueous sodium bicarbonate. The organic phase was extracted with saturated aqueous sodium bicarbonate (200 mL $\times$ 2) and subsequently washed with brine. After drying with $MgSO_4$, the mixture was filtered through a pore 4 filter and the filtrate was evaporated *in vacuum*. The resulting residue was then purified by silica gel column chromatography (10% MeOH in DCM), and afforded a white solid (270 mg, 56%), mp: 176-178 °C. ^{1}H NMR (600 MHz, DMSO-d_6) δ 8.96 (s, 1H), 5.97 (d, J = 5.0 Hz, 1H), 5.65 (s, 1H), 5.32 (s, 1H), 5.10 (s, 1H), 4.52 (t, J = 5.0 Hz, 1H), 4.18 (t, J = 4.5 Hz, 1H), 3.99 (q, J = 4.0 Hz, 1H), 3.73 – 3.68 (m, 1H), 3.62 – 3.57 (m, 1H). ^{13}C NMR (151 MHz, DMSO-d_6) δ 153.06, 151.16, 149.59, 141.77, 131.31, 88.23, 85.78, 74.07, 69.94, 60.83. LC-MS (*m/z*): positive mode 366.9 [M + H]$^+$. Purity by HPLC-UV (254 nm)-ESI-MS: 94.0%.

(2*R*,3*S*,4*R*,5*R*)-2-(Hydroxymethyl)-5-(3*H*-imidazo[2,1-*i*]purin-3-yl)tetrahydrofuran-3,4-diol (60a), CAS: 39007-51-7

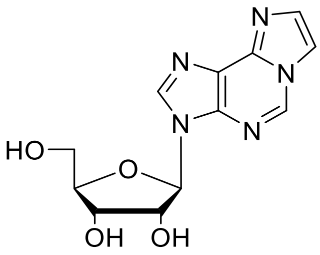

The compound was synthesized using adenosine (**4**, 600 mg, 2.25 mmol), and afforded a brown solid (605 mg, 92%). LC-MS (*m/z*): positive mode 292.20 $[M + H]^+$. Purity by HPLC-UV (254 nm)-ESI-MS: 78.4%.

(2*R*,3*R*,4*S*,5*R*)-2-(2-(Butylthio)-3*H*-imidazo[2,1-*i*]purin-3-yl)-5-(hydroxymethyl)tetrahydrofuran-3,4-diol (60b)

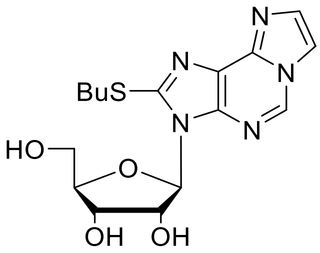

The compound was synthesized using **37** (100 mg, 0.28 mmol), and afforded a yellowish solid (122 mg, >100%), mp: 180.0-182.0 °C. ^{1}H NMR (500 MHz, DMSO-d_6) δ 9.24 (s, 1H), 8.06 (s, 1H), 7.58 (s, 1H), 5.90 (d, J = 6.3 Hz, 1H), 5.42 (d, J = 6.2 Hz, 1H), 5.25 – 5.17 (m, 1H), 5.05 (q, J = 5.6 Hz, 1H), 4.88 (s, 1H), 4.23 (s, 1H), 3.94 (d, J = 4.1 Hz, 1H), 3.70 (dd, J = 11.9, 5.1 Hz, 1H), 3.56 (d, J = 11.7 Hz, 1H), 3.36 (dd, J = 11.5, 7.1 Hz, 2H), 1.73 (p, J = 7.2 Hz, 2H), 1.45 (h, J = 7.4 Hz, 2H), 0.92 (t, J = 7.3 Hz, 3H). ^{13}C NMR (126 MHz, DMSO-d_6) δ 149.47, 139.58, 139.41, 135.60, 132.82, 123.52, 112.00, 88.99, 85.88, 71.18, 70.46, 61.87, 31.92, 30.95, 21.20, 13.40. LC-MS (*m/z*): positive mode 380.20 $[M + H]^+$. Purity by HPLC-UV (254 nm)-ESI-MS: 98.9%.

3 LC-MS spectra of selected AMP derivatives

LC-MS spectrum of compound **8r**

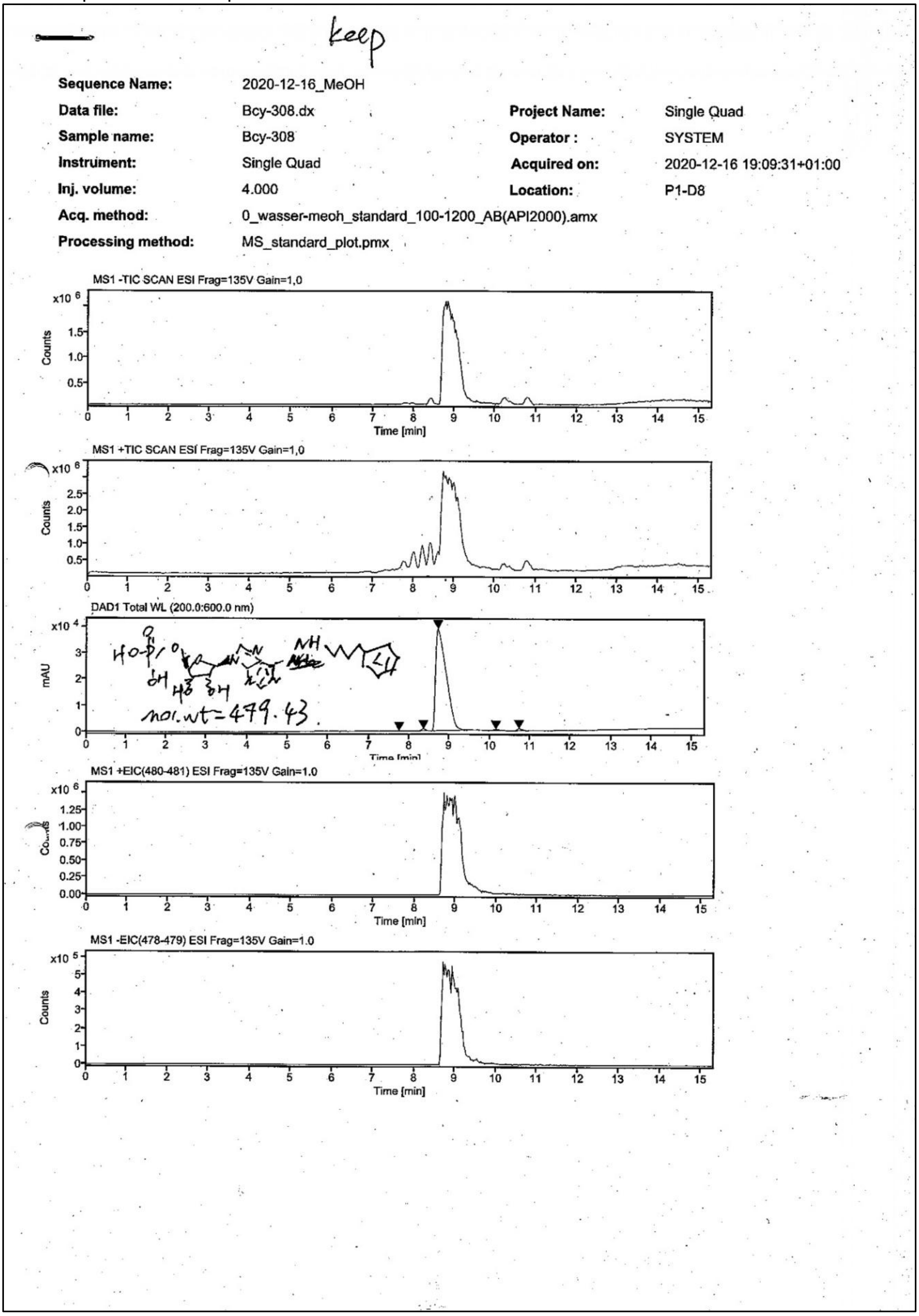

LC-MS spectrum of compound **8r** (continued)

[illegible]al: DAD1 Total WL (200.0:600.0 nm)

RT [min]	Peak MS Base Peak m/z	Area	Area%	Max Peak%	Height
7.750		1708.2469	0.2339	0.239	117.305
8.352		4299.8221	0.5886	0.601	681.634
8.710	480.300	714910.9848	97.8712	100.000	38871.961
10.153		4533.3820	0.6206	0.634	461.368
10.725		5008.9155	0.6857	0.701	650.189
	Sum	730461.3513			

Signal: MS1 +TIC SCAN ESI Frag=135V Gain=1,0

RT [min]	Peak MS Base Peak m/z	Area	Area%	Max Peak%	Height
8.736	480.300	76736721.2750	100.0000	100.000	2827055.226
	Sum	76736721.2750			

Signal: MS1 -TIC SCAN ESI Frag=135V Gain=1,0

RT [min]	Peak MS Base Peak m/z	Area	Area%	Max Peak%	Height
8.786	957.500	49717467.9780	100.0000	100.000	2013158.568
	Sum	49717467.9780			

LC-MS spectrum of compound **8r** (continued)

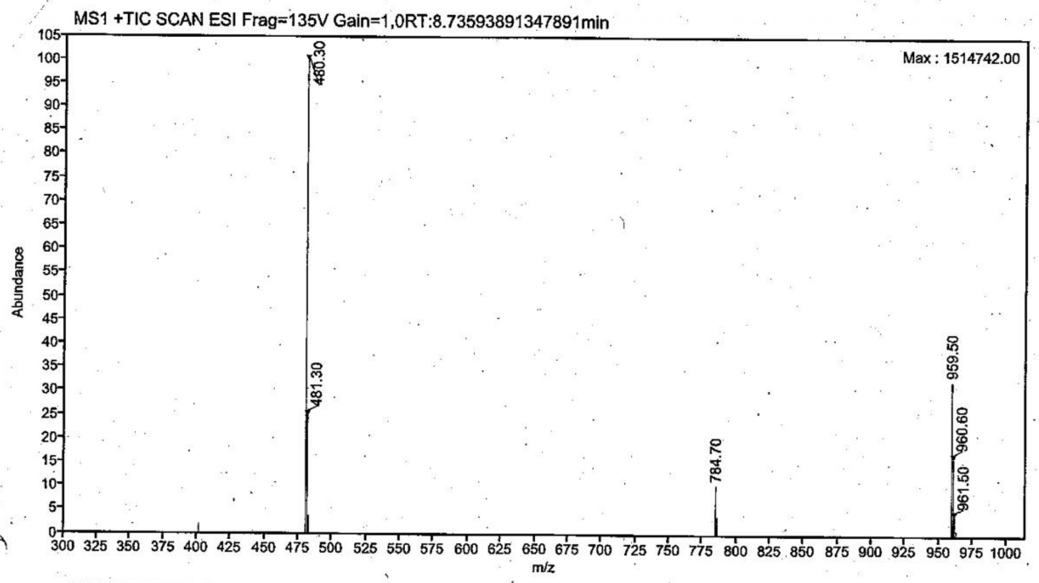

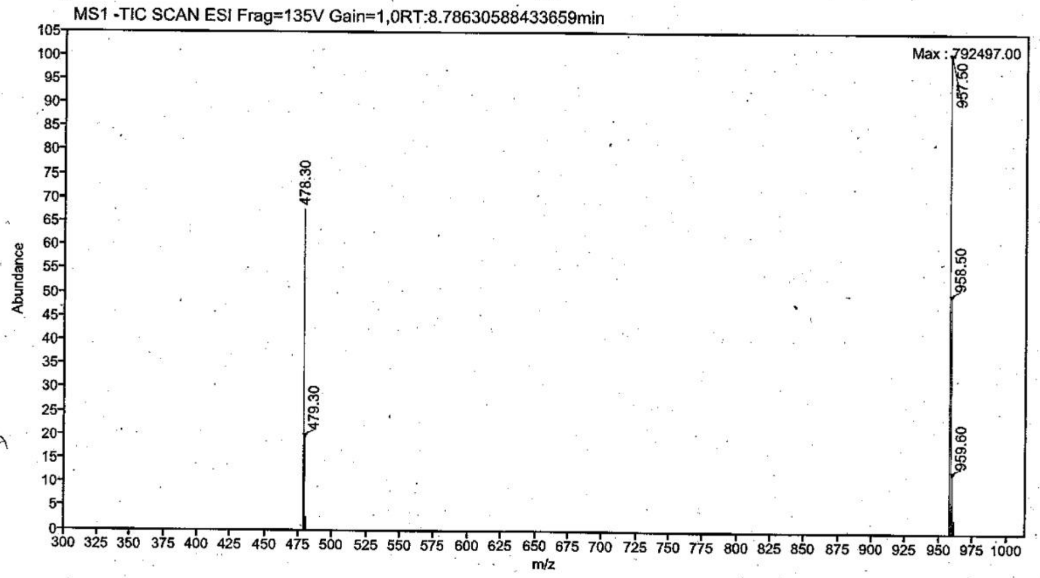

LC-MS spectrum of compound **8s**

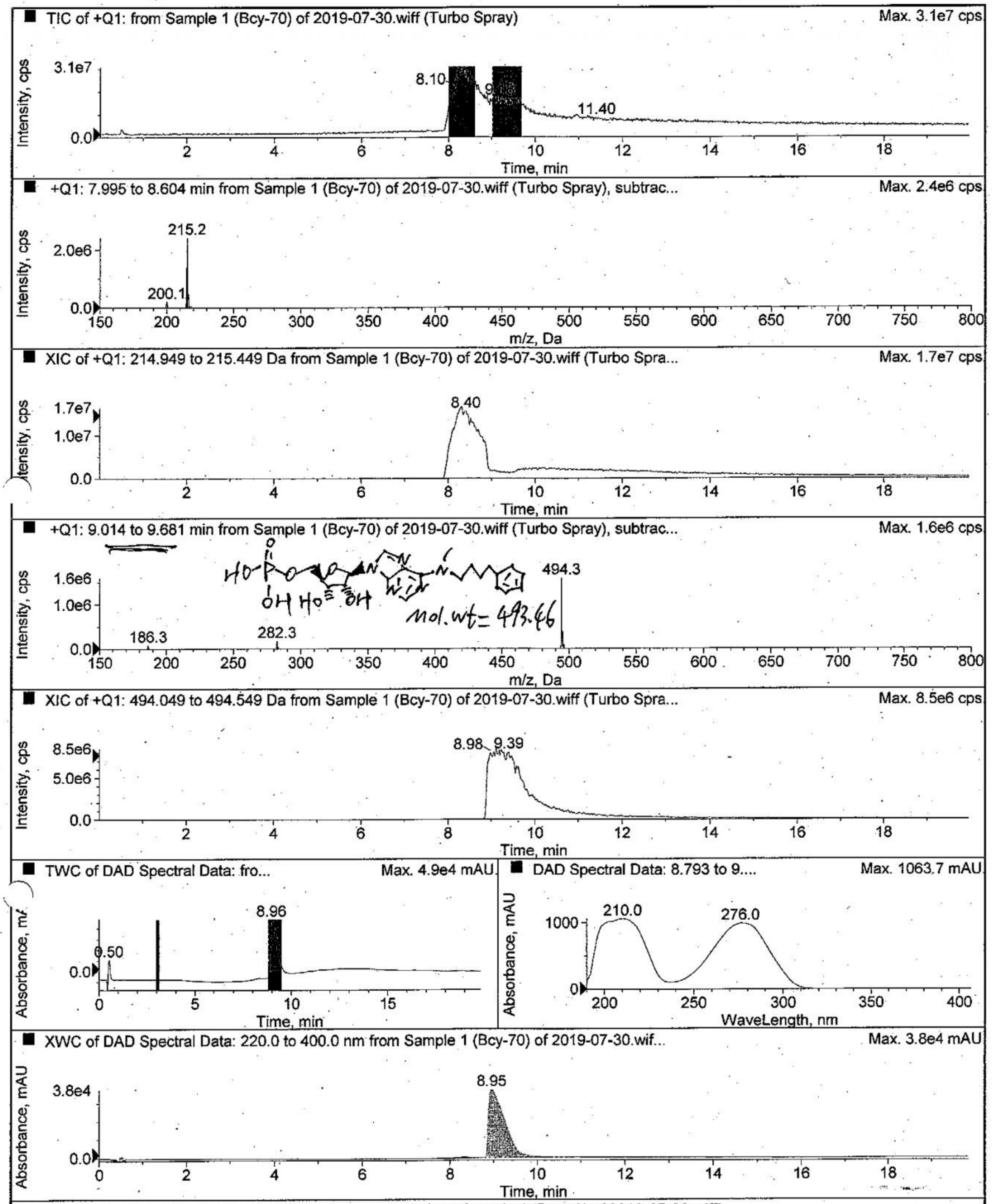

Peak List for "XWC of DAD Spectral Data: 220.0 to 400.0 nm from Sample 1 (Bcy-70) of 2019-07-30.wiff"

	Time (min)	Area (mAU x min)	% Area	Height (mAU)	% Height	Width (min)	Baseline Type
1	0.3788	5907.8807	0.5752	876.0336	2.1283	0.2133	Base to Base
2	0.5183	1.4380e4	1.4001	1935.1748	4.7015	0.2667	Base to Base
3	8.3256	1.2496e4	1.2166	591.8963	1.4380	0.9467	Base to Base
4	8.9532	9.9430e5	96.8081	3.7758e4	91.7321	1.8733	Base to Base

98.8%

LC-MS spectrum of compound **8v**

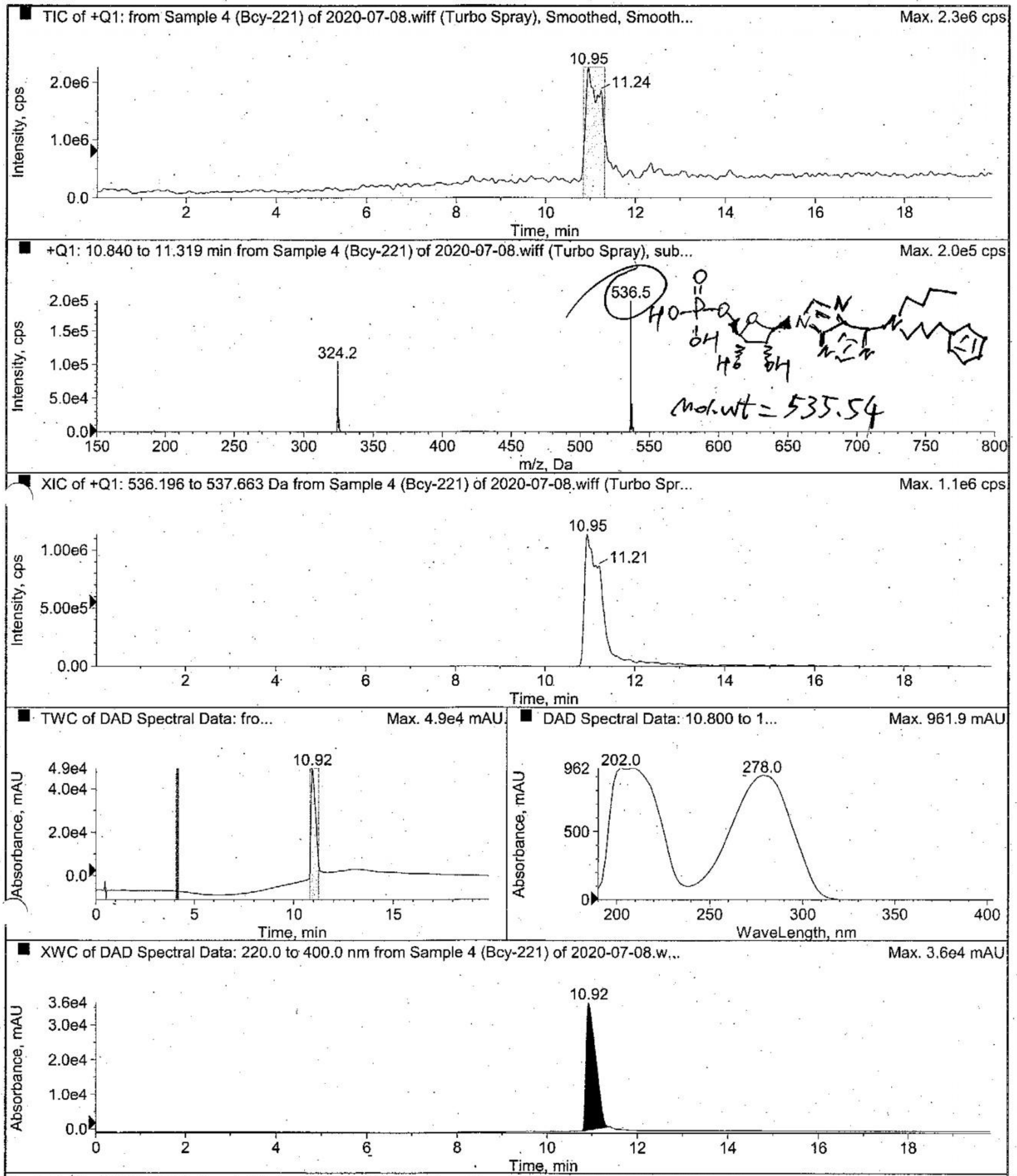

Peak List for "XWC of DAD Spectral Data: 220.0 to 400.0 nm from Sample 4 (Bcy-221) of 2020-07-08.wiff"

	Time (min)	Area (mAU x min)	%Area	Height (mAU)	%Height	Width (min)	Baseline Type
1	0.4590	1263.1684	0.2225	389.0842	1.0393	0.1267	Base to Base
2	0.6038	734.0584	0.1293	85.4925	0.2284	0.1400	Base to Base
3	10.9204	5.6471e5	99.4851	3.6721e4	98.0881	0.6133	Base to Base
4	11.4197	582.4669	0.1026	172.2921	0.4602	0.0933	Base to Base
5	12.2684	343.3113	0.0605	68.8942	0.1840	0.2467	Base to Base

99.8%.

LC-MS spectrum of compound **25a**

**Sequence Name:**	2021-01-14-b		
**Data file:**	Bcy-148K.dx	**Project Name:**	Single Quad
**Sample name:**	Bcy-148K	**Operator :**	SYSTEM
**Instrument:**	Single Quad	**Acquired on:**	2021-01-14 15:22:50+01:00
**Inj. volume:**	6.000	**Location:**	P1-E4
**Acq. method:**	0_wasser-meoh_standard_100-1200_AB(API2000).amx		
**Processing method:**	MS_standard_plot.pmx		

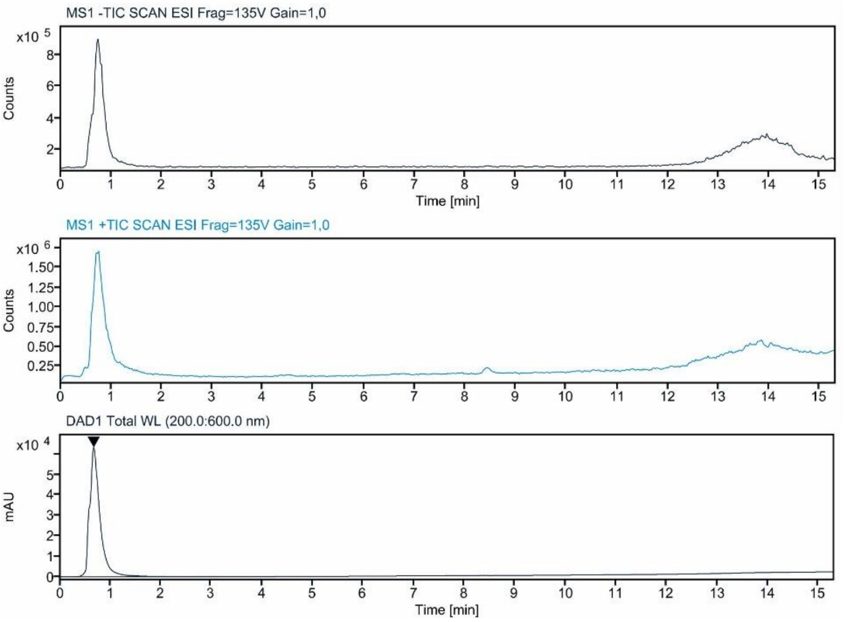

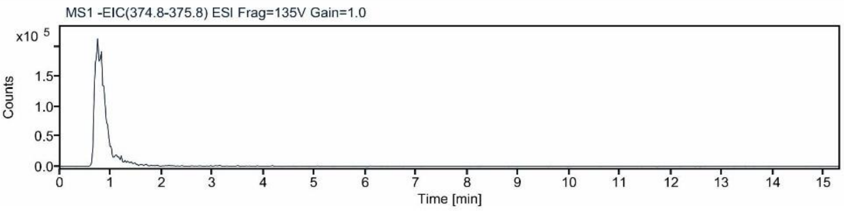

Signal: DAD1 Total WL (200.0:600.0 nm)

RT [min]	Peak MS Base Peak m/z	Area	Area%	Max Peak%	Height
0.669		900979.8842	100.0000	100.000	63327.080
	Sum	900979.8842			

Signal: MS1 +TIC SCAN ESI Frag=135V Gain=1,0

RT [min]	Peak MS Base Peak m/z	Area	Area%	Max Peak%	Height
0.751	377.200	31820629.2651	100.0000	100.000	1620863.574
	Sum	31820629.2651			

LC-MS spectrum of compound **25a** (continued)

Signal: MS1 -TIC SCAN ESI Frag=135V Gain=1,0

RT [min]	Peak MS Base Peak m/z	Area	Area%	Max Peak%	Height
0.742	751.400	13003088.1239	100.0000	100.000	824761.359
	Sum	13003088.1239			

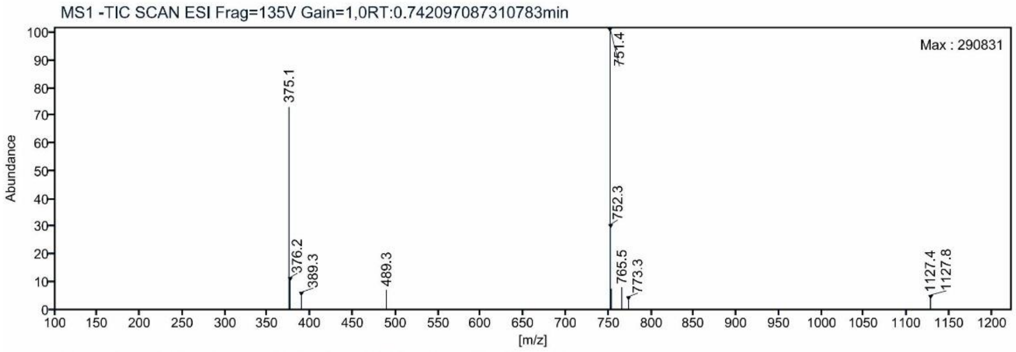

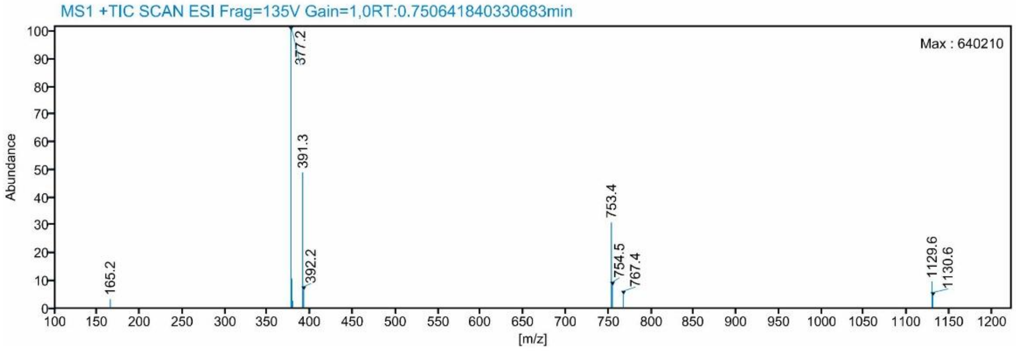

LC-MS spectrum of compound **31d**

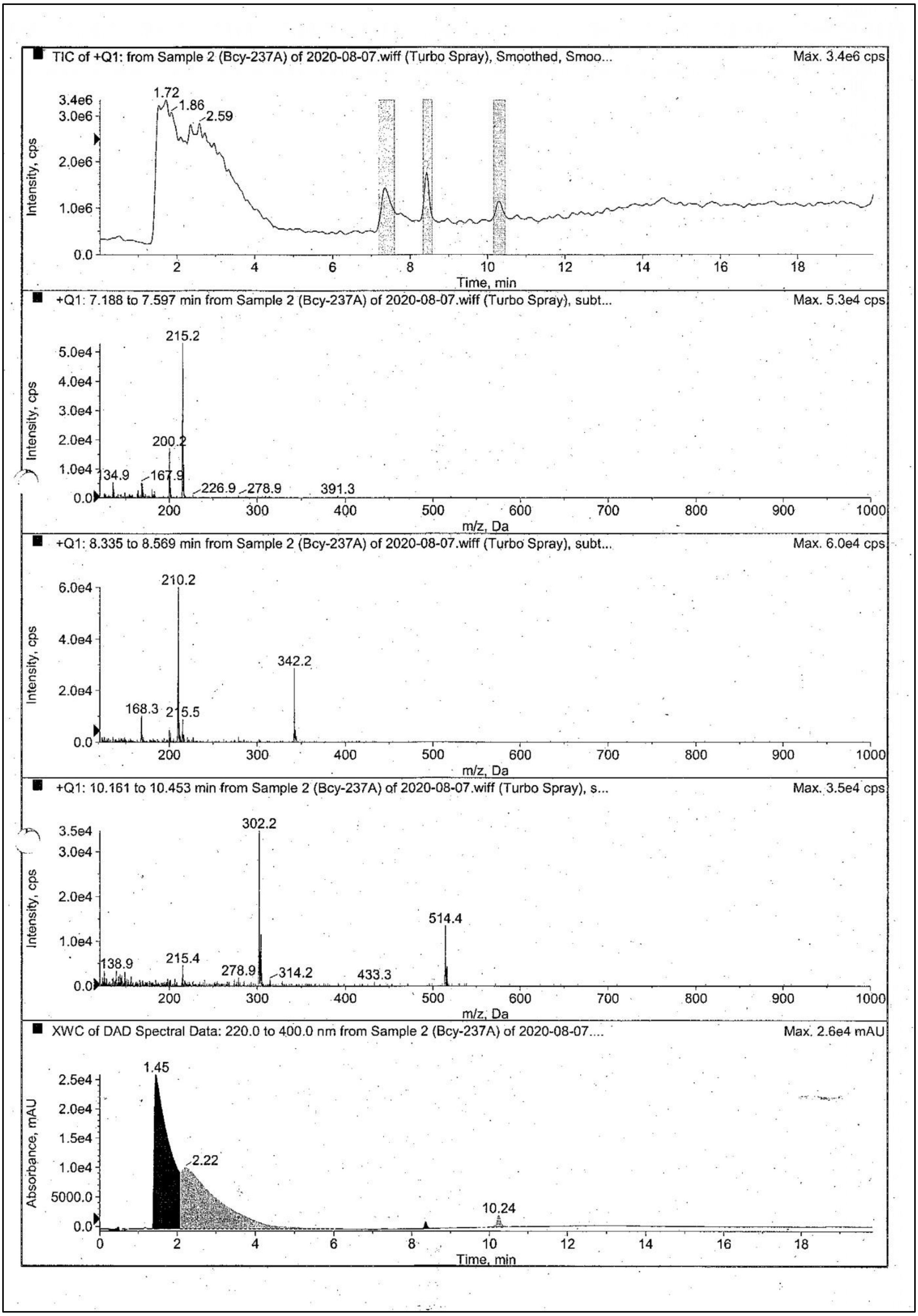

LC-MS spectrum of compound **31d** (continued)

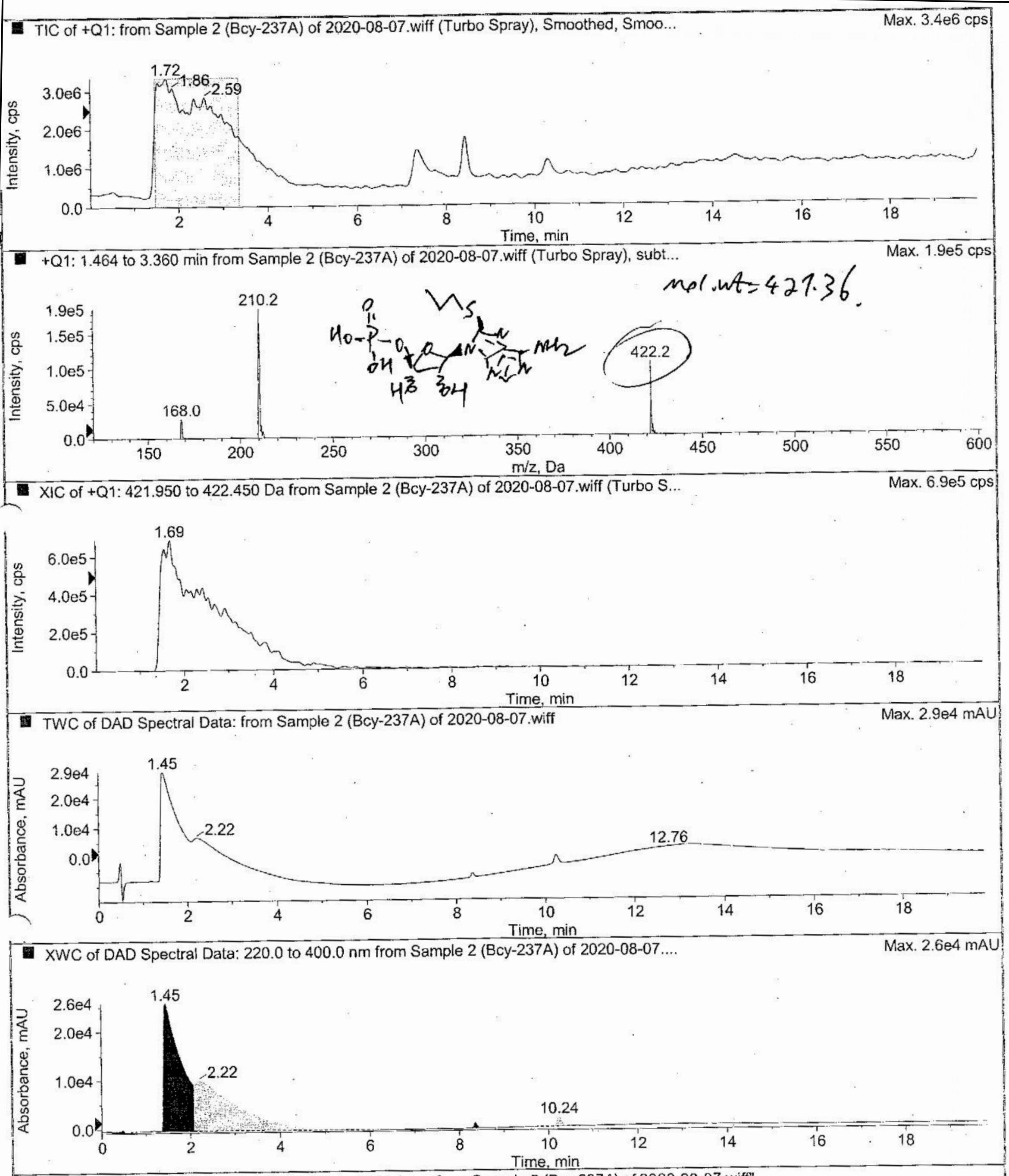

Peak List for "XWC of DAD Spectral Data: 220.0 to 400.0 nm from Sample 2 (Bcy-237A) of 2020-08-07.wiff"

	Time (min)	Area (mAU x min)	%Area	Height (mAU)	%Height	Width (min)	Baseline Type
1	0.4834	5698.3071	0.4100	678.8345	1.6709	0.3200	Valley
2	0.6024	2183.9832	0.1572	301.7535	0.7427	0.2200	Base to Base
3	1.4522	6.9477e5	49.9952	2.6244e4	64.5977	0.7933	Valley
4	2.2151	6.6647e5	47.9589	1.0356e4	25.4899	4.0200	Valley
5	8.3577	5268.3875	0.3791	1101.9841	2.7124	0.3133	Base to Base
6	10.2356	1.5281e4 → carry over	1.0996	1944.5687	4.7864	0.9533	Valley

Bcy-224-1 98.58

LC-MS spectrum of compound **31i**

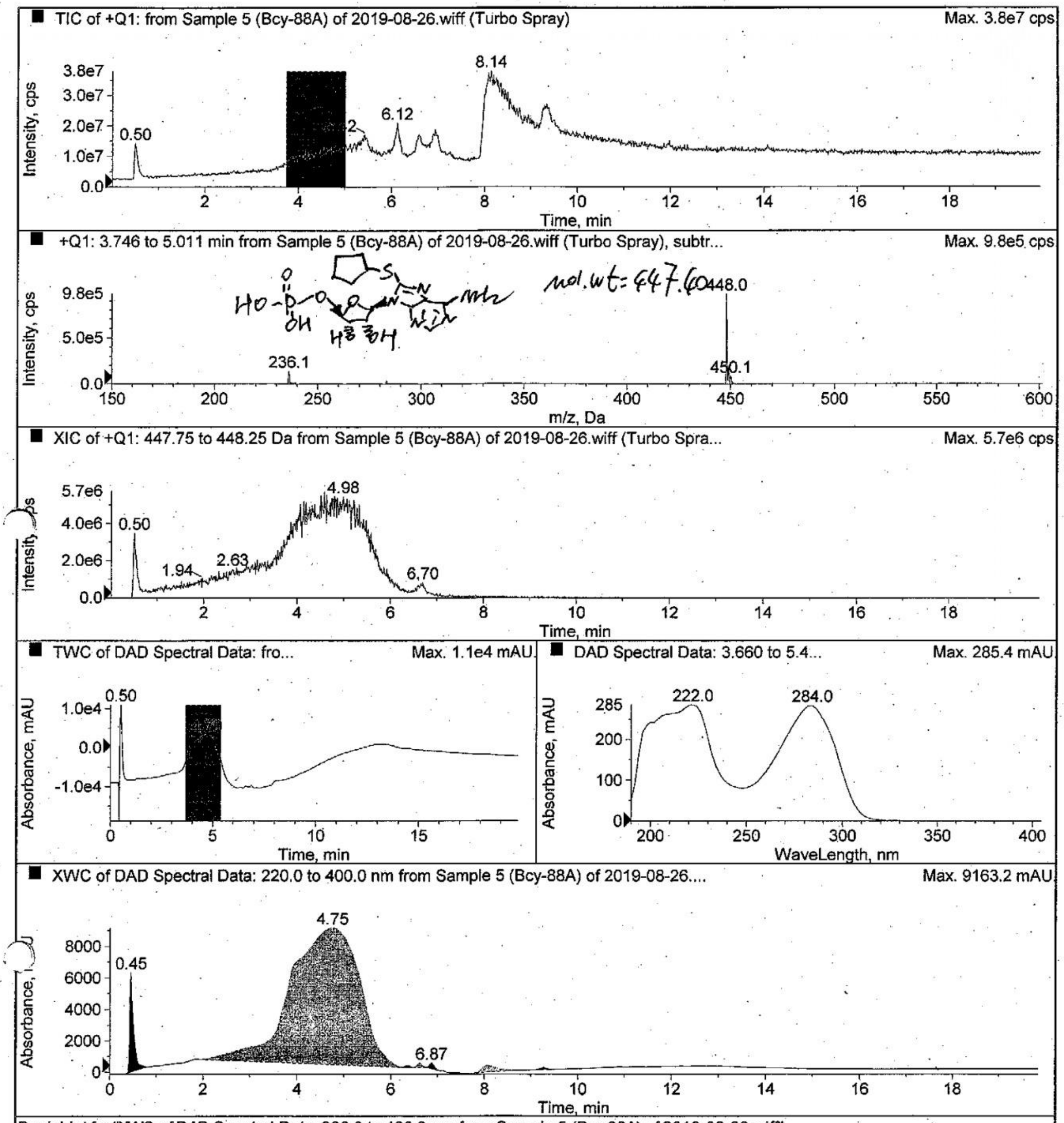

Peak List for "XWC of DAD Spectral Data: 220.0 to 400.0 nm from Sample 5 (Bcy-88A) of 2019-08-26.wiff"

	Time (min)	Area (mAU x min)	% Area	Height (mAU)	% Height	Width (min)	Baseline Type
1	0.4521	3.6096e4	3.8286	6318.8559	38.7862	0.4733	Base to Base
2	4.7548	8.9018e5	94.4169	8671.0653	53.2244	4.2867	Base to Base
3	6.3535	913.6885	0.0969	126.8858	0.7788	0.2467	Base to Base
4	6.6218	2091.5360	0.2218	269.0484	1.6515	0.2867	Base to Base
5	6.8725	2507.6768	0.2660	348.3673	2.1383	0.2733	Base to Base
6	8.0625	9895.9371	1.0496	425.7302	2.6132	0.8800	Base to Base
7	9.2795	1133.3965	0.1202	131.5585	0.8075	0.3600	Base to Base

98.2%.

LC-MS spectrum of compound **31i** (continued)

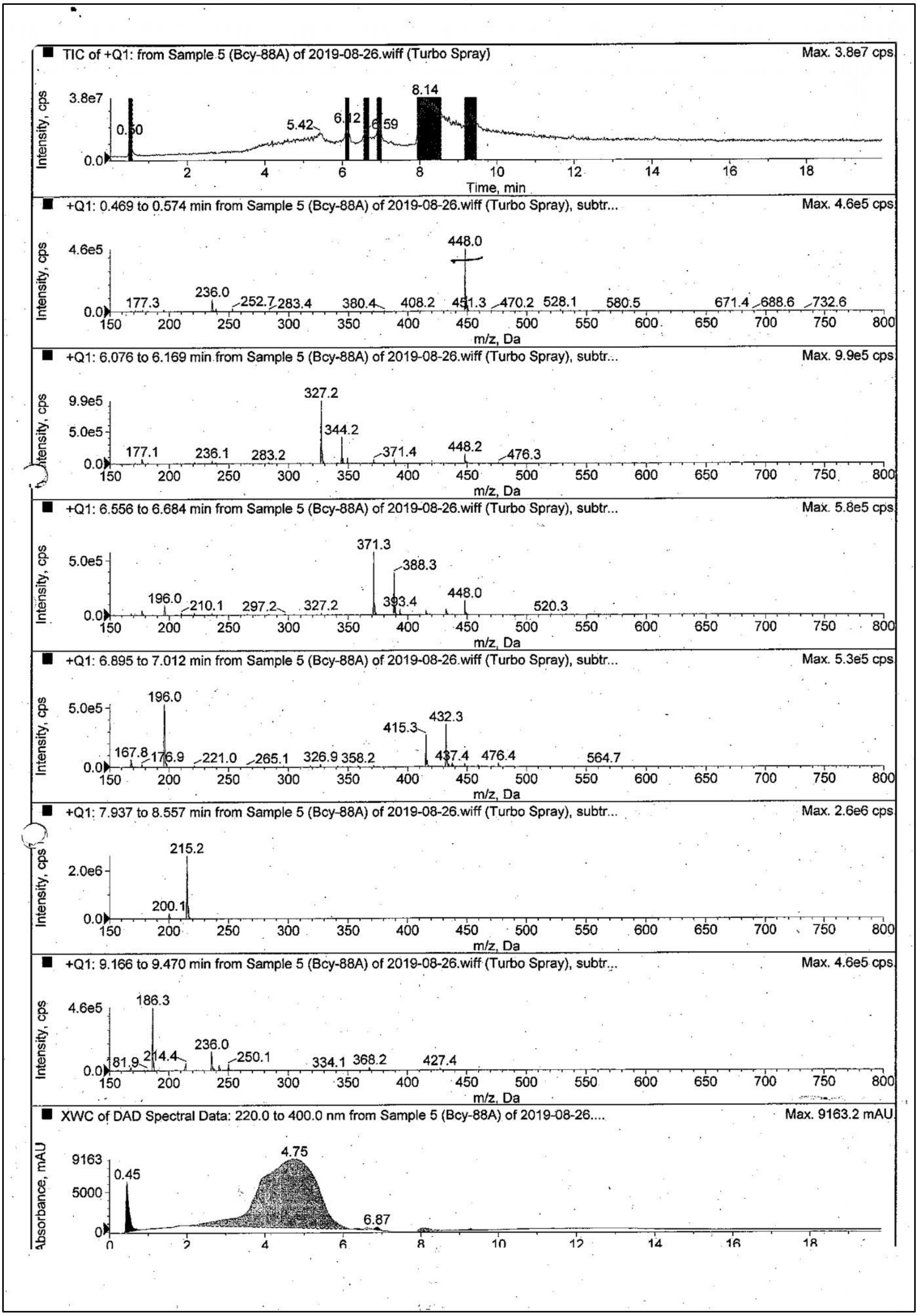

LC-MS spectrum of compound **31j**

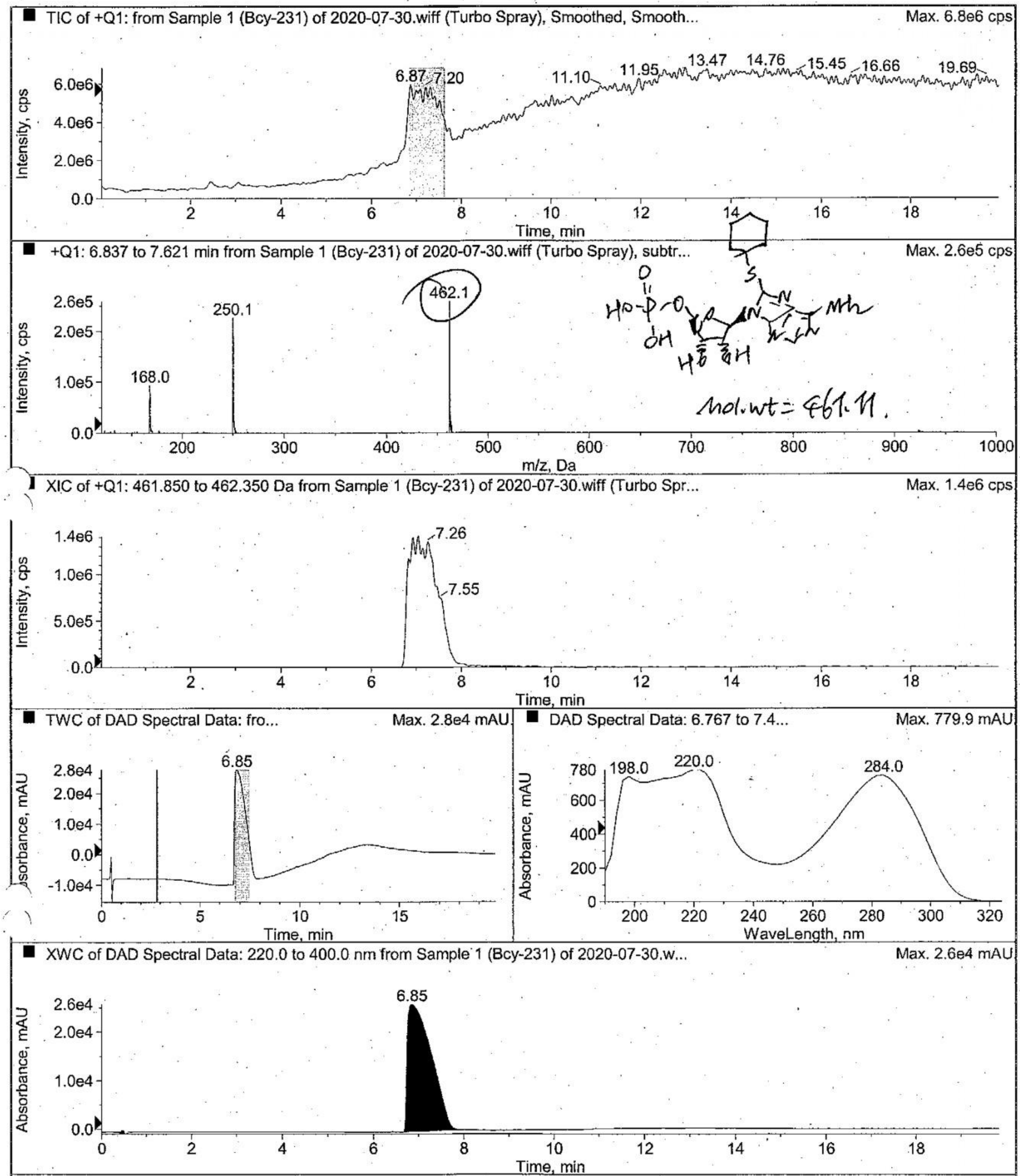

Peak List for "XWC of DAD Spectral Data: 220.0 to 400.0 nm from Sample 1 (Bcy-231) of 2020-07-30.wiff"

	Time (min)	Area (mAU x min)	%Area	Height (mAU)	%Height	Width (min)	Baseline Type
1	0.4812	4546.7186	0.4631	760.3408	2.7802	0.2800	Base to Base
2	0.6589	1390.5976	0.1416	86.5062	0.3163	0.1467	Base to Base
3	6.8522	9.7463e5	99.2769	2.6233e4	95.9223	1.3200	Base to Base
4	8.2744	564.3609	0.0575	134.7576	0.4927	0.1600	Base to Base
5	9.7880	597.6026	0.0609	133.5832	0.4885	0.2867	Base to Base

99.9%.

LC-MS spectrum of compound **31g**

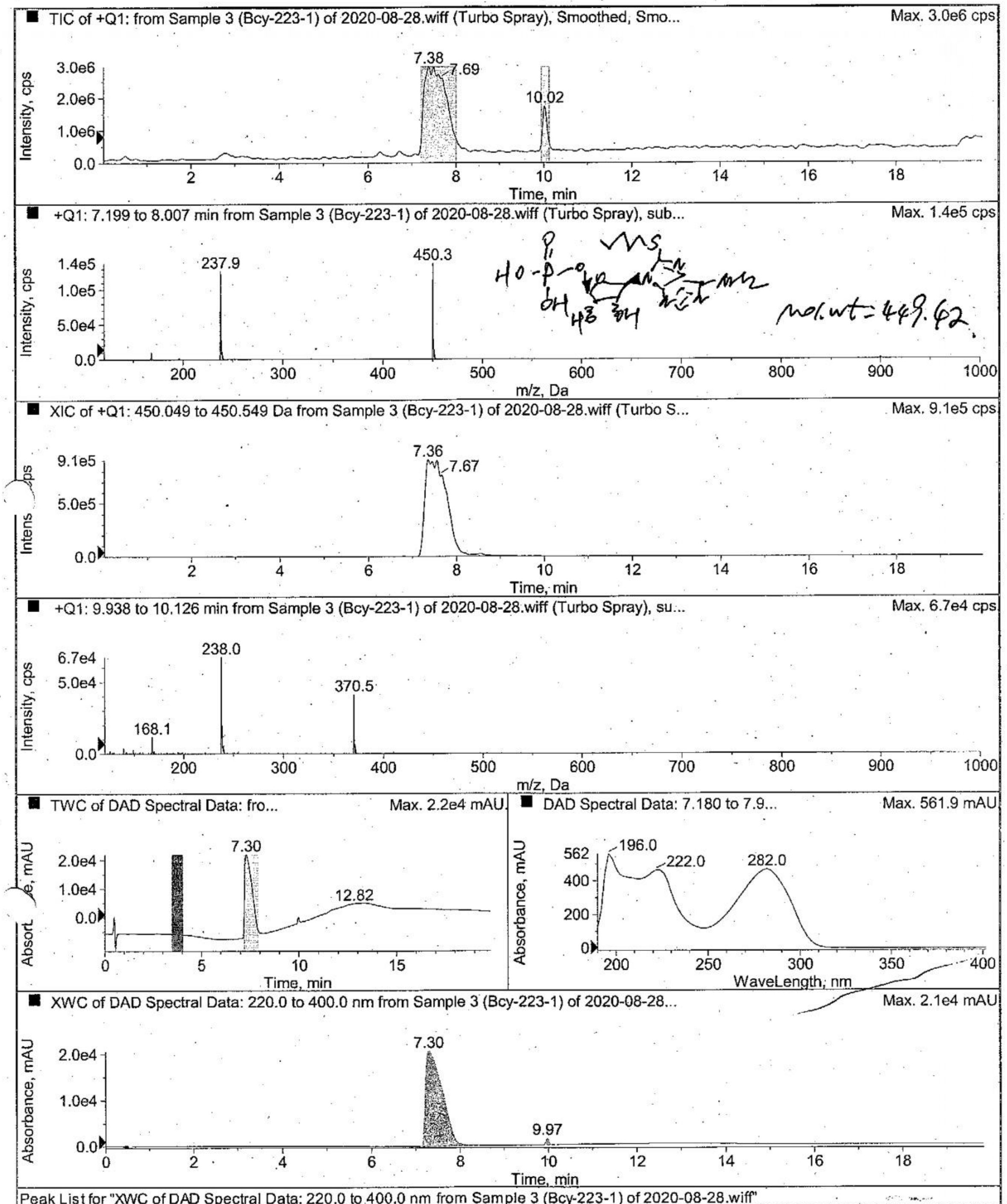

Peak List for "XWC of DAD Spectral Data: 220.0 to 400.0 nm from Sample 3 (Bcy-223-1) of 2020-08-28.wiff"

	Time (min)	Area (mAU x min)	%Area	Height (mAU)	%Height	Width (min)	Baseline Type
1	0.4889	2484.4490	0.4490	537.0505	2.3414	0.1733	Base to Base
2	0.6920	1354.3301	0.2448	73.4203	0.3201	0.1733	Base to Base
3	2.7135	762.2013	0.1378	60.7589	0.2649	0.5067	Base to Base
4	7.2988	5.4032e5	97.6577	2.0680e4	90.1623	1.4000	Base to Base
5	8.5412	599.8368	0.1084	98.3912	0.4290	0.3000	Base to Base
6	9.9744	7758.4528	1.4023	1486.8515	6.4824	0.3267	Base to Base

98.48%.

LC-MS spectrum of compound **33e**

**Sequence Name:**	2021-02-22		
**Data file:**	Bcy.96A1.dx	**Project Name:**	Single Quad
**Sample name:**	Bcy.96A1	**Operator :**	SYSTEM
**Instrument:**	Single Quad	**Acquired on:**	2021-02-22 13:18:33+01:00
**Inj. volume:**	8.000	**Location:**	P1-B3
**Acq. method:**	0_wasser-meoh_standard_100-1000_AB(API2000).amx		
**Processing method:**	MS_standard_plot.pmx		

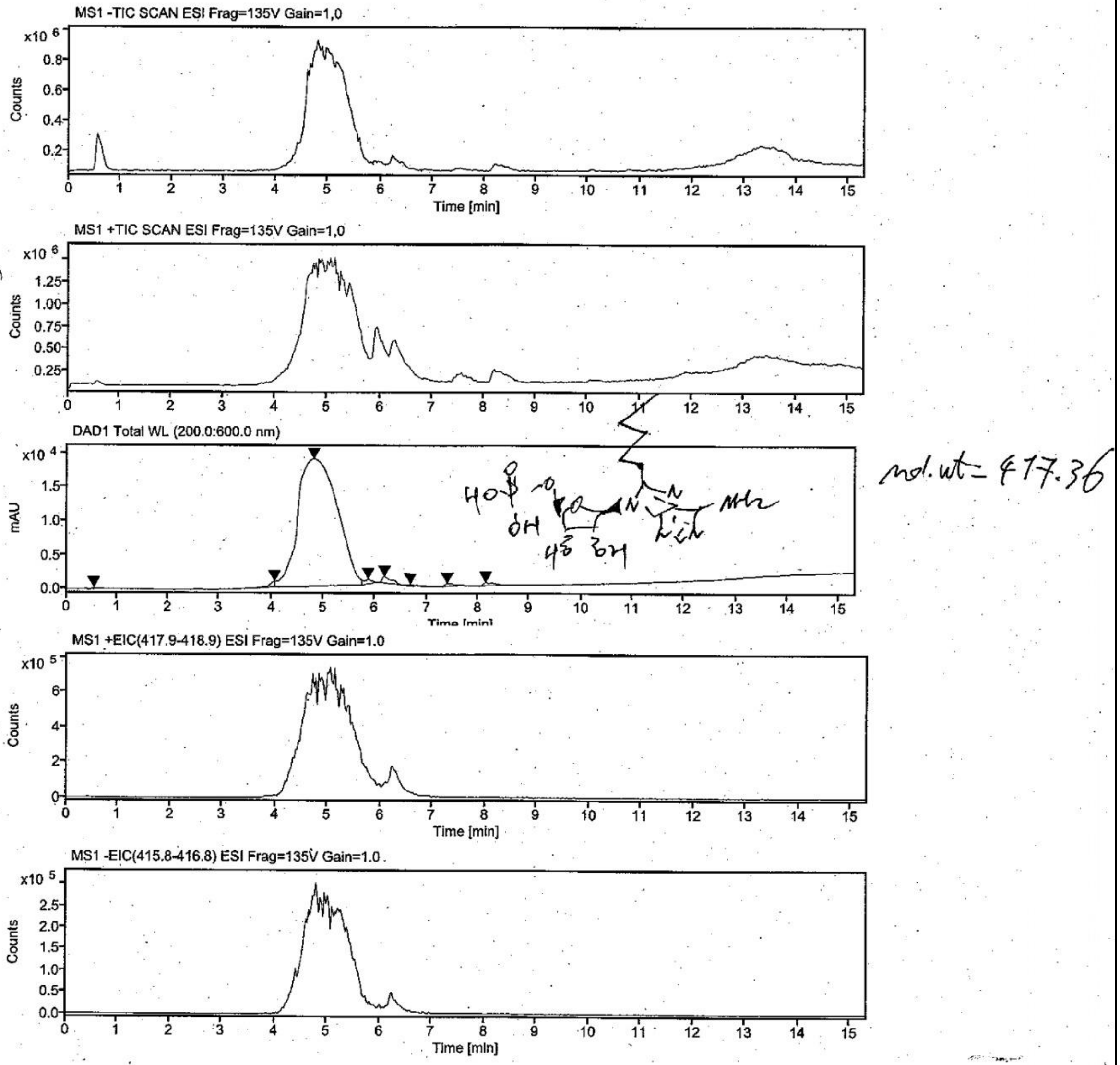

D:\CDSProjects\Default\0-standardplot+EIC.rdl [Rev. 200] **Printed: 2021-02-23 07:23:37+01:00** **Page 1 of 3**

LC-MS spectrum of compound **33e** (continued)

gnal: DAD1 Total WL (200.0:600.0 nm)

RT [min]	Peak MS Base Peak m/z	Area	Area%	Max Peak%	Height
0.547		1351.9327	0.1318	0.137	166.653
4.056		5266.0088	0.5132	0.533	781.775
4.815	833.400	987658.7173	96.2543	100.000	18680.548
5.867		7817.7324	0.7619	0.792	671.263
6.181		11719.6763	1.1422	1.187	901.822
6.683		1737.6050	0.1693	0.176	102.297
7.399		3859.1149	0.3761	0.391	345.653
8.147		6682.2118	0.6512	0.677	443.706
	Sum	1026092.9992			

Signal: MS1 +TIC SCAN ESI Frag=135V Gain=1,0

RT [min]	Peak MS Base Peak m/z	Area	Area%	Max Peak%	Height
5.138	418.200	83151663.8467	100.0000	100.000	1351793.362
	Sum	83151663.8467			

Signal: MS1 -TIC SCAN ESI Frag=135V Gain=1,0

RT [min]	Peak MS Base Peak m/z	Area	Area%	Max Peak%	Height
4.800	833.400	42761329.6205	100.0000	100.000	859540.526
	Sum	42761329.6205			

D:\CDSProjects\Default\0-standardplot+EIC.rdl [Rev. 200] Printed: 2021-02-23 07:23:37+01:00 Page 2 of 3

LC-MS spectrum of compound **33e** (continued)

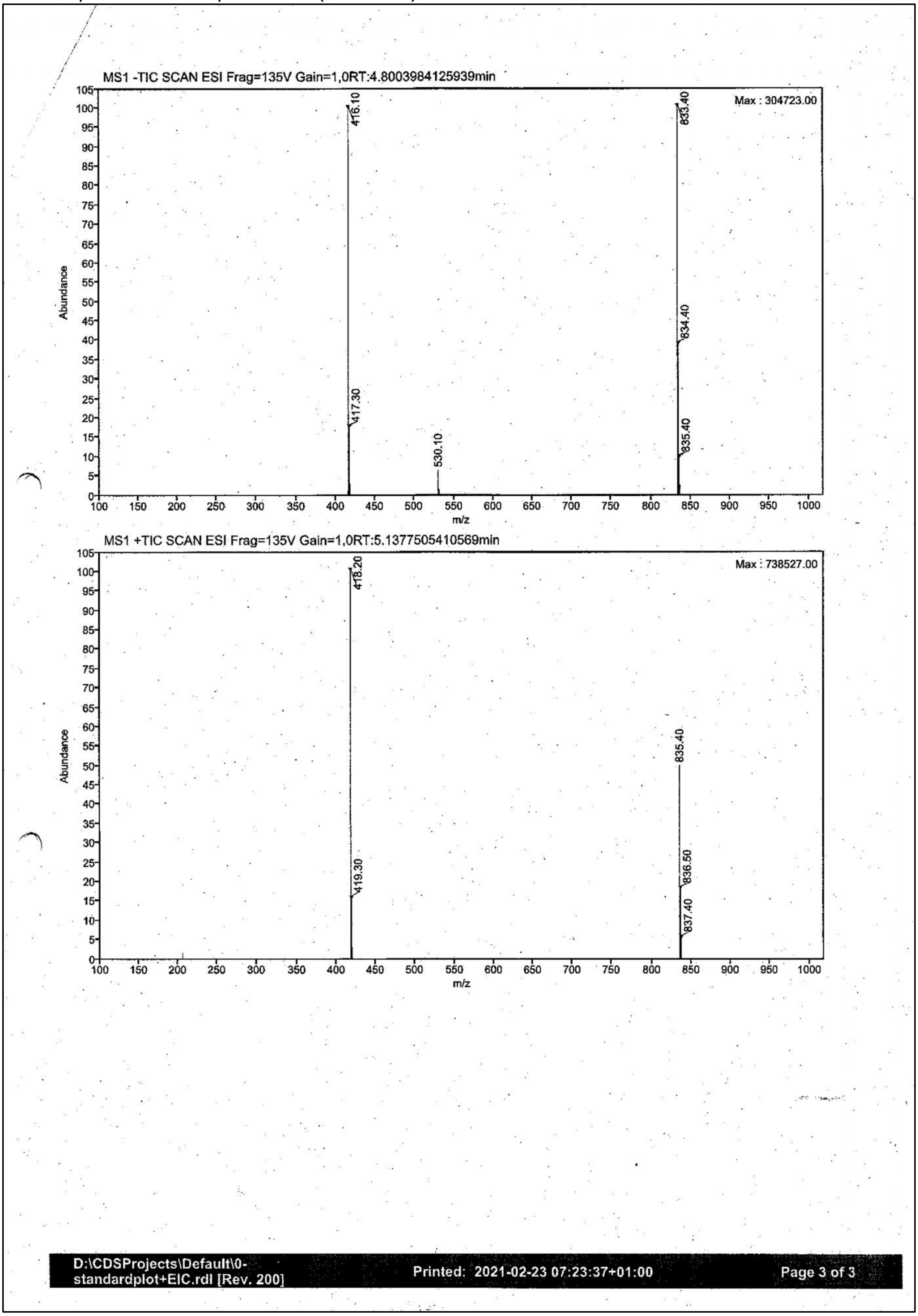

LC-MS spectrum of compound **33f**

**Sequence Name:**	2020-10-14		
**Data file:**	2020-10-14 02-09-37+02-00.dx	**Project Name:**	Single Quad
**Sample name:**	Bcy-230-1A	**Operator :**	SYSTEM
**Instrument:**	Single Quad	**Acquired on:**	2020-10-14 02:10:28+02:00
**Inj. volume:**	2.000	**Location:**	P2-D5
**Acq. method:**	0_wasser-meoh_standard_100-1000_AB(API2000).amx		
**Processing method:**	MS_standard_plot.pmx		

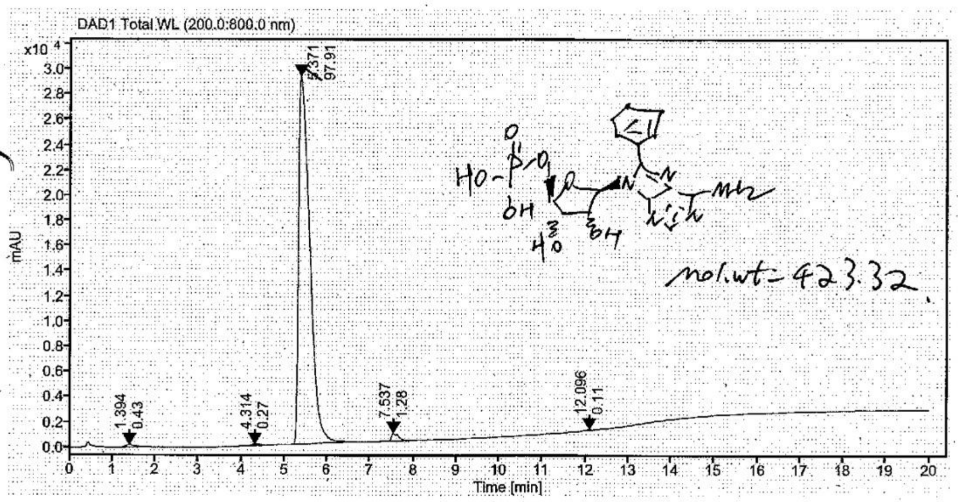

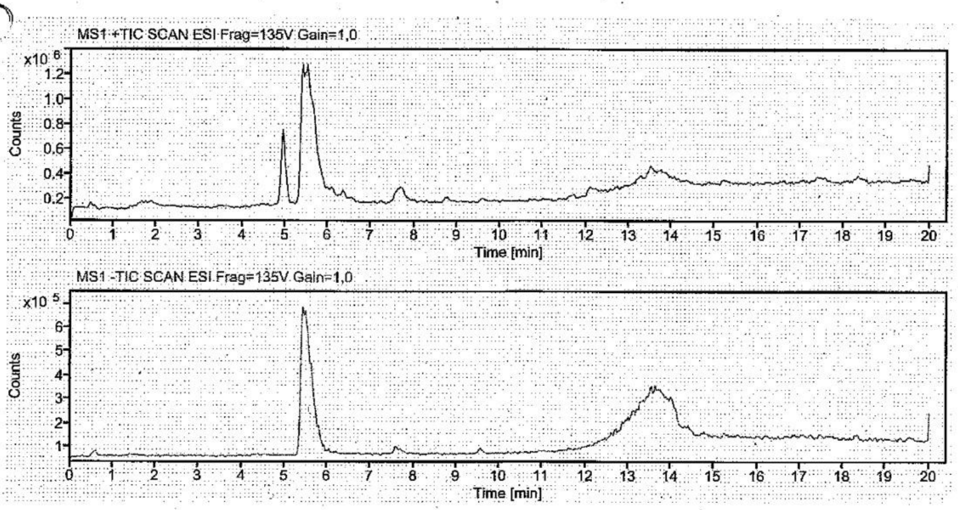

LC-MS spectrum of compound **33f** (continued)

Signal: DAD1 Total WL (200.0:800.0 nm)

RT [min]	Peak MS Base Peak m/z	Type	Width [min]	Col0	Area	Height	Area%
1.394		MM m	0.5650		2318.0437	177.5734	0.4313
4.314		MM m	0.6659		1448.2919	100.9436	0.2695
5.371		MM m	1.3722		526246.9046	29213.2487	97.9111
7.537		MM m	0.5852		6865.8149	610.9589	1.2774
12.096		MM m	0.3027		594.8928	56.7073	0.1107
				Sum	537473.9479		

Signal: MS1 +TIC SCAN ESI Frag=135V Gain=1,0

RT [min]	Peak MS Base Peak m/z	Type	Width [min]	Col0	Area	Height	Area%
5.419	424.200	MM m	0.5045		18170844.5050	958100.9539	100.0000
				Sum	18170844.5050		

Signal: MS1 -TIC SCAN ESI Frag=135V Gain=1,0

RT [min]	Peak MS Base Peak m/z	Type	Width [min]	Col0	Area	Height	Area%
5.433	422.100	MM m	0.5852		9941380.9220	605071.5989	100.0000
				Sum	9941380.9220		

LC-MS spectrum of compound **33f** (continued)

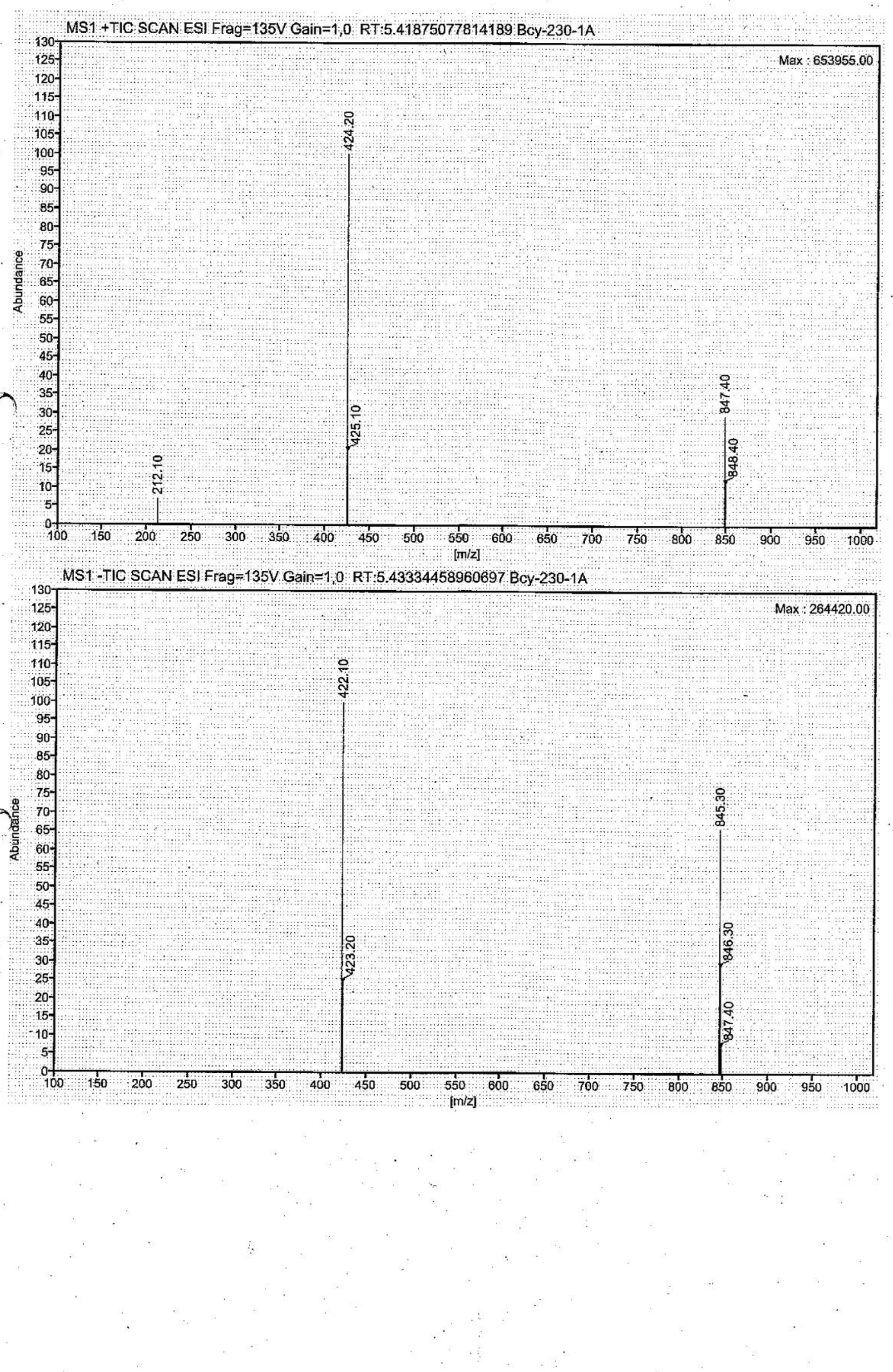

LC-MS spectrum of compound **42a**

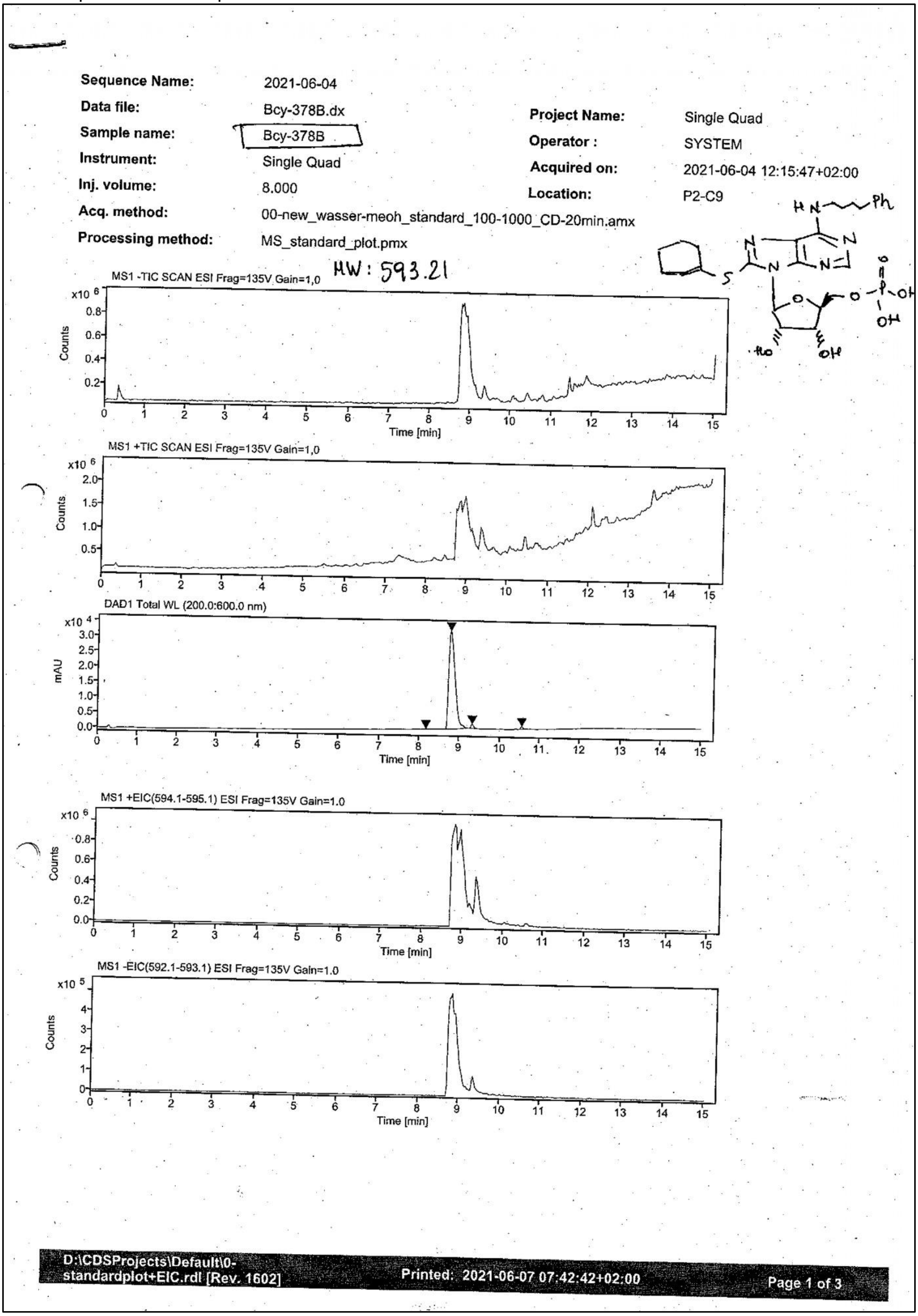

**Sequence Name:**	2021-06-04
**Data file:**	Bcy-378B.dx
**Sample name:**	Bcy-378B
**Instrument:**	Single Quad
**Inj. volume:**	8.000
**Acq. method:**	00-new_wasser-meoh_standard_100-1000_CD-20min.amx
**Processing method:**	MS_standard_plot.pmx
**Project Name:**	Single Quad
**Operator :**	SYSTEM
**Acquired on:**	2021-06-04 12:15:47+02:00
**Location:**	P2-C9

D:\CDSProjects\Default\0-standardplot+EIC.rdl [Rev. 1602] Printed: 2021-06-07 07:42:42+02:00 Page 1 of 3

LC-MS spectrum of compound **42a** (continued)

Signal: DAD1 Total WL (200.0:600.0 nm)

RT [min]	Peak MS Base Peak m/z	Area	Area%	Max Peak%	Height
8.162		212.5077	0.0603	0.062	108.432
8.760		343241.9746	97.3260	100.000	32010.267
9.319		5918.7603	1.6783	1.724	1330.589
10.551		3299.1787	0.9355	0.961	749.936
	Sum	352672.4214			

Signal: MS1 +TIC SCAN ESI Frag=135V Gain=1,0

RT [min]	Peak MS Base Peak m/z	Area	Area%	Max Peak%	Height
8.953	594.400	22398151.6610	90.1153	100.000	1221498.925
9.340	594.300	2456847.3105	9.8847	10.969	415709.332
	Sum	24854998.9715			

Signal: MS1 -TIC SCAN ESI Frag=135V Gain=1,0

RT [min]	Peak MS Base Peak m/z	Area	Area%	Max Peak%	Height
8.821	592.400	11496165.3370	95.4520	100.000	779913.888
9.353	592.300	547759.4926	4.5480	4.765	111476.803
	Sum	12043924.8296			

LC-MS spectrum of compound **42a** (continued)

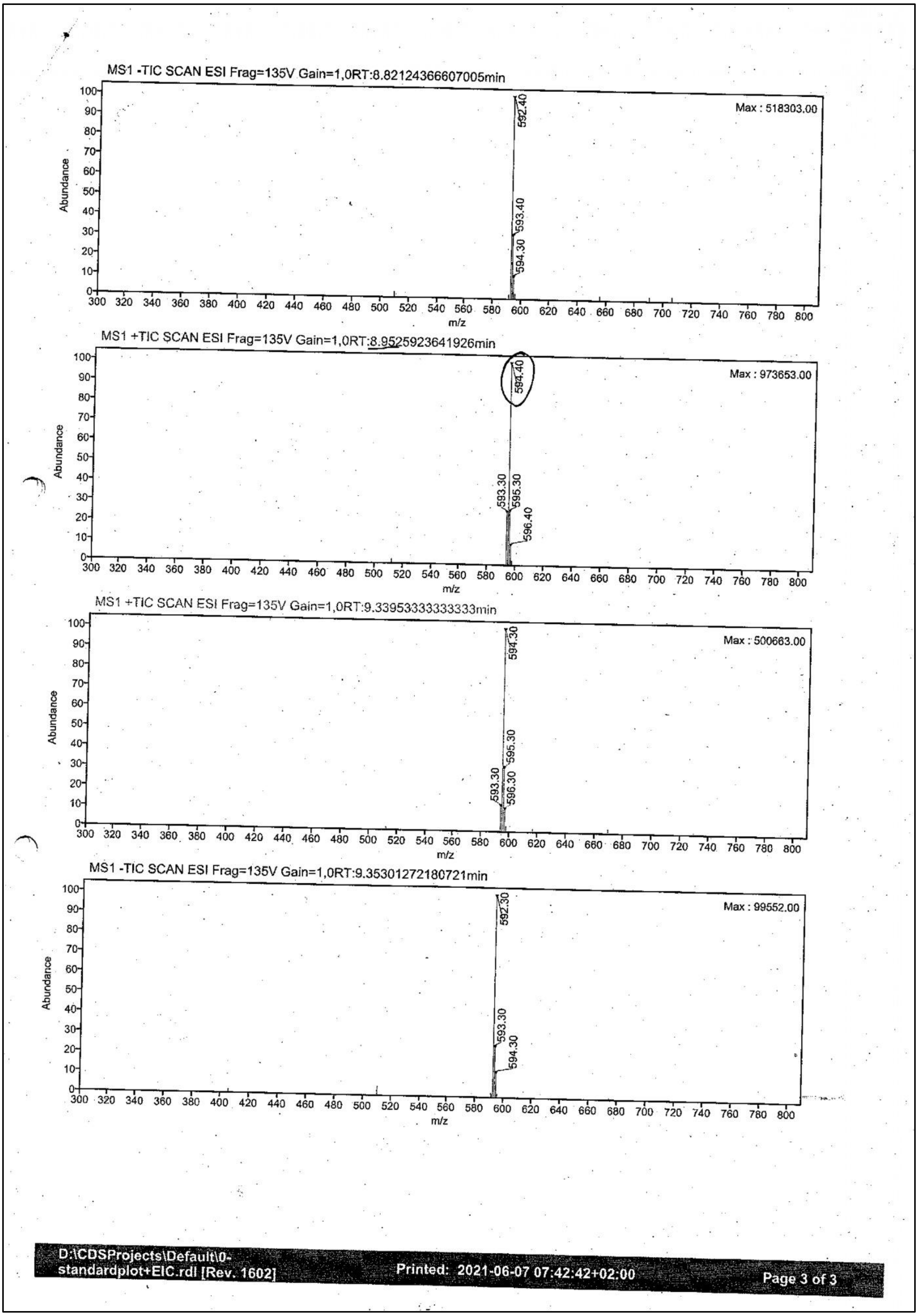

LC-MS spectrum of compound **42b**

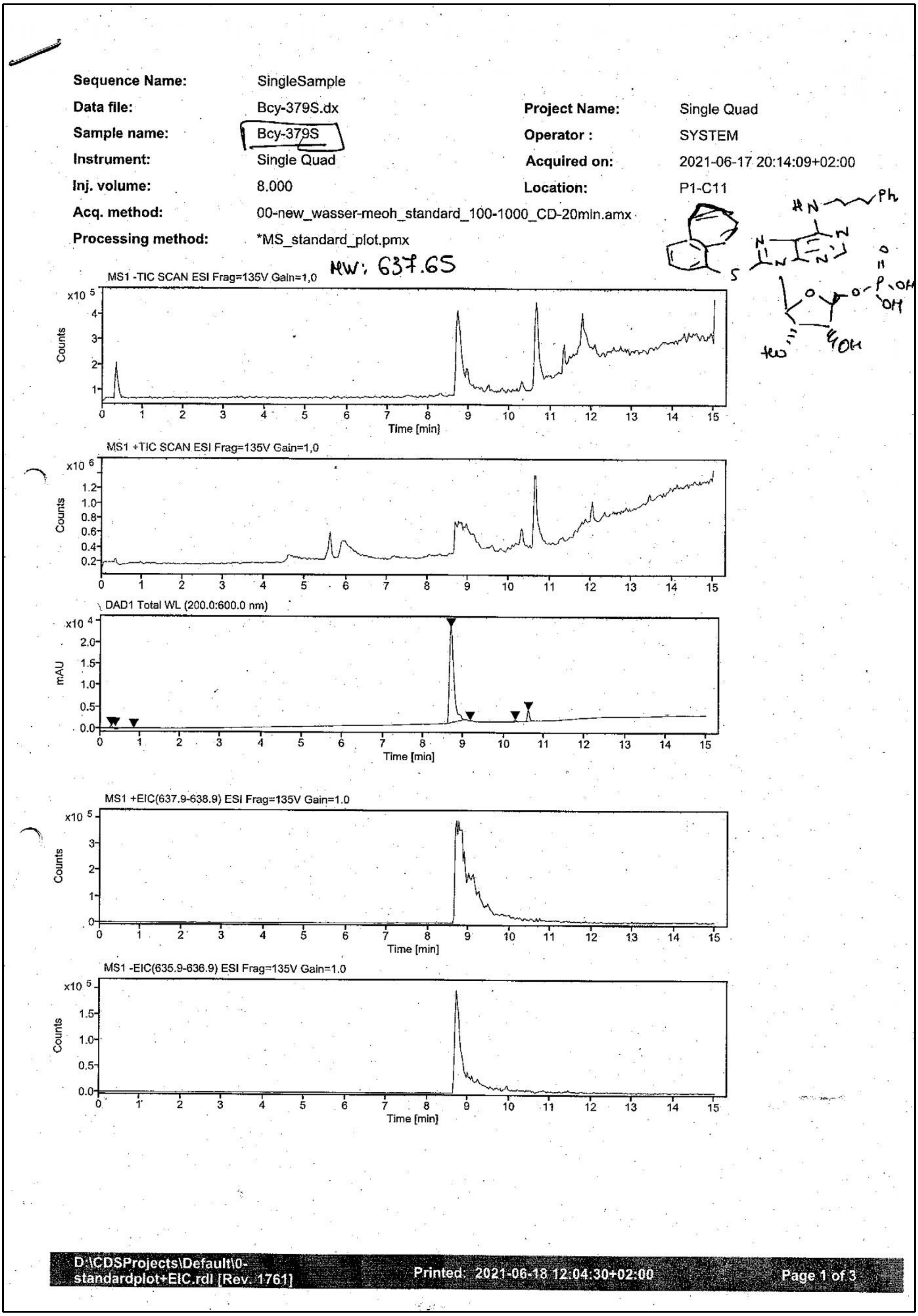

LC-MS spectrum of compound **42b** (continued)

Signal: DAD1 Total WL (200.0:600.0 nm)

RT [min]	Peak MS Base Peak m/z	Area	Area%	Max Peak%	Height
0.282		1190.3547	0.6414	0.695	455.741
0.388		1568.5642	0.8452	0.916	311.963
0.849		420.1834	0.2264	0.245	63.164
8.688	638.200	171234.8146	92.2650	100.000	22312.243
9.166		182.1338	0.0981	0.106	89.607
10.285		1329.4854	0.7164	0.776	378.885
10.616		9664.6866	5.2075	5.644	2533.333
	Sum	185590.2227			

Signal: MS1 +TIC SCAN ESI Frag=135V Gain=1,0

RT [min]	Peak MS Base Peak m/z	Area	Area%	Max Peak%	Height
8.689	638.200	10538617.5951	100.0000	100.000	467918.388
	Sum	10538617.5951			

Signal: MS1 -TIC SCAN ESI Frag=135V Gain=1,0

RT [min]	Peak MS Base Peak m/z	Area	Area%	Max Peak%	Height
8.721	636.200	3779976.7141	100.0000	100.000	328776.139
	Sum	3779976.7141			

LC-MS spectrum of compound **42b** (continued)

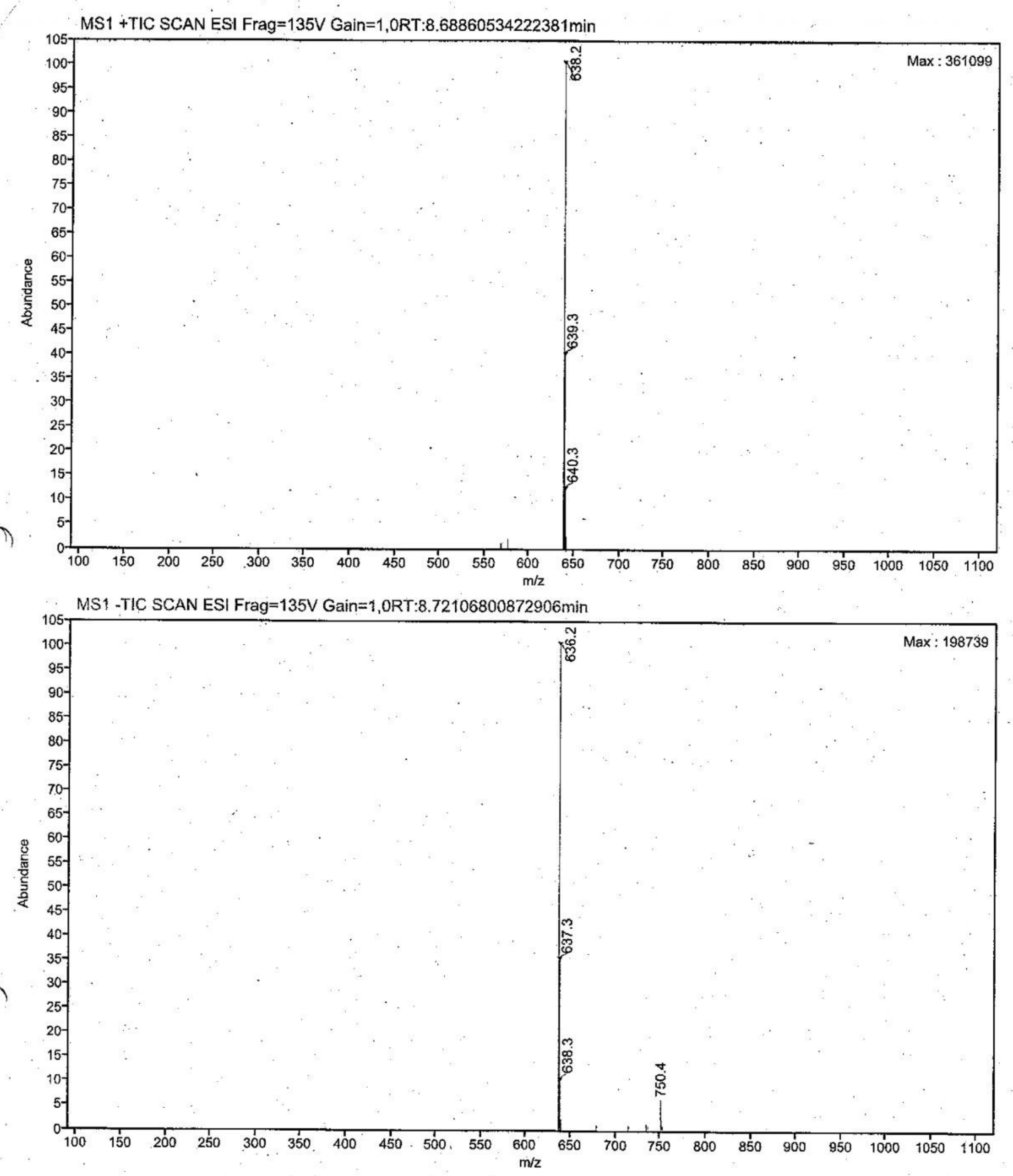

D:\CDSProjects\Default\0-standardplot+EIC.rdl [Rev. 1761] Printed: 2021-06-18 12:04:30+02:00 Page 3 of 3

LC-MS spectrum of compound **42d**

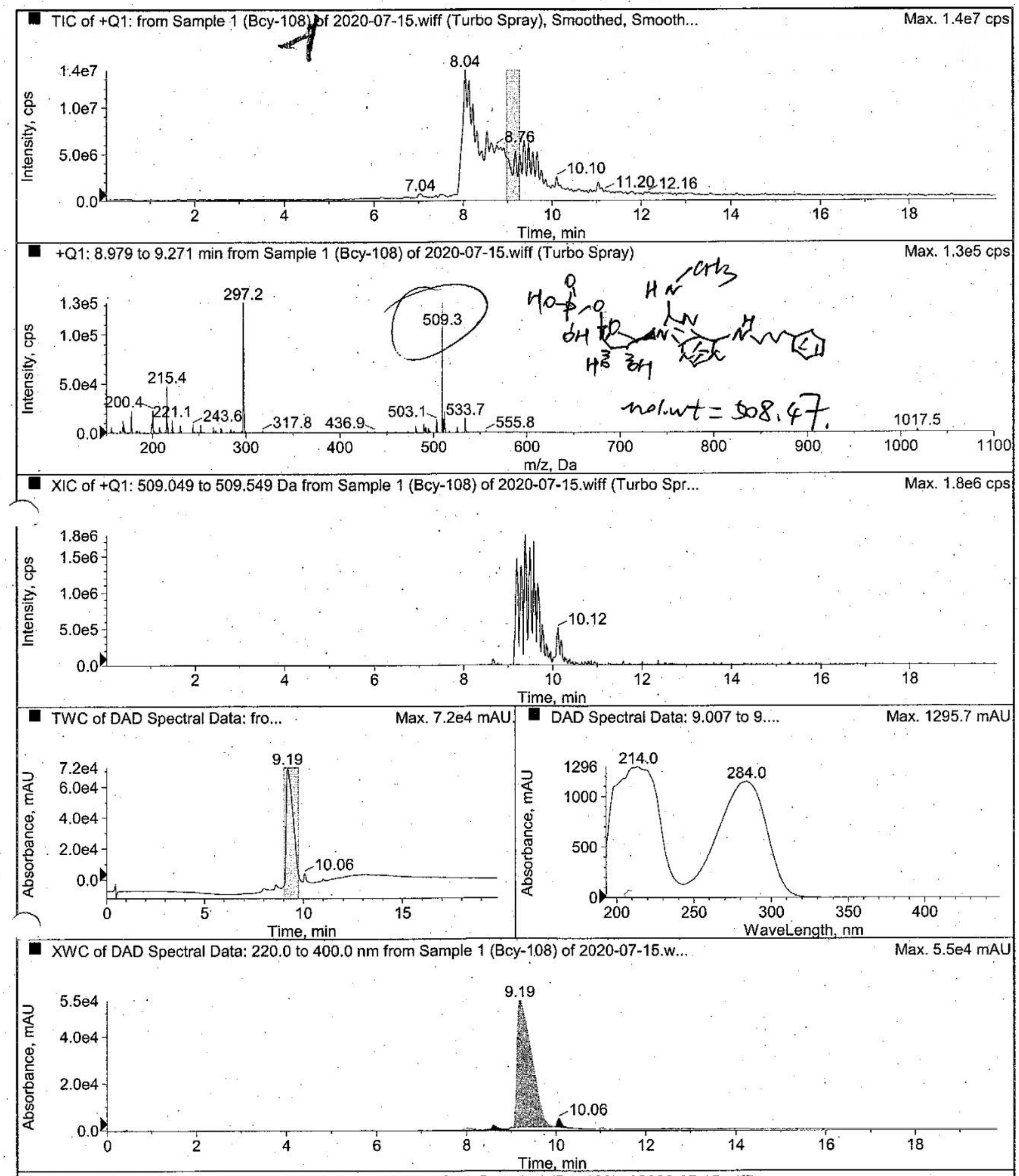

Peak List for "XWC of DAD Spectral Data: 220.0 to 400.0 nm from Sample 1 (Bcy-108) of 2020-07-15.wiff"

	Time (min)	Area (mAU x min)	% Area	Height (mAU)	% Height	Width (min)	Baseline Type
1	0.4385	2906.6780	0.2239	455.1628	0.7306	0.3200	Base to Base
2	8.0139	1.6844e4	1.2977	1034.9723	1.6612	0.6533	Base to Base
3	8.6040	1.5748e4	1.2132	1894.4266	3.0406	0.4467	Base to Base
4	9.1938	1.2314e6	94.8665	5.4346e4	87.2285	0.9333	Base to Base
5	10.0617	2.6495e4	2.0412	3799.1605	6.0978	0.4200	Base to Base
6	11.0004	4638.7259	0.3574	773.3608	1.2413	0.5333	Base to Base

95.1%.

LC-MS spectrum of compound **42e**

**Sequence Name:**	2021-06-01_new		
**Data file:**	Bcy-110-1Y.dx	**Project Name:**	Single Quad
**Sample name:**	Bcy-110-1Y	**Operator :**	SYSTEM
**Instrument:**	Single Quad	**Acquired on:**	2021-06-01 18:45:56+02:00
**Inj. volume:**	3.000	**Location:**	P1-F7
**Acq. method:**	00-new_wasser-meoh_standard_100-1000_CD-20min.amx		
**Processing method:**	MS_standard_plot.pmx		

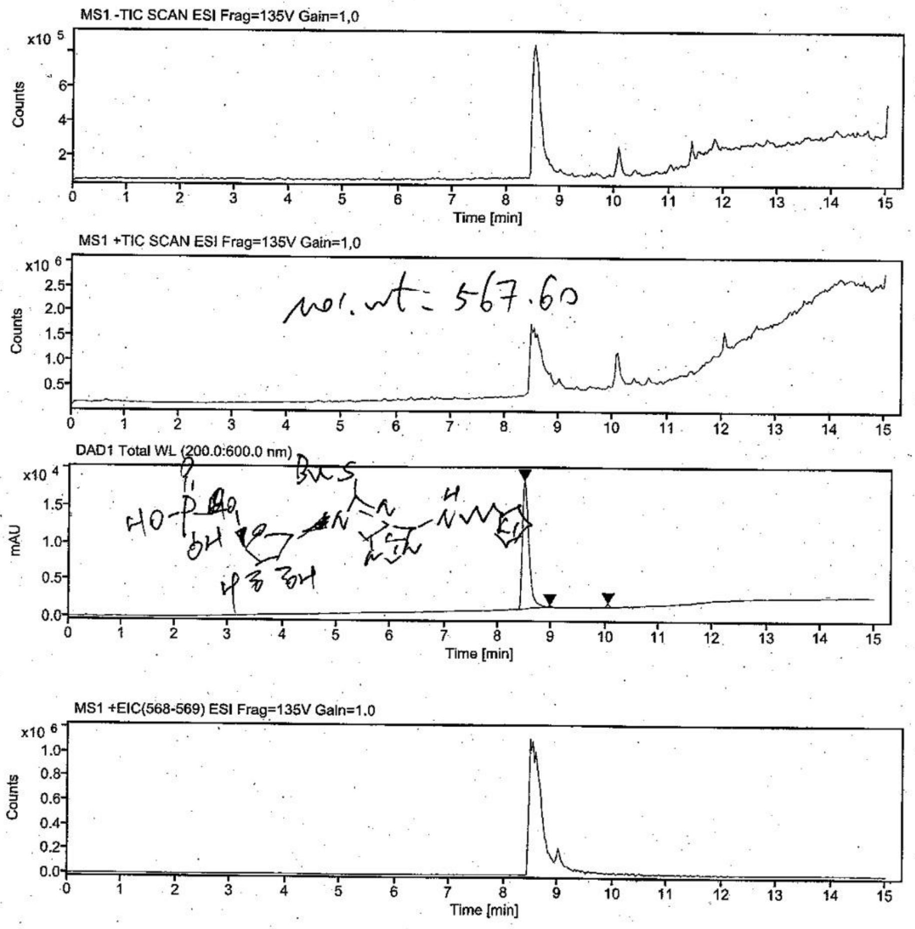

Signal: DAD1 Total WL (200.0:600.0 nm)

RT [min]	Peak MS Base Peak m/z	Area	Area%	Max Peak%	Height
8.479	568.300	144345.8832	98.3109	100.000	17254.523
8.967		579.1297	0.3944	0.401	145.595
10.049		1900.8492	1.2946	1.317	421.085
	Sum	146825.8620			

D:\CDSProjects\Default\0-standardplot+EIC.rdl [Rev. 1566] Printed: 2021-06-02 09:02:36+02:00 Page 1 of 2

LC-MS spectrum of compound **42e** (continued)

Signal: MS1 +TIC SCAN ESI Frag=135V Gain=1,0

RT [min]	Peak MS Base Peak m/z	Area	Area%	Max Peak%	Height
8.484	568.300	11319752.3407	100.0000	100.000	1212332.620
	Sum	11319752.3407			

Signal: MS1 -TIC SCAN ESI Frag=135V Gain=1,0

RT [min]	Peak MS Base Peak m/z	Area	Area%	Max Peak%	Height
8.522	566.300	7048504.0972	100.0000	100.000	732475.844
	Sum	7048504.0972			

MS1 +TIC SCAN ESI Frag=135V Gain=1,0RT:8.48355min

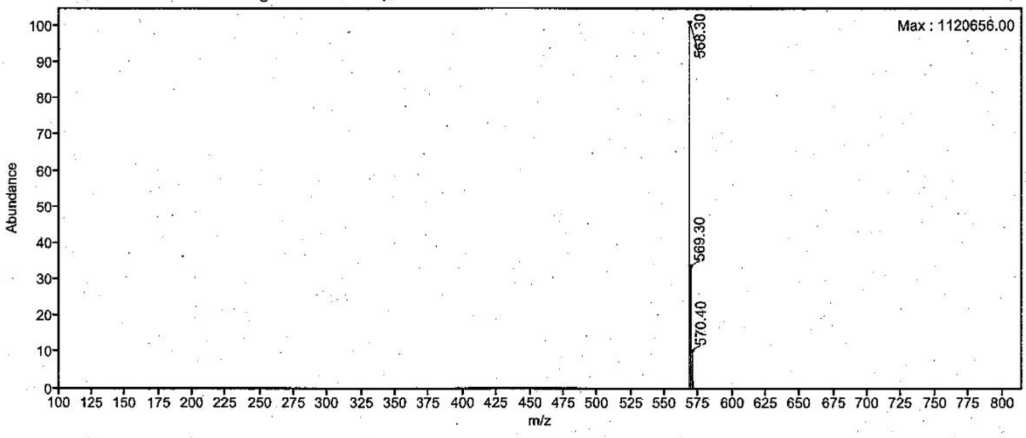

MS1 -TIC SCAN ESI Frag=135V Gain=1,0RT:8.52201949700369min

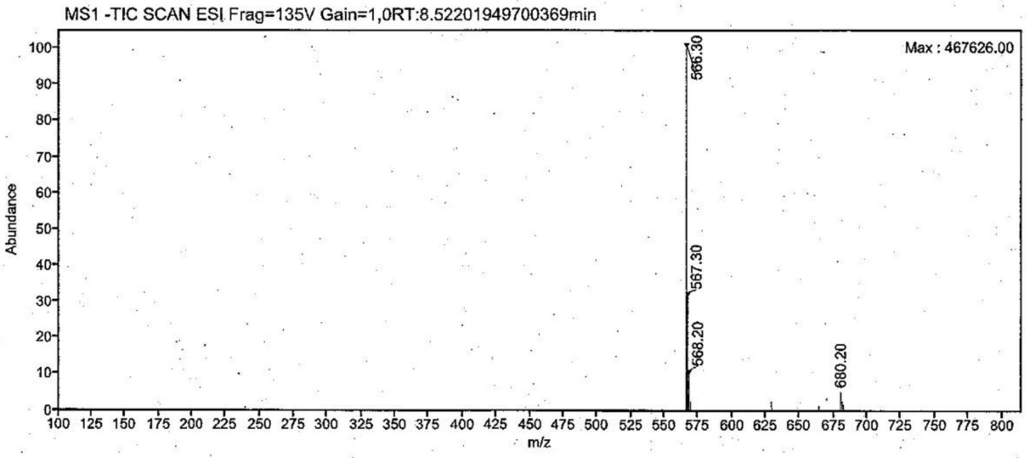

D:\CDSProjects\Default\0-standardplot+EIC.rdl [Rev. 1566] Printed: 2021-06-02 09:02:36+02:00 Page 2 of 2

4 ^{1}H, ^{13}C, and ^{31}P NMR spectra of selected AMP derivatives

^{1}H NMR spectrum of compound **8r**

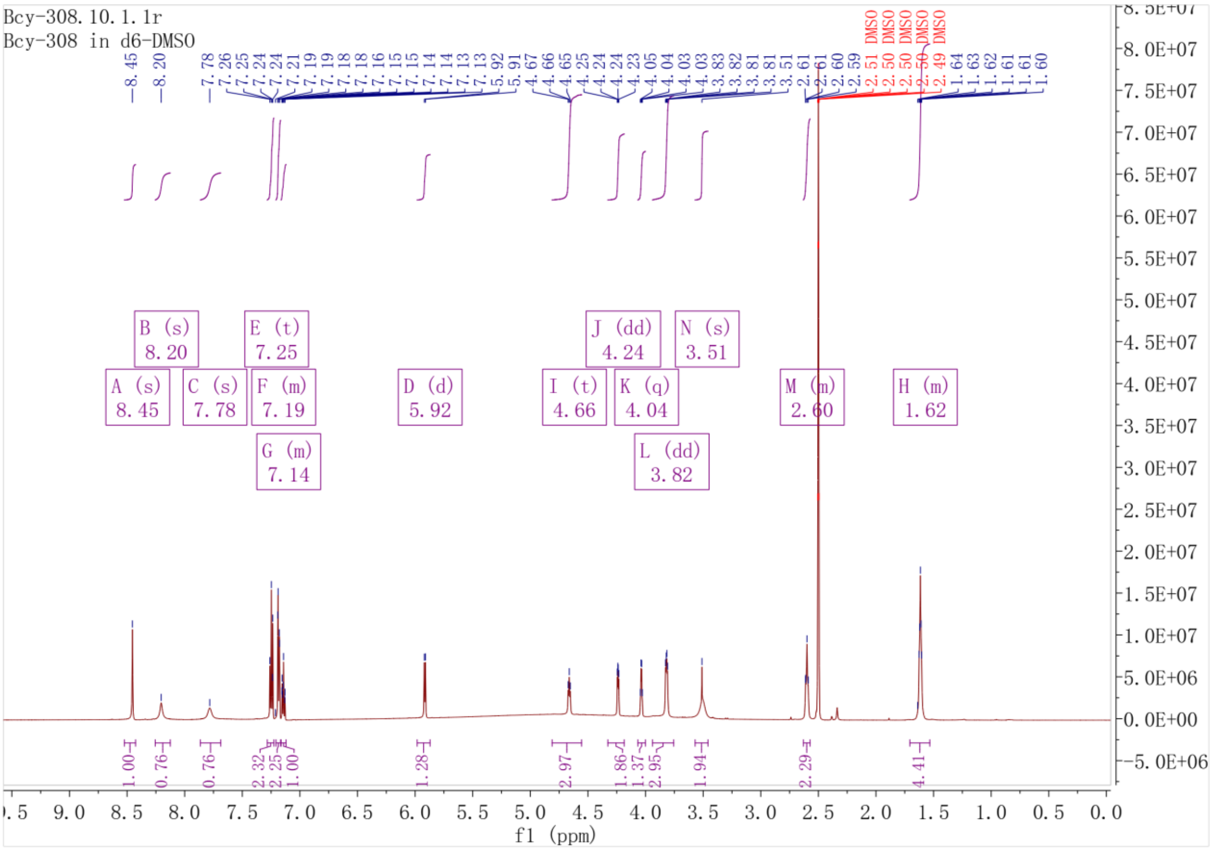

^{13}C NMR spectrum of compound **8r**

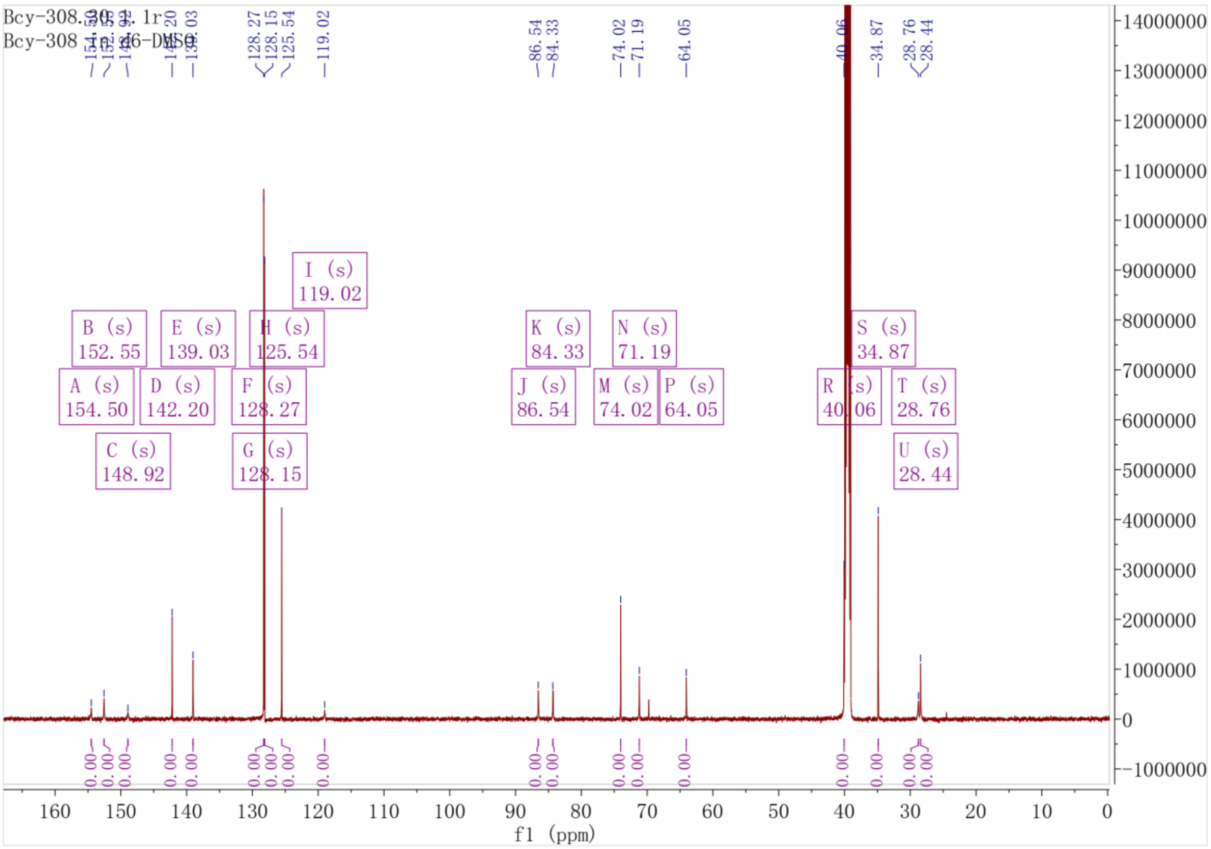

^{31}P NMR spectrum of compound **8r**

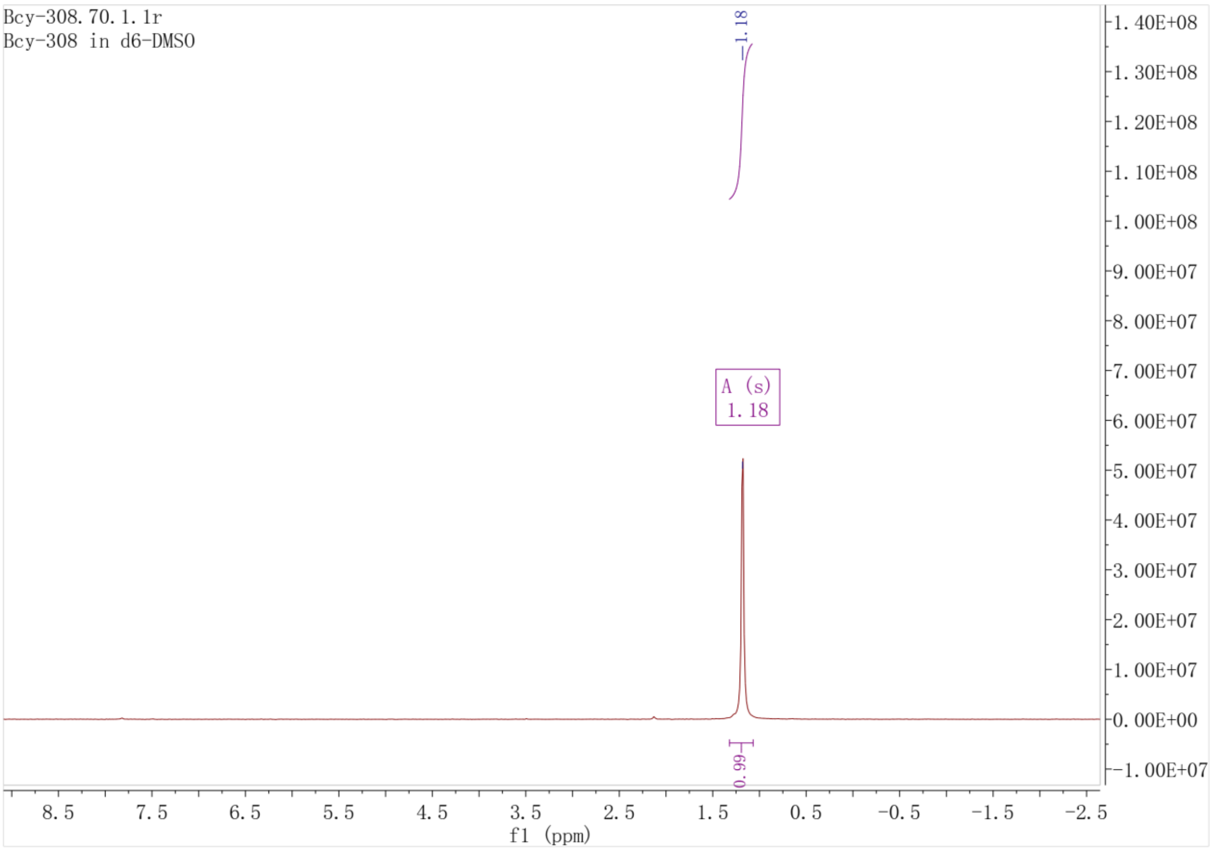

^{1}H NMR spectrum of compound **8s**

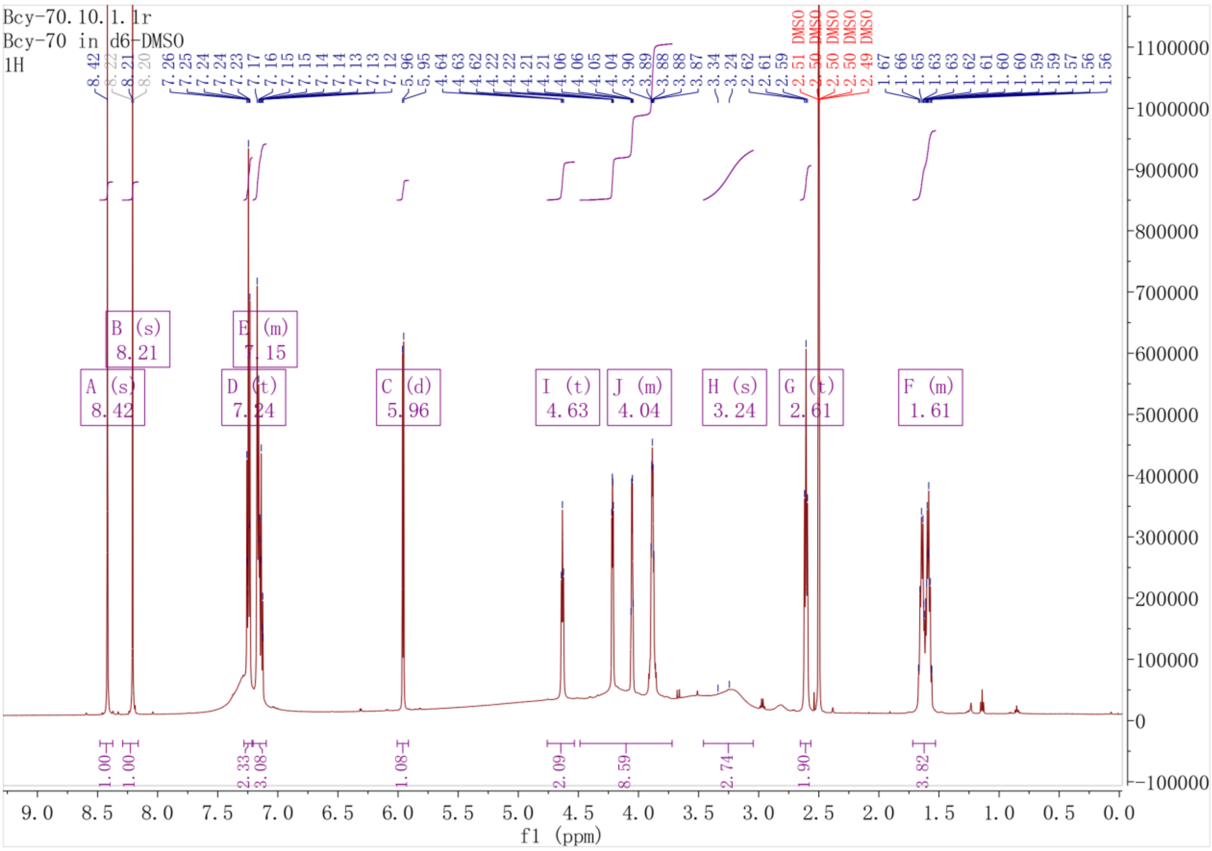

^{13}C NMR spectrum of compound **8s**

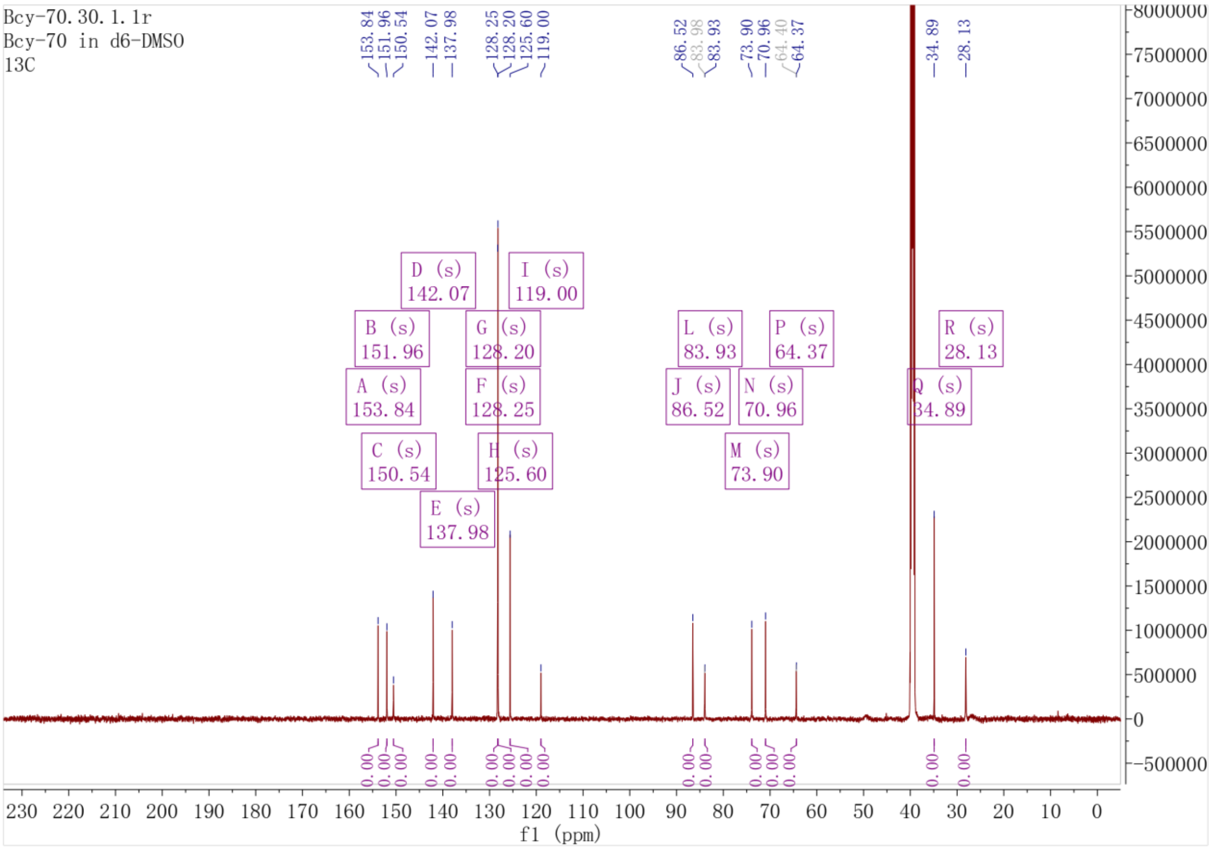

^{31}P NMR spectrum of compound **8s**

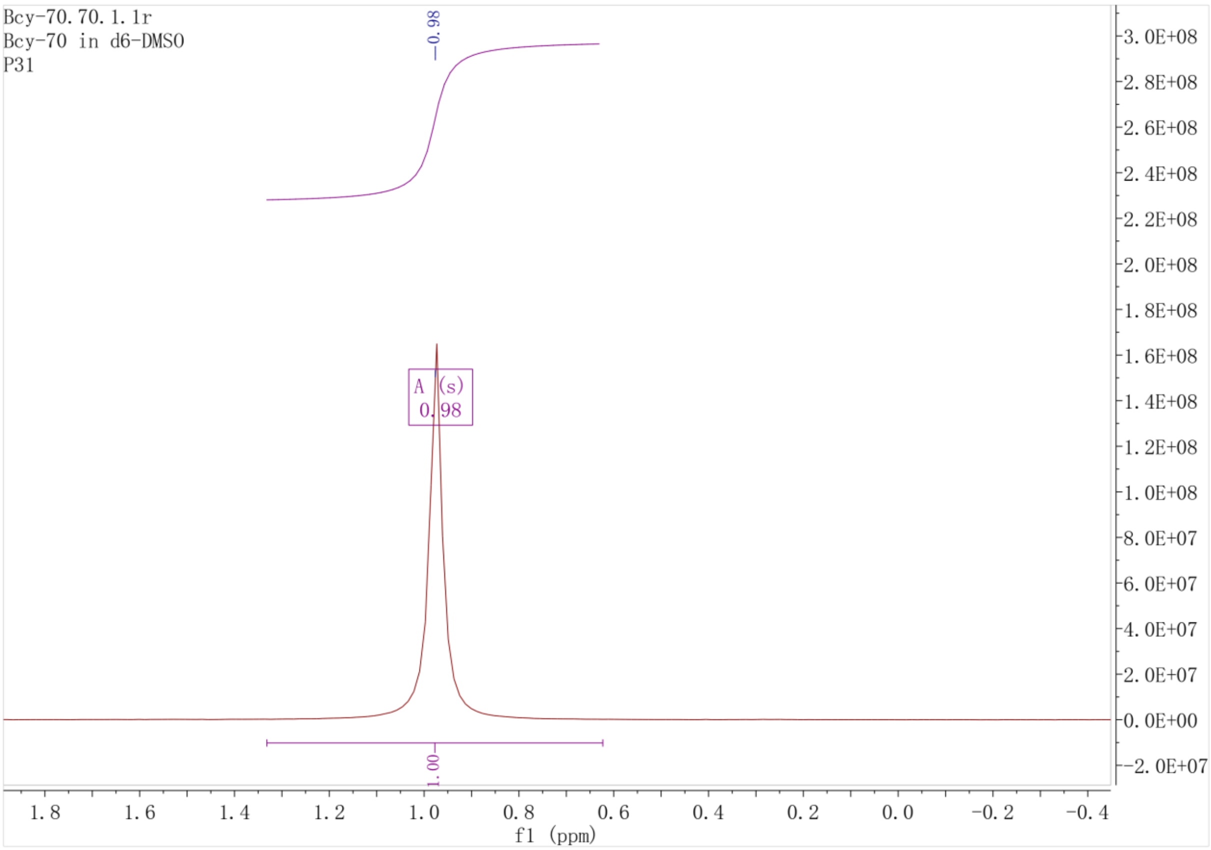

^{1}H NMR spectrum of compound **8v**

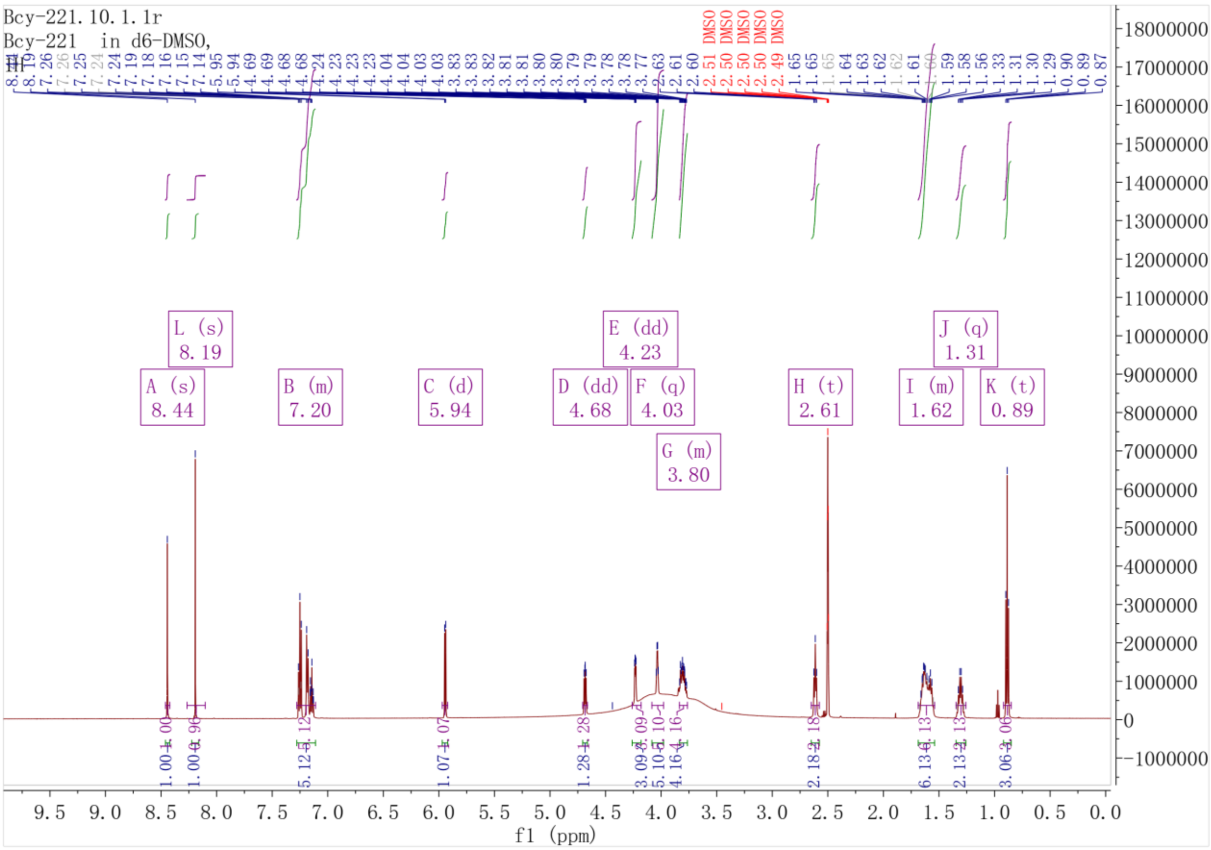

^{13}C NMR spectrum of compound **8v**

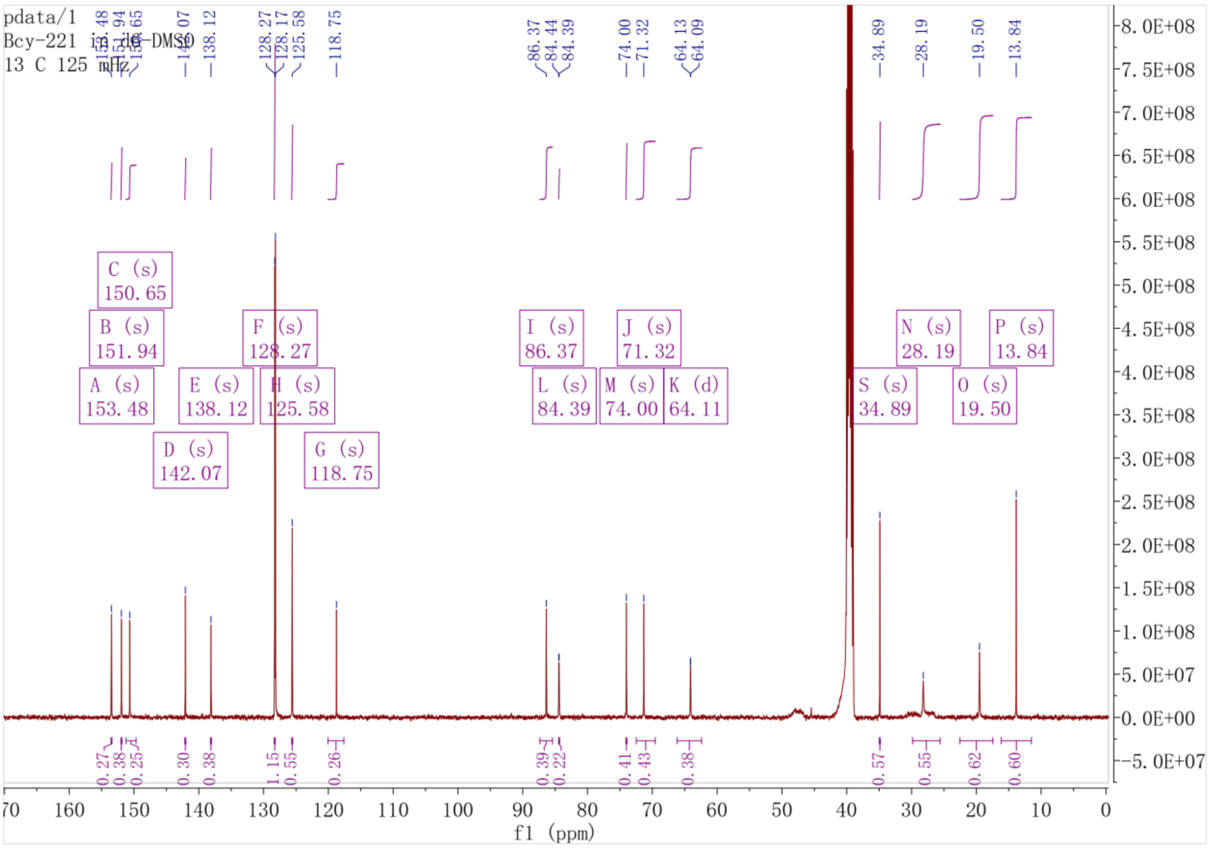

^{31}P NMR spectrum of compound **8v**

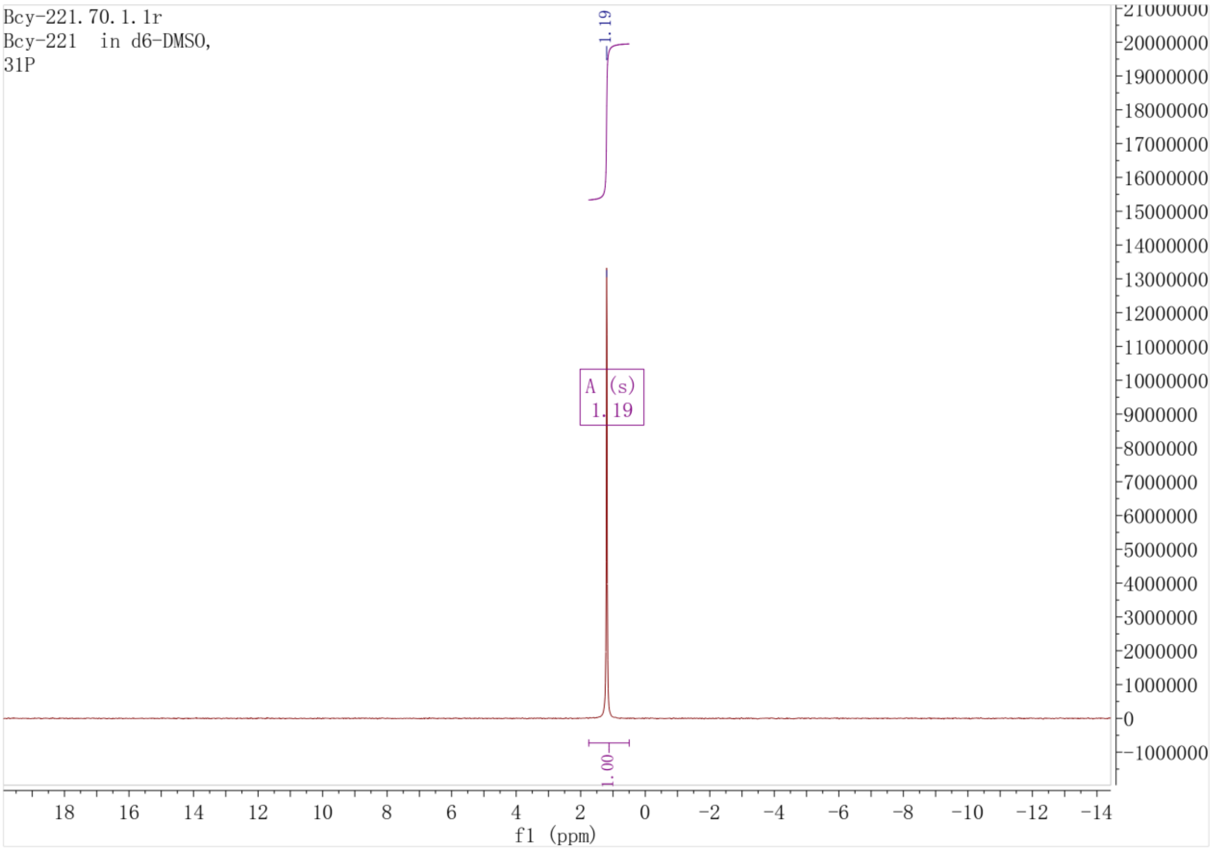

^{1}H NMR spectrum of compound **25a**

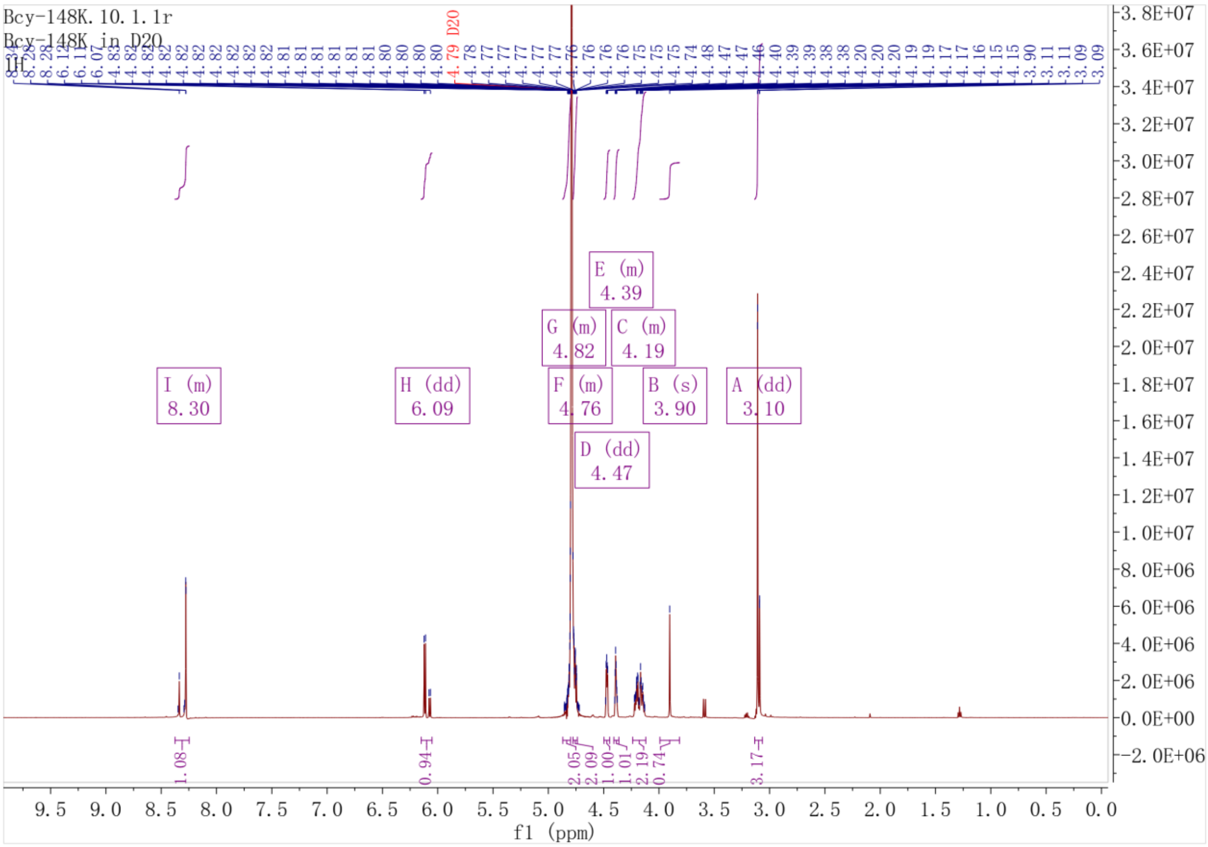

^{13}C NMR spectrum of compound **25a**

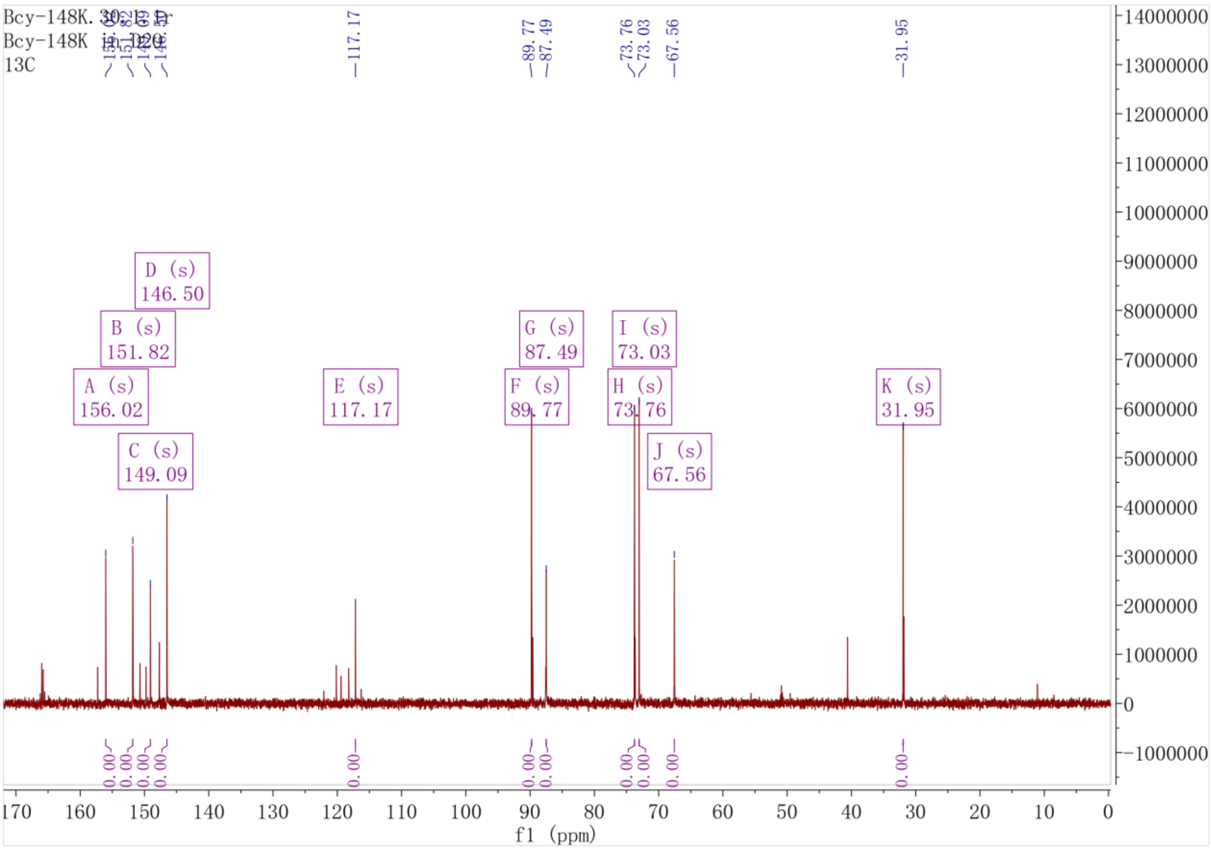

^{31}P NMR spectrum of compound **25a**

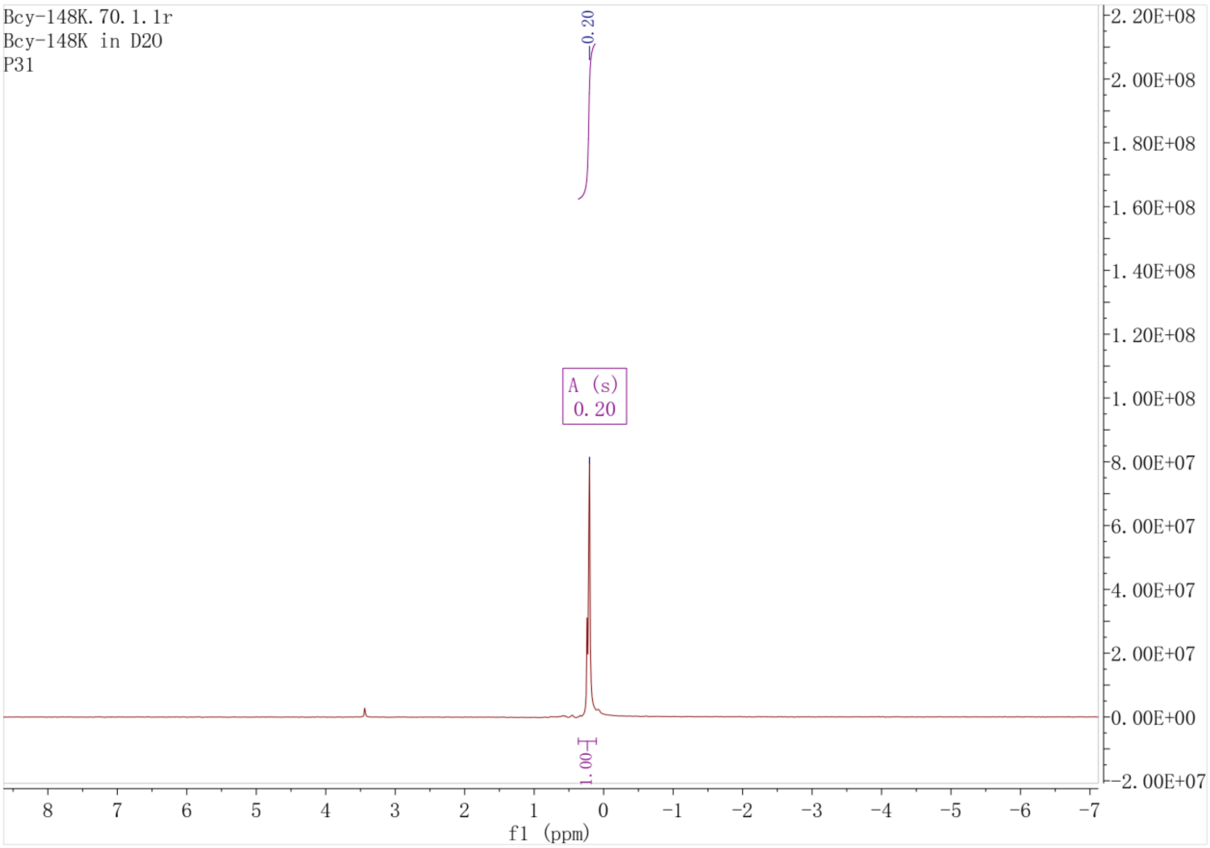

^{1}H NMR spectrum of compound **31d**

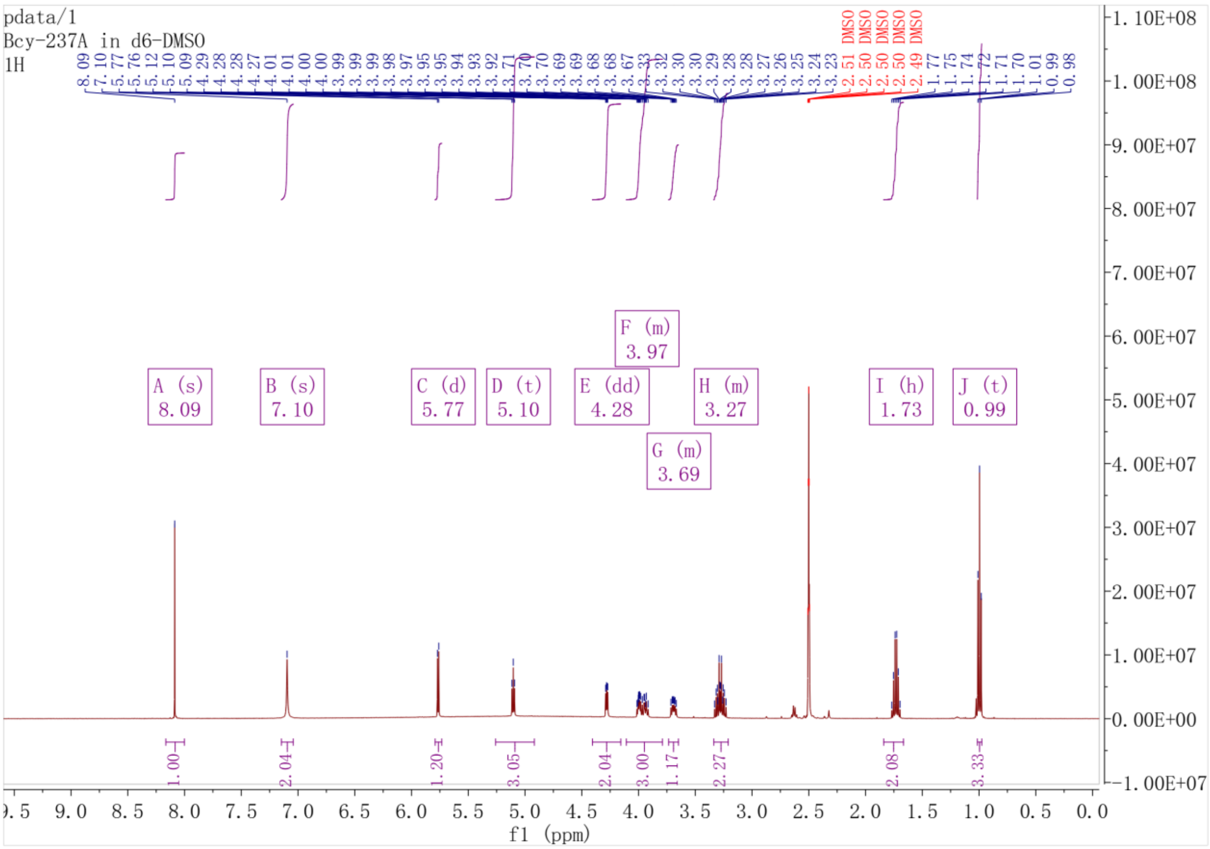

^{13}C NMR spectrum of compound **31d**

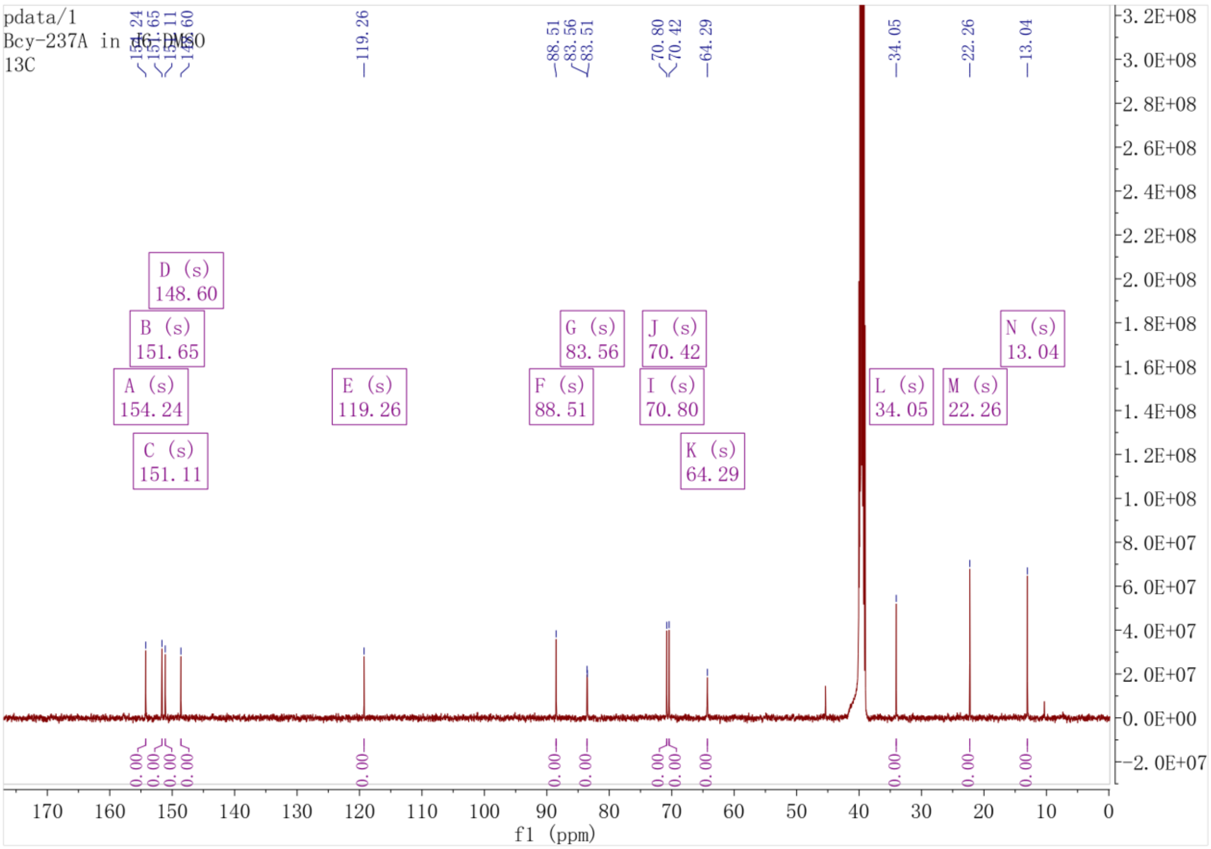

^{31}P NMR spectrum of compound **31d**

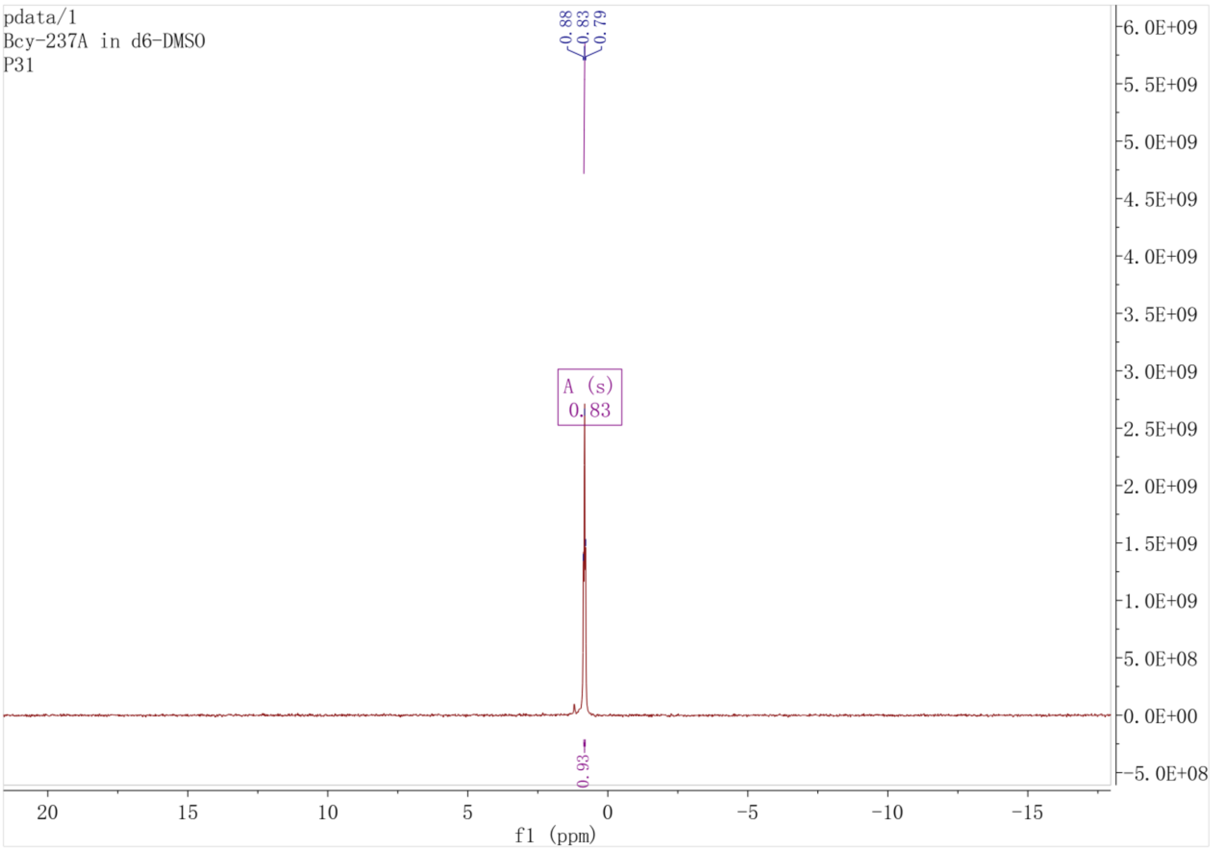

^{1}H NMR spectrum of compound **31g**

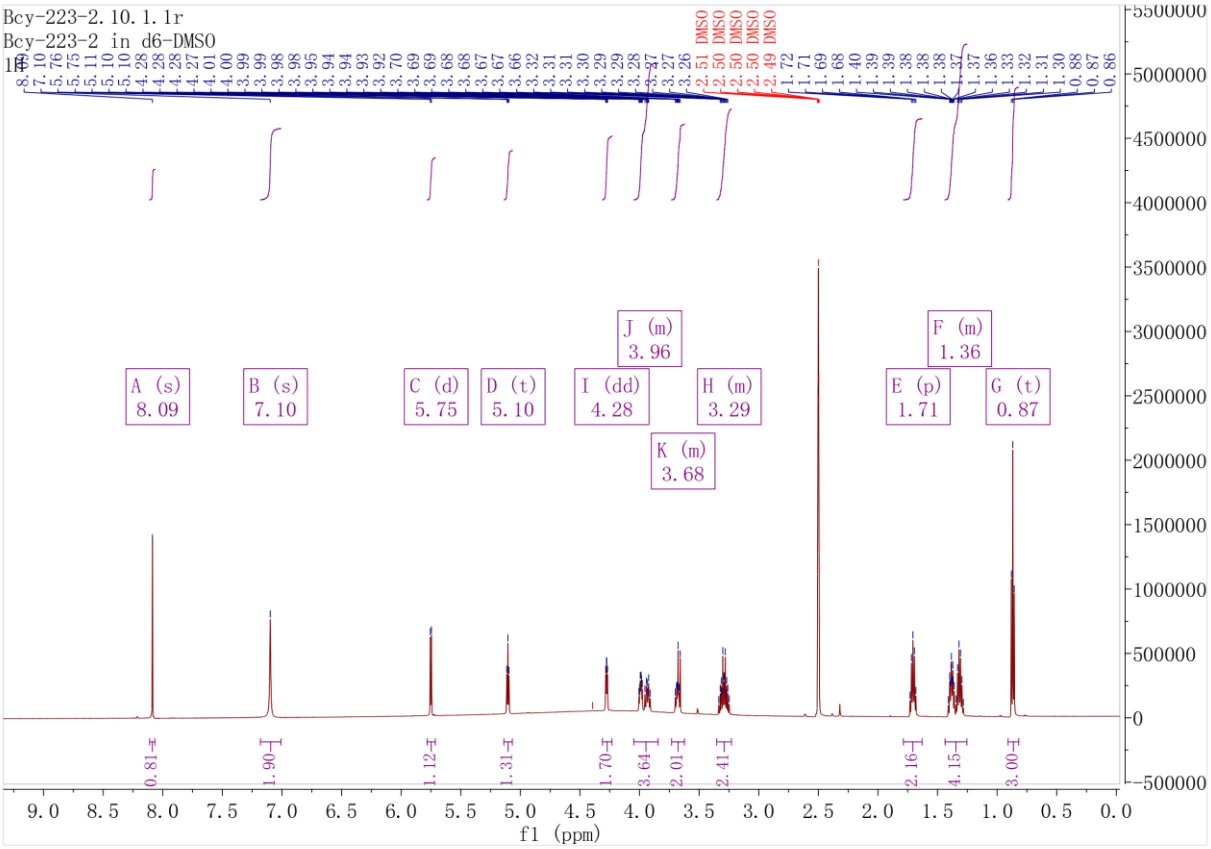

^{13}C NMR spectrum of compound **31g**

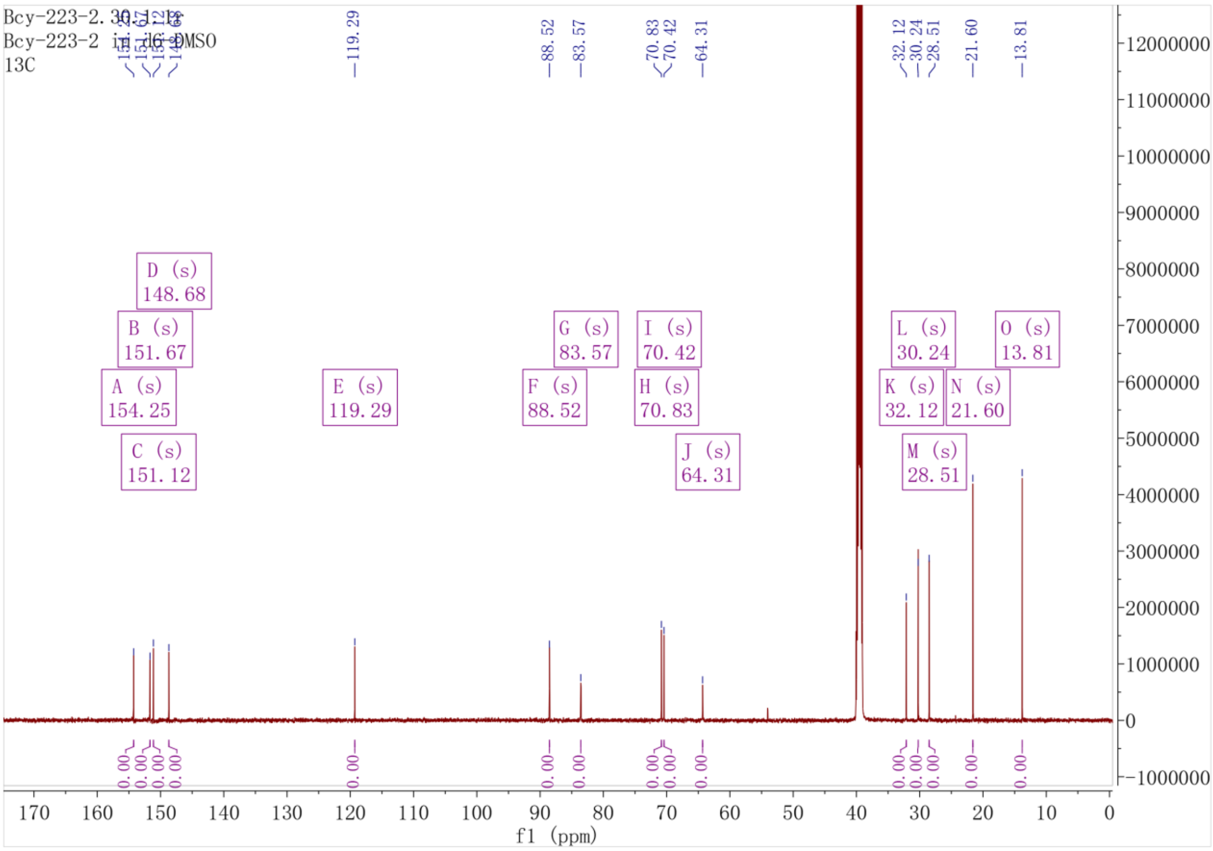

^{31}P NMR spectrum of compound **31g**

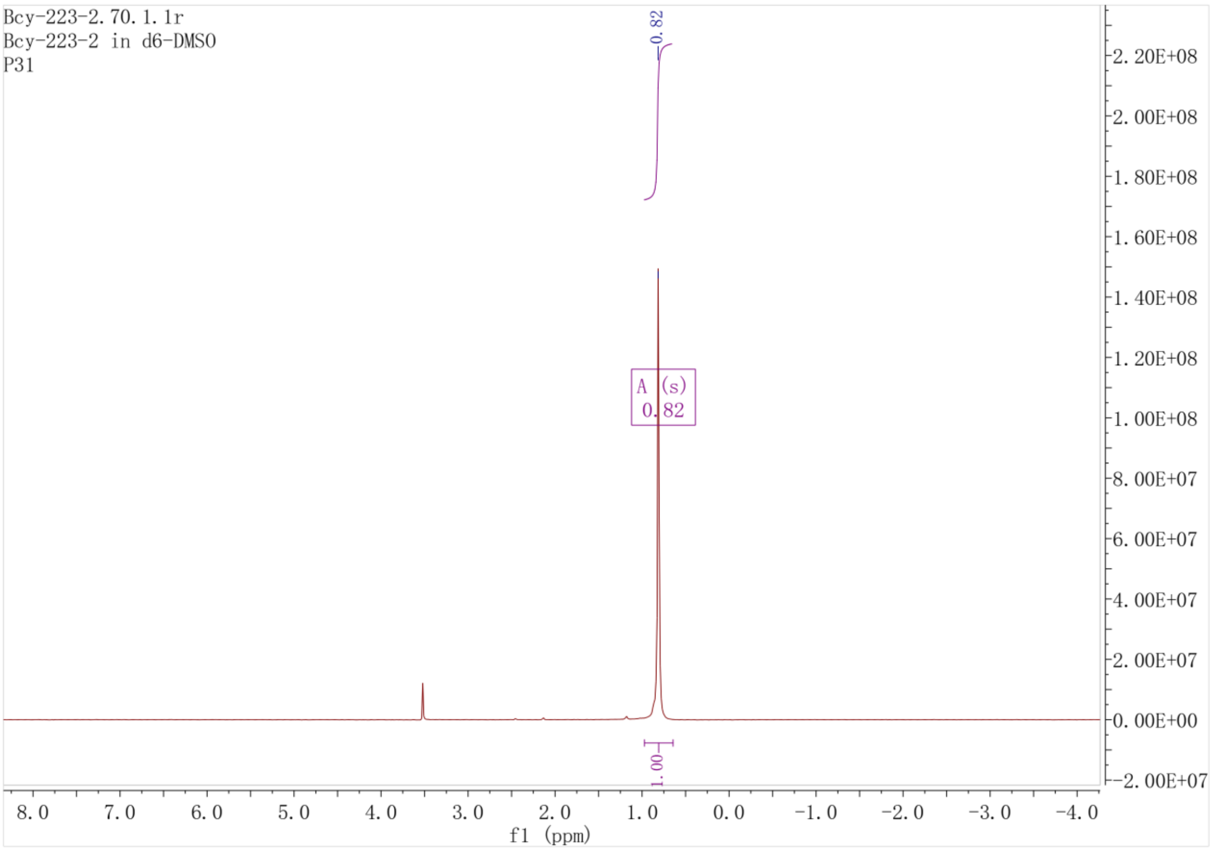

^{1}H NMR spectrum of compound **31i**

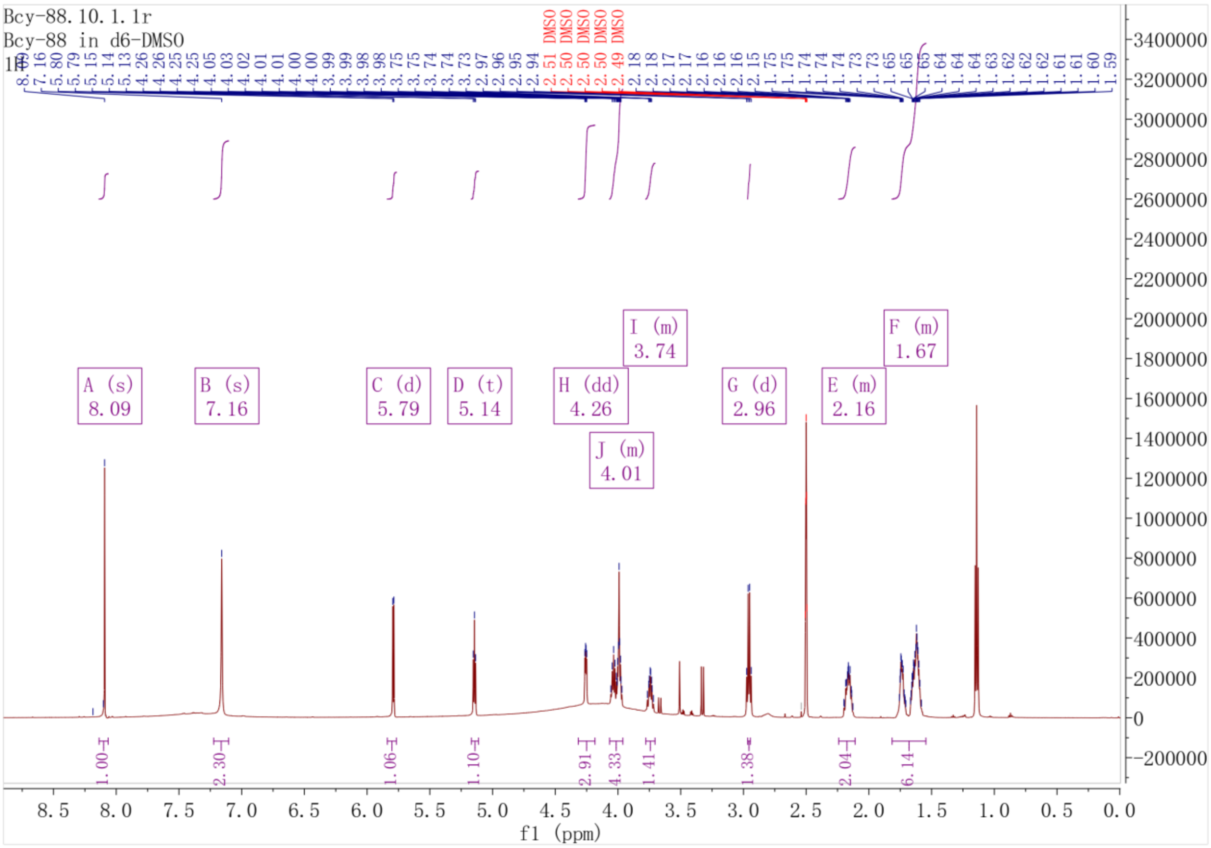

^{13}C NMR spectrum of compound **31i**

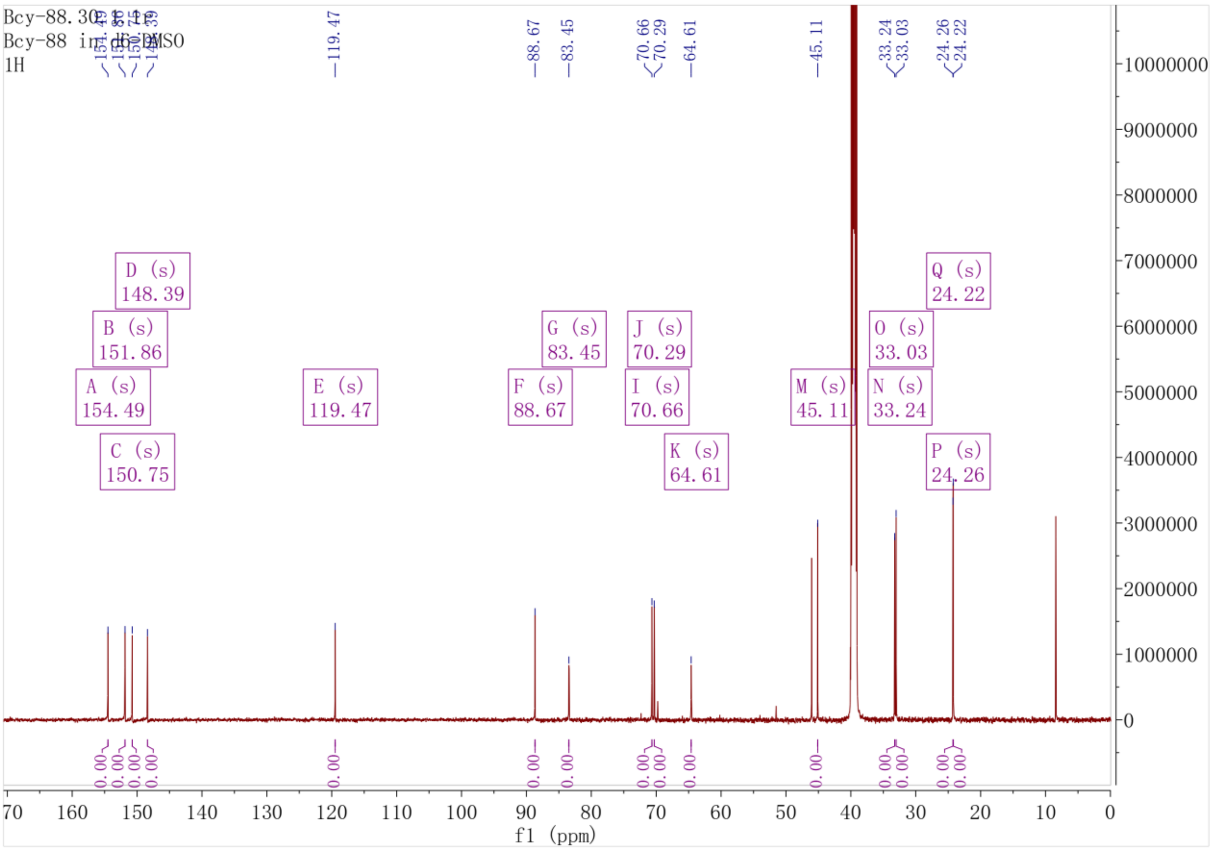

^{31}P NMR spectrum of compound **31i**

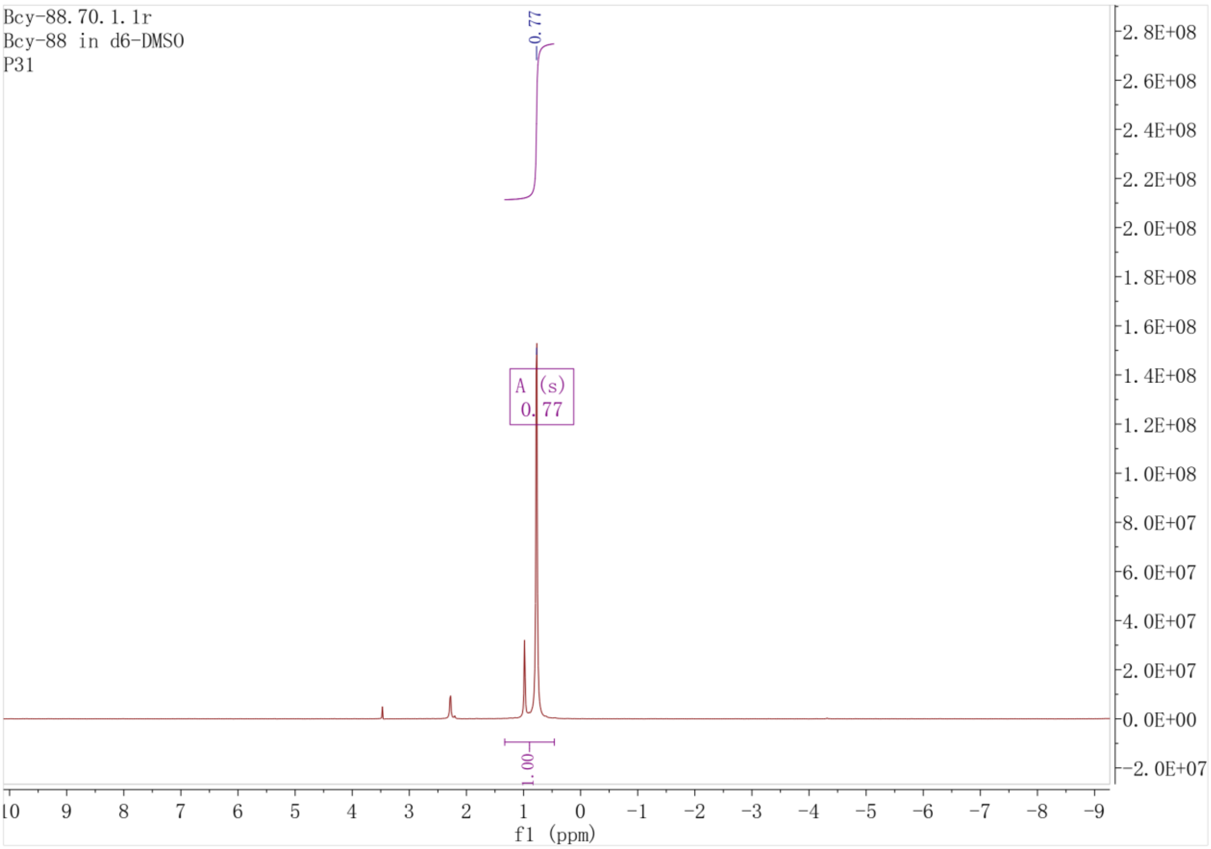

^{1}H NMR spectrum of compound **31j**

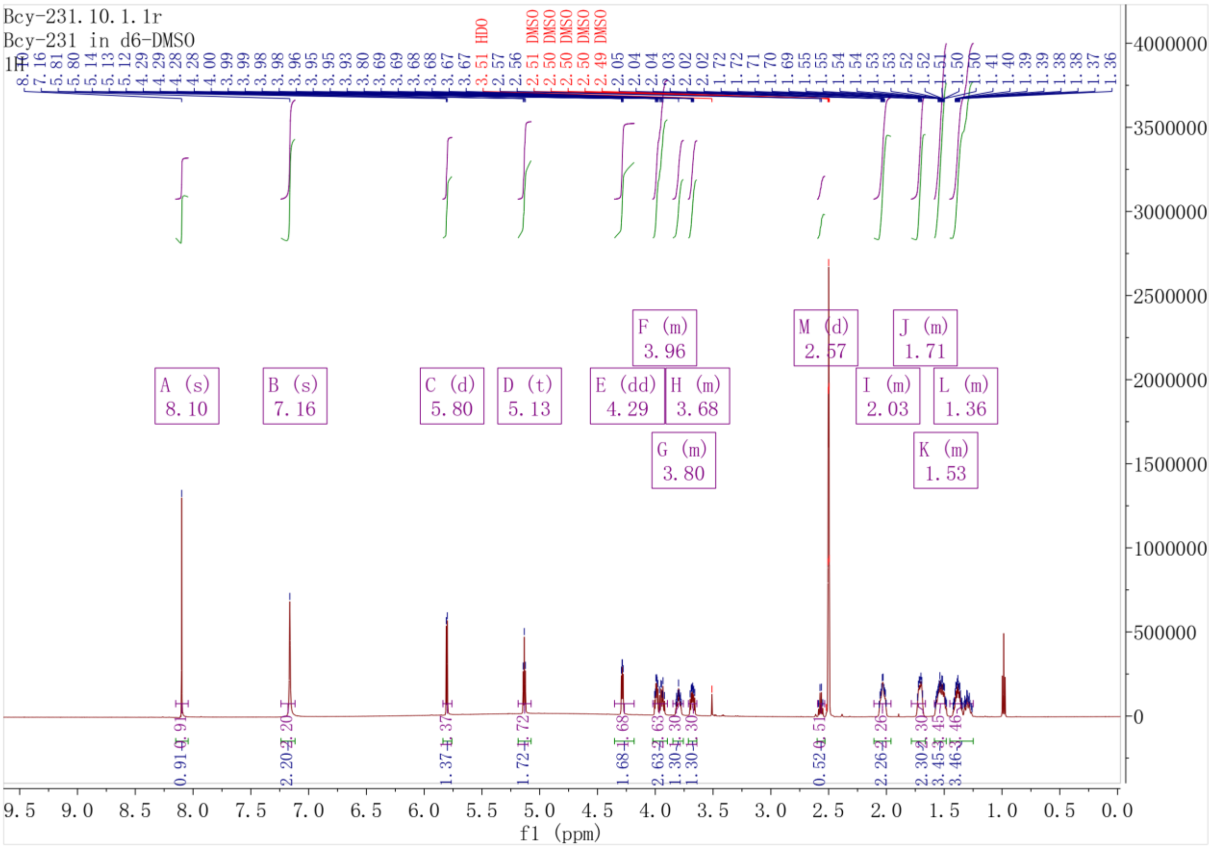

^{13}C NMR spectrum of compound **31j**

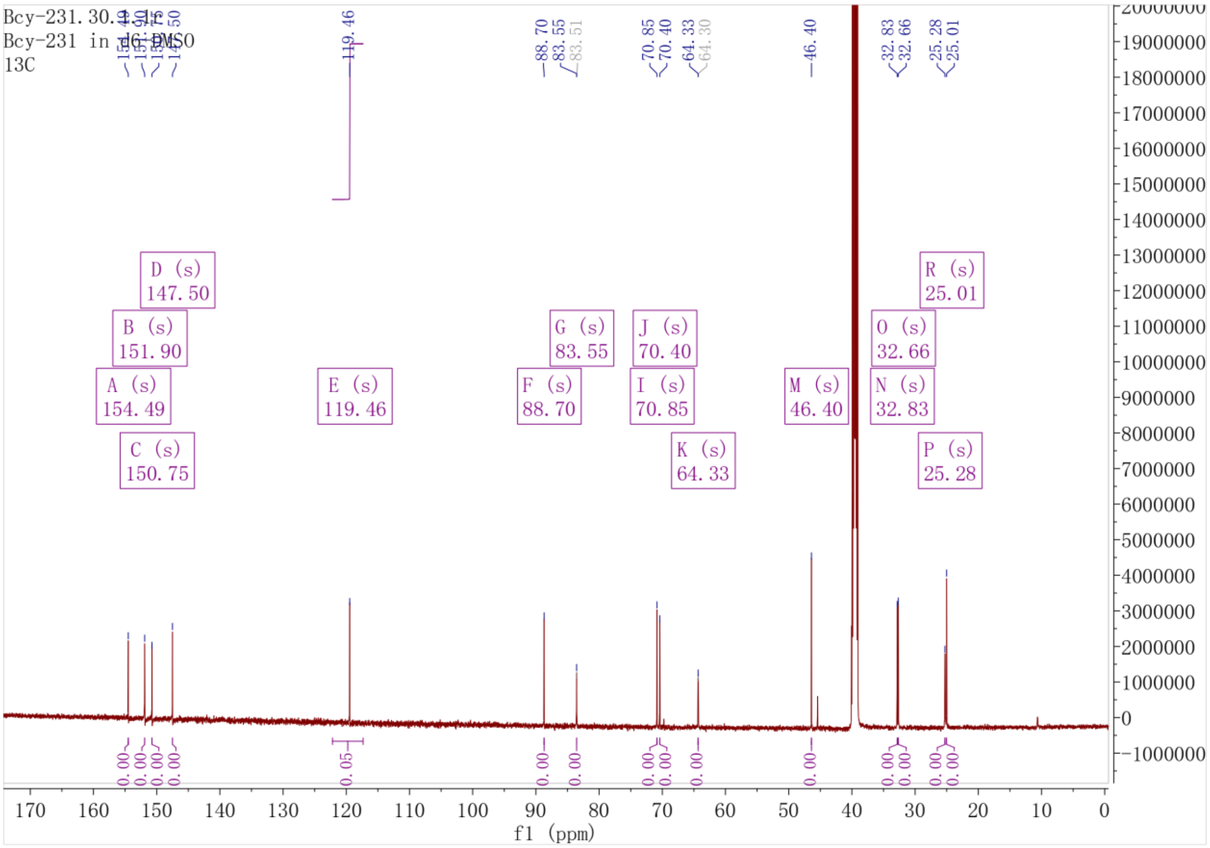

^{31}P NMR spectrum of compound **31j**

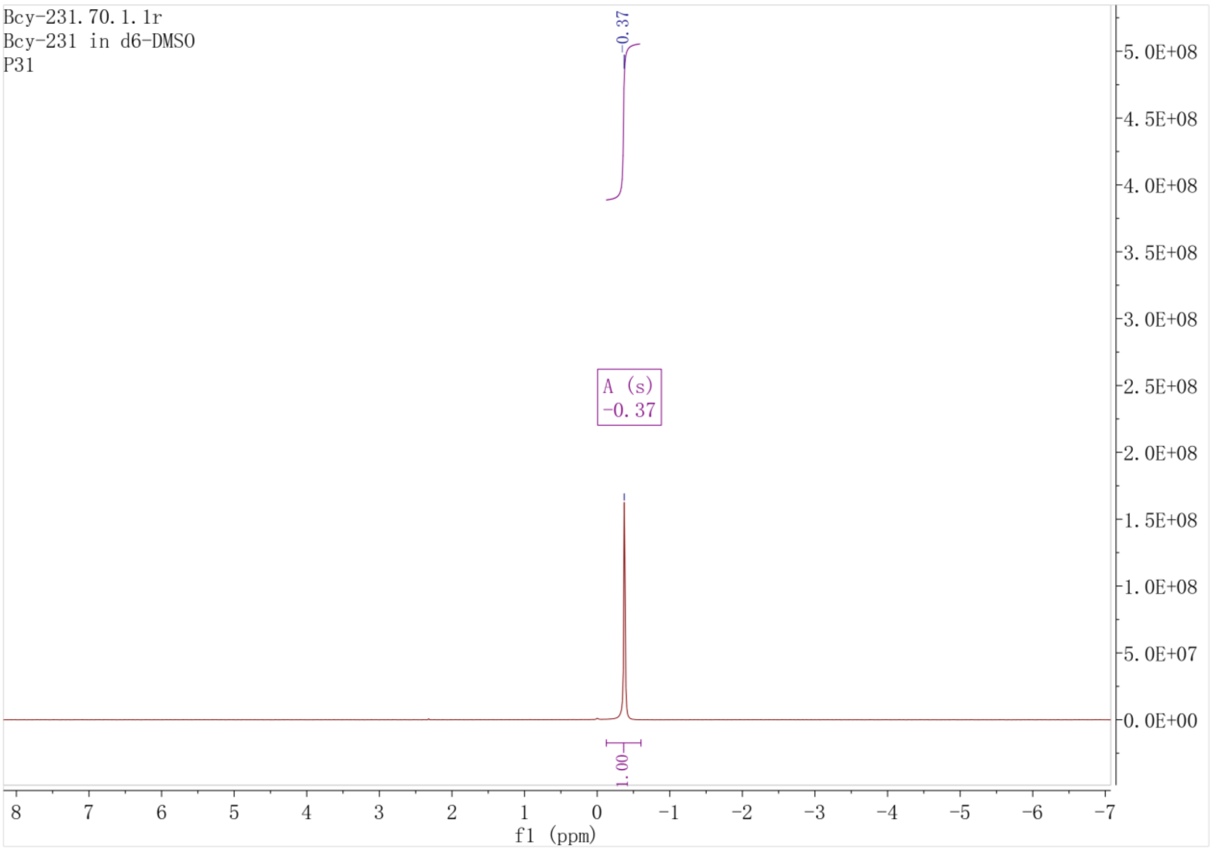

^{1}H NMR spectrum of compound **31r**

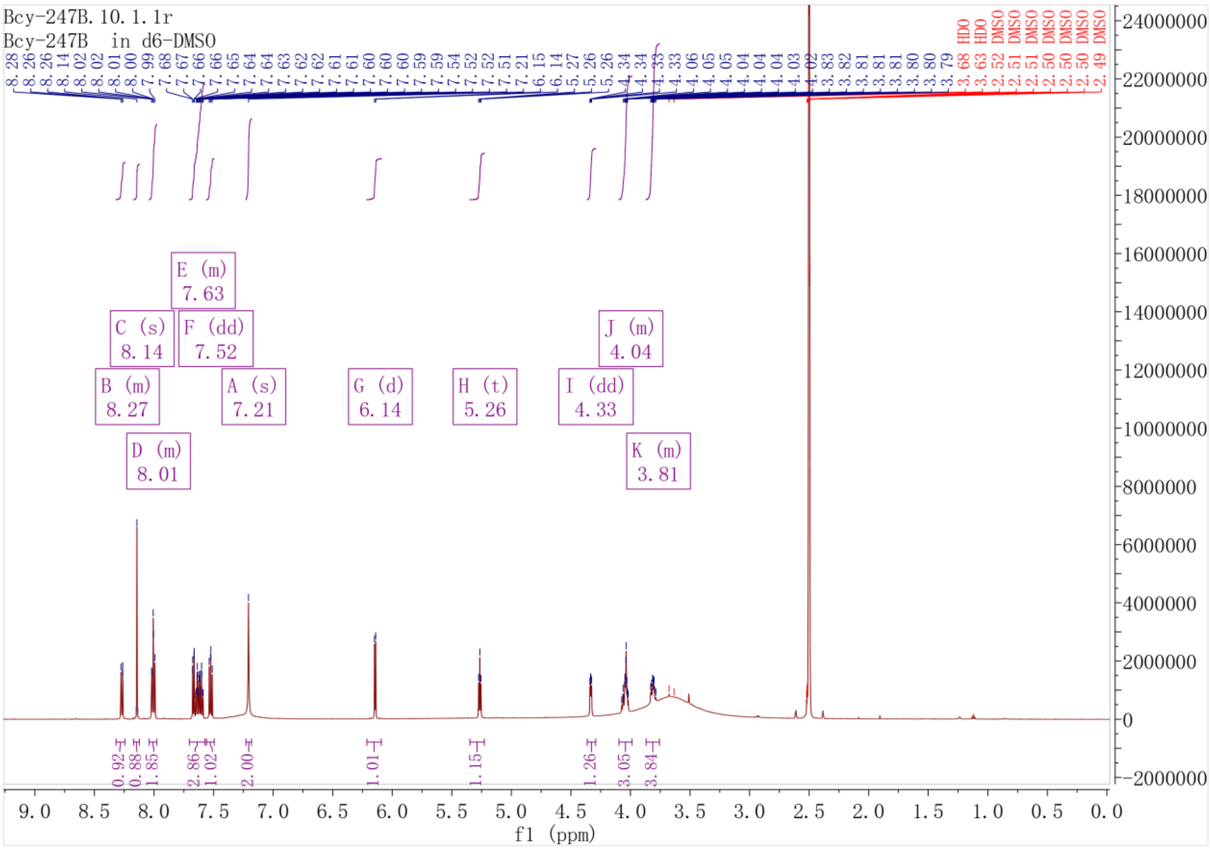

^{13}C NMR spectrum of compound **31r**

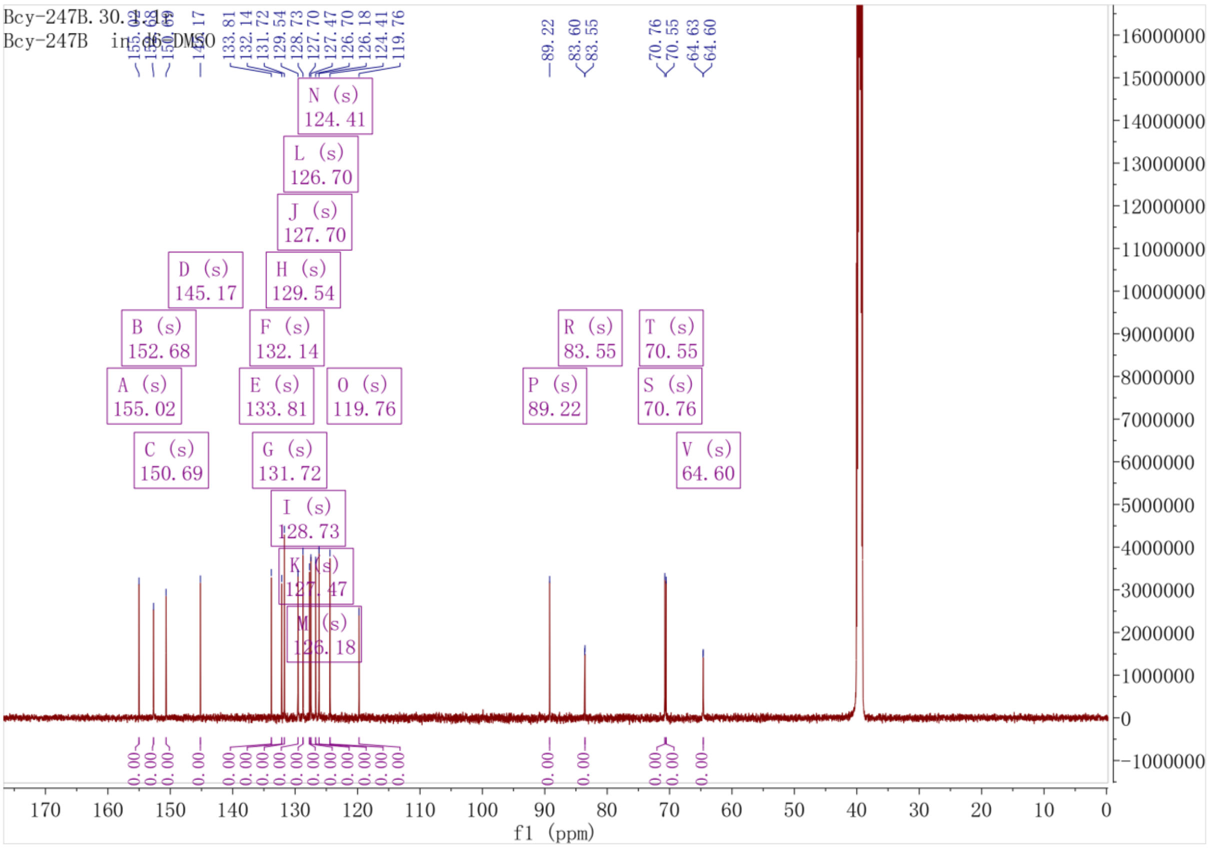

^{31}P NMR spectrum of compound **31r**

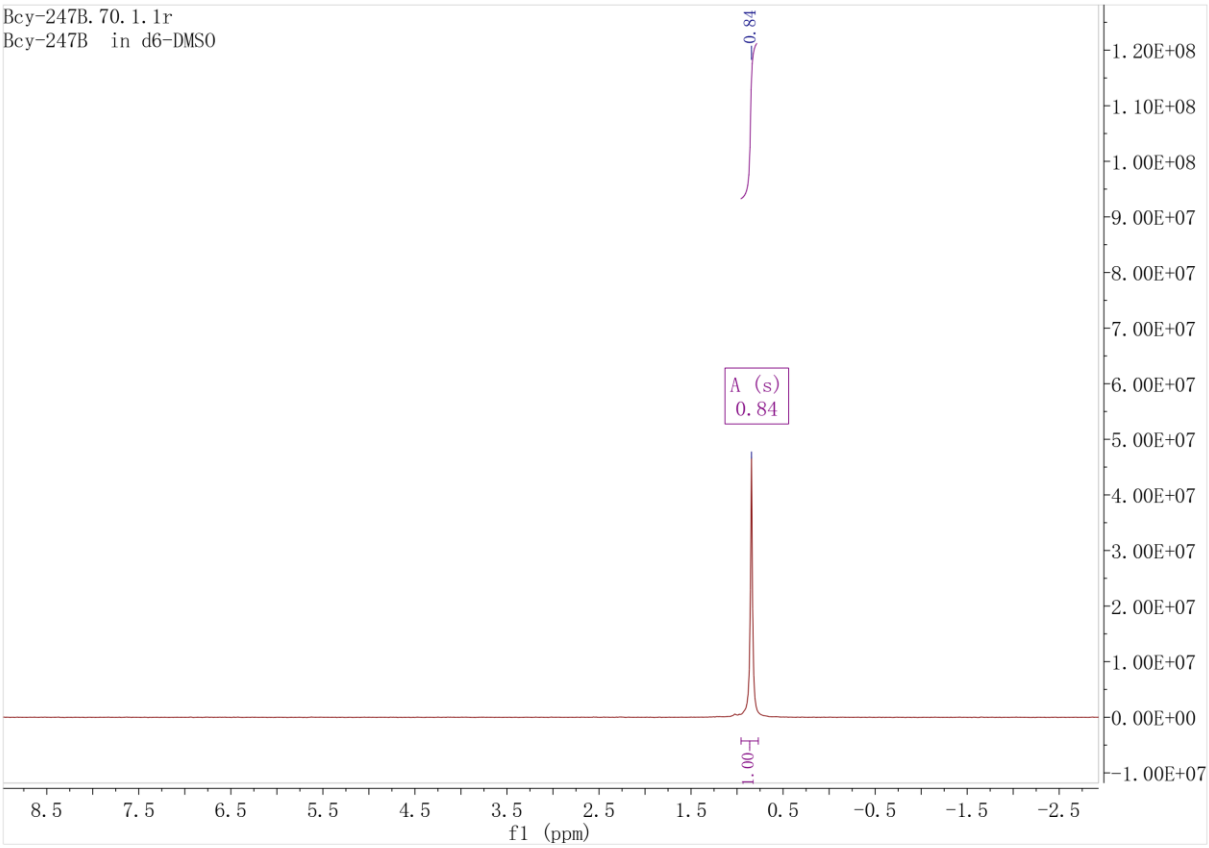

^{1}H NMR spectrum of compound **33f**

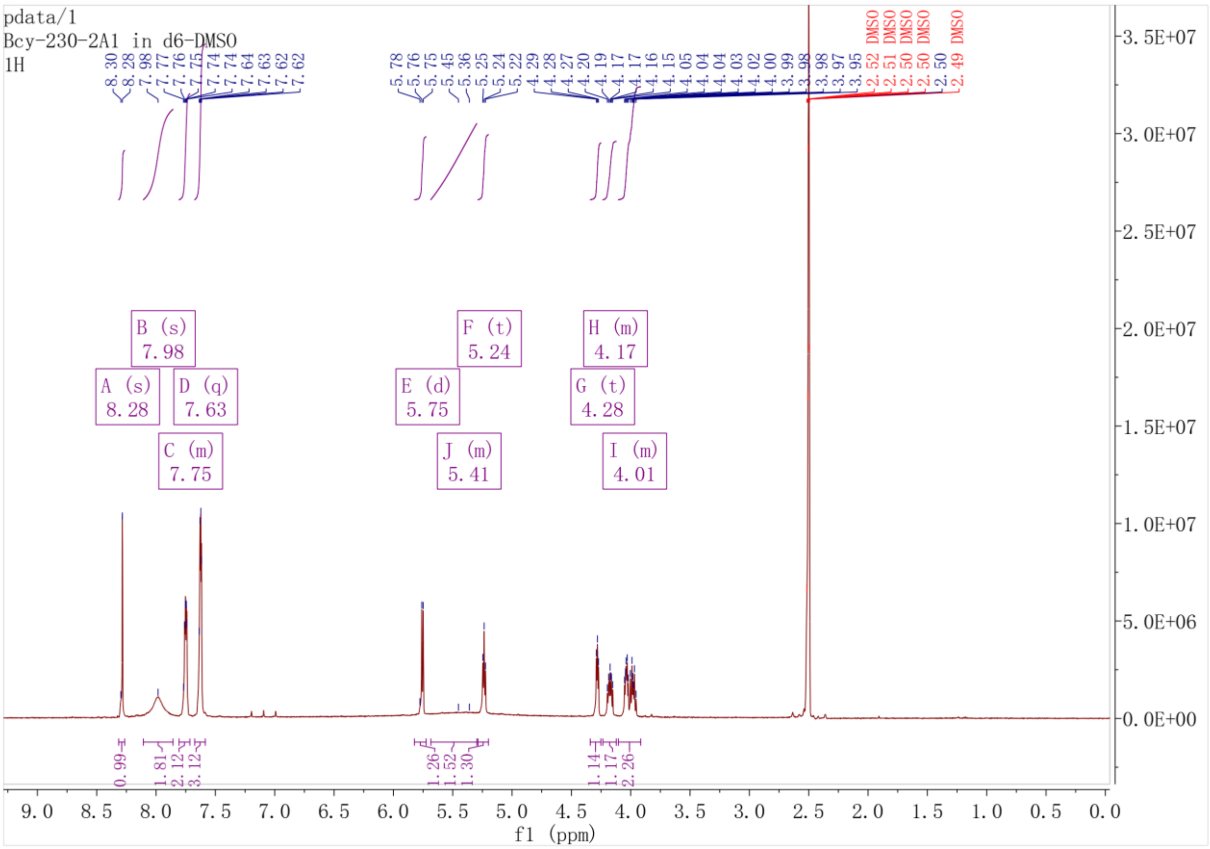

^{13}C NMR spectrum of compound **33f**

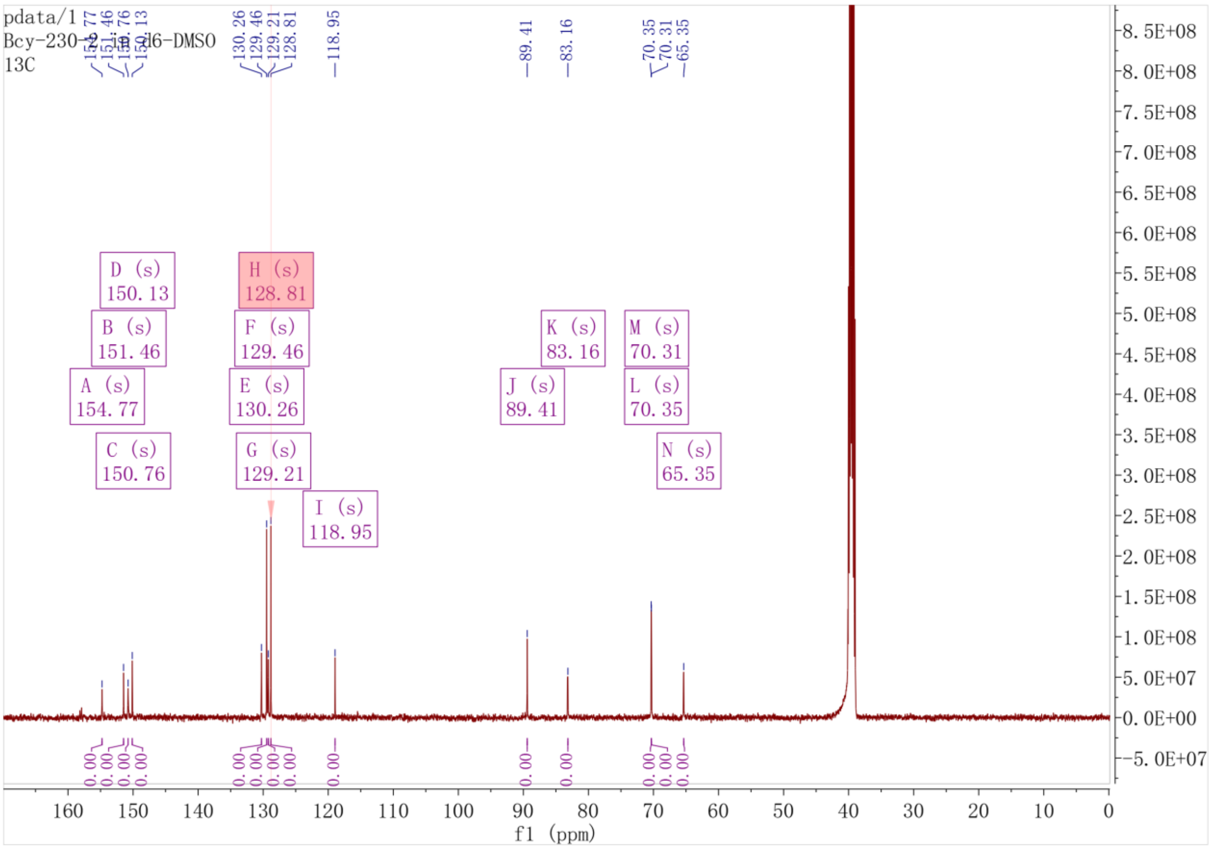

^{31}P NMR spectrum of compound **33f**

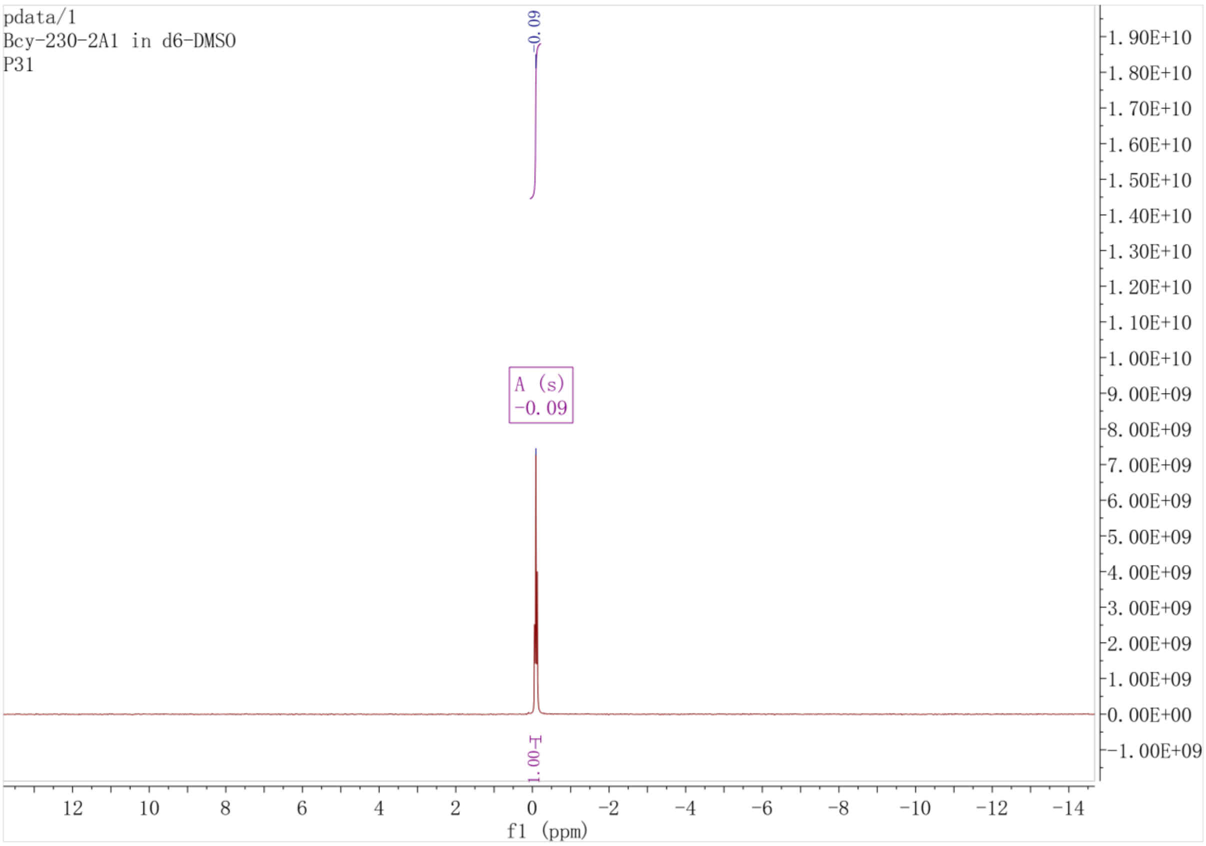

^{1}H NMR spectrum of compound **33e**

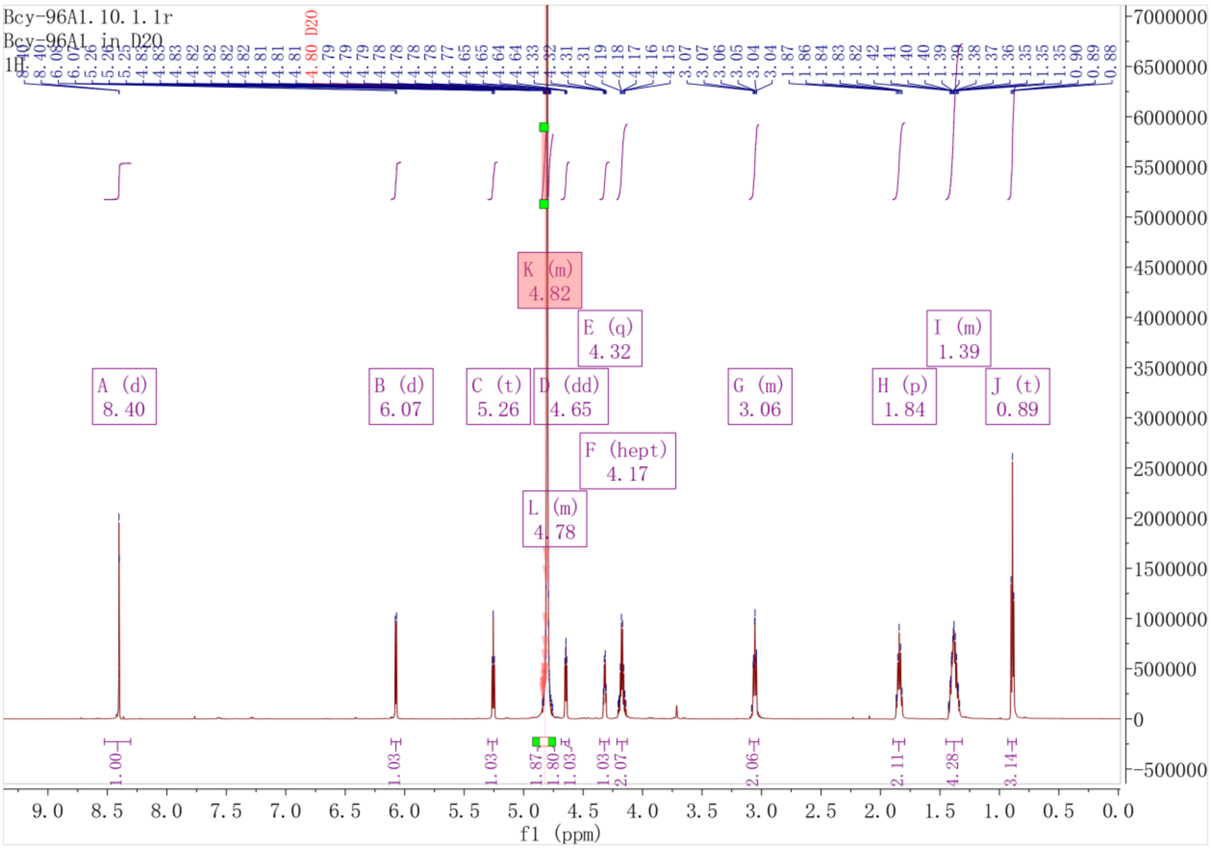

^{13}C NMR spectrum of compound **33e**

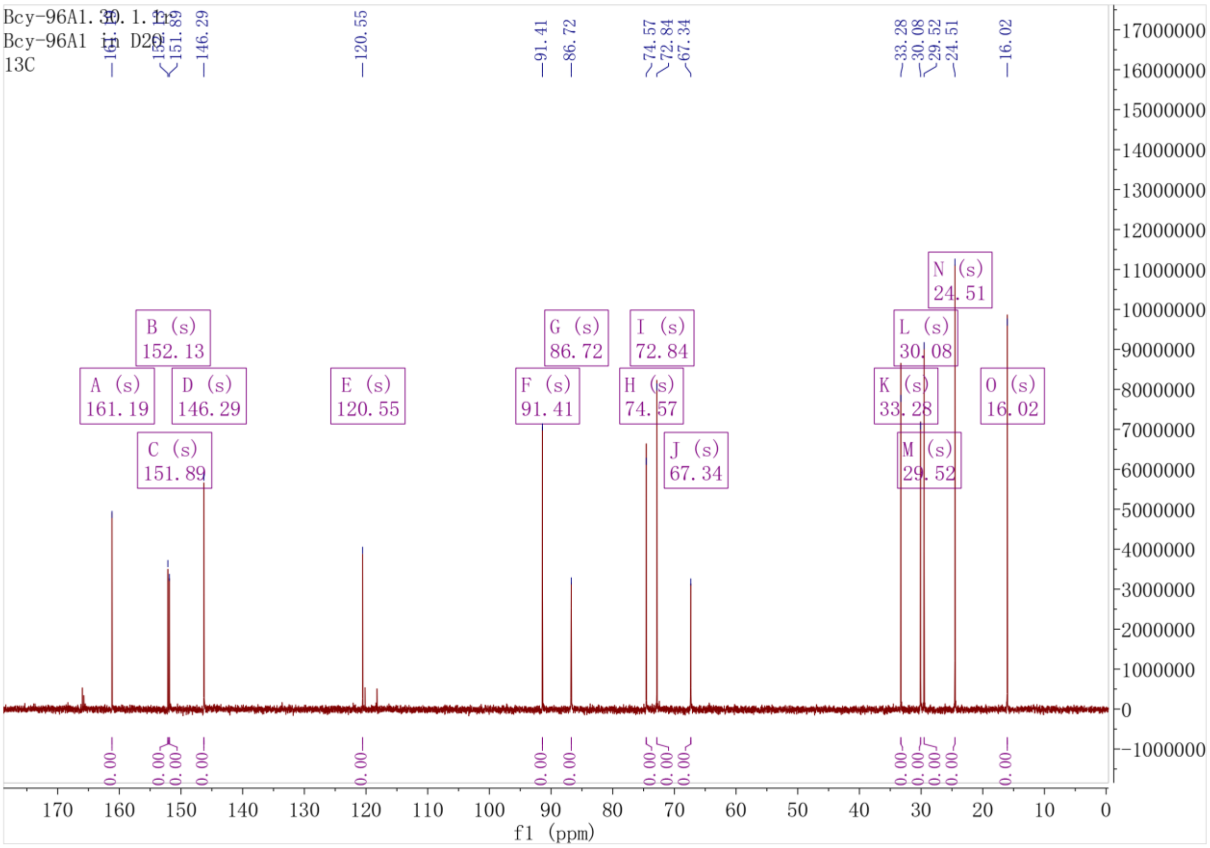

^{31}P NMR spectrum of compound **33e**

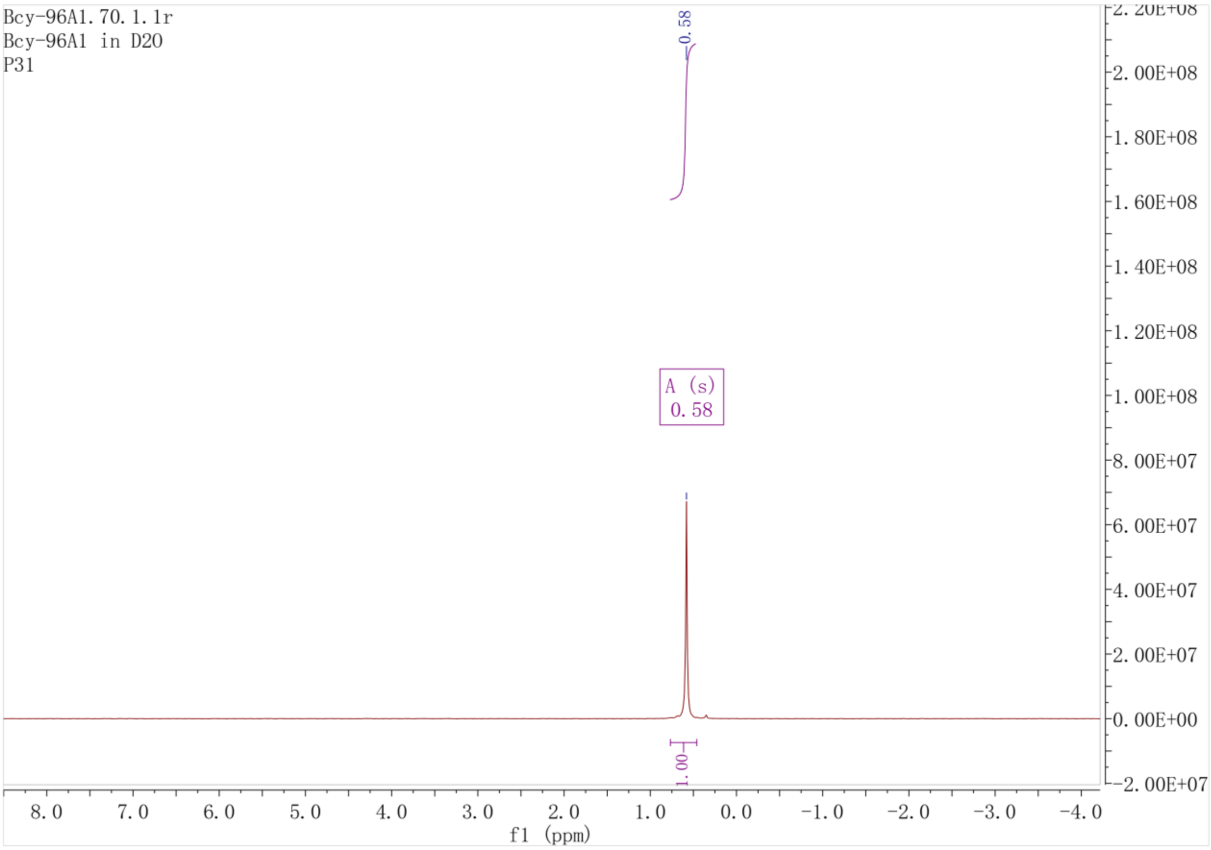

^{1}H NMR spectrum of compound **42a**

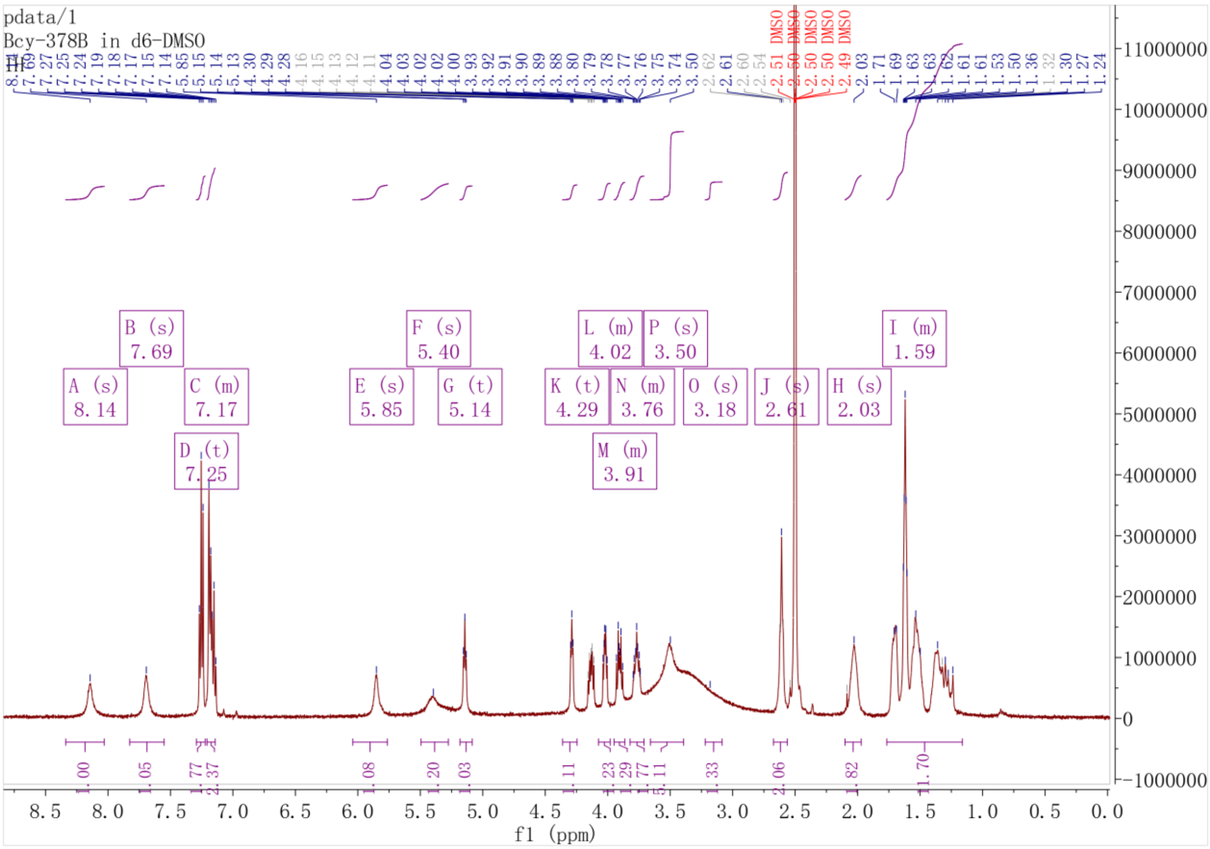

^{13}C NMR spectrum of compound **42a**

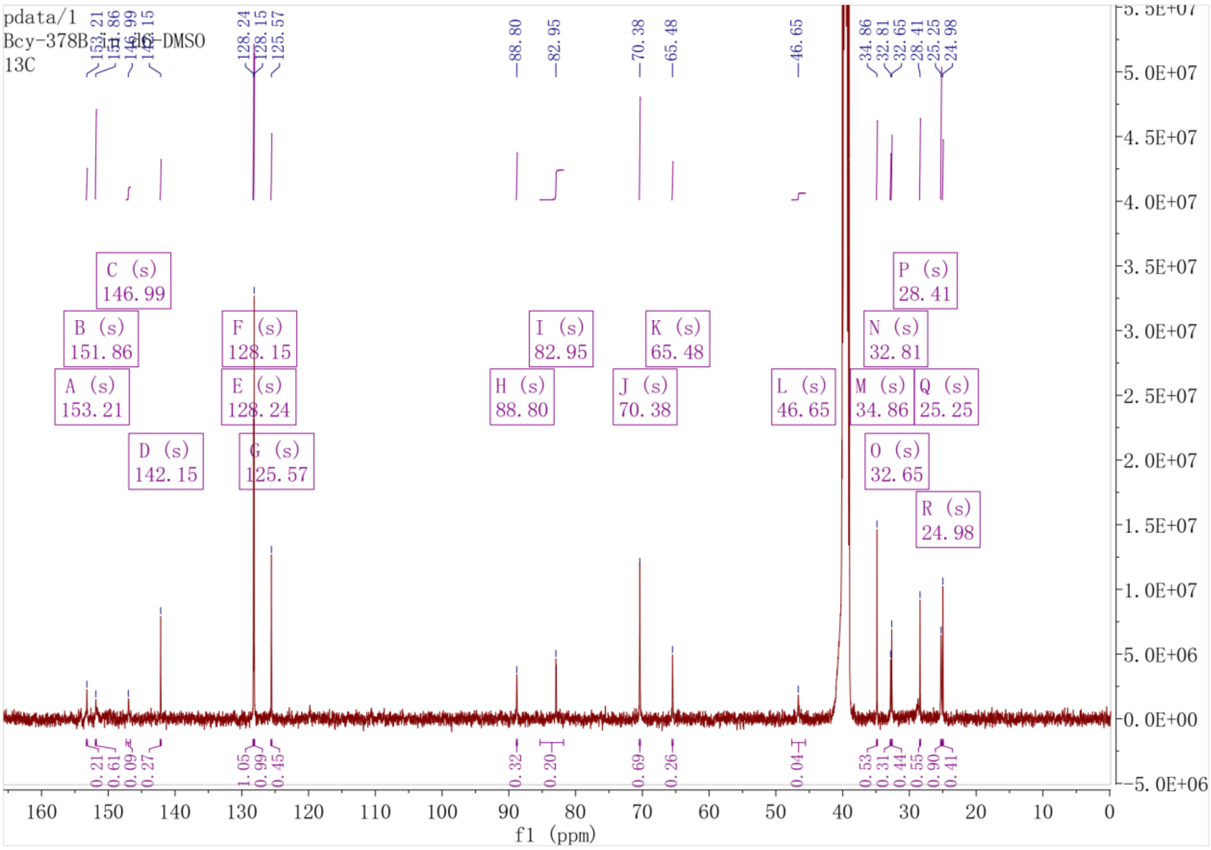

^{31}P NMR spectrum of compound **42a**

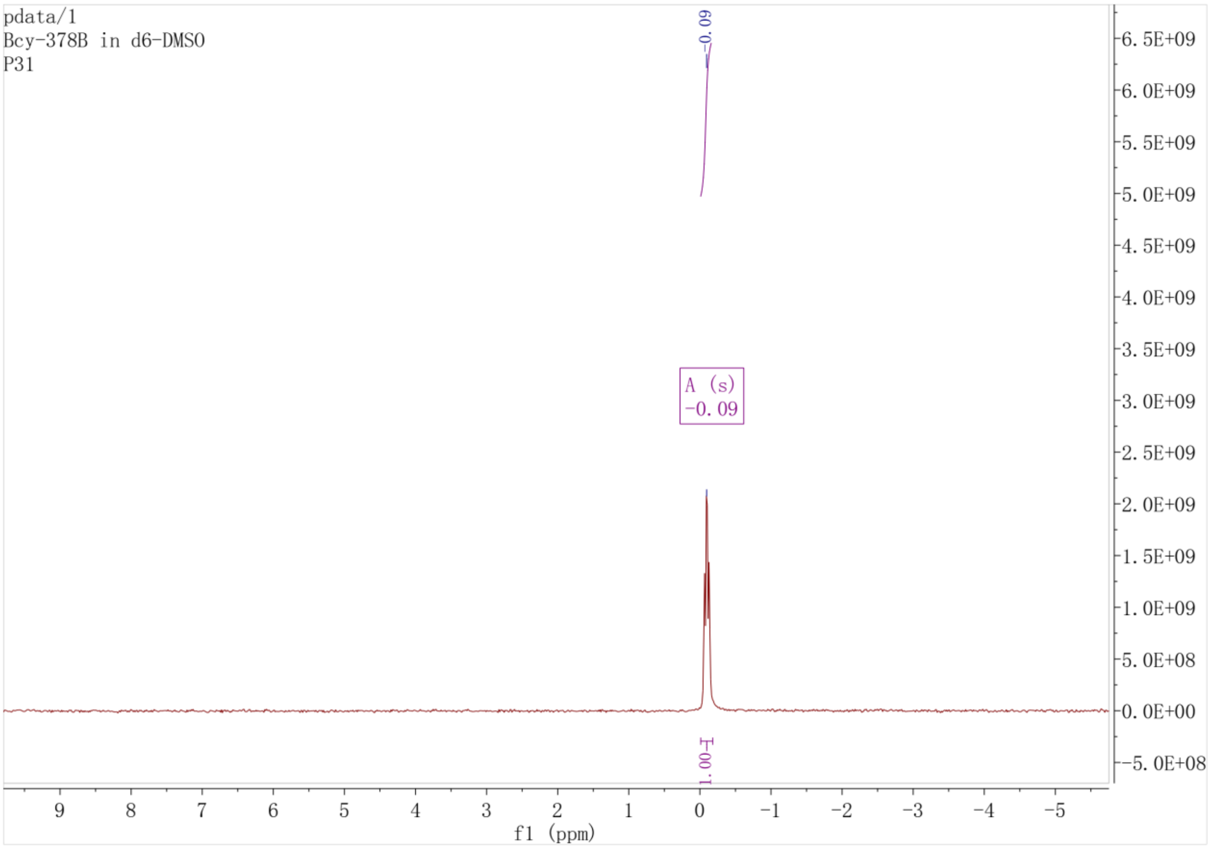

^{1}H NMR spectrum of compound **42b**

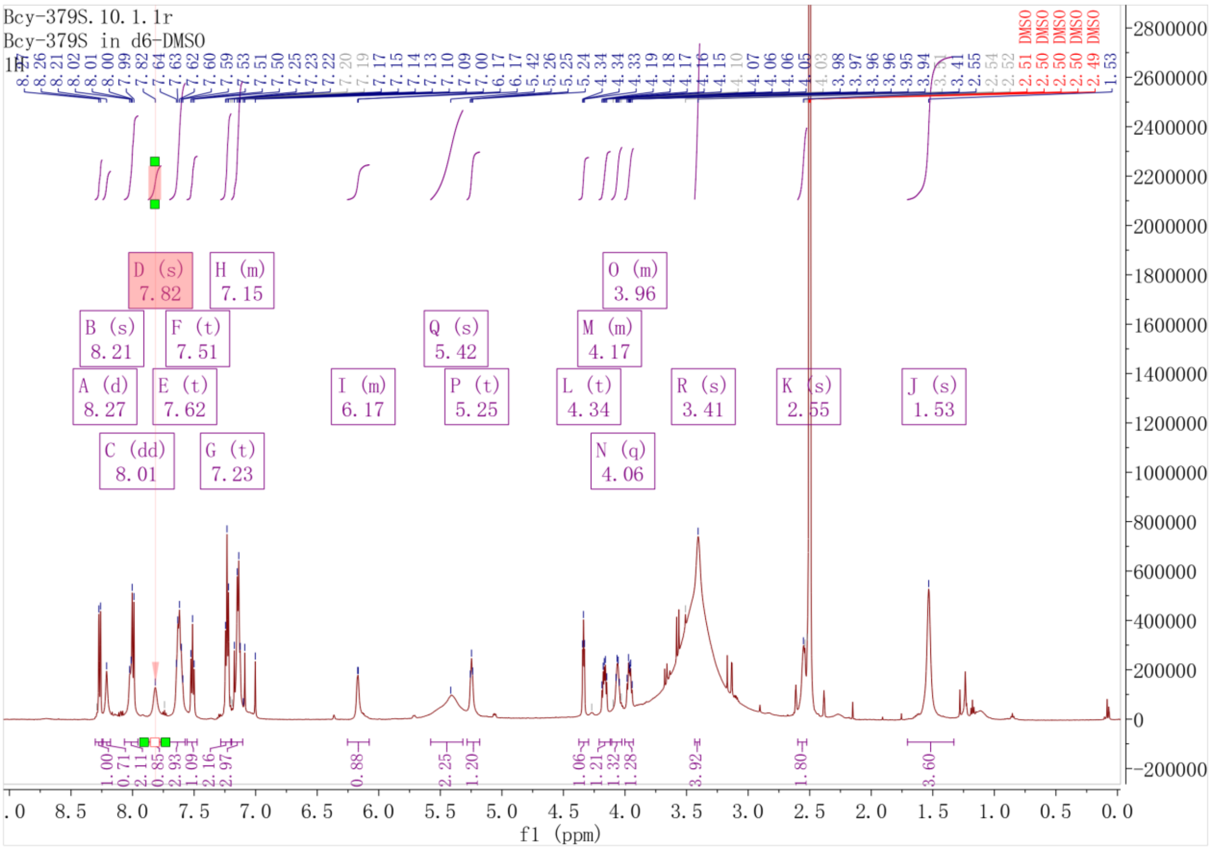

^{13}C NMR spectrum of compound **42b**

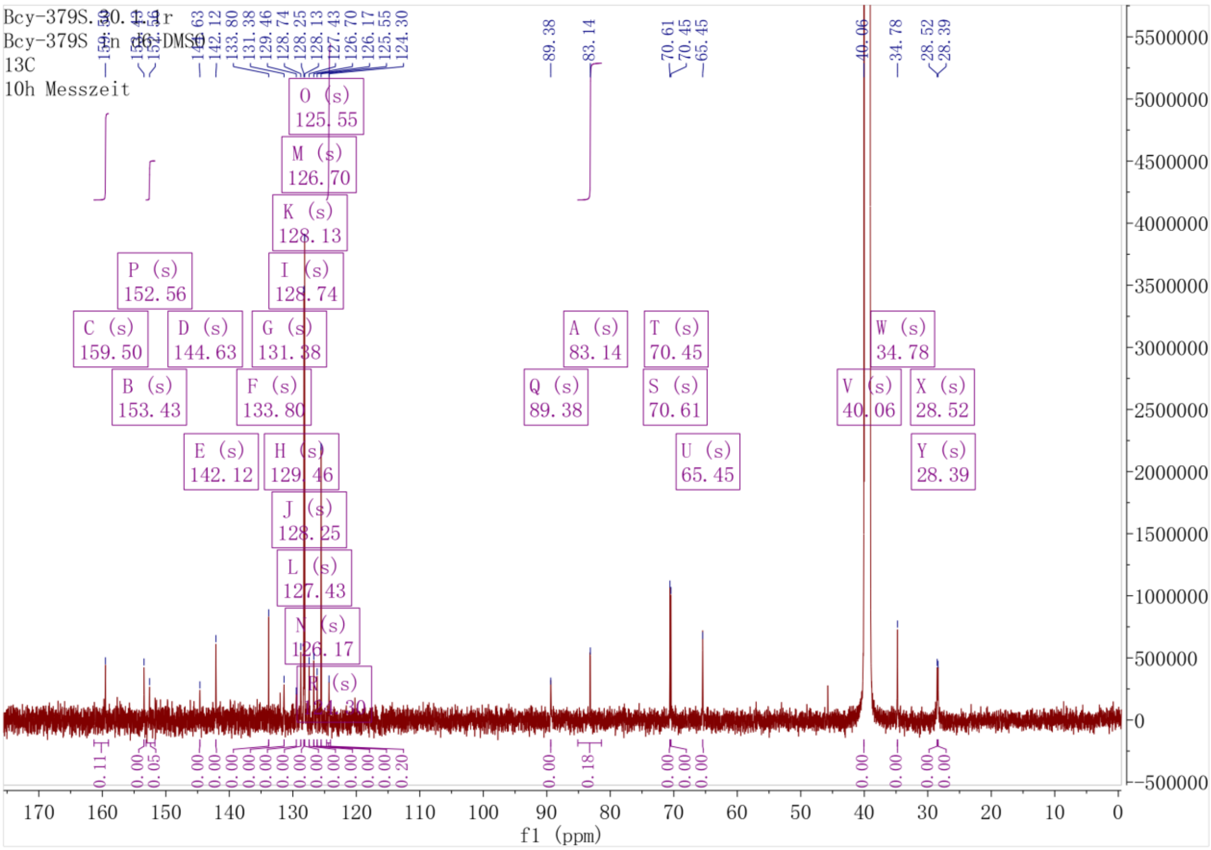

^{31}P NMR spectrum of compound **42b**

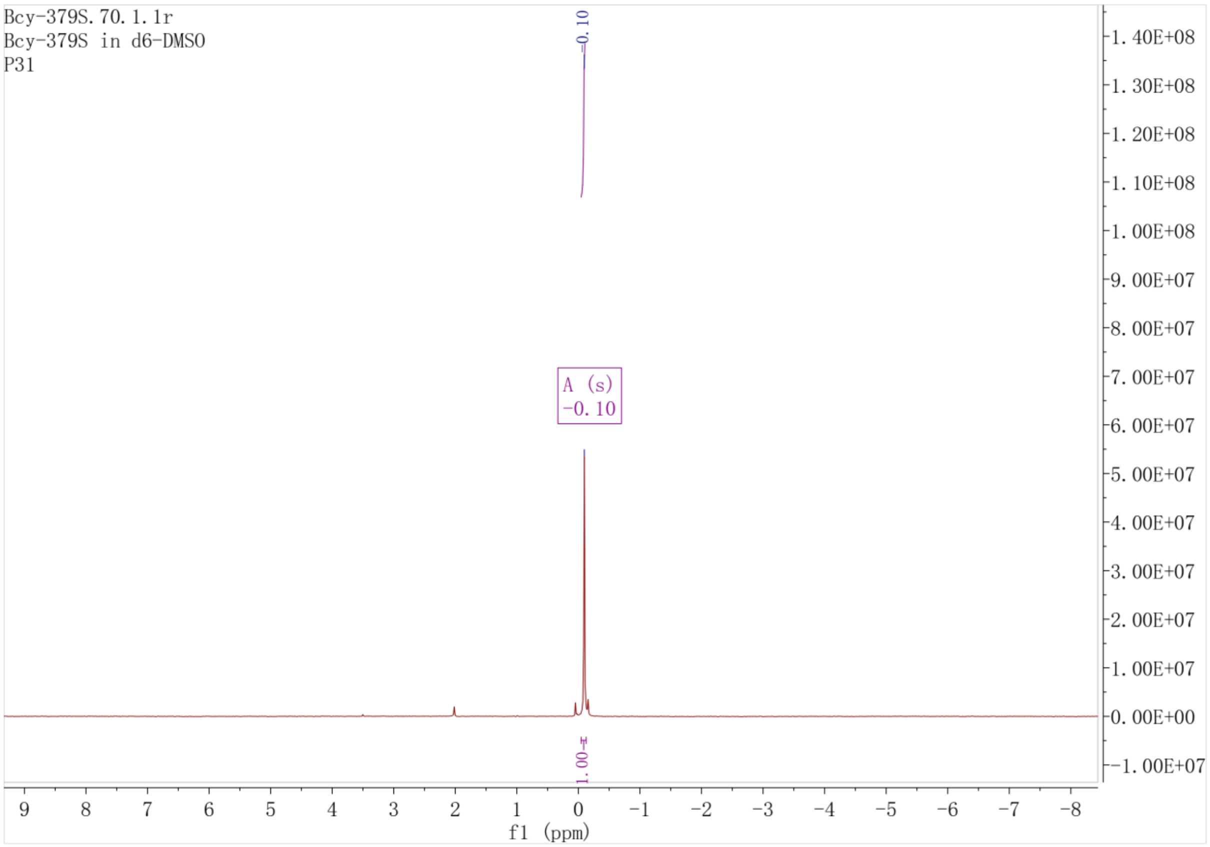

^{1}H NMR spectrum of compound **42c**

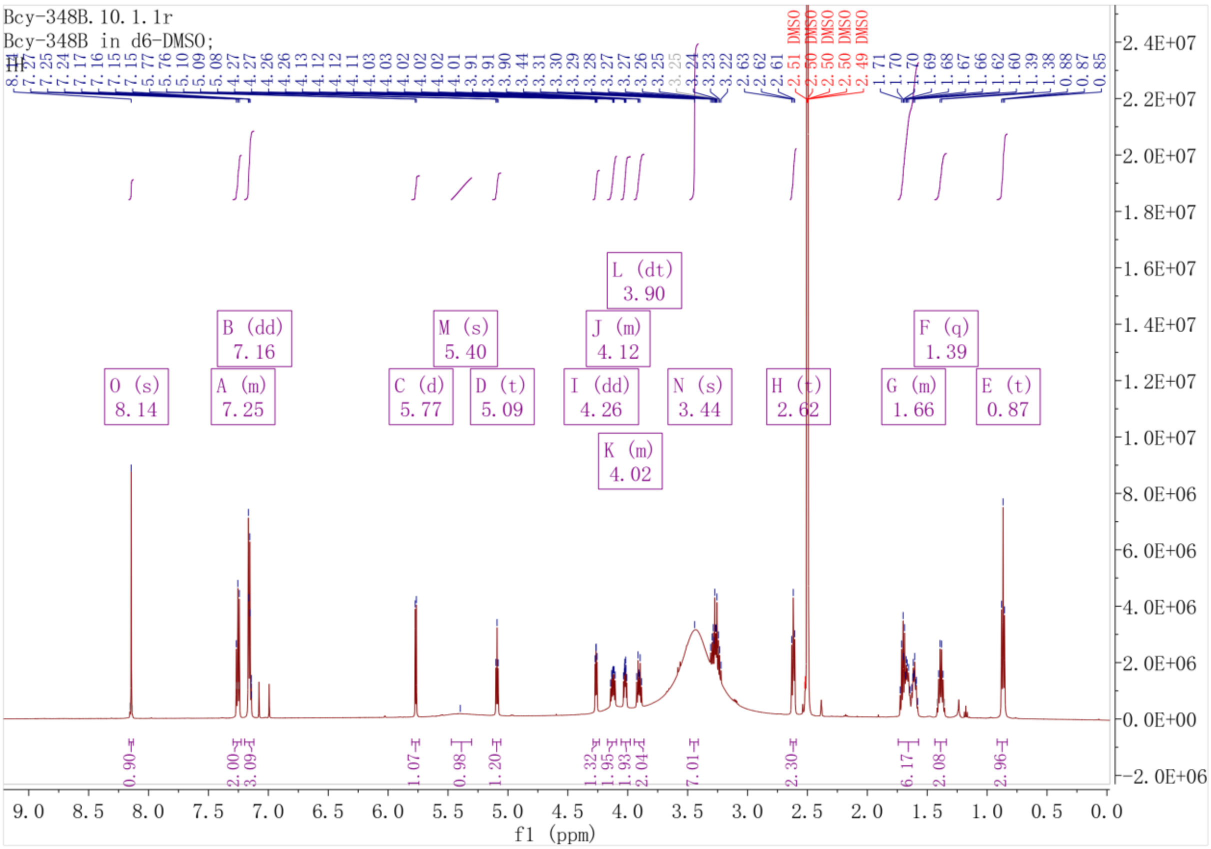

^{13}C NMR spectrum of compound **42c**

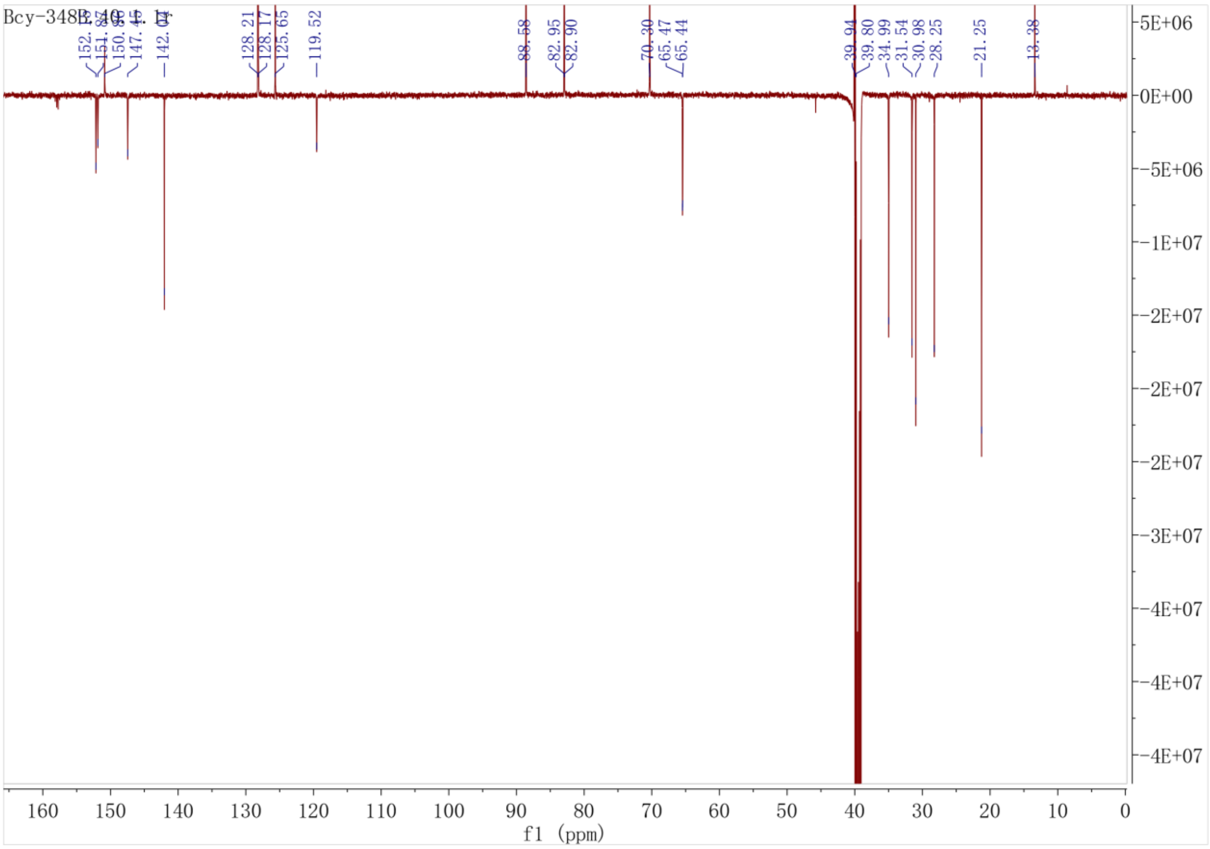

^{31}P NMR spectrum of compound **42c**

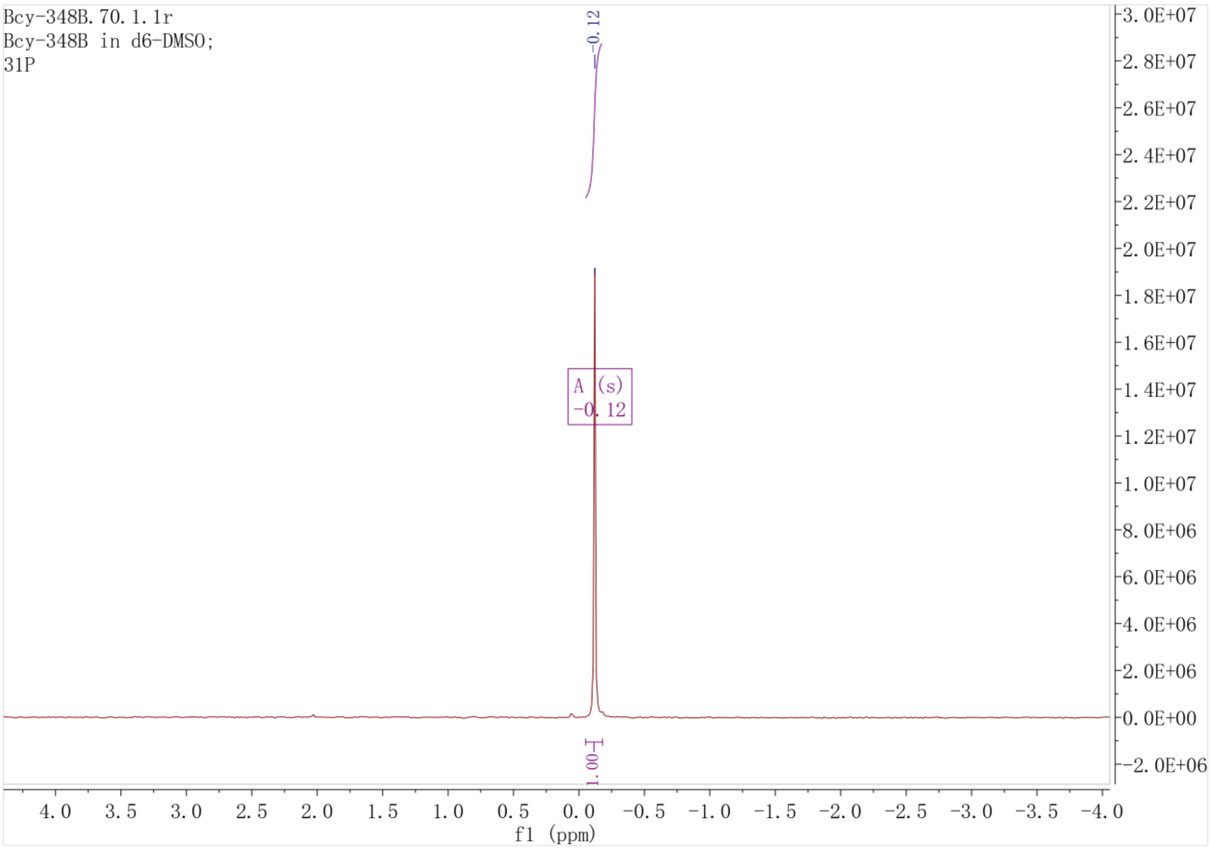

^{1}H NMR spectrum of compound **42d**

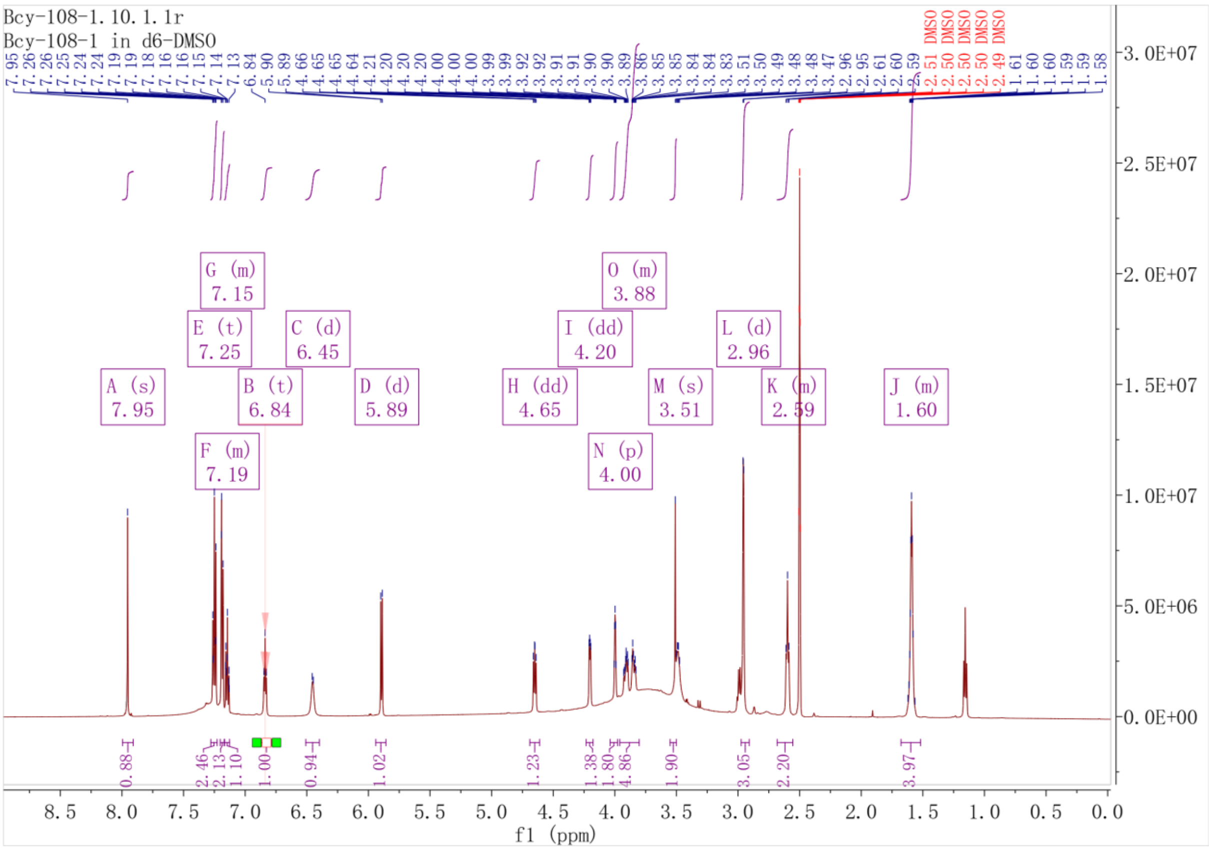

^{13}C NMR spectrum of compound **42d**

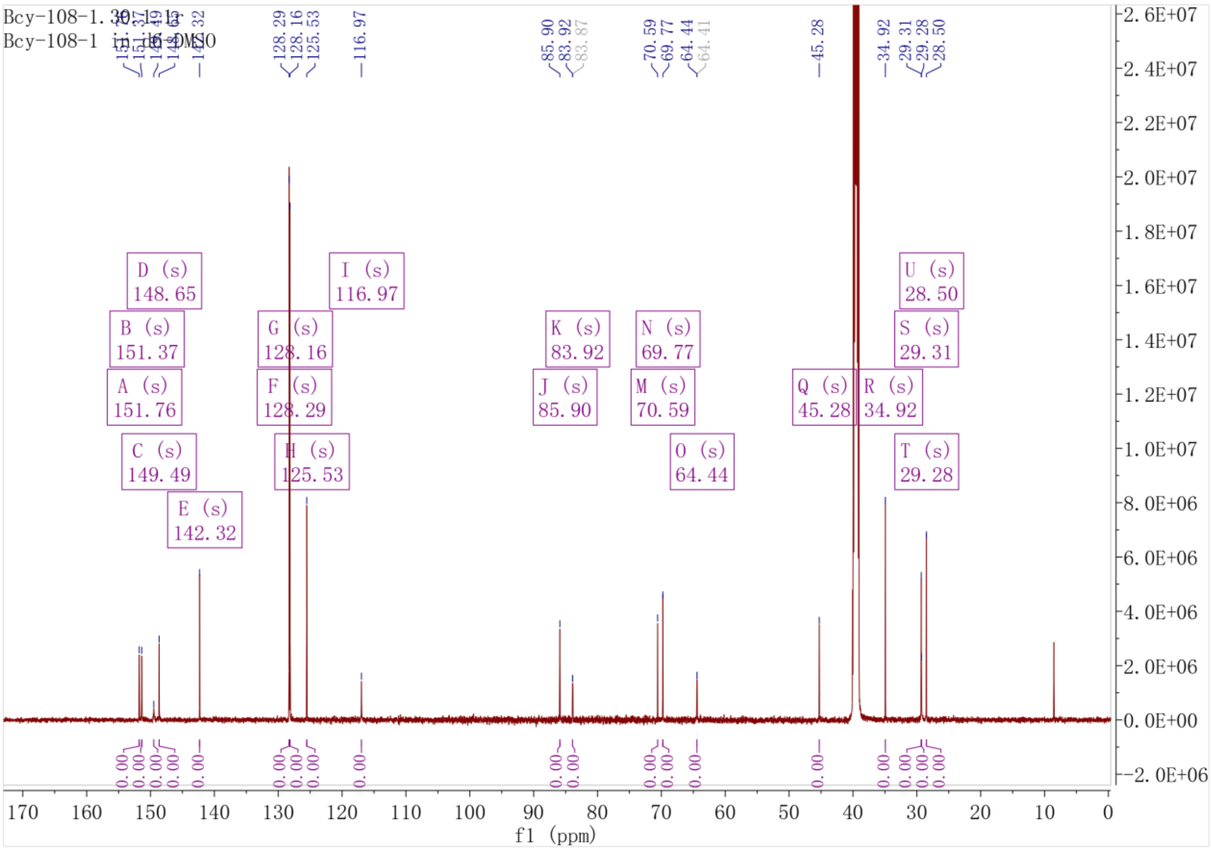

^{31}P NMR spectrum of compound **42d**

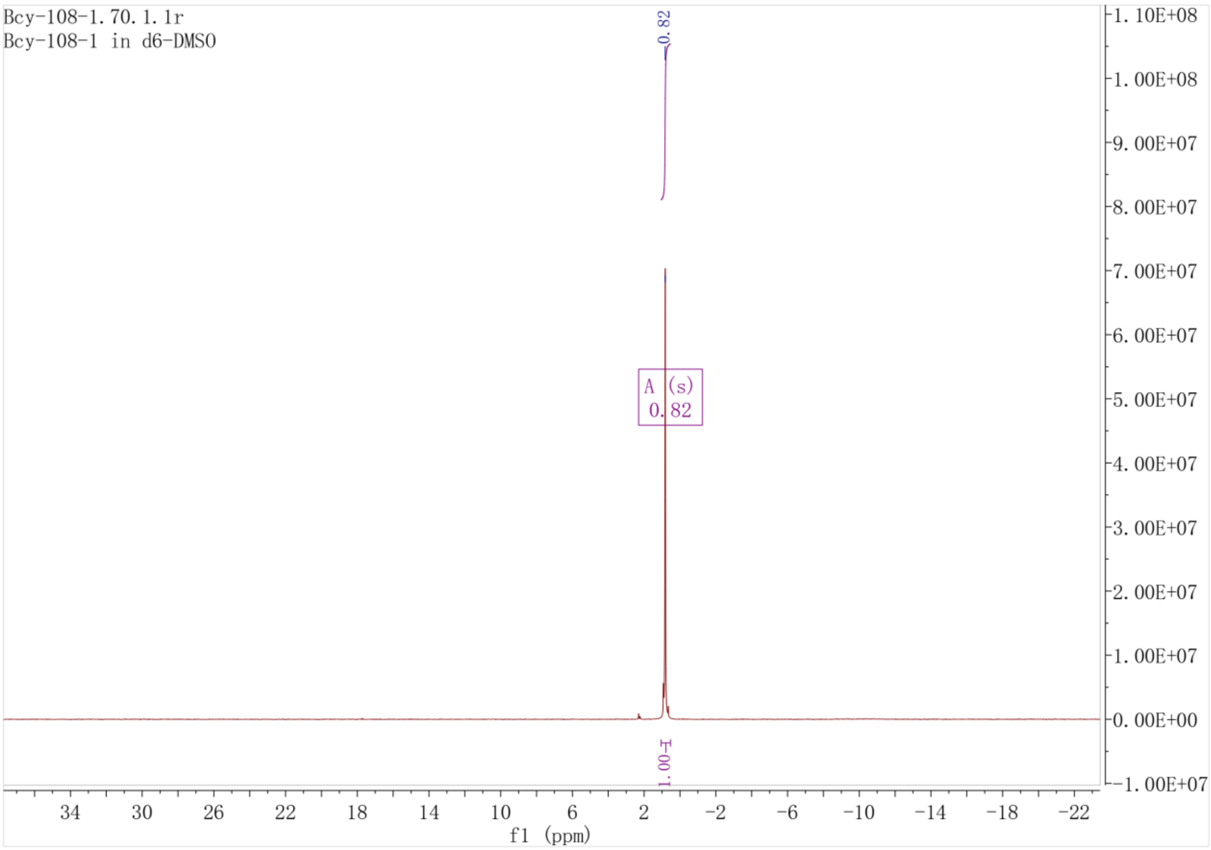

^{1}H NMR spectrum of compound **42e**

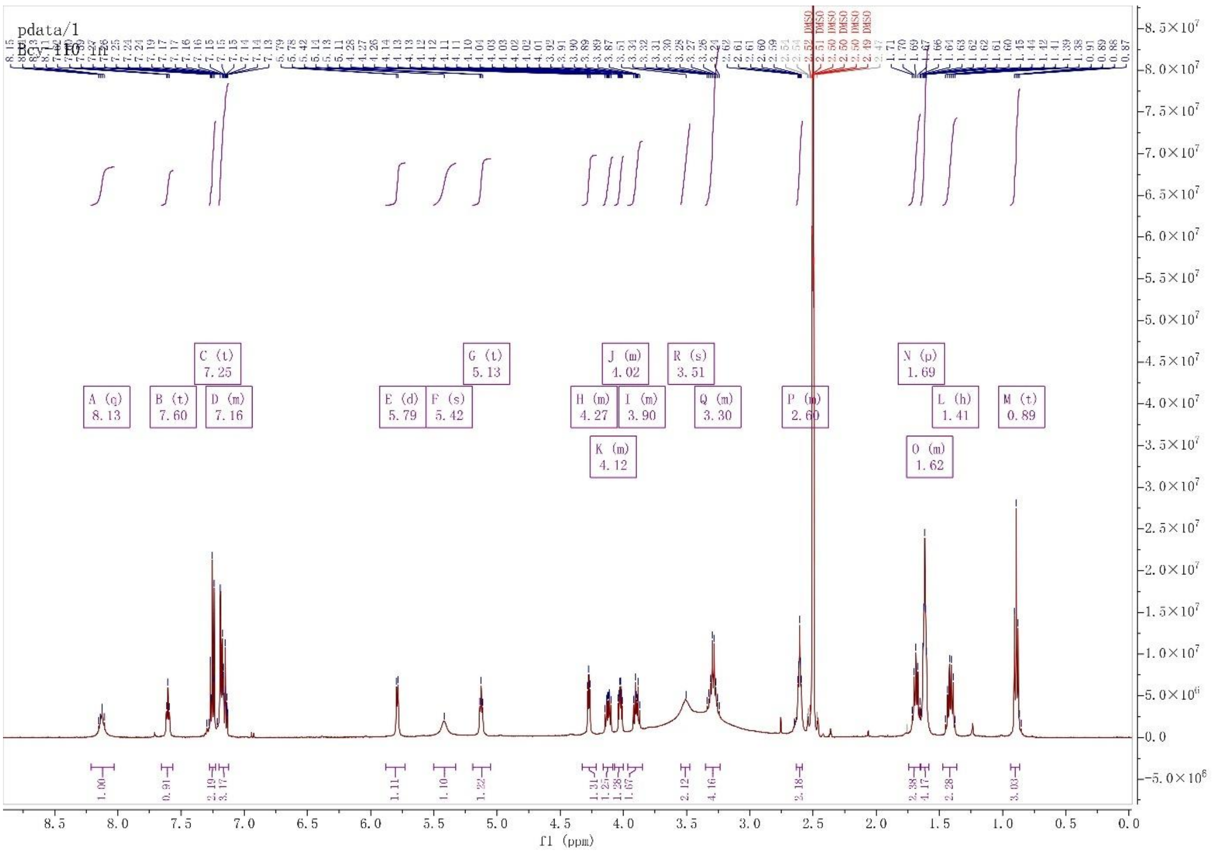

^{13}C NMR spectrum of compound **42e**

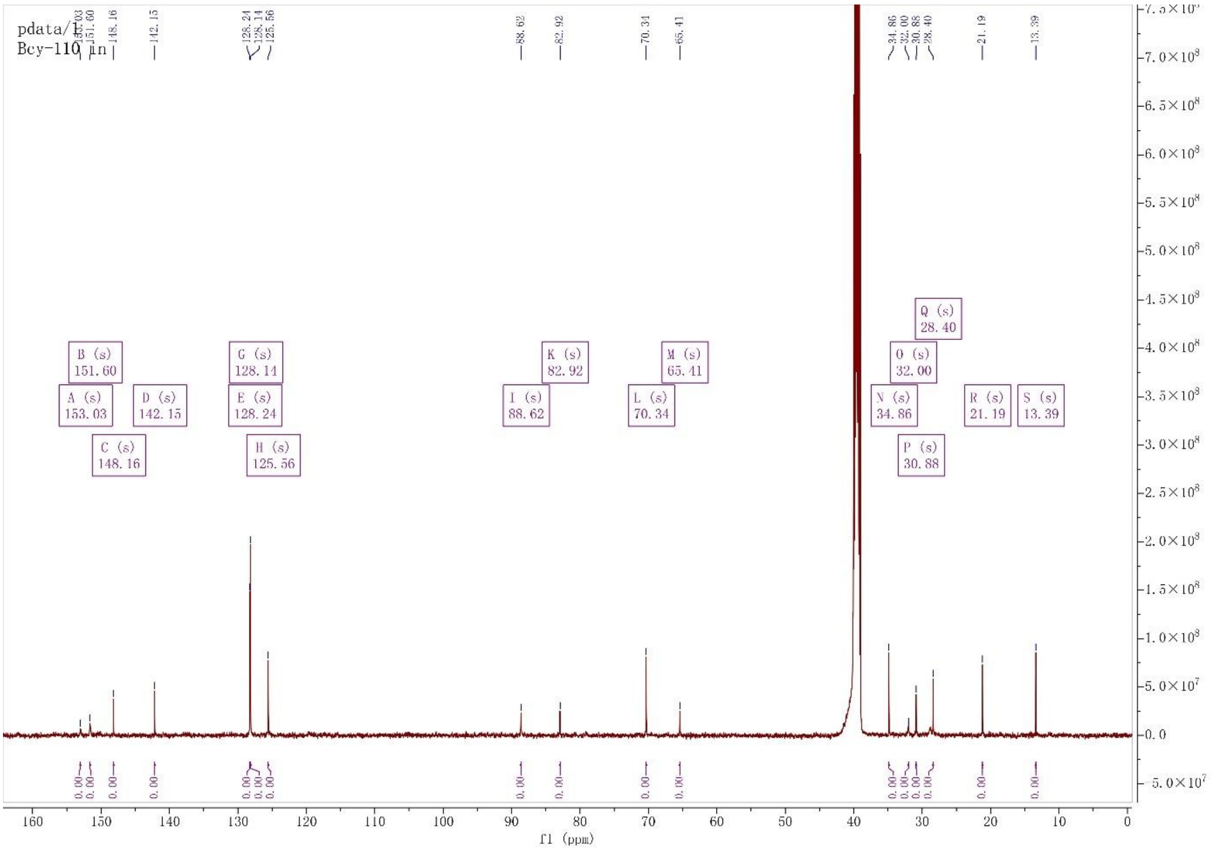

^{31}P NMR spectrum of compound **42e**

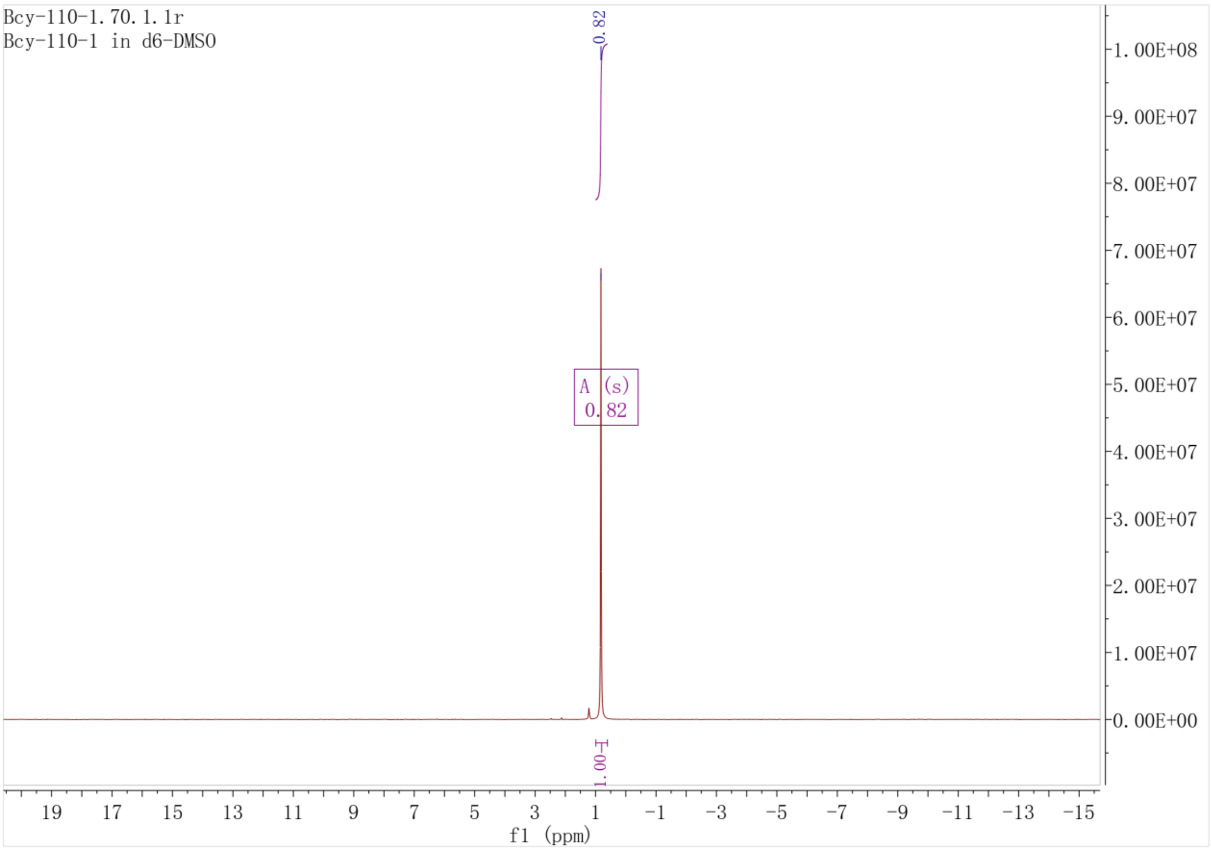

5 References

(1) Bi, C.; Mirza, S.; Baburi, H.; Schäkel, L.; Winzer, R.; Moschütz, S.; Keetz, K.; Lopez, V.; Pelletier, J.; Sévigny, J.; Schulze Zur Wiesch, J.; Claff, T.; Tolosa, E.; Namasivayam, V.; Sträter, N.; Müller, C. E. Synthesis, Characterization, Interactions, and Immunomodulatory Function of Ectonucleotidase CD39/CD73 Inhibitor 8-Butylthioadenosine 5′-Monophosphate. *ACS Pharmacol. Transl. Sci.* **2025**, *8* (5), 1401–1415. https://doi.org/10.1021/acsptsci.5c00126.

(2) Zebisch, M.; Krauss, M.; Schäfer, P.; Sträter, N. Crystallographic Evidence for a Domain Motion in Rat Nucleoside Triphosphate Diphosphohydrolase (NTPDase) 1. *J. Mol. Biol.* **2012**, *415* (2), 288–306. https://doi.org/10.1016/j.jmb.2011.10.050.

(3) Lee, S.-Y.; Luo, X.; Namasivayam, V.; Geiss, J.; Mirza, S.; Pelletier, J.; Stephan, H.; Sévigny, J.; Müller, C. E. Development of a Selective and Highly Sensitive Fluorescence Assay for Nucleoside Triphosphate Diphosphohydrolase1 (NTPDase1, CD39). *Analyst* **2018**, *143* (22), 5417–5430. https://doi.org/10.1039/C8AN01108G.

(4) Meng, E. C.; Goddard, T. D.; Pettersen, E. F.; Couch, G. S.; Pearson, Z. J.; Morris, J. H.; Ferrin, T. E. UCSF ChimeraX: Tools for Structure Building and Analysis. *Protein Sci.* **2023**, *32* (11), e4792. https://doi.org/10.1002/pro.4792.

(5) Niiya, K.; Thompson, R. D.; Silvia, S. K.; Olsson, R. A. 2-(*N*'-Aralkylidenehydrazino)Adenosines: Potent and Selective Coronary Vasodilators. *J. Med. Chem.* **1992**, *35* (24), 4562–4566. https://doi.org/10.1021/jm00102a008.

(6) Junker, A.; Renn, C.; Dobelmann, C.; Namasivayam, V.; Jain, S.; Losenkova, K.; Irjala, H.; Duca, S.; Balasubramanian, R.; Chakraborty, S.; Börgel, F.; Zimmermann, H.; Yegutkin, G. G.; Müller, C. E.; Jacobson, K. A. Structure–Activity Relationship of Purine and Pyrimidine Nucleotides as Ecto-5′-Nucleotidase (CD73) Inhibitors. *J. Med. Chem.* **2019**, *62* (7), 3677–3695. https://doi.org/10.1021/acs.jmedchem.9b00164.

(7) Lee, S.-Y.; Sarkar, S.; Bhattarai, S.; Namasivayam, V.; De Jonghe, S.; Stephan, H.; Herdewijn, P.; El-Tayeb, A.; Müller, C. E. Substrate-Dependence of Competitive Nucleotide Pyrophosphatase/Phosphodiesterase1 (NPP1) Inhibitors. *Front. Pharmacol.* **2017**, *8*. https://doi.org/10.3389/fphar.2017.00054.

(8) Lopez, V.; Lee, S.-Y.; Stephan, H.; Müller, C. E. Recombinant Expression of Ecto-Nucleotide Pyrophosphatase/Phosphodiesterase 4 (NPP4) and Development of a Luminescence-Based Assay to Identify Inhibitors. *Anal. Biochem.* **2020**, *603*, 113774. https://doi.org/10.1016/j.ab.2020.113774.

(9) Blacher, E.; Ben Baruch, B.; Levy, A.; Geva, N.; Green, K. D.; Garneau-Tsodikova, S.; Fridman, M.; Stein, R. Inhibition of Glioma Progression by a Newly Discovered CD38 Inhibitor. *Int. J. Cancer* **2015**, *136* (6), 1422–1433. https://doi.org/10.1002/ijc.29095.

(10) Ikehara, M.; Hitoshi, U. Studies of Nucleosides and Nucleotides. XXVI. Further Studies on the Chlorination of Inosine Derivatives with Dimethylformamide-Thionyl Chloride Complex. *Chem. Pharm. Bull.* **1965**, *13* (2), 221–223. https://doi.org/10.1248/cpb.13.221.

(11) Lettré, H.; Ballweg, H. Riboside einiger cytotoxischer Purinderivate. *Justus Liebigs Ann. Chem.* **1962**, *656* (1), 158–162. https://doi.org/10.1002/jlac.19626560125.

(12) Čechová, L.; Jansa, P.; Šála, M.; Dračínský, M.; Holý, A.; Janeba, Z. The Optimized Microwave-Assisted Decomposition of Formamides and Its Synthetic Utility in the Amination Reactions of Purines. *Tetrahedron* **2011**, *67* (5), 866–871. https://doi.org/10.1016/j.tet.2010.12.040.

(13) McLaughlin, L. W.; Piel, N.; Hellmann, T. Preparation of Protected Ribonucleosides Suitable for Chemical Oligoribonucleotide Synthesis. *Synthesis* **1985**, *1985* (3), 322–323. https://doi.org/10.1055/s-1985-31198.

(14) Katritzky, A. R.; Wu, J.; Rachwal, S.; Rachwal, B.; Macomber, D. W.; Smith, T. P. Preparation of 6-, 7- and 8-membered Sultams by Friedel-Crafts Cyclization of ω-Phenylalkanesulfamoyl Chlorides. *Org. Prep. Proced. Int.* **1992**. https://doi.org/10.1080/00304949209356228.

(15) Hampton, A.; Kappler, F.; Picker, D. Species- or Isozyme-Specific Enzyme Inhibitors. 4. Design of a Two-Site Inhibitor of Adenylate Kinase with Isozyme Selectivity. *J. Med. Chem.* **1982**, *25* (6), 638–644. https://doi.org/10.1021/jm00348a006.

(16) Paul, B.; Chen, M. F.; Paterson, A. R. P. Inhibitors of Nucleoside Transport. Structure-Activity Study Using Human Erythrocytes. *J. Med. Chem.* **1975**, *18* (10), 968–973. https://doi.org/10.1021/jm00244a003.

(17) Chen, L. S.; Sheppard, T. L. Synthesis and Hybridization Properties of RNA Containing 8-Chloroadenosine. *Nucleosides Nucleotides Nucleic Acids* **2002**, *21* (8–9), 599–617. https://doi.org/10.1081/NCN-120015071.

(18) Lin, T. S.; Cheng, J. C.; Ishiguro, K.; Sartorelli, A. C. Purine and 8-Substituted Purine Arabinofuranosyl and Ribofuranosyl Nucleoside Derivatives as Potential Inducers of the Differentiation of the Friend Erythroleukemia. *J. Med. Chem.* **1985**, *28* (10), 1481–1485. https://doi.org/10.1021/jm00148a018.

(19) Bhattarai, S.; Freundlieb, M.; Pippel, J.; Meyer, A.; Abdelrahman, A.; Fiene, A.; Lee, S.-Y.; Zimmermann, H.; Yegutkin, G. G.; Sträter, N.; El-Tayeb, A.; Müller, C. E. α,β-Methylene-ADP (AOPCP) Derivatives and Analogues: Development of Potent and Selective Ecto-5′-Nucleotidase (CD73) Inhibitors. *J. Med. Chem.* **2015**, *58* (15), 6248–6263. https://doi.org/10.1021/acs.jmedchem.5b00802.

(20) Holmes, R. E.; Robins, R. K. Purine Nucleosides. IX. The Synthesis of 9-β-D-Ribofuranosyl Uric Acid and Other Related 8-Substituted Purine Ribonucleosides1. *J. Am. Chem. Soc.* **1965**, *87* (8), 1772–1776. https://doi.org/10.1021/ja01086a028.

(21) Halbfinger, E.; Major, D. T.; Ritzmann, M.; Ubl, J.; Reiser, G.; Boyer, J. L.; Harden, K. T.; Fischer, B. Molecular Recognition of Modified Adenine Nucleotides by the P2Y1-Receptor. 1. A Synthetic, Biochemical, and NMR Approach. *J. Med. Chem.* **1999**, *42* (26), 5325–5337. https://doi.org/10.1021/jm990156d.

(22) Buenger, G. S.; Nair, V. Dideoxygenated Purine Nucleosides Substituted at the 8-Position: Chemical Synthesis and Stability. *Synthesis* **1990**, *1990* (10), 962–966. https://doi.org/10.1055/s-1990-27066.

(23) Gendron, F.-P.; Halbfinger, E.; Fischer, B.; Duval, M.; D'Orléans-Juste, P.; Beaudoin, A. R. Novel Inhibitors of Nucleoside Triphosphate Diphosphohydrolases: Chemical Synthesis and Biochemical and Pharmacological Characterizations. *J. Med. Chem.* **2000**, *43* (11), 2239–2247. https://doi.org/10.1021/jm000020b.

(24) Yoneda, K.; Iwamura, R.; Kishi, H.; Mizukami, Y.; Mogami, K.; Kobayashi, S. Identification of the Active Metabolite of Ticlopidine from Rat in Vitro Metabolites. *Br. J. Pharmacol.* **2004**, *142* (3), 551–557. https://doi.org/10.1038/sj.bjp.0705808.

(25) Moreau, C.; A. Ashamu, G.; C. Bailey, V.; Galione, A.; H. Guse, A.; L. Potter, B. V. Synthesis of Cyclic Adenosine 5′-Diphosphate Ribose Analogues: A C2′ Endo / Syn "Southern" Ribose Conformation Underlies Activity at the Sea Urchin cADPR Receptor. *Org. Biomol. Chem.* **2011**, *9* (1), 278–290. https://doi.org/10.1039/C0OB00396D.

(26) Storr, T. E.; Firth, A. G.; Wilson, K.; Darley, K.; Baumann, C. G.; Fairlamb, I. J. S. Site-Selective Direct Arylation of Unprotected Adenine Nucleosides Mediated by Palladium and Copper: Insights into the Reaction Mechanism. *Tetrahedron* **2008**, *64* (26), 6125–6137. https://doi.org/10.1016/j.tet.2008.01.062.

(27) Saladino, R.; Crestini, C.; Occhionero, F.; Nicoletti, R. Ozonation of Thionucleosides. A New Chemical Transformation of 4-Thiouracil and 6-Thioguanine Nucleosides to

Cytosine and Adenosine Counterparts. *Tetrahedron* **1995**, *51* (12), 3607–3616. https://doi.org/10.1016/0040-4020(95)00076-K.
